# What if I fail? Unsuccessful smoking cessation attempts and symptoms of depression and anxiety: a systematic review and meta-analysis

**DOI:** 10.1136/bmjopen-2024-091419

**Published:** 2025-05-02

**Authors:** Amy Bethan Crabb, Jennifer Allen, Gemma Taylor

**Affiliations:** 1 Psychology, University of Bath, Bath, UK; 2 University of Bath, Bath, Somerset, UK

**Keywords:** MENTAL HEALTH, Tobacco Use, Anxiety disorders, Depression & mood disorders, Systematic Review, PUBLIC HEALTH

## Abstract

**Abstract:**

**Objectives:**

Evidence that smoking cessation benefits physical and mental health has led to recommendations to support quitting. Unsuccessful quit attempts are common and associated with guilt and frustration; however, their impact on mental health is unclear. This review investigated the association between the success/failure of smoking cessation attempts and changes in symptoms of depression and anxiety.

**Design:**

Systematic review and meta-analysis, following Preferred Reporting Items for Systematic Reviews and Meta-Analyses (PRISMA) and Meta-analysis of Observational Studies in Epidemiology (MOOSE) guidelines.

**Data sources:**

Inclusion and exclusion lists of two previous reviews, plus update searches of Embase, Medline and PsycINFO (January 2020–January 2025).

**Eligibility criteria:**

Trials and longitudinal observational studies measuring symptoms of anxiety or depression before and after a smoking cessation attempt, beyond the withdrawal period (6 weeks), in adults who successfully quit and made an unsuccessful attempt.

**Data extraction and synthesis:**

Standardised methods were used for screening and data extraction. Two independent reviewers screened a minimum of 25% and extracted data for 100% of studies. Meta-analyses were conducted using random effects models, and narrative synthesis was used when necessary. Study quality, heterogeneity and publication bias were assessed using the adapted Newcastle-Ottawa Scale, *I*^*2*^ and funnel plots, respectively.

**Results:**

62 studies were included, representing 36 150 participants. Most featured behavioural smoking cessation interventions and defined successful cessation attempts by self-reported or biologically verified abstinence. Follow-up ranged from 6 weeks to 4 years. Overall, successfully quitting smoking was associated with reduced symptoms of depression (standardised mean difference (SMD)=–0.21, 95% CI –0.27 to –0.16) and anxiety (SMD=–0.22, 95% CI –0.33 to –0.12) compared with unsuccessful quit attempts. Heterogeneity was substantial *(I*^2^=50-69%).

**Conclusions:**

Most studies indicated a positive trend in alleviating symptoms of anxiety and depression during a quit attempt. Successful quitters experienced more substantial reductions in these symptoms compared with those who were unsuccessful. Importantly, those who made an unsuccessful quit attempt did not experience worse mental health.

**PROSPERO registration number:**

CRD42022314728.

**Implications:**

The majority of studies in our review indicated a positive trend in alleviating symptoms of anxiety and depression when individuals attempt to quit smoking. Successful quitters experienced more substantial reductions in these symptoms compared with those who were unsuccessful. Importantly, those who attempted to quit but failed did not experience worse mental health. These findings are relevant to people who smoke tobacco and the health professionals who support them as they may hold some apprehensions about quitting smoking or the anticipated emotional consequences of failing to quit. The current review contributes to clinical practice by adding to the information on which risk-benefit decisions are made regarding smoking cessation.

**STRENGTHS AND LIMITATIONS OF THIS STUDY**

- This review’s strengths lie in its comprehensive literature identification methods with broad search terms and inclusive screening criteria. Authors were contacted for additional data when not reported in a usable manner.
- There were very few major concerns regarding study quality, risk of bias or publication bias.
- Limitations include substantial heterogeneity and exclusion of studies due to data not reported by the analyses of interest.
- A more clinically relevant approach would involve comparing individuals attempting and failing to quit with those opting to continue smoking without attempting cessation, reflecting real-world decisions faced by smokers. However, limited studies are available using this comparison method.
- This review focused on depression and anxiety due to their close association with smoking. Other outcomes, such as post-traumatic stress, quality of life and suicidality, warrant further exploration in policies for combined smoking and mental health interventions.

## Introduction

 Smoking tobacco is a recognised major modifiable risk factor for global illness and death. In the UK, despite a decline from 20% in 2011 to 13% in 2021, smoking still incurs significant financial and health burdens.^1^ The associated risks include heightened cardiovascular disease, coronary heart disease, chronic obstructive pulmonary disease and an elevated risk of various cancers.^2^ Smoking-related illnesses contribute to 5% of the National Health Service budget annually, result in 50 million lost working days and cause approximately 100 000 deaths yearly.^3^

While the physical health benefits of smoking cessation are widely acknowledged, a common belief persists that smoking is beneficial for mental health, providing relief from anxiety and stress.^4 5^ The short-term alleviation of tobacco withdrawal symptoms, such as irritability, restlessness and mood changes, is often misattributed to an ability of smoking to improve mental health symptoms.^6^ However, research demonstrates that smoking cessation leads to improved mental health outcomes, including reduced depression, anxiety and stress, as well as an enhanced quality of life.^7^ Public health messaging and legislation changes have aligned with this evidence, dispelling the misconception that smoking benefits mental well-being.^8^,^11^

Individuals with common mental health difficulties, such as depression or anxiety, are over 50% more likely to smoke than the general population, facing challenges in quitting despite motivation.^12 13^ Preliminary evidence suggests that integrating smoking cessation support into mental health services is beneficial.^14^

Despite the known benefits, unsuccessful smoking cessation attempts are prevalent, with nearly 80% relapsing within 6 months.^15^ On average, it takes an estimated 30 attempts to successfully quit smoking for longer than 1 year.^16^ A quarter of people attempting to quit smoking report a fear of failure, which is generally associated with feelings of depression, anxiety, frustration, guilt and self-criticism.^5 17^ Attributing failure to personal qualities may discourage further quit attempts and result in learnt helplessness and shame associated with depression.^18^,^20^

Understanding the psychological consequences of failed attempts is crucial for health professionals to support individuals in making informed choices about smoking cessation. The current review explores the link between unsuccessful smoking cessation attempts and symptoms of depression and anxiety. Subgroup analyses consider follow-up duration and intervention types. The majority of relapse occurs within 6 months; therefore, mental health changes during this time may be considered more directly related to the quit attempt than later changes.^15 21^ Additionally, interventions with mood management components are expected to mitigate potential negative impacts of an unsuccessful quit attempt on mental health.

In conclusion, dispelling the misconception that smoking has a positive impact on mental health is essential. Recognising the psychological challenges of failed attempts and tailoring interventions accordingly could enhance overall cessation success.

## Methods

This review followed Preferred Reporting Items for Systematic Reviews and Meta-Analyses (PRISMA) and Meta-analysis of Observational Studies in Epidemiology (MOOSE) reporting guidelines.^22 23^ The protocol was registered with the International Prospective Register of Systematic Reviews.^24^

### Eligibility criteria

#### Population

The population of interest was adults (≥ 18 years) who had attempted to quit smoking tobacco during the study period. Participating in a smoking cessation trial was considered attempting to quit even if intention was not explicitly stated. There were no restrictions regarding population type; studies were included if participants were recruited from the general population or specific clinical groups.

#### Exposure

Studies were included that reported data on participants who had successfully quit smoking tobacco and those who made an unsuccessful smoking cessation attempt, as defined by the included studies. Studies were still included if, in addition to smoking tobacco, participants used electronic cigarettes or smoked other substances. Studies including participants who exclusively smoked electronic cigarettes were excluded.

#### Outcomes

Studies were included if they measured symptoms of anxiety or depression prior to quitting and at least 6 weeks after quitting using a standardised measure. The minimum follow-up of 6 weeks was chosen to exclude studies that reported data during the tobacco withdrawal period only. Self-report or clinician-rated measures were categorised as measuring symptoms of anxiety or depression consistent with a previous Cochrane review.^7^

#### Study design

The review included randomised controlled trials (RCTs) analysed by quit success/failure rather than the randomised treatment arms, non-randomised experimental studies, and longitudinal observational studies.

### Search methods

Inclusion and exclusion criteria for the initial stage of screening did not differ from two previous reviews^7 25^ (see online supplemental appendix A for search strategies). These reviews explored the relationships between quitting smoking and changes in various aspects of mental health, including symptoms of depression and anxiety, comparing people who successfully quit smoking with all continuing smokers, including people who experienced an unsuccessful quit attempt and those who did not attempt to quit smoking. Therefore, studies that were included in the previous reviews, or excluded at the full text screening stage, were screened for inclusion in the current review. Update searches of Embase, Medline and PsycINFO were conducted from 7 January 2020, the date of the most recent search, to 2 January 2025, to identify any new studies.

### Study selection

Titles and abstracts of all studies were screened by one researcher, a minimum of 25% were screened by a second reviewer. To maximise sensitivity, studies were initially included even if information regarding the measurement of anxiety or depression was not present in the abstract. The full texts of articles included at the title and abstract stage were examined by one researcher, and a minimum of 25% were screened by a second reviewer. Studies were not excluded based on language; non-English-language studies were translated. Abstract-only publications were included if they met inclusion criteria. Disagreements were resolved by discussion. Reasons for exclusion were recorded at the full-text screening stage (online supplemental appendix B). Covidence systematic review software was used for all stages.^26^

### Data extraction

For each study, two independent researchers extracted data. Any disagreements were resolved by discussion, involving a third researcher where necessary. Data were extracted pertaining to each study, including study design, data collection period, country, funding sources, interventions, definitions of a successful smoking cessation attempt, follow-up duration, population characteristics, baseline and follow-up participant numbers, age distribution, tobacco dependence measures, outcome measures for anxiety or depression, covariates adjusted for and information relevant to quality assessment. Means and measures of variance were extracted to calculate the standardised mean difference (SMD) for anxiety and depression outcomes. Where studies reported follow-up data at multiple time points, the latest follow-up in each of the following categories was extracted: (a) between 6 weeks and 6 months and (b) over 6 months.

#### Missing data

Where it was evident that relevant data had been collected but not reported, authors were contacted for additional data. If a study reported an analysis by the exposure of interest and it was not possible to obtain data for meta-analysis, this was reported narratively. If contact could not be made with the authors and an analysis by the exposure of interest was not reported in the main paper, the study was excluded.

### Meta-analyses

For each study outcome, the SMD was calculated, summarising the difference between successful and unsuccessful quit attempts, for change in symptoms of anxiety or depression from baseline to longest follow-up. The SMD is recommended in the *Cochrane Handbook for Systematic Reviews of Interventions* where different scales are used to measure the same construct.^27^ The SMD was calculated using standard formulae.^28 29^ Where SMD was calculated using mean at baseline and follow-up for each group, the correlation coefficient calculated for previous reviews was imputed.^25^ A generic inverse variance random effects model was used to pool SMDs for each outcome—anxiety and depression—using RevMan.^30^ An SMD less than zero indicated that an unsuccessful smoking cessation attempt was associated with greater symptoms of anxiety or depression compared with a successful attempt. The direction of change in symptoms of depression or anxiety from baseline to follow-up was examined to observe whether an unsuccessful quit attempt was associated with worsening mental health.

#### Subgroup analyses

Effect estimates and direction of change were compared for the following subgroups:

##### Assessment of quality and risk of bias

Studies which scored greater than 3 out of 5 on the adapted Newcastle-Ottawa Scale and studies which scored 3 .

##### Study design

Secondary analyses of RCTs, non-randomised experimental studies and observational studies.

##### Population type

Studies which recruited from the general population and specific clinical populations.

##### Length of follow-up

Studies with the longest follow-up between 6 weeks to 6 months, and longest follow-up longer than 6 months.

##### Intervention to support mood

Studies which did and did not include a mood management component in interventions delivered.

##### Definition of successful quit attempt

Studies with different definitions of a successful quit attempt.

##### Antidepressant medication

Studies which included medications classed as antidepressants, other pharmacological interventions such as varenicline or nicotine replacement therapy and no pharmacological intervention.

##### Leave-one-out

A leave-one-out analysis was conducted manually in RevMan^30^ to explore the impact of omitting a single study on effect estimates and heterogeneity.

### Assessment of heterogeneity

Heterogeneity was estimated using I^2^, the percentage of variability explained by differences between studies rather than sampling error. Consistent with the *Cochrane Handbook*,^27^ 50–90% was considered to represent substantial heterogeneity.

### Assessment of publication bias

Publication bias was assessed using Egger’s test and visual examination of funnel plots produced using RevMan.^30^,^32^

### Assessment of quality and risk of bias

For each study, quality and risk of bias were assessed by two independent reviewers, using the Newcastle-Ottawa quality assessment scale (NOS), adapted by Taylor and colleagues for use with studies measuring associations between smoking cessation and mental health^25^ (online supplemental table E1). The maximum rating for the adapted NOS is 5. Disagreements were resolved by discussion.

### Patient and public involvement

The focus of the current review, that is, the impact of an unsuccessful quit attempt on mental health, was identified through patient and public involvement work carried out for a previous review.^7^ This work aimed to identify outcomes of quitting smoking that were of particular relevance to the public.

## Results

### Description of studies

**Figure 1:**
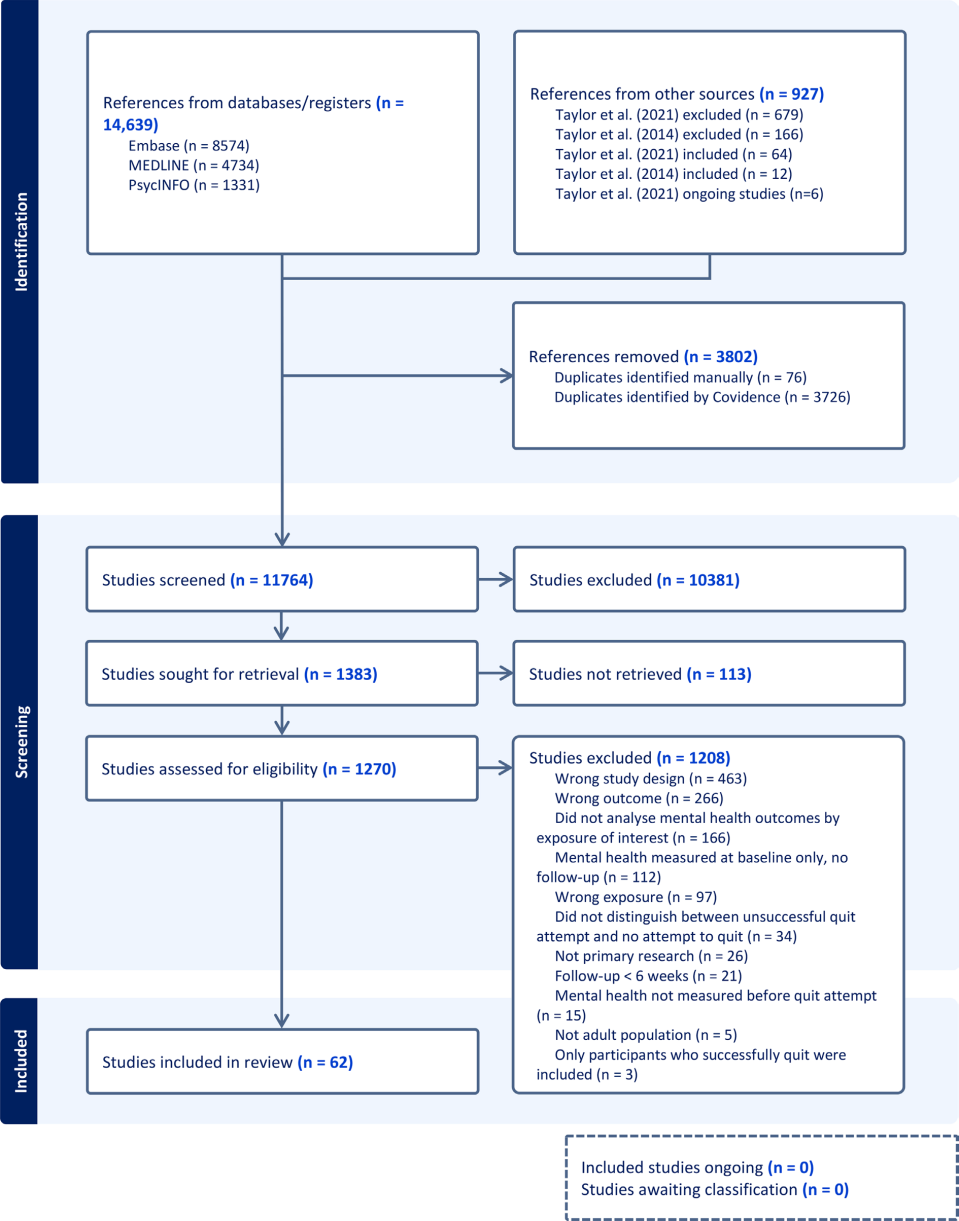
Preferred Reporting Items for Systematic Reviews and Meta-Analyses (PRISMA) flow diagram depicting the number of records identified, included and excluded at each stage of the review.

Search methods identified 11 764 unique studies, and 1270 full-text reports were reviewed (figure 1). Inter-rater reliability was 88% for titles and abstracts and 94% for full-text reports; the majority of disagreements occurred when the analyses of interest were not published, and it was unclear whether the data could be obtained by contacting the researchers.

62 studies were included in the current review (online supplemental appendix C); 59 measured symptoms of depression and 25 measured symptoms of anxiety; 49 were included in the meta-analyses and 13 were summarised narratively; 47 were secondary analyses of RCTs, 7 were non-randomised experimental studies and 8 were longitudinal observational studies. Length of follow-up ranged from 6 weeks to 4 years, the median and mode length of longest follow-up was 6 months. The most common outcome measures were the Beck Depression Inventory, Center for Epidemiological Studies Depression Scale, Patient Health Questionnaire, Hospital Anxiety and Depression Scale and GAD-7. Characteristics of included studies can be found in online supplemental appendix D.

#### Participants

The review included a total of 36 150 participants, with 21 282 included in meta-analyses. The pooled average age across studies was *M*=39.77 years (SD=10.70), the median gender proportion was 46% male, 54% female. At baseline, the pooled average number of cigarettes smoked per day was *M=*19.63 (SD=8.59) and the pooled average Fagerström test for nicotine dependence score was *M*=5.95 (SD=2.50), indicating a moderate dependence level overall.^33^

Studies recruited participants from a variety of populations; people diagnosed with a psychotic disorder (6), current or historic depression (5), HIV (5), ADHD (1), cancer (1) or type 2 diabetes (1); pregnant women (4); military veterans (4); people hospitalised for cardiovascular conditions (4); people attending homeless centres (1); postmenopausal women (1) and caregivers of children with asthma (1). In 28 studies, participants were recruited from the general population.

#### Definition of successful/unsuccessful quit attempts

The most common definition of a successful quit attempt was self-reported point-prevalence abstinence; not smoking for a specified period, for example, 7 days, prior to the follow-up assessment (47 studies). Continuous abstinence from quit date, or the end of a grace period, to follow-up was measured in 22 studies. 5 studies defined success by reduction in the number of cigarettes smoked or number of smoking days. More than one definition was reported in 26 studies. Self-reported abstinence was biologically verified through measurement of exhaled carbon monoxide levels, or cotinine concentration in urine or saliva, in 47 studies.

Some studies provided further specification for the definition of self-reported abstinence; in 15 studies, abstinence was defined as ‘not even a puff’, 2 studies included more lenient criteria, allowing up to 5 cigarettes during the period of continuous abstinence,^34 35^ and 2 studies defined abstinence according to criteria from the National Heart Lung and Blood Institute as never smoking for 7 or more consecutive days.^36 37^

Studies did not always explicitly define an unsuccessful quit attempt. Participants engaging in a smoking cessation intervention were considered to be making a smoking cessation attempt. An unsuccessful attempt was therefore defined by the absence of abstinence, that is, self-reported smoking within a specified period of time prior to the assessment or exceeding the threshold of exhaled carbon monoxide levels or cotinine concentration required for biological verification. In observational studies, an unsuccessful quit attempt was defined by smoking trajectories, that is, self-reported smoking at baseline, abstinence at an intermediary follow-up and smoking at a final follow-up.

#### Interventions

In 56 studies, participants engaged in a behavioural smoking cessation intervention, 43 studies included pharmacological smoking cessation treatments and 16 studies reported an intervention including a mood management component.

### Assessment of quality and risk of bias

On the adapted Newcastle-Ottawa Scale, 2 studies obtained a score of 1, 8 studies obtained a score of 2, 15 studies obtained a score of 3, 28 studies obtained a score of 4 and 9 studies obtained a score of 5 (online supplemental table E2). No studies obtained a score of 0. For each criterion, greater than 40% of studies included in the meta-analyses for depressive symptoms (online supplemental figure E1) and anxiety symptoms (online supplemental figure E2) were awarded a star.

### Assessment of publication bias

From visual examination of funnel plots, there was no clear asymmetry for studies included in the meta-analyses measuring depressive symptoms (online supplemental figure E3) or anxiety symptoms (online supplemental figure E4). Egger’s test identified weak evidence of small study bias for depressive symptoms, *p*=0.04 (online supplemental figure E5), and no evidence for anxiety symptoms, *p*=0.31 (online supplemental figure E6). For many of the included studies, the analyses of interest in this review were secondary to the primary aims of the study, and so would be less likely to influence publication.

### Change in mental health

#### Depression

**Figure 2:**
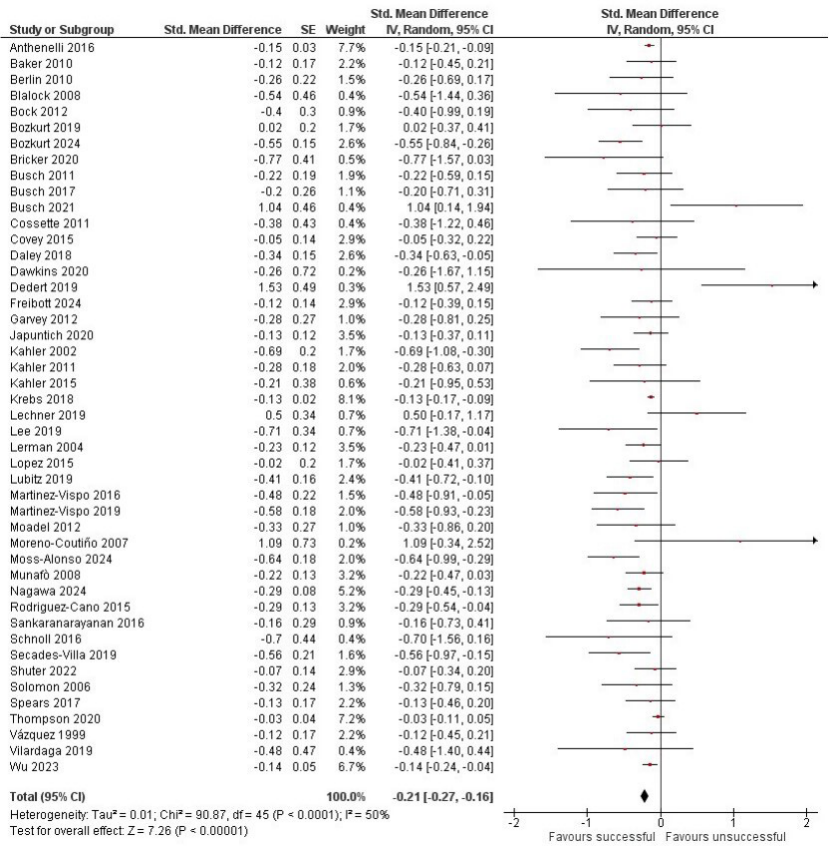
Difference between change in depressive symptoms from baseline to follow-up for people who successfully quit smoking after a quit attempt and those who made an unsuccessful quit attempt.

Sufficient data to calculate the SMD for change in symptoms of depression from baseline to follow-up was obtained for 46 studies. Data from 3412 people who successfully quit smoking and 16 674 people who made an unsuccessful quit attempt provided evidence that, overall, successfully quitting smoking was associated with a decrease in depressive symptoms compared with an unsuccessful quit attempt, SMD=−0.21, 95% CI −0.27 to −0.16, *p*<0.001 (figure 2). There was substantial heterogeneity between studies (*I*^2^=50%).

The direction of change in depressive symptoms from baseline to follow-up was examined. Data from 45 studies showed a reduction or no change in depressive symptoms during unsuccessful quit attempts; in 3 studies, this was reported as a statistically significant improvement. Data from 1 study suggested the possibility of worsening depressive symptoms (>1 SD) during an unsuccessful quit attempt; this was reported as significant up to 6 months, then non-significant at later follow-ups.

13 studies were synthesised narratively (online supplemental appendix F). 4 studies found no association between change in depressive symptoms from baseline to follow-up and cessation success/failure and did not report the direction of change for depressive symptoms; one of these studies reported that participants who smoked more cigarettes reported more depressive symptoms. 3 studies reported that depressive symptoms decreased from baseline to follow-up, regardless of the success or failure of the quit attempt. 2 studies reported a non-significant trend towards reduction of depressive symptoms for both successful and unsuccessful quit attempts. 2 studies reported an increase in depressive symptoms associated with an unsuccessful quit attempt compared with no change for successful quitters; however, the increase was considered marginal or not clinically meaningful. 2 studies reported that depressive symptoms remained unchanged in unsuccessful quitters compared with an improvement in successful quitters.

2 studies, included in the meta-analysis, reported on shared trajectories between depressive symptoms and quit success; successful quitters had reduced depressive symptoms compared with baseline at all follow-ups, unsuccessful quitters who experienced no success in quitting, classed as *smokers*, had stable depressive symptoms over all timepoints and unsuccessful quitters classed as *relapsers* had reduced depressive symptoms in line with initial cessation success, which returned to baseline levels on resuming smoking.^38 39^

Overall, the evidence suggested that a successful quit attempt was associated with reduced depressive symptoms compared with an unsuccessful quit attempt; however, an unsuccessful attempt was most often associated with either no change or a reduction in depressive symptoms compared with baseline.

#### Anxiety

**Figure 3:**
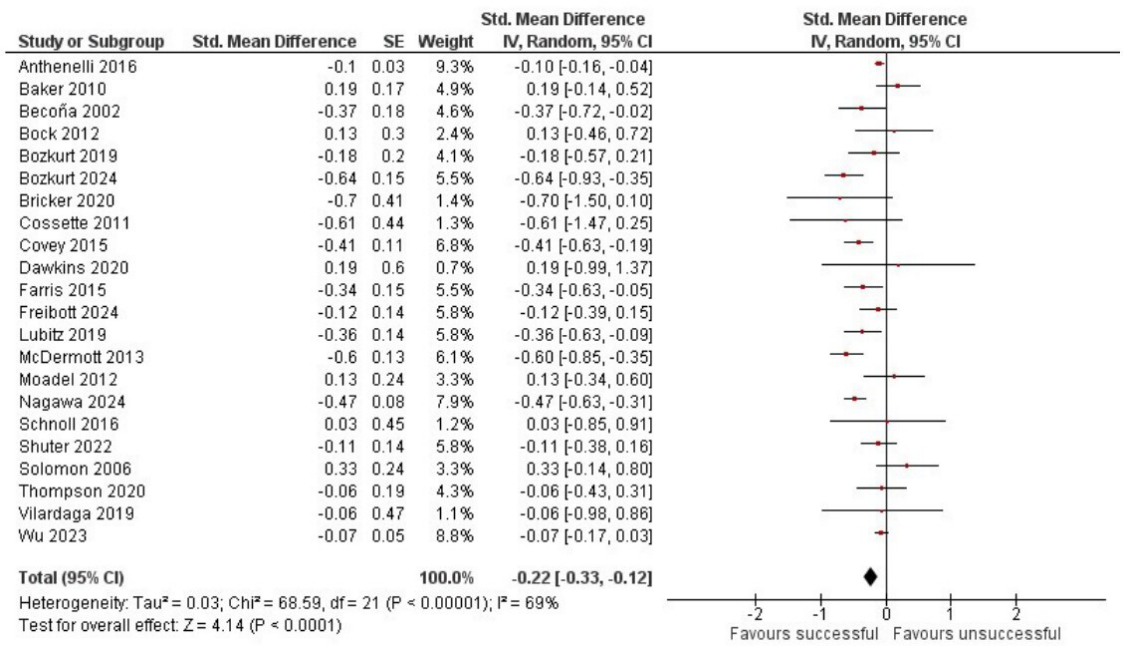
Difference between change in anxiety symptoms from baseline to follow-up for people who successfully quit smoking after a quit attempt and those who made an unsuccessful quit attempt.

Sufficient data to calculate the SMD for change in symptoms of anxiety from baseline to follow-up were obtained for 22 studies. Data from 2649 people who successfully quit smoking and 13 672 people who made an unsuccessful quit attempt provided evidence that, overall, successfully quitting smoking was associated with a decrease in anxiety symptoms compared with an unsuccessful quit attempt, SMD=−0.22, 95% CI −0.33 to −0.12, *p*<0.001 (figure 3). There was substantial heterogeneity between studies (*I*^2^=69%).

The direction of change in symptoms of anxiety from baseline to follow-up was examined. Data from all studies suggested a reduction or no change in anxiety symptoms during an unsuccessful quit attempt; in 1 study, this was reported as a statistically significant improvement.

3 studies were synthesised narratively (online supplemental appendix G). 1 study reported reduced anxiety symptoms for both successful and unsuccessful quitters, with ‘more pronounced’ changes for successful quitters. 1 study reported a reduction in anxiety symptoms for successful quitters compared with no change for unsuccessful quitters. 1 study reported no significant change for either successful or unsuccessful quitters, with a trend towards reduction in anxiety for successful quitters; women tended to report greater anxiety and depression symptoms than men, and sex differences were not significant.

Overall, the evidence suggested that a successful quit attempt was associated with reduced anxiety symptoms compared with an unsuccessful quit attempt; however, an unsuccessful attempt was most often associated with either no change or a reduction in anxiety symptoms compared with baseline.

#### Subgroup analyses

##### Assessment of quality and risk of bias

For depressive symptoms, there was no evidence of subgroup differences between studies which received a higher score (> 3) and studies which received a lower score (≤ 3) on the adapted Newcastle-Ottawa quality assessment scale (online supplemental table H1). Subgroup differences were observed for anxiety symptoms, although these were not significant (online supplemental table H2); for studies with scores of 3 or less, the overall effect was only borderline significant (*p*=0.07), and heterogeneity was higher (*I^2^*=74%) compared with studies scoring 4 or 5 (*I^2^*=59%).

##### Study design

There were no significant subgroup differences between studies of different designs for either depressive symptoms (online supplemental table H3) or anxiety symptoms (online supplemental table H4). For anxiety, the overall effect was mainly driven by secondary analyses of RCTs, possibly because there were few studies of other designs.

##### Population type

Where there were too few studies within each category to make a meaningful comparison, studies that recruited from specific populations were combined into one subgroup. For depressive symptoms, there was some evidence of weak subgroup effects across different clinical populations (*p*=0.03, *χ²*=15.15). The forest plot indicates that, in populations with HIV, psychotic disorders, veterans and ‘other’, the association crossed the null line, suggesting no clear direction of effect. In contrast, for other populations, the association favoured successful quitters, indicating better mental health outcomes in those who successfully quit smoking (online supplemental table H5). For anxiety symptoms, there was no significant difference between the general population and specific populations (online supplemental table H6).

##### Length of follow-up

For depressive symptoms, there was no evidence for subgroup differences between follow-ups longer than 6 months and those between 6 weeks and 6 months in length (online supplemental table H7). For anxiety symptoms, the overall effect was mainly driven by studies with a follow-up of 6 months or less; there were few studies with longer follow-ups (online supplemental table H8).

##### Definition of successful quit attempt

For depressive symptoms, there were no significant subgroup differences; for some definitions containing few studies, the association crossed the null line (online supplemental table H9). For anxiety symptoms, there was evidence of subgroup effects across different definitions (*p*=0.006, *χ²*=16.14). The forest plot indicates that, when a successful quit attempt was defined by self-reported smoking status, reduction or continuous abstinence, the association crossed the null line, suggesting no clear direction of effect (online supplemental table H10). In contrast, for studies defining a successful quit attempt by point-prevalence abstinence, with or without biological verification, the association favoured successful quitters.

##### Intervention to support mood

For depressive symptoms, narrative appraisals and meta-analyses revealed no evidence of subgroup differences between studies that did and did not include an intervention with a mood management component (online supplemental table H11). For anxiety symptoms, non-significant subgroup differences were observed, likely because very few studies included an intervention to support mood (online supplemental table H12).

##### Pharmacological intervention

For depressive symptoms, there was no evidence of subgroup differences between studies that included antidepressant medications, studies that included other pharmacological interventions such as varenicline or nicotine replacement therapy and studies with no pharmacological intervention (online supplemental table H13). For anxiety symptoms, the overall effect was mainly driven by studies that included pharmacological interventions, although subgroup differences were not significant (online supplemental table H14).

##### Leave-one-out

No single study had a disproportionate impact on effect estimates for either depressive symptoms (online supplemental table H15) or anxiety symptoms (online supplemental table H16). For depressive symptoms, leave-one-out analysis identified 2 studies disproportionately contributing to heterogeneity.^40 41^ Omitting both these studies reduced *I*^2^ from 50% to 38%. For anxiety symptoms, leave-one-out analysis identified 1 study disproportionately contributing to heterogeneity.^42^ Omitting this study reduced *I*^2^ from 69% to 62%.

### Certainty of evidence

The certainty of evidence, as assessed by Grading of Recommendations, Assessment, Development, and Evaluation criteria, was *low* for depressive symptoms and *very low* for anxiety symptoms (online supplemental appendix I). Both outcomes began with a *low* rating as studies were not randomised by the exposure of interest. The evidence for anxiety symptoms was downgraded due to substantial unexplained heterogeneity.

## Discussion

The majority of studies show that attempting to quit smoking improves symptoms of anxiety and depression, with successful quitters experiencing greater reductions than unsuccessful ones. Importantly, we found little evidence to suggest that those who attempted to quit but failed experienced worse mental health as a result. A successful quit attempt often leads to a sense of achievement, increased self-efficacy and improved mood.^43^ Unsuccessful attempts are generally associated with reduced or unchanged symptoms, potentially reassuring those fearing failure. Encouragingly, this implies that an unsuccessful quit attempt is unlikely to harm mental health compared with not attempting. Individuals typically undergo about 30 unsuccessful attempts before successfully quitting for over a year,^16^ emphasising the importance of persistence. While a few studies reported increased symptoms post-unsuccessful quit attempts, evidence on associated risk factors is limited. Future research should explore factors affecting mental health changes during unsuccessful quit attempts, aiding informed decision-making and risk minimisation.^44^

This review aimed to extend previous evidence on smoking cessation benefits for mental health by specifically comparing successful and unsuccessful quit attempts, excluding continuing smokers. A more clinically relevant approach would involve comparing individuals attempting and failing to quit with those opting to continue smoking without attempting cessation, reflecting real-world decisions faced by smokers. However, limited studies were found using this comparison method. Observational studies typically combined participants who had unsuccessfully attempted to quit smoking and participants who had not attempted to quit into a single category, even when they collected data on quit attempts. Most experimental studies compared cessation treatments, where all participants were considered to attempt quitting. Reporting data by quit attempts would enhance understanding of the specific impact on mental health and enable real-time associations between depression/anxiety symptoms and quit success. While follow-ups in many studies were tied to set time points, ecological momentary assessment, such as daily diaries, could provide more accurate tracking of associations between mental health symptoms and smoking behaviour, particularly lapses.^45^

The variation between studies suggests a nuanced relationship between smoking cessation success and mental health, beyond a direct link. Cognitive behavioural therapy principles indicate that an individual’s perception of an unsuccessful cessation attempt is crucial to their emotional response. For instance, interpreting it as a personal failure may lead to self-critical thoughts and increased depression or anxiety symptoms.^46^ Motivations to smoke and quit could influence this perception. McDermott *et al*^35^ found differing anxiety outcomes based on smoking motives, and Vázquez and Becoña^47^ observed a link between nicotine dependence and depressive symptoms for unsuccessful quitters. Investigating individual beliefs as a moderator could guide addressing unhelpful beliefs in smoking cessation interventions.

The majority of studies in this review defined successful quit attempts using abstinence measures, such as 7-day point-prevalence abstinence or complete abstinence with bio-verification. Four studies measured reduction in addition to other definitions.^3448^,^50^ One of these studies used ≥50% reduction as their primary definition due to low abstinence rates.^48^ Qualitative studies suggest a mismatch between researchers' and smokers' perceptions of success, with smokers more likely to define success in terms of reduction than abstinence; a co-production approach would benefit future research.^51^ Lechner *et al*^49^ proposed smoking reduction as a promising goal for those with depressive symptoms.

This review’s strength lies in its comprehensive literature identification methods with broad search terms and inclusive screening criteria, maximising sensitivity. Authors were contacted for additional data when not reported in a usable manner. Despite this, there remains a possibility that some relevant studies were not identified as it was not feasible for all studies to be screened by a second independent reviewer; 25% was chosen as a reasonable target based on other published reviews. There was a low risk of publication bias, and very few studies with major concerns regarding quality or risk of bias. Limitations of this review include substantial heterogeneity, some of which may be attributed to a few studies. The review was also limited by the types of data reported. Exclusion often resulted from mental health outcomes not analysed by cessation success/failure. Many studies collected relevant data but did not report the relevant analyses, as the primary focus was comparison of smoking cessation interventions. Many studies investigated mental health symptoms as predictors, not outcomes. The majority were RCTs, but participants were not randomised by quit attempt success/failure. Only two observational studies detailed smoking and depression trajectories, suggesting more research is needed. The review focused on depression and anxiety due to their close association with smoking.^12 13 52^ Other outcomes, like post-traumatic stress, quality of life and suicidality, identified through the review, warrant further exploration in policies for combined smoking and mental health interventions.

## Conclusion

The results of this review are consistent with the position that smoking cessation is beneficial for mental health and suggest that an unsuccessful cessation attempt is unlikely to be detrimental to mental health. These findings are relevant to people who smoke tobacco and the health professionals who support them as they may hold some apprehensions about quitting smoking or the anticipated emotional consequences of failing to quit. The current review contributes to clinical practice by adding to the information on which risk–benefit decisions are made regarding smoking cessation. Recommendations for future research include identifying factors that may be associated with a risk of worsening mental health during a quit attempt, such as considering the role of individual beliefs in the perception of failure. It will also be important for future research to map trajectories of smoking behaviour, including successful and unsuccessful quit attempts, relapse and symptoms of depression and anxiety over time, to better understand the patterns, risks and benefits of smoking cessation for mental health.

## Supplementary material

10.1136/bmjopen-2024-091419 online supplemental file 1

## Data Availability

Data are available upon reasonable request.

## References

[1] Office for National Statistics (2022). Adult smoking habits in the UK: 2021 [statistical bulletin]. https://www.ons.gov.uk/peoplepopulationandcommunity/healthandsocialcare/healthandlifeexpectancies/bulletins/adultsmokinghabitsingreatbritain/2021#:~:text=There%20has%20been%20a%20statistically,20.2%25%20of%20the%20population).

[2] U.S. Department of Health and Human Services (2014). The health consequences of smoking -- 50 years of progress: a report of the surgeon general.

[3] Ekpu VU, Brown AK (2015). The Economic Impact of Smoking and of Reducing Smoking Prevalence: Review of Evidence. Tob Use Insights.

[4] Perski O, Theodoraki M, Cox S (2022). Associations between smoking to relieve stress, motivation to stop and quit attempts across the social spectrum: A population survey in England. PLoS One.

[5] Tulloch HE, Pipe AL, Clyde MJ (2016). The Quit Experience and Concerns of Smokers With Psychiatric Illness. Am J Prev Med.

[6] Taylor GMJ, Baker AL, Fox N (2021). Addressing concerns about smoking cessation and mental health: theoretical review and practical guide for healthcare professionals. *BJPsych Adv*.

[7] Taylor GM, Lindson N, Farley A (2021). Smoking cessation for improving mental health. Cochrane Database Syst Rev.

[8] Department of Health (2017). Towards a smoke-free generation: a tobacco control plan for England. https://www.gov.uk/government/publications/towards-a-smoke-free-generation-tobacco-control-plan-for-england.

[9] NHS & Better Health (2020). Quit smoking. https://www.nhs.uk/better-health/quit-smoking/.

[10] Office for Health and Improvement Disparities (2022). Smoking and tobacco: applying all our health. https://www.gov.uk/government/publications/smoking-and-tobacco-applying-all-our-health/smoking-and-tobacco-applying-all-our-health.

[11] World Health Organization (2021). WHO report on the global tobacco epidemic 2021: addressing new and emerging products (p. 210). https://www.who.int/publications-detail-redirect/9789240032095.

[12] Richardson S, McNeill A, Brose LS (2019). Smoking and quitting behaviours by mental health conditions in Great Britain (1993-2014). Addict Behav.

[13] Smith PH, Chhipa M, Bystrik J (2020). Cigarette smoking among those with mental disorders in the US population: 2012-2013 update. Tob Control.

[14] Fredman Stein K, Sawyer K, Daryan S (2023). Service-user experiences of an integrated psychological intervention for depression or anxiety and tobacco smoking in improving access to psychological therapies services: A qualitative investigation into mechanisms of change in quitting smoking. Health Expect.

[15] Zhou X, Nonnemaker J, Sherrill B (2009). Attempts to quit smoking and relapse: factors associated with success or failure from the ATTEMPT cohort study. Addict Behav.

[16] Chaiton M, Diemert L, Cohen JE (2016). Estimating the number of quit attempts it takes to quit smoking successfully in a longitudinal cohort of smokers. BMJ Open.

[17] Johnson J, Panagioti M, Bass J (2017). Resilience to emotional distress in response to failure, error or mistakes: A systematic review. Clin Psychol Rev.

[18] Buczkowski K, Dachtera-Frąckiewicz M, Luszkiewicz D (2021). Reasons for and Scenarios Associated with Failure to Cease Smoking: Results from a Qualitative Study Among Polish Smokers Who Had Unsuccessfully Attempted to Quit. Patient Prefer Adherence.

[19] Kim S, Thibodeau R, Jorgensen RS (2011). Shame, guilt, and depressive symptoms: a meta-analytic review. Psychol Bull.

[20] Klein DC, Fencil-Morse E, Seligman ME (1976). Learned helplessness, depression, and the attribution of failure. J Pers Soc Psychol.

[21] West R, Hajek P, Stead L (2005). Outcome criteria in smoking cessation trials: proposal for a common standard. Addiction.

[22] Page MJ, McKenzie JE, Bossuyt PM (2021). The PRISMA 2020 statement: an updated guideline for reporting systematic reviews. BMJ.

[23] Brooke BS, Schwartz TA, Pawlik TM (2021). MOOSE Reporting Guidelines for Meta-analyses of Observational Studies. JAMA Surg.

[24] Crabb A, Taylor G, Allen J (2022). Failure to quit smoking and mental health: a systematic review and meta-analysis. PROSPERO: International Prospective Register of Systematic Reviews, CRD42022314728. https://www.crd.york.ac.uk/prospero/display_record.php?ID=CRD42022314728.

[25] Taylor G, McNeill A, Girling A (2014). Change in mental health after smoking cessation: systematic review and meta-analysis. BMJ.

[26] Veritas Health Innovation (2023). Covidence systematic review software [computer software]. www.covidence.org.

[27] Higgins J, Thomas J, Chandler J (2022). Cochrane handbook for systematic reviews of interventions (6.3).

[28] Viechtbauer W (2007). Approximate confidence intervals for standardized effect sizes in the two-independent and two-dependent samples design. J Educ Behav Stat.

[29] White IR, Thomas J (2005). Standardized mean differences in individually-randomized and cluster-randomized trials, with applications to meta-analysis. *Clin Trials*.

[30] The Cochrane Collaboration (2020). Review manager (RevMan) (5.4) [computer software].

[31] Egger M, Davey Smith G, Schneider M (1997). Bias in meta-analysis detected by a simple, graphical test. BMJ.

[32] Harbord RM, Egger M, Sterne JAC (2006). A modified test for small-study effects in meta-analyses of controlled trials with binary endpoints. Stat Med.

[33] Heatherton TF, Kozlowski LT, Frecker RC (1991). The Fagerström Test for Nicotine Dependence: a revision of the Fagerström Tolerance Questionnaire. Br J Addict.

[34] Dawkins L, Bauld L, Ford A (2020). A cluster feasibility trial to explore the uptake and use of e-cigarettes versus usual care offered to smokers attending homeless centres in Great Britain. PLoS One.

[35] McDermott MS, Marteau TM, Hollands GJ (2013). Change in anxiety following successful and unsuccessful attempts at smoking cessation: cohort study. Br J Psychiatry.

[36] Garvey AJ, Kalman D, Hoskinson RA (2012). Front-loaded versus weekly counseling for treatment of tobacco addiction. Nicotine Tob Res.

[37] Mathew AR, Robinson JD, Norton PJ (2013). Affective trajectories before and after a quit attempt among smokers with current depressive disorders. Nicotine Tob Res.

[38] Moss-Alonso E, Martínez-Vispo C, López-Durán A (2024). Does quitting smoking affect depressive symptoms? A longitudinal study based on treatment-seeking smokers with a history of depressive episode. Int J Ment Health Addiction.

[39] Rodríguez-Cano R, López-Durán A, del Río EF (2016). Smoking cessation and depressive symptoms at 1-, 3-, 6-, and 12-months follow-up. J Affect Disord.

[40] Dedert EA, Resick PA, Dennis PA (2019). Pilot Trial of a Combined Cognitive Processing Therapy and Smoking Cessation Treatment. J Addict Med.

[41] Thompson M, Schnoll R, Serrano K (2020). The effect of varenicline on mood and cognition in smokers with HIV. Psychopharmacology (Berl).

[42] Nagawa CS, Rigotti NA, Chang Y (2025). Association Between Smoking Abstinence and Depression and Anxiety Symptoms After Hospital Discharge: The Helping HAND 4 Trial. J Addict Med.

[43] Vangeli E, West R (2012). Transition towards a “non-smoker” identity following smoking cessation: an interpretative phenomenological analysis. Br J Health Psychol.

[44] Panagioti M, Khan K, Keers RN (2019). Prevalence, severity, and nature of preventable patient harm across medical care settings: systematic review and meta-analysis. BMJ.

[45] Shiffman S, Stone AA, Hufford MR (2008). Ecological momentary assessment. Annu Rev Clin Psychol.

[46] Beck A, Leahy R (2002). Clinical advances in cognitive psychotherapy: theory and application.

[47] Vázquez FL, Becoña E (1999). Depression and smoking in a smoking cessation programme. J Affect Disord.

[48] Baker A, Richmond R, Lewin TJ (2010). Cigarette smoking and psychosis: naturalistic follow up 4 years after an intervention trial. Aust N Z J Psychiatry.

[49] Lechner WV, Sidhu NK, Cioe PA (2019). Effects of time-varying changes in tobacco and alcohol use on depressive symptoms following pharmaco-behavioral treatment for smoking and heavy drinking. Drug Alcohol Depend.

[50] Sankaranarayanan A, Clark V, Baker A (2016). Reducing smoking reduces suicidality among individuals with psychosis: Complementary outcomes from a Healthy Lifestyles intervention study. Psychiatry Res.

[51] Pratt R, Xiong S, Kmiecik A (2022). The implementation of a smoking cessation and alcohol abstinence intervention for people experiencing homelessness. BMC Public Health.

[52] Levine MD, Cheng Y, Marcus MD (2016). Preventing Postpartum Smoking Relapse: A Randomized Clinical Trial. JAMA Intern Med.

