# Supplementary material for: What if I fail? Unsuccessful smoking cessation attempts and symptoms of depression and anxiety: a systematic review and meta-analysis
Source: BMJ Open. 2025 May 2;15(5):e091419. doi: 10.1136/bmjopen-2024-091419 (PMC12049876; doi:10.1136/bmjopen-2024-091419)
Supplement: online supplemental file 1 [file bmjopen-15-5-s001.docx]

**List of Appendices**

Appendix A: Search Strategies for Previous Reviews (Taylor et al., 2014, 2021)

Appendix B: References for Articles Excluded at Full Text Screening and Reasons for Exclusion

Appendix C: References for Included Studies

Appendix D: Characteristics of Included Studies, Ordered Alphabetically by Study ID

Appendix E: Assessment of Quality, Risk of Bias, and Publication Bias

Appendix F: Narrative Data Synthesis for Depressive Symptoms

Appendix G: Narrative Data Synthesis for Anxiety Symptoms

Appendix H: Subgroup Analyses

Appendix I: Certainty of Evidence, Assessed using the Grading of Recommendations, Assessment, Development, and Evaluation (GRADE) Criteria

**Appendix A**

Search Strategies for Previous Reviews (Taylor et al., 2014, 2021)

**Medline**

| 1. | exp "Tobacco Use Cessation"/ or exp Smoking Cessation/ |
| --- | --- |
| 2. | smoking cessation.mp. |
| 3. | ((reduc* or modif*) adj3 (smok* or cigar* or tobacco)).mp. |
| 4. | ((quit* or stop* or give* or cease) adj3 (smok* or cigar* or tobacco)).mp. |
| 5. | Harm Reduction/ or harm reduction.mp. |
| 6. | Smoking Reduction/ |
| 7. | tobacco consumption.mp. |
| 8. | cold turkey.mp. |
| 9. | Smoking Cessation Agents/ |
| 10. | "Tobacco Use Cessation Devices"/ |
| 11. | Electronic Nicotine Delivery Systems/ |
| 12. | 1 or 2 or 3 or 4 or 5 or 6 or 7 or 8 or 9 or 10 or 11 |
| 13. | Mental Health/ or mental health.mp. |
| 14. | Stress, Psychological/ |
| 15. | psychological health.mp. |
| 16. | Resilience, Psychological/ or psychological resilience.mp. |
| 17. | Anxiety/ or anxiety.mp. or Anxiety Disorders/ |
| 18. | anxious.mp. |
| 19. | Depression/ or depression.mp. |
| 20. | Depressive Disorders/ or depressive.mp. |
| 21. | Emotions/ or emotion*.mp. |
| 22. | psychological process$.mp. |
| 23. | mental hygiene.mp. |
| 24. | "Quality of Life"/ or quality of life.mp. |
| 25. | (well being or well?being).mp. |
| 26. | Affect/ or affect.mp. or Affective Symptoms/ |
| 27. | Adaptation, Psychological/ |
| 28. | 13 or 14 or 15 or 16 or 17 or 18 or 19 or 20 or 21 or 22 or 23 or 24 or 25 or 26 or 27 |
| 29. | 12 and 28 |
| 30. | limit 29 to yr="2011 -Current" |

**Embase**

| 1. | reduc$ smoking.mp. |
| --- | --- |
| 2. | exp Smoking Cessation/ or modified smoking.mp. |
| 3. | modified tobacco consumption.mp. |
| 4. | modification of cig$.mp. |
| 5. | modification of smoking.mp. |
| 6. | cigarette reduction.mp. |
| 7. | reduced cig$.mp. |
| 8. | reduction in cig$.mp. |
| 9. | harm reduction.mp. or exp Harm Reduction/ |
| 10. | reduced tobacco consumption.mp. |
| 11. | cold turkey.mp. |
| 12. | abrupt.mp. |
| 13. | smoking cessation.mp. |
| 14. | (quit$ smoking or stop$ smoking or give$ smoking or cease smoking).mp. |
| 15. | 1 or 2 or 3 or 4 or 5 or 6 or 7 or 8 or 9 or 10 or 11 or 12 or 13 or 14 |
| 16. | mental health.mp. or exp Mental Health/ |
| 17. | Well Being/ or Psychological Wellbeing/ |
| 18. | Psychological aspect/ or Psychological health/ or Mental stress/ or psychological well?being.mp. |
| 19. | exp "Hospital Anxiety and Depression Scale"/ or exp anxiety/ or exp Hamilton Anxiety Scale/ or exp State Trait Anxiety Inventory/ or exp Self-rating Anxiety Scale/ or anxiety.mp. or exp Beck Anxiety Inventory/ |
| 20. | exp Anxiety/ or Depression/ or anxious.mp. |
| 21. | depression inventory/ or exp "mixed anxiety and depression"/ or depression.mp. or exp Center for Epidemiological Studies Depression Scale/ or exp "Hospital Anxiety and Depression Scale"/ or exp Montgomery Asberg Depression Rating Scale/ or exp Self-rating Depression Scale/ or exp Beck Depression Inventory/ or exp long term depression/ or exp depression/ |
| 22. | depressive.mp. |
| 23. | emotion/ or psychological process$.mp. |
| 24. | mental hygiene.mp. or exp mental hygiene/ |
| 25. | quality of life.mp. or exp "Quality of Life"/ |
| 26. | (well?being or well being).mp. |
| 27. | exp Emotional States/ or affect.mp. |
| 28. | exp Emotion/ or emotion$.mp. |
| 29. | exp Emotional Disturbances/ or exp Emotional Adjustment/ or emotional problem$.mp. |
| 30. | psychological disturbance?.mp. |
| 31. | psychological problem$.mp. |
| 32. | 16 or 17 or 18 or 19 or 20 or 21 or 22 or 23 or 24 or 25 or 26 or 27 or 28 or 29 or 30 or 31 |
| 33. | 15 and 32 |
| 34. | limit 33 to yr="2011 -Current" |
| 35. | limit 34 to embase |

**PsycINFO**

| 1. | reduc$ smoking.mp. |
| --- | --- |
| 2. | exp Smoking Cessation/ or modified smoking.mp. |
| 3. | modified tobacco consumption.mp. |
| 4. | modification of cig$.mp. |
| 5. | modification of smoking.mp. |
| 6. | cigarette reduction.mp. |
| 7. | reduced cig$.mp. |
| 8. | reduction in cig$.mp. |
| 9. | (harm reduction.mp. or exp Harm Reduction/) and (smok* or cigar* or tobacco).mp. [mp=title, abstract, heading word, table of contents, key concepts, original title, tests & measures, mesh] |
| 10. | reduced tobacco consumption.mp. |
| 11. | cold turkey.mp. |
| 12. | abrupt cessation.mp. |
| 13. | smoking cessation.mp. |
| 14. | (quit$ smoking or stop$ smoking or give$ smoking or cease smoking).mp. |
| 15. | 1 or 2 or 3 or 4 or 5 or 6 or 7 or 8 or 9 or 10 or 11 or 12 or 13 or 14 |
| 16. | mental health.mp. or exp Mental Health/ |
| 17. | exp Well Being/ or psychological health.mp. |
| 18. | psychological well?being.mp. |
| 19. | exp Anxiety/ or anxiety.mp. or exp State Trait Anxiety Inventory/ or exp Taylor Manifest Anxiety Scale/ |
| 20. | exp Anxiety/ or anxious.mp. |
| 21. | depression.mp. or exp Beck Depression Inventory/ or exp "Depression (Emotion)"/ |
| 22. | exp "Depression (Emotion)"/ or depressive.mp. |
| 23. | psychological assessment/ or interview schedules/ or psychological report/ |
| 24. | mental hygiene.mp. |
| 25. | quality of life.mp. or exp "Quality of Life"/ |
| 26. | health/ or global health/ or holistic health/ or mental health/ |
| 27. | well?being.mp. |
| 28. | exp Emotional States/ or affect.mp. |
| 29. | emotion$.mp. or exp Emotions/ |
| 30. | exp Emotional Disturbances/ or exp Emotional Adjustment/ or emotional problem$.mp. |
| 31. | psychological disturbance?.mp. |
| 32. | psychological endurance/ or psychological stress/ or stress reactions/ |
| 33. | 16 or 17 or 18 or 19 or 20 or 21 or 22 or 23 or 24 or 25 or 26 or 27 or 28 or 29 or 30 or 31 or 32 |
| 34. | 15 and 33 |
| 35. | limit 34 to yr="2012 -Current" |

**Appendix B**

References for Articles Excluded at Full Text Screening and Reasons for Exclusion

| Reference | Reason for exclusion |
| --- | --- |
| Abrantes A. M., Browne J., Uebelacker L. A., Anderson B. J., Barter S., Shah Z., Kunicki Z. J., Caviness C., Price L. H., Desaulniers J., & Brown R. A. (2024). Randomized Controlled Trial of Aerobic Exercise for Smoking Cessation Among Individuals with Elevated Depressive Symptoms. *Nicotine and Tobacco Research*, *26*(5), 634-638. https://doi.org/10.1093/ntr/ntad201 | Did not analyse mental health outcomes by exposure of interest |
| Adkins-Hempel, M., Japuntich, S. J., Chrastek, M., Dunsiger, S., Breault, C. E., Ayenew, W., Everson-Rose, S. A., Nijjar, P. S., Bock, B. C., Wu, W.-C., Miedema, M. D., Carlson, B. M., & Busch, A. M. (2023). Integrated smoking cessation and mood management following acute coronary syndrome: Protocol for the post-acute cardiac event smoking (PACES) trial. *Addiction Science & Clinical Practice*, *18*(1). https://doi.org/10.1186/s13722-023-00388-9 | Did not analyse mental health outcomes by exposure of interest |
| Agterberg, S., Shuter, J., Stanton, C. A., Seng, E. K., & Weinberger, A. H. (2024). Race/ethnicity-based discrimination, depressive symptoms, and smoking-related variables among people with HIV participating in a randomized clinical trial for cigarette smoking cessation. *AIDS Care, 36*(12), 1781-1794. https://doi.org/10.1080/09540121.2024.2373403 | Did not analyse mental health outcomes by exposure of interest |
| Alfredsson, L., Klareskog, L., & Hedstrom, A. K. (2023). Influence of Smoking on Disease Activity and Quality of Life in Patients With Rheumatoid Arthritis: Results From a Swedish Case-Control Study With Longitudinal Follow-Up. *Arthritis Care and Research*, *75*(6), 1269-1277. https://doi.org/10.1002/acr.25026 | Wrong outcome |
| Anastasopoulou, S. V., Bonotis, K. S., Hatzoglou, C., Dafopoulos, K. C., & Gourgoulianis, K. I. (2022). Smoking Patterns and Anxiety Factors Among Women Expressing Perinatal Depression. Women’s Health Reports, 3(1), 198–206. https://doi.org/10.1089/whr.2021.0111 | Did not distinguish between unsuccessful quit attempt and no attempt to quit |
| Anghel, R., Adam, C. A., Marcu, D. T. M., Mitu, O., Roca, M., Tinica, G., & Mitu, F. (2022). Cardiac Rehabilitation in Peripheral Artery Disease in a Tertiary Center-Impact on Arterial Stiffness and Functional Status after 6 Months. Life (Basel, Switzerland), 12(4). https://doi.org/10.3390/life12040601 | Wrong outcome |
| Anonymous. (2022). Erratum: Sustained care smoking cessation intervention for individuals hospitalized for psychiatric disorders: The Helping HAND 3 randomized clinical trial (JAMA Psychiatry (2021) 78:8 (839-847) DOI: 10.1001/jamapsychiatry.2021.0707). JAMA Psychiatry, 79(1), 87. https://doi.org/10.1001/jamapsychiatry.2021.3312 | Not primary research |
| Aonso-Diego G., Gonzalez-Roz A., Krotter A., Garcia-Perez A., & Secades-Villa R. (2021). Contingency management for smoking cessation among individuals with substance use disorders: In-treatment and post-treatment effects. Addictive Behaviors, 119, 106920. https://doi.org/10.1016/j.addbeh.2021.106920 | Mental health measured at baseline only, no follow up |
| Apollonio D.E., Dutra L.M., & Glantz S.A. (2021). Associations between smoking trajectories, smoke-free laws and cigarette taxes in a longitudinal sample of youth and young adults. PLoS ONE, 16(2 February), e0246321. https://doi.org/10.1371/journal.pone.0246321 | Not adult population |
| Appleton, P. L. (2024). Smoking cessation and social support during pregnancy: A longitudinal study of the impact of close relationship support on smoking cessation in pregnancy. *Dissertation Abstracts International: Section B: The Sciences and Engineering*, *85*(8-B). | Wrong study design |
| Arana-Chicas, E., Cupertino, A. P., Goggin, K., Richter, K. P., Harris, K. J., & Catley, D. (2021). Stress, depression and quit attempt outcomes among unmotivated smokers. Substance Use & Misuse. https://doi.org/10.1080/10826084.2021.1936053 | Mental health measured at baseline only, no follow up |
| Arancini L, Bortolasci CC, Dodd S, Dean OM, & Berk M. (2019). N-acetylcysteine for cessation of tobacco smoking: Rationale and study protocol for a randomised controlled trial. Trials, 20(1), 555. https://doi.org/10.1186/s13063-019-3628-5 | Did not analyse mental health outcomes by exposure of interest |
| Armstrong, S. L. (2021). The association of religious coping and perceived stress with tobacco cessation. *Dissertation Abstracts International: Section B: The Sciences and Engineering*, *82*(1-B), No-Specified. | Not primary research |
| Asfar T., Koru-Sengul T., Annane D., McClure L.A., Perez A., Antoni M.A., Brewer J., & Lee D.J. (2021). Reach versus effectiveness: The design and protocol of randomized clinical trial testing a smartphone application versus in-person mindfulness-based smoking cessation intervention among young cancer survivors. Contemporary Clinical Trials Communications, 22, 100784. https://doi.org/10.1016/j.conctc.2021.100784 | Wrong study design |
| Asnaani, A., Kaczkurkin, A. N., Fitzgerald, H. E., Jerud, A., & Foa, E. B. (2020). The association between cognitive coping strategies and treatment outcomes in smokers with PTSD. Psychological Trauma : Theory, Research, Practice and Policy, 12(1), 92–100. https://doi.org/10.1037/tra0000473 | Wrong outcome |
| Audrain-McGovern, J., Rodriguez, D., Rodgers, K., & Cuevas, J. (2011). Declining alternative reinforcers link depression to young adult smoking. *Addiction*, *106*(1), 178–187. | Wrong exposure |
| Awaisu A, Haniki Nik Mohamed M, Noordin NM, Muttalif AR, Aziz NA, Syed Sulaiman SA, & Mahayiddin AA. (2012). Impact of connecting tuberculosis directly observed therapy short-course with smoking cessation on health-related quality of life. Tobacco Induced Diseases, 10(1), 2. https://doi.org/10.1186/1617-9625-10-2 | Did not analyse mental health outcomes by exposure of interest |
| Ayers CR, Heffner JL, Russ C, Lawrence D, McRae T, Evins AE, & Anthenelli RM. (2019). Efficacy and safety of pharmacotherapies for smoking cessation in anxiety disorders: Subgroup analysis of the randomized, active- and placebo-controlled EAGLES trial. Depression and Anxiety. https://doi.org/10.1002/da.22982 | Did not analyse mental health outcomes by exposure of interest |
| Azeem T., Khan S., & Samiullah F. (2024). Effects of smoking behaviour changes on depression in older people: A retrospective study. *Australasian Journal on Ageing*, *43*(2), 430-431. https://doi.org/10.1111/ajag.13327 | Not primary research |
| Babaoglu, A. B., Tozun, M., Padir, I., & Ertem, M. (2017). Some sociodemographic factors on smoking cessation rate in Konak smoking cessation outpatient clinic. Journal of Experimental and Clinical Medicine (Turkey), 34(1), 33–38. https://doi.org/10.5835/jecm.omu.33.04.007 | Mental health measured at baseline only, no follow up |
| Bailey, S. R., Voss, R., Angier, H., Huguet, N., Marino, M., Valenzuela, S. H., Chung-Bridges, K., & DeVoe, J. E. (2022). Affordable Care Act Medicaid expansion and access to primary-care based smoking cessation assistance among cancer survivors: An observational cohort study. BMC Health Services Research, 22(1), 488. https://doi.org/10.1186/s12913-022-07860-3 | Wrong outcome |
| Baker AL, Borland R, Bonevski B, Segan C, Turner A, Brophy L, McCarter K, Kelly PJ, Williams JM, Baird D, Attia J, Sweeney R, White SL, Filia S, & Castle D. (2019). ‘Quitlink’-A Randomized Controlled Trial of Peer Worker Facilitated Quitline Support for Smokers Receiving Mental Health Services: Study Protocol. *Frontiers in Psychiatry*, *10*, 124. | Wrong outcome |
| Baker A. L., McCarter K., Turner A., Segan C., Castle D., Brophy L., Borland R., Kelly P. J., Bonevski B., Baird D., Filia S., Attia J., Szwec S., Palazzi K., White S. L., Williams J. M., Wrobel A. L., Ireland A., Saxby K., … Sweeney R. (2024). ‘Quitlink’: Outcomes of a randomised controlled trial of peer researcher facilitated referral to a tailored quitline tobacco treatment for people receiving mental health services. *Australian and New Zealand Journal of Psychiatry*, *58*(3), 260-276. https://doi.org/10.1177/00048674231181039 | Wrong outcome |
| Baker AL, Richmond R, Kay-Lambkin FJ, Filia SL, Castle D, Williams JM, Lewin TJ, Clark V, Callister R, & Palazzi K. (2018). Randomised controlled trial of a healthy lifestyle intervention among smokers with psychotic disorders: Outcomes to 36months. Australian and New Zealand Journal of Psychiatry, 52(3), 239–252. https://doi.org/10.1177/0004867417714336 | Did not analyse mental health outcomes by exposure of interest |
| Baker, A. L., Richmond, R., Kay-Lambkin, F. J., Filia, S. L., Castle, D., Williams, J. M., Lewin, T. J., Clark, V., Callister, R., & Weaver, N. (2015). Randomized controlled trial of a healthy lifestyle intervention among smokers with psychotic disorders. Nicotine & Tobacco Research, 17(8), 946–954. https://doi.org/10.1093/ntr/ntv039 | Did not analyse mental health outcomes by exposure of interest |
| Balfour Louise, Wiebe Stephanie A, Cameron William D, Sandre Daniella, Pipe Andrew, Cooper Curtis, Angel Jonathan, Garber Gary, Holly Crystal, Dalgleish Tracy L, Tasca Giorgio A, & MacPherson Paul A. (2017). An HIV-tailored quit-smoking counselling pilot intervention targeting depressive symptoms plus Nicotine Replacement Therapy. AIDS Care, 29(1), 24–31. https://doi.org/10.1080/09540121.2016.1201195 | Did not analyse mental health outcomes by exposure of interest |
| Ballbe, M., Martinez, C., Feliu, A., Torres, N., Nieva, G., Pinet, C., Raich, A., Mondon, S., Barrio, P., Hernandez-Ribas, R., Vicens, J., Costa, S., Vilaplana, J., Alaustre, L., Vilalta, E., Blanch, R., Subira, S., Bruguera, E., Suelves, J. M., … Fernandez, E. (2019). Effectiveness of a telephone-based intervention for smoking cessation in patients with severe mental disorders: Study protocol for a randomized controlled trial. Trials, 20(1). https://doi.org/10.1186/s13063-018-3106-5 | Mental health not measured before quit attempt |
| Ban, K., Rogers, E., Khan, M., Scheidell, J., Charles, D., Bryant, K. J., Justice, A. C., Braithwaite, R. S., & Caniglia, E. C. (2024). Does smoking cessation reduce other substance use, psychiatric symptoms, and pain symptoms? Results from an emulated hypothetical randomized trial of US veterans. *PLoS ONE*, *19*(7). | Did not distinguish between unsuccessful quit attempt and no attempt to quit |
| Bandopadhyay T., Sen A., & Bhattacharya R. (2020). Depression, sexual dysfunction and medical comorbidities in young adults having nicotine dependence. *Indian Journal of Psychiatry*, *62*(7 Supplement 1), S21–S22. | Wrong study design |
| Banducci, A. N., Lejuez, C. W., & MacPherson, L. (2013). Pilot of a behavioral activation-enhanced smoking cessation program for substance users with elevated depressive symptoms in residential treatment. *Addict Newsl Am Psychol Div 50*, *2013*, 16–20. | Follow up < 6 weeks |
| Barbosa A., Costa A.R., Fontes F., Dias T., Pereira S., & Lunet N. (2019). Changes in health behaviours and body mass index after a breast cancer diagnosis: Results from a prospective cohort study. European Journal of Cancer Prevention, 28(4), 330–337. https://doi.org/10.1097/CEJ.0000000000000469 | Wrong study design |
| Barrio, P., Hidalgo-Mazzei, D., Ilzarbe, D., Fabrega, M., Moreno, J., Balcells, M., Mondon, S., & Gual, A. (2014). Motivational interviewing for smoking cessation in a psychiatric day hospital: A feasibility study. Journal of Dual Diagnosis, 10(4), 226–229. https://doi.org/10.1080/15504263.2014.961851 | Wrong outcome |
| Barroso-Hurtado, M., Suarez-Castro, D., Martinez-Vispo, C., Becona, E., & Lopez-Duran, A. (2023). Perceived Stress and Smoking Cessation: The Role of Smoking Urges. *International Journal of Environmental Research and Public Health, 20*(2). https://doi.org/10.3390/ijerph20021257 | Wrong outcome |
| Bashirian, S., Barati, M., Sharma, M., Abasi, H., & Karami, M. (2019). Water Pipe Smoking Reduction in the Male Adolescent Students: An Educational Intervention Using Multi-Theory Model. *Journal of Research in Health Sciences*, *19*(1), e00438. | Wrong outcome |
| Baskerville, W.-A., Friedman, T. C., Hurley, B., Hsieh, S., Dixon, T., Mtume, N., Lee, M. L., Rodriguez, L., Lopez, B., & Ray, L. A. (2022). Embedding comprehensive smoking cessation programs into community clinics: Study protocol for a cluster-randomized controlled trial. Trials, 23(1), 109. https://doi.org/10.1186/s13063-022-06023-3 | Not primary research |
| Baspinar, M. M., & Basat, O. (2022). Frequency and severity of irritable bowel syndrome in cigarette smokers, Turkey 2019. Tobacco Induced Diseases, 20(101201591), 27. https://doi.org/10.18332/tid/145925 | Wrong outcome |
| Bastian, L. A., Driscoll, M., DeRycke, E., Edmond, S., Mattocks, K., Goulet, J., Kerns, R. D., Lawless, M., Quon, C., Selander, K., Snow, J., Casares, J., Lee, M., Brandt, C., Ditre, J., & Becker, W. (2021). Pain and smoking study (PASS): A comparative effectiveness trial of smoking cessation counseling for veterans with chronic pain. Contemporary Clinical Trials Communications, 23(101671157), 100839. https://doi.org/10.1016/j.conctc.2021.100839 | Wrong study design |
| Battaglia C, Peterson J, Whitfield E, Min S-J, Benson SL, Maddox TM, & Prochazka AV. (2016). Integrating motivational interviewing into a home telehealth program for veterans with posttraumatic stress disorder who smoke: A randomized controlled trial. Journal of Clinical Psychology, 72(3), 194–206. https://doi.org/10.1002/jclp.22252 | Did not analyse mental health outcomes by exposure of interest |
| Bednarczuk N., Williams E.E., Absalom G., Olaitan-Salami J., & Greenough A. (2022). The impact of COVID-19 on smoking cessation in pregnancy. Journal of Perinatal Medicine, 50(7), 1001–1004. https://doi.org/10.1515/jpm-2022-0178 | Wrong outcome |
| Bellini B., Scholz J.R., Arnaut D., Abe T.M.O., Alberto R.L., Ferrari C.L., Jafet A.F., Teixeira M.J., & Marcolin M.A. (2020). Preliminary characteristic of 45 patient of the study placebo-controlled, randomized, double-blind clinical trial to assess the safety and efficacy of using Deep Transcranial Magnetic Stimulation (EMTp) in smoking cessation. Brain Stimulation, 13(6), 1844–1845. https://doi.org/10.1016/j.brs.2020.06.025 | Wrong outcome |
| Bellini B.B., Scholz J.R., Arnaut D., Alberto R.L., Ogawa T., Teixeira M.J., & Marcolin M. (2019). Deep transcranial magnetic stimulation for smoking cessation study: Partial results. Brain Stimulation, 12(2), 478–479. https://doi.org/10.1016/j.brs.2018.12.561 | Wrong study design |
| Bendixen, K., Brund, R. B. K., Jorgensen, T. B., Kristiansen, N. K., Kesmodel, U. S., Fonager, K., & Heuckendorff, S. (2023). Inequality in smoking and related risk factors for smoking in expectant mothers—A nationwide Danish register-based study. Scandinavian Journal of Public Health, dew, 100883503, 14034948221149758. https://doi.org/10.1177/14034948221149758 | Wrong study design |
| Bennett ME, Brown CH, Li L, Himelhoch S, Bellack A, & Dixon L. (2015). Smoking cessation in individuals with serious mental illness: A randomized controlled trial of two psychosocial interventions. Journal of Dual Diagnosis, 11(3–4), 161–173. https://doi.org/10.1080/15504263.2015.1104481 | Wrong outcome |
| Benson, L., Ra, C. K., Hebert, E. T., Kendzor, D. E., Oliver, J. A., Frank-Pearce, S. G., Neil, J. M., & Businelle, M. S. (2022). Quit Stage and Intervention Type Differences in the Momentary Within-Person Association Between Negative Affect and Smoking Urges. Frontiers in Digital Health, 4(101771889), 864003. https://doi.org/10.3389/fdgth.2022.864003 | Follow up < 6 weeks |
| Bernard P, Ninot G, Cyprien F, Courtet P, Guillaume S, Georgescu V, Picot M-C, Taylor A, & Quantin X. (2015). Exercise and counseling for smoking cessation in smokers with depressive symptoms: A randomized controlled pilot trial. Journal of Dual Diagnosis, 11(3–4), 205–216. https://doi.org/10.1080/15504263.2015.1113842 | Did not analyse mental health outcomes by exposure of interest |
| Bernard P, Ninot G, Guillaume S, Fond G, Courtet P, Picot MC, & et al. (2012). Physical activity as a protective factor in relapse following smoking cessation in participants with a depressive disorder. *The American Journal on Addictions*, *21*(4), 348–355. | Mental health measured at baseline only, no follow up |
| Bernstein, S. L., Rosner, J., & Toll, B. (2016). A Multicomponent Intervention Including Texting to Promote Tobacco Abstinence in Emergency Department Smokers: A Pilot Study. Academic Emergency Medicine, 23(7), 803–808. https://doi.org/10.1111/acem.12990 | Wrong outcome |
| Bick, D., Taylor, C., Avery, A., Bhavnani, V., Craig, V., Healey, A., Khazaezadeh, N., McMullen, S., Oki, B., Oteng-Ntim, E., O’Connor, S., Poston, L., Seed, P., Roberts, S., & Ussher, M. (2019). Protocol for a two-arm feasibility RCT to support postnatal maternal weight management and positive lifestyle behaviour in women from an ethnically diverse inner city population: The SWAN feasibility trial. Pilot and Feasibility Studies, 5(101676536), 117. https://doi.org/10.1186/s40814-019-0497-3 | Not primary research |
| Billingsley, B. E., Steinberg, M. L., & Amrhein, A. (2021). Motivational interviewing produces change talk in smokers with serious mental illness. Journal of Dual Diagnosis, 17(2), 151–158. https://doi.org/10.1080/15504263.2021.1896826 | Wrong outcome |
| Bioprojet. (2015). *Randomized Placebo Controlled Trial Assessing the Efficacy and Safety of BP1.4979 in Smoking Cessation*. clinicaltrials.gov. | Not primary research |
| Birke, H., Foxvig, I., Burns, K., Toft, U., Hansen, A. B. G., Hauge, P. I., Foghmar, S., Mindegaard, R. B., & Jakobsen, L. M. (2022). Heart Rehabilitation for All (HeRTA): Protocol for a feasibility study and pilot randomized trial. PloS One, 17(6), e0270159. https://doi.org/10.1371/journal.pone.0270159 | Not primary research |
| Bjornestad, E. D., Vederhus, J.-K., Clausen, T., & Abel, A. (2022). High smoking and low cessation rates among patients in treatment for opioid and other substance use disorders. BMC Psychiatry, 22. https://doi.org/10.1186/s12888-022-04283-6 | Wrong outcome |
| Blackwell, A. K. M., Daryan, S., Roy, D., Duffy, D., Hisler, G., Sawyer, K., Ainsworth, B., Richards, D., Hiscock, D., Papadakis, S., Brown, J., Munafo, M. R., Jacobsen, P., Aveyard, P., & Taylor, G. (2024). IntEgrating smoking cessation treAtment into usual online psychological care for people with common mEntal illness: Protocol for an online randomised feasibility and pilot study (ESCAPE digital). *Contemporary Clinical Trials*, *141*, 107541. https://doi.org/10.1016/j.cct.2024.107541 | Did not analyse mental health outcomes by exposure of interest |
| Blanquet M., Debost-Legrand A., Arnoult M., & Vendittelli F. (2019). Tobacco smoking relapse in post-partum period. European Journal of Obstetrics Gynecology and Reproductive Biology, 234, e93. https://doi.org/10.1016/j.ejogrb.2018.08.362 | Not primary research |
| Bloom, E. L., Oliver, J. A., Sutton, S. K., Brandon, T. H., Jacobsen, P. B., & Simmons, V. N. (2015). Post-operative smoking status in lung and head and neck cancer patients: Association with depressive symptomatology, pain, and fatigue. Psycho-Oncology, 24(9), 1012–1019. https://doi.org/10.1002/pon.3682 | Did not distinguish between unsuccessful quit attempt and no attempt to quit |
| Bloom, E. L., Ramsey, S. E., Abrantes, A. M., Hunt, L., Wing, R. R., Kahler, C. W., Molino, J., Brown, R. A., & Abrantes, A. (2020). A pilot randomized controlled trial of distress tolerance treatment for weight concern in smoking cessation among women. Nicotine & Tobacco Research, 22(9), 1578–1586. https://doi.org/10.1093/ntr/ntaa026 | Follow up < 6 weeks |
| Boehm, G., Schroeder, Y., & Schoberberger, R. (2015). Inpatient smoking cessation therapy: Truth or dare? Wiener Klinische Wochenschrift, 127(19–20), 786–791. https://doi.org/10.1007/s00508-015-0820-9 | Wrong outcome |
| Bogaerts, A. F. L., Devlieger, R., Nuyts, E., Witters, I., Gyselaers, W., & Van Den Bergh, B. R. H. (2013). Effects of lifestyle intervention in obese pregnant women on gestational weight gain and mental health: A randomized controlled trial. International Journal of Obesity, 37(6), 814–821. https://doi.org/10.1038/ijo.2012.162 | Wrong exposure |
| Boozary, L. K., Frank-Pearce, S. G., Alexander, A. C., Waring, J. J. C., Ehlke, S. J., Businelle, M. S., Cohn, A. M., Kendzor, D. E., & Abrams, A. (2021). Correlates of e-cigarette use among adults initiating smoking cessation treatment. Drug and Alcohol Dependence, 224. https://doi.org/10.1016/j.drugalcdep.2021.108724 | Mental health measured at baseline only, no follow up |
| Borges, A. M., Versella, M. V., Kibbey, M. M., Hall, S. M., Leyro, T. M., & Abrams. (2021). The interactive effect of anxiety sensitivity and negative smoking cessation cognitions on reductions in cigarette consumption during acute cessation. Addictive Behaviors, 117. https://doi.org/10.1016/j.addbeh.2021.106839 | Follow up < 6 weeks |
| Bouchet-Benezech, B., Champanet, B., & Rouzaud, P. (2018). Smoking cessation at the pharmacy: Feasibility and benefits based on a French observational study with six-month follow-up. *Substance Abuse & Rehabilitation*, *9*, 31–42. | Did not analyse mental health outcomes by exposure of interest |
| Boudreaux, E. D., O’Hea, E., Wang, B., Quinn, E., Bergman, A. L., Bock, B. C., & Becker, B. M. (2022). Modeling Health Event Impact on Smoking Cessation. Journal of Smoking Cessation, 2022(101478447), 2923656. https://doi.org/10.1155/2022/2923656 | Wrong outcome |
| Boulate, D., Fidelle, M., Caramella, C., Issard, J., Planche, O., Pradere, P., Garelik, D., Hache, O., Lamrani, L., Zins, M., Beaussier, H., Chatellier, G., Fadel, E., Zitvogel, L., Besse, B., & Mercier, O. (2022). Epidemiological Study to Assess the Prevalence of Lung Cancer in patients with smoking-associated atherosclerotic cardiovascular diseases: PREVALUNG study protocol. BMJ Open, 12(12), e067191. https://doi.org/10.1136/bmjopen-2022-067191 | Wrong study design |
| Bovill, M., Bar-Zeev, Y., Bonevski, B., Reath, J., Oldmeadow, C., Hall, A., Gould, G. S., I. C. A. N. Q. U. I. T. in Pregnancy Pilot Group, & Fredericks, B. (2021). Ngaa-bi-nya-nhumi-nya (to test first): Piloting the feasibility of using the Growth and Empowerment Measure with Aboriginal pregnant women who smoke. Journal of Smoking Cessation. https://doi.org/10.1155/2021/6610500 | Not adult population |
| Bovill, M., Chamberlain, C., Bennett, J., Longbottom, H., Bacon, S., Field, B., Hussein, P., Berwick, R., Gould, G., & O’Mara, P. (2021). Building an Indigenous-Led Evidence Base for Smoking Cessation Care among Aboriginal and Torres Strait Islander Women during Pregnancy and Beyond: Research Protocol for the Which Way? Project. International Journal of Environmental Research and Public Health, 18(3). https://doi.org/10.3390/ijerph18031342 | Not primary research |
| Bozkurt N., Altintas F., Bozkurt A.I., Turgut G., & Turgut S. (2019). Effect of mdr c3435t polymorphism on varenicline treatment in quit smoking. Brazilian Journal of Pharmaceutical Sciences, 55, e18186. https://doi.org/10.1590/s2175-97902019000118186 | Wrong outcome |
| Bozkurt, N., Uzun, S. U., Bozkurt, A. I., & Turgut, S. (2022). Does Cardiovascular Disease Risk Decrease after Smoking Cessation in Occupational Risk Groups?. Heart Views : The Official Journal of the Gulf Heart Association, 23(4), 208–214. https://doi.org/10.4103/heartviews.heartviews_67_22 | Mental health measured at baseline only, no follow up |
| Brady, B. R., Crane, T. E., O’Connor, P. A., Nair, U. S., Yuan, N. P., & Biener, B. (2019). Electronic cigarette use and tobacco cessation in a state-based quitline. Journal of Smoking Cessation, 14(3), 176–185. https://doi.org/10.1017/jsc.2019.2 | Wrong outcome |
| Brady, B. R., O’Connor, P. A., Martz, M. P., Grogg, T., & Nair, U. S. (2022). Medicaid-Insured Client Characteristics and Quit Outcomes at the Arizona Smokers’ Helpline. The Journal of Behavioral Health Services & Research, 49(1), 61–75. https://doi.org/10.1007/s11414-021-09756-2 | Wrong outcome |
| Brady, D. J., Phalen, P. L., Roche, D. J. O., Cowan, T., & Bennett, M. E. (2024). A reduction in cigarette smoking improves health-related quality of life and does not worsen psychiatric symptoms in individuals with serious mental illness. *Addictive Behaviors*, *151*, 1–5. https://doi.org/10.1016/j.addbeh.2023.107949 | Wrong outcome |
| Bricker, J. B., Levin, M., Lappalainen, R., Mull, K., Sullivan, B., & Santiago-Torres, M. (2021). Mechanisms of Smartphone Apps for Cigarette Smoking Cessation: Results of a Serial Mediation Model From the iCanQuit Randomized Trial. JMIR mHealth and uHealth, 9(11), e32847. https://doi.org/10.2196/32847 | Mental health measured at baseline only, no follow up |
| Brody, A. L., Zorick, T., Hubert, R., Hellemann, G. S., Balali, S., Kawasaki, S. S., Garcia, L. Y., Enoki, R., Abraham, P., Young, P., & McCreary, C. (2017). Combination extended smoking cessation treatment plus home visits for smokers with schizophrenia: A randomized controlled trial. Nicotine & Tobacco Research, 19(1), 68–76. https://doi.org/10.1093/ntr/ntw190 | Did not analyse mental health outcomes by exposure of interest |
| Brooks, J. M., & Mermelstein, R. J. (2022). Negative Affect and Cigarette Cessation in Dual Users of Cigarettes and Electronic Nicotine Delivery Systems. Substance Use & Misuse, 57(8), 1294–1302. https://doi.org/10.1080/10826084.2022.2079135 | Mental health measured at baseline only, no follow up |
| Brose, L. S., Brown, J., & McNeill, A. (2020). Mental health and smoking cessation-a population survey in England. BMC Medicine, 18(1), 161. https://doi.org/10.1186/s12916-020-01617-7 | Wrong study design |
| Brothers, B. M., & Borrelli, B. (2011). Motivating Latino smokers to quit: Does type of social support matter? *American Journal of Health Promotion*, *25*(5 Suppl), S96-102. | Did not analyse mental health outcomes by exposure of interest |
| Brown RA, Abrantes AM, Strong DR, Niaura R, Kahler CW, Miller IW, & Price LH. (2014). Efficacy of sequential use of fluoxetine for smoking cessation in elevated depressive symptom smokers. Nicotine & Tobacco Research, 16(2), 197–207. https://doi.org/10.1093/ntr/ntt134 | Did not analyse mental health outcomes by exposure of interest |
| Brown RA, Reed KM, Bloom EL, Minami H, Strong DR, Lejuez CW, Kahler CW, Zvolensky MJ, Gifford EV, & Hayes SC. (2013). Development and preliminary randomized controlled trial of a distress tolerance treatment for smokers with a history of early lapse. Nicotine & Tobacco Research, 15(12), 2005–2015. https://doi.org/10.1093/ntr/ntt093 | Wrong outcome |
| Brown, R. A., Minami, H., Hecht, J., Kahler, C. W., Price, L. H., Kjome, K. L., Bloom, E. L., Levy, D. E., Carpenter, K. M., Smith, A., Smits, J. A. J., Rigotti, N. A., & Abadie, A. (2021). Sustained care smoking cessation intervention for individuals hospitalized for psychiatric disorders: The Helping HAND 3 randomized clinical trial. JAMA Psychiatry, 78(8), 839–847. https://doi.org/10.1001/jamapsychiatry.2021.0707 | Did not analyse mental health outcomes by exposure of interest |
| Browne, J., Halverson, T. F., Vilardaga, R., & Barisic, B. (2021). Engagement with a digital therapeutic for smoking cessation designed for persons with psychiatric illness fully mediates smoking outcomes in a pilot randomized controlled trial. Translational Behavioral Medicine, 11(9), 1717–1725. https://doi.org/10.1093/tbm/ibab100 | Wrong outcome |
| Brunette M.F., Ferron J.C., Geiger P., & Villanti A.C. (2019). Menthol cigarette use in young adult smokers with severe mental illnesses. Nicotine and Tobacco Research, 21(5), 691–694. https://doi.org/10.1093/ntr/nty064 | Wrong outcome |
| Brunette, M. F., Ferron, J. C., McGurk, S. R., Williams, J. M., Harrington, A., Devitt, T., & Xie, H. (2020). Brief, Web-Based Interventions to Motivate Smokers With Schizophrenia: Randomized Trial. JMIR Mental Health, 7(2), e16524. https://doi.org/10.2196/16524 | Mental health measured at baseline only, no follow up |
| Brusadelli E., Tedone A., Galli F., Vegni E., Tomasich A., Mills J.G., & Borghi L. (2022). Psychological determinants of smoking cessation behaviors post group-intervention: A grounded theory explanatory model of long-term outcomes. Minerva Psychiatry, 63(2), 125–134. https://doi.org/10.23736/S2724-6612.21.02169-5 | Wrong study design |
| Bu, J., Young, K. D., Hong, W., Ma, R., Song, H., Wang, Y., Zhang, W., Hampson, M., Hendler, T., Zhang, X., & Amano, A. (2019). Effect of deactivation of activity patterns related to smoking cue reactivity on nicotine addiction. Brain: A Journal of Neurology, 142(6), 1827–1841. https://doi.org/10.1093/brain/awz114 | Mental health measured at baseline only, no follow up |
| Buckner, J. D., Zvolensky, M. J., Walukevich-Dienst, K., Lewis, E. M., Dean, K. E., Zielinski, M. H., & Audrain-McGovern, B. (2019). Integrated cognitive behavioral therapy for anxiety and smoking cessation. Clinical Case Studies, 18(5), 378–396. https://doi.org/10.1177/1534650119859094 | Wrong study design |
| Bui Q.T.H. & Nguyen A.T.D. (2020). Effectiveness of education intervention carried out by clinical pharmacist on quality of life of patients with COPD: A randomized controlled trial. Pharmaceutical Sciences Asia, 47(3), 238–245. https://doi.org/10.29090/psa.2020.03.019.0021 | Wrong outcome |
| Bullen C, Verbiest M, Galea-Singer S, Kurdziel T, Laking G, Newcombe D, Parag V, & Walker N. (2018). The effectiveness and safety of combining varenicline with nicotine e-cigarettes for smoking cessation in people with mental illnesses and addictions: Study protocol for a randomised-controlled trial. BMC Public Health, 18(1), 596. https://doi.org/10.1186/s12889-018-5351-7 | Wrong outcome |
| Burke, M. V., Ebbert, J. O., Schroeder, D. R., McFadden, D. D., & Hays, J. T. (2015). Treatment outcomes from a specialist model for treating tobacco use disorder in a medical center. Medicine (United States), 94(44), e1903. https://doi.org/10.1097/MD.0000000000001903 | Wrong outcome |
| Businelle, M. S., Garey, L., Gallagher, M. W., Hebert, E. T., Vujanovic, A., Alexander, A., Kezbers, K., Matoska, C., Robison, J., Montgomery, A., & Zvolensky, M. J. (2022). An Integrated mHealth App for Smoking Cessation in Black Smokers With Anxiety: Protocol for a Randomized Controlled Trial. JMIR Research Protocols, 11(5), e38905. https://doi.org/10.2196/38905 | Not primary research |
| Buttery, S. C., Williams, P., Mweseli, R., Philip, K. E. J., Sadaka, A., Bartlett, E. J., Devaraj, A., Kemp, S., Addis, J., Derbyshire, J., Chen, M., Morris, K., Laverty, A., & Hopkinson, N. S. (2022). Immediate smoking cessation support versus usual care in smokers attending a targeted lung health check: The QuLIT trial. BMJ Open Respiratory Research, 9(1). https://doi.org/10.1136/bmjresp-2021-001030 | Wrong outcome |
| Cano, M. T., Pennington, D. L., Reyes, S., Pineda, B. S., Llamas, J. A., Periyakoil, V. S., & Munoz, R. F. (2021). Factors associated with smoking in low-income persons with and without chronic illness. Tobacco Induced Diseases, 19(101201591), 59. https://doi.org/10.18332/tid/138241 | Wrong exposure |
| Cao, P., Smith, L., Mandelblatt, J. S., Jeon, J., Taylor, K. L., Zhao, A., Levy, D. T., Williams, R. M., Meza, R., & Jayasekera, J. (2022). Cost-Effectiveness of a Telephone-Based Smoking Cessation Randomized Trial in the Lung Cancer Screening Setting. JNCI Cancer Spectrum, 6(4). https://doi.org/10.1093/jncics/pkac048 | Wrong outcome |
| Caponnetto, P., Cibella, F., Mancuso, S., Campagna, D., Arcidiacono, G., & Polosa, R. (2011). Effect of a nicotine-free inhalator as part of a smoking-cessation programme. *European Respiratory Journal*, *38*(5), 1005–1011. | Mental health measured at baseline only, no follow up |
| Carl, E., Liskiewicz, A., Rivard, C., Alberico, R., Belal, A., Mahoney, M. C., Quisenberry, A. J., Bickel, W. K., Sheffer, C. E., & Abrams, A. (2020). Dosing parameters for the effects of high-frequency transcranial magnetic stimulation on smoking cessation: Study protocol for a randomized factorial sham-controlled clinical trial. BMC Psychology, 8. https://doi.org/10.1186/s40359-020-00403-7 | Not primary research |
| Carmody TP, McFall M, Saxon AJ, Malte CA, Chow B, Joseph AM, & et al. (2012). Smoking outcome expectancies in military Veteran smokers with posttraumatic stress disorder. Nicotine & Tobacco Research, 14(8), 919–926. https://doi.org/10.1093/ntr/ntr304 | Wrong outcome |
| Carney G., Bassett K., Maclure M., Taylor S., & Dormuth C.R. (2020). Cardiovascular and neuropsychiatric safety of smoking cessation pharmacotherapies in non-depressed adults: A retrospective cohort study. Addiction (Abingdon, England), 115(8), 1534–1546. https://doi.org/10.1111/add.14951 | Mental health not measured before quit attempt |
| Carpenter, K. M., Nash, C. M., Vargas-Belcher, R. A., Vickerman, K. A., & Haufle, V. (2019). Feasibility and early outcomes of a tailored quitline protocol for smokers with mental health conditions. Nicotine and Tobacco Research, 21(5), 584–591. https://doi.org/10.1093/ntr/ntz023 | Wrong outcome |
| Carrasco-Hernandez, L., Jodar-Sanchez, F., Nunez-Benjumea, F., Moreno Conde, J., Mesa Gonzalez, M., Civit-Balcells, A., Hors-Fraile, S., Parra-Calderon, C. L., Bamidis, P. D., & Ortega-Ruiz, F. (2020). A Mobile Health Solution Complementing Psychopharmacology-Supported Smoking Cessation: Randomized Controlled Trial. JMIR mHealth and uHealth, 8(4), e17530. https://doi.org/10.2196/17530 | Wrong outcome |
| Carroll, A. J., Huffman, M. D., Wileyto, E. P., Khan, S. S., Fox, E., Smith, J. D., Bauer, A.-M., Leone, F. T., Schnoll, R. A., & Hitsman, B. (2023). Change in cardiovascular health among adults with current or past major depressive disorder enrolled in intensive smoking cessation treatment. *Journal of Affective Disorders*, *333*, 527–534. https://doi.org/10.1016/j.jad.2023.04.089 | Wrong exposure |
| Carroll, A. J., Huffman, M. D., Zhao, L., Jacobs, D. R., Stewart, J. C., Kiefe, C. I., Brunner, W., Liu, K., & Hitsman, B. (2020). Associations between depressive symptoms, cigarette smoking, and cardiovascular health: Longitudinal results from CARDIA. Journal of Affective Disorders, 260, 583–591. https://doi.org/10.1016/j.jad.2019.09.049 | Did not distinguish between unsuccessful quit attempt and no attempt to quit |
| Carroll, A. J., Kim, K., Miele, A., Olonoff, M., Leone, F. T., Schnoll, R. A., Hitsman, B., & Anthenelli, B. (2019). Longitudinal associations between smoking and affect among cancer patients using varenicline to quit smoking. Addictive Behaviors, 95, 206–210. https://doi.org/10.1016/j.addbeh.2019.04.003 | Wrong outcome |
| Carroll, A. J., Mathew, A. R., Leone, F. T., Wileyto, E. P., Miele, A., Schnoll, R. A., Hitsman, B., & Anthenelli, A. (2020). Extended nicotine patch treatment among smokers with and without comorbid psychopathology. Nicotine & Tobacco Research, 22(1), 24–31. https://doi.org/10.1093/ntr/nty191 | Mental health measured at baseline only, no follow up |
| Carroll, D. M., & Cole, A. (2022). Racial/ethnic group comparisons of quit ratios and prevalences of cessation-related factors among adults who smoke with a quit attempt. The American Journal of Drug and Alcohol Abuse, 48(1), 58–68. https://doi.org/10.1080/00952990.2021.1977310 | Wrong study design |
| Cartujano-Barrera, F., Arana-Chicas, E., Ramirez-Mantilla, M., Perales, J., Cox, L. S., Ellerbeck, E. F., Catley, D., & Cupertino, A. P. (2019). ‘Every day I think about your messages’: Assessing text messaging engagement among Latino smokers in a mobile cessation program. Patient Preference and Adherence, 13(101475748), 1213–1219. https://doi.org/10.2147/PPA.S209547 | Wrong outcome |
| Casas, L., Medina-Ramirez, P., Carreno, V., Calixte-Civil, P., Martinez, U., Brandon, T. H., & Simmons, V. N. (2023). Hispanic/Latinx individuals’ attributions for abstinence and smoking: A content analysis of open-ended responses from a randomized cessation trial. *Addictive Behaviors Reports, 17,* 100478. https://doi.org/10.1016/j.abrep.2022.100478 | Wrong outcome |
| Cassidy, R. N., Tidey, J. W., Cao, Q., Colby, S. M., McClernon, F. J., Smith, T. T., Dermody, S., Koopmeiners, J. S., Jensen, J. A., Strayer, L. G., Donny, E. C., & Hatsukami, D. (2021). Responses to Gradual and Immediate Reduction of Nicotine in Cigarettes in Young Versus Older Adult Smokers. Nicotine & Tobacco Research, 23(9), 1559–1566. https://doi.org/10.1093/ntr/ntab049 | Wrong outcome |
| Castro-Conde A., Abeytua M., Arrarte Esteban V.I., Caravaca Perez P., Dalmau Gonzalez-Gallarza R., Garza Benito F., Hidalgo Urbano R.J., Torres Marques J., Vidal-Perez R., & Nunez-Gil I.J. (2021). Feasibility and results of an intensive cardiac rehabilitation program. Insights from the MxM (Mas por Menos) randomized trial. Revista Espanola de Cardiologia, 74(6), 518–525. https://doi.org/10.1016/j.recesp.2020.03.017 | Wrong exposure |
| Catley, D., Grobe, J., Moreno, J. L., Stortz, S., Fox, A. T., Bradley-Ewing, A., Richter, K. P., Resnicow, K., Harris, K. J., Goggin, K., & Aharonovich, A. (2021). Differential mechanisms of change in motivational interviewing versus health education for smoking cessation induction. Psychology of Addictive Behaviors, 35(7), 778–787. https://doi.org/10.1037/adb0000720 | Wrong exposure |
| Celikhisar, H., Dasdemir Ilkhan, G., & Irer, B. (2021). Effects of smoking cessation on sexual functions and health quality of life in premenopausal women: A prospective case—Controlled study. International Journal of Clinical Practice, 75(3), e13796. https://doi.org/10.1111/ijcp.13796 | Wrong outcome |
| Chai, Y., Luo, H., Yip, P. S. F., Perlman, C. M., & Hirdes, J. P. (2021). Factors Associated With Hospital Presentation of Self-Harm Among Older Canadians in Long-Term Care: A 12-Year Cohort Study. Journal of the American Medical Directors Association, 22(10), 2160-2168.e18. https://doi.org/10.1016/j.jamda.2020.12.022 | Wrong outcome |
| Chalati, W., Crilly, P., Fletcher, J., & Kayyali, R. (2020). A Comparative Study of the Cost and Uptake of Community Pharmacy ‘Stop Smoking and Emergency Contraception’ Services from the Perspective of the National Health Service. Journal of Research in Pharmacy Practice, 9(2), 73–87. https://doi.org/10.4103/jrpp.JRPP_20_35 | Did not analyse mental health outcomes by exposure of interest |
| Chalela, P., McAlister, A. L., Munoz, E., Despres, C., Akopian, D., Kaghyan, S., Fernandez, A., Sukumaran, P., & Ramirez, A. G. (2020). Reaching Latinos Through Social Media and SMS for Smoking Cessation. 187–196. https://doi.org/10.1007/978-3-030-29286-7 | Wrong outcome |
| Chang, C.-P., Huang, W.-H., You, C.-H., Hwang, L.-C., Lu, I.-J., & Chan, H.-L. (2019). Factors Correlated with Smoking Cessation Success in Older Adults: A Retrospective Cohort Study in Taiwan. International Journal of Environmental Research and Public Health, 16(18). https://doi.org/10.3390/ijerph16183462 | Wrong outcome |
| Charbonnier, J.-P., Pompe, E., Moore, C., Humphries, S., van Ginneken, B., Make, B., Regan, E., Crapo, J. D., van Rikxoort, E. M., Lynch, D. A., & COPDGene investigators. (2019). Airway wall thickening on CT: Relation to smoking status and severity of COPD. Respiratory Medicine, 146(8908438, rme), 36–41. https://doi.org/10.1016/j.rmed.2018.11.014 | Wrong outcome |
| Chen C., Anderson C.M., Babb S.D., Frank R., Wong S., Kuiper N.M., & Zhu S.-H. (2021). Evaluation of the Asian Smokers’ Quitline: A Centralized Service for a Dispersed Population. American Journal of Preventive Medicine, 60(3 Supplement 2), S154–S162. https://doi.org/10.1016/j.amepre.2020.01.033 | Mental health measured at baseline only, no follow up |
| Chen M. & Chen L. (2021). Promoting Smoking Cessation in China: Using an Expansion of the EPPM with Other-oriented Threat. Journal of Health Communication, 26(3), 174–183. https://doi.org/10.1080/10810730.2021.1906360 | Wrong study design |
| Chen, J., Chen, R., Xiang, S., Li, N., Gao, C., Wu, C., Zhang, Q., Zhao, Y., Liao, Y., Stewart, R., Xu, Y., Shi, Y., Li, Z., & Bowden, B. (2021). Cigarette smoking and schizophrenia: Mendelian randomisation study. The British Journal of Psychiatry, 218(2), 98–103. https://doi.org/10.1192/bjp.2020.116 | Wrong study design |
| Chen, L.-S., Baker, T. B., Miller, J. P., Bray, M., Smock, N., Chen, J., Stoneking, F., Culverhouse, R. C., Saccone, N. L., Amos, C. I., Carney, R. M., Jorenby, D. E., & Bierut, L. J. (2020). Genetic Variant in CHRNA5 and Response to Varenicline and Combination Nicotine Replacement in a Randomized Placebo-Controlled Trial. Clinical Pharmacology and Therapeutics, 108(6), 1315–1325. https://doi.org/10.1002/cpt.1971 | Wrong outcome |
| Cheng A.Q., Liu Z., Zhao L., Zhou X.M., Cui Z.Y., Qin R., Li J.X., Wei X.W., Xiao D., & Wang C. (2022). Effect evaluation of ‘Smoking cessation: Doctor first’program in China. Zhonghua Yi Xue Za Zhi, 102, 94–99. https://doi.org/10.3760/cma.j.cn112137-20211119-02582 | Wrong study design |
| Chengappa KNR, Perkins KA, Brar JS, Schlicht PJ, Turkin SR, Hetrick ML, Levine MD, & George TP. (2014). Varenicline for smoking cessation in bipolar disorder: A randomized, double-blind, placebo-controlled study. Journal of Clinical Psychiatry, 75(7), 765–772. https://doi.org/10.4088/JCP.13m08756 | Did not analyse mental health outcomes by exposure of interest |
| Cheung YT, Lam TH, Chan CHH, Ho KS, Fok WYP, Wang MP, & Li WHC. (2020). Brief handgrip and isometric exercise intervention for smoking cessation: A pilot randomized trial. Addictive Behaviors, 100, 106119. https://doi.org/10.1016/j.addbeh.2019.106119 | Wrong outcome |
| Cho J.-H., Kwon H.-M., Park S.-E., Jung J.-H., Han K.-D., Park Y.-G., Kim Y.-H., Rhee E.-J., & Lee W.-Y. (2020). Protective effect of smoking cessation on subsequent myocardial infarction and ischemic stroke independent of weight gain: A nationwide cohort study. PLoS ONE, 15(7 July), e0235276. https://doi.org/10.1371/journal.pone.0235276 | Wrong outcome |
| Cho, Y.-M., Kim, H.-R., Kang, M.-Y., Myong, J.-P., & Koo, J. W. (2019). Fixed night workers and failed smoking cessation. Journal of Occupational Medicine and Toxicology, 14(101245790), 23. https://doi.org/10.1186/s12995-019-0243-z | Wrong study design |
| Chockalingam, L., Pence, B., Frangakis, C. E., Ha, T. V., Latkin, C. A., Sripaipan, T., Quan, V. M., Go, V. F., & Calvo-Sanchez, C. (2019). The relationship between health-related variables and increases in smoking among recently diagnosed HIV+ people who inject drugs in Vietnam. Addictive Behaviors, 95, 118–124. https://doi.org/10.1016/j.addbeh.2019.03.008 | Wrong exposure |
| Chu, S., Liang, L., Jing, H., Zhang, D., & Tong, Z. (2020). Safety of varenicline as an aid to smoking cessation in professional drivers and its impact on driving behaviors: An observational cohort study of taxi drivers in Beijing. Tobacco Induced Diseases, 18(101201591), 45. https://doi.org/10.18332/tid/120935 | Wrong outcome |
| Ciccolo, J. T., Williams, D. M., Dunsiger, S. I., Whitworth, J. W., McCullough, A. K., Bock, B. C., Marcus, B. H., & Myerson, M. (2014). Efficacy of resistance training as an aid to smoking cessation: Rationale and design of the Strength to Quit study. Mental Health and Physical Activity, 7(2), 95–103. https://doi.org/10.1016/j.mhpa.2014.05.004 | Did not analyse mental health outcomes by exposure of interest |
| Cinciripini, P. M., Karam-Hage, M., Kypriotakis, G., Robinson, J. D., Rabius, V., Beneventi, D., Minnix, J. A., & Blalock, J. A. (2019). Association of a Comprehensive Smoking Cessation Program With Smoking Abstinence Among Patients With Cancer. JAMA Network Open, 2(9), e1912251. https://doi.org/10.1001/jamanetworkopen.2019.12251 | Mental health measured at baseline only, no follow up |
| Cinciripini, P. M., Kypriotakis, G., Green, C., Lawrence, D., Anthenelli, R. M., Minnix, J., Blalock, J. A., Beneventi, D., Morris, C., Karam-Hage, M., & Anthenelli, A. (2022). The effects of varenicline, bupropion, nicotine patch, and placebo on smoking cessation among smokers with major depression: A randomized clinical trial. Depression and Anxiety, 39(5), 429–440. https://doi.org/10.1002/da.23259 | Wrong outcome |
| Cioe, P. A., Mercurio, A. N., Lechner, W., Costantino, C. C., Tidey, J. W., Eissenberg, T., Kahler, C. W., & Ashare, B. (2020). A pilot study to examine the acceptability and health effects of electronic cigarettes in HIV-positive smokers. Drug and Alcohol Dependence, 206. https://doi.org/10.1016/j.drugalcdep.2019.107678 | Wrong outcome |
| Clair C., Augsburger A., Birrer P., Locatelli I., Schwarz J., Greub G., Zanchi A., Jacot-Sadowski I., & Puder J.J. (2020). Assessing the efficacy and impact of a personalised smoking cessation intervention among type 2 diabetic smokers: Study protocol for an open-label randomised controlled trial (DISCGO-RCT). BMJ Open, 10(11), e040117. https://doi.org/10.1136/bmjopen-2020-040117 | Wrong study design |
| Clark, M. E., Young, B., Bedford, L. E., das Nair, R., Robertson, J. F. R., Vedhara, K., Sullivan, F., Mair, F. S., Schembri, S., Littleford, R. C., & Kendrick, D. (2019). Lung cancer screening: Does pulmonary nodule detection affect a range of smoking behaviours?. Journal of Public Health (Oxford, England), 41(3), 600–608. https://doi.org/10.1093/pubmed/fdy158 | Did not analyse mental health outcomes by exposure of interest |
| Clark, V., Baker, A., Lewin, T., Richmond, R., Kay-Lambkin, F., Filia, S., Castle, D., Williams, J., & Todd, J. (2017). Self-Reported Reasons for Smoking: Predicting Abstinence and Implications for Smoking Cessation Treatments Among Those With a Psychotic Disorder. Journal of Dual Diagnosis, 13(1), 6–14. https://doi.org/10.1080/15504263.2016.1271489 | Wrong outcome |
| Clawson, A. H., Cole, A. B., Ruppe, N. M., Nwankwo, C. N., Blair, A. L., Berlin, K. S., Naifeh, M. M., & Anderson, A. (2022). Smoking across adolescence and adulthood with cardiovascular risk among American Indian peoples. Health Psychology. https://doi.org/10.1037/hea0001227 | Wrong exposure |
| Clyde, M., Pipe, A., Reid, R., Els, C., & Tulloch, H. (2019). A bidirectional path analysis model of smoking cessation self-efficacy and concurrent smoking status: Impact on abstinence outcomes. Addiction Biology, 24(5), 1034–1043. https://doi.org/10.1111/adb.12647 | Wrong outcome |
| Cobos-Campos R., Mar J., Apinaniz A., de Lafuente A.S., Parraza N., Aizpuru F., & Orive G. (2021). Cost-effectiveness analysis of text messaging to support health advice for smoking cessation. Cost Effectiveness and Resource Allocation, 19(1), 9. https://doi.org/10.1186/s12962-021-00262-y | Wrong study design |
| Coca-Martinez, M., Carli, F., & Gill, H. L. (2021). Multimodal Prehabilitation to Improve Quality of Life and Functional Capacity in Peripheral Arterial Disease: A Case Series. Archives of Rehabilitation Research and Clinical Translation, 3(3), 100139. https://doi.org/10.1016/j.arrct.2021.100139 | Did not distinguish between unsuccessful quit attempt and no attempt to quit |
| Cody, G. R., Wang, B., Link, A. R., Sherman, S. E., & Caudill-Slosberg, D. (2019). Characteristics of urban inpatient smokers with and without chronic pain: Foundations for targeted cessation programs. Substance Use & Misuse, 54(7), 1138–1145. https://doi.org/10.1080/10826084.2018.1563186 | Wrong study design |
| Cohn, A. M., Zhou, Y., Cha, S., Perreras, L., Graham, A. L., & Alkhaldi, A. (2019). Treatment engagement mediates the links between symptoms of anxiety, depression, and alcohol use disorder with abstinence among smokers registered on an internet cessation program. Journal of Substance Abuse Treatment, 98, 59–65. https://doi.org/10.1016/j.jsat.2018.11.001 | Mental health measured at baseline only, no follow up |
| Collins B.N., Lepore S.J., Winickoff J.P., & Sosnowski D.W. (2019). Parents’ self-efficacy for tobacco exposure protection and smoking abstinence mediate treatment effects on child cotinine at 12-month follow-up: Mediation results from the Kids Safe and Smokefree trial. Nicotine & Tobacco Research. https://doi.org/10.1093/ntr/ntz175 | Mental health measured at baseline only, no follow up |
| Collins, B. N., Nair, U. S., Davis, S. M., Rodriguez, D., & Akinbami, A. (2019). Increasing home smoking restrictions boosts underserved moms’ bioverified quit success. *American Journal of Health Behavior*, *43*(1), 50–56. | Did not analyse mental health outcomes by exposure of interest |
| Collins, B. N., Nair, U. S., DiSantis, K. I., Hovell, M. F., Davis, S. M., Rodriguez, D., Audrain-McGovern, J., & Abraham, A. (2020). Long-term results from the FRESH RCT: Sustained reduction of children’s tobacco smoke exposure. American Journal of Preventive Medicine, 58(1), 21–30. https://doi.org/10.1016/j.amepre.2019.08.021 | Mental health measured at baseline only, no follow up |
| Collins, S. E., Nelson, L. A., Stanton, J., Mayberry, N., Ubay, T., Taylor, E. M., Hoffmann, G., Goldstein, S. C., Saxon, A. J., Malone, D. K., Clifasefi, S. L., Okuyemi, K., HaRT-S Community Advisory Board, & Arnsten, B. (2019). Harm reduction treatment for smoking (HaRT-S): Findings from a single-arm pilot study with smokers experiencing chronic homelessness. Substance Abuse, 40(2), 229–239. https://doi.org/10.1080/08897077.2019.1572049 | Wrong outcome |
| Colston, D. C., Simard, B. J., Xie, Y., McLeod, M. C., Elliott, M. R., Thrasher, J. F., & Fleischer, N. L. (2021). The Association between Quitline Characteristics and Smoking Cessation by Educational Attainment, Income, Race/Ethnicity, and Sex. International Journal of Environmental Research and Public Health, 18(6). https://doi.org/10.3390/ijerph18063297 | Wrong exposure |
| Condinho M., Ramalhinho I., & Sinogas C. (2021). Smoking cessation at the community pharmacy: Determinants of success from a real-life practice. Pharmacy, 9(3), 143. https://doi.org/10.3390/pharmacy9030143 | Mental health measured at baseline only, no follow up |
| Conroy, H. E., Jacquart, J., Baird, S. O., Rosenfield, D., Davis, M. L., Powers, M. B., Frierson, G. M., Marcus, B. H., Otto, M. W., Zvolensky, M. J., Smits, J. A. J., & Ahluwalia, A.-M. (2020). Age and pre quit-day attrition during smoking cessation treatment. Cognitive Behaviour Therapy, 49(5), 361–373. https://doi.org/10.1080/16506073.2020.1751262 | Mental health measured at baseline only, no follow up |
| Convill J., Blackhall F., Yorke J., Faivre-Finn C., & Gomes F. (2022). The Role of Electronic Patient-Reported Outcome Measures in Assessing Smoking Status and Cessation for Patients with Lung Cancer. Oncology and Therapy, 10(2), 481–491. https://doi.org/10.1007/s40487-022-00210-7 | Wrong study design |
| Cooley M.E., Blonquist T.M., Hong F., Nayak M.M., Crouter S.E., Hayman L.L., Jaklitsch M.T., Emmons K.M., & Bueno R. (2019). The effect of a lifestyle risk reduction intervention on lifestyle adherence and health-related quality of life in nonsmall cell lung cancer survivors: Feasibility study outcomes. Psycho-Oncology, 28(4), 920–923. https://doi.org/10.1002/pon.5002 | Wrong outcome |
| Cooley, M. E., Sarna, L., Kotlerman, J., Lukanich, J. M., Jaklitsch, M., Green, S. B., & Bueno, R. (2009). Smoking cessation is challenging even for patients recovering from lung cancer surgery with curative intent. Lung Cancer, 66(2), 218–225. https://doi.org/10.1016/j.lungcan.2009.01.021 | Did not analyse mental health outcomes by exposure of interest |
| Cooney NL, Litt MD, Sevarino KA, Levy L, Kranitz LS, Sackler H, & Cooney JL. (2015). Concurrent alcohol and tobacco treatment: Effect on daily process measures of alcohol relapse risk. Journal of Consulting and Clinical Psychology, 83(2), 346–358. https://doi.org/10.1037/a0038633 | Mental health measured at baseline only, no follow up |
| Cooper J, Borland R, Yong HH, & Fotuhi O. (2016). The impact of quitting smoking on depressive symptoms: Findings from the International Tobacco Control Four-Country Survey. Addiction, 111(8), 1448–1456. https://doi.org/10.1111/add.13367 | Wrong outcome |
| Cooper M., Yaqub M., Hinds J.T., & Perry C.L. (2019). A longitudinal analysis of tobacco use in younger and older U.S. veterans. Preventive Medicine Reports, 16, 100990. https://doi.org/10.1016/j.pmedr.2019.100990 | Mental health measured at baseline only, no follow up |
| Cooperman, N. A., Rizvi, S. L., Hughes, C. D., Williams, J. M., & Abrams, A. (2019). Field test of a dialectical behavior therapy skills training-based intervention for smoking cessation and opioid relapse prevention in methadone treatment. Journal of Dual Diagnosis, 15(1), 67–73. https://doi.org/10.1080/15504263.2018.1548719 | Wrong outcome |
| Correa, J. B., Lawrence, D., McKenna, B. S., Gaznick, N., Saccone, P. A., Dubrava, S., Doran, N., & Anthenelli, R. M. (2021). Psychiatric Comorbidity and Multimorbidity in the EAGLES Trial: Descriptive Correlates and Associations With Neuropsychiatric Adverse Events, Treatment Adherence, and Smoking Cessation. Nicotine & Tobacco Research, 23(10), 1646–1655. https://doi.org/10.1093/ntr/ntab056 | Mental health measured at baseline only, no follow up |
| Correa-Fernandez, V., Wilson, W. T., Kyburz, B., O’Connor, D. P., Stacey, T., Williams, T., Lam, C. Y., & Reitzel, L. R. (2019). Evaluation of the Taking Texas Tobacco Free Workplace Program within behavioral health centers. Translational Behavioral Medicine, 9(2), 319–327. https://doi.org/10.1093/tbm/iby067 | Wrong exposure |
| Courtney, R. J., Bradford, D., Martire, K. A., Bonevski, B., Borland, R., Doran, C., Hall, W., Farrell, M., Siahpush, M., Sanson-Fisher, R., West, R., & Mattick, R. P. (2014). A randomized clinical trial of a financial education intervention with nicotine replacement therapy (NRT) for low socio-economic status Australian smokers: A study protocol. Addiction, 109(10), 1602–1611. https://doi.org/10.1111/add.12669 | Did not analyse mental health outcomes by exposure of interest |
| Crawford G, Weisbrot J, Bastian J, Flitter A, Jao NC, Carroll A, Kalhan R, Leone F, Hitsman B, & Schnoll R. (2019). Predictors of varenicline adherence among cancer patients treated for tobacco dependence and its association with smoking cessation. Nicotine & Tobacco Research, 21(8), 1135–1139. https://doi.org/10.1093/ntr/nty133 | Did not analyse mental health outcomes by exposure of interest |
| Creswell KG, Cheng Y, & Levine MD. (2015). A test of the stress-buffering model of social support in smoking cessation: Is the relationship between social support and time to relapse mediated by reduced withdrawal symptoms? Nicotine & Tobacco Research, 17(5), 566–571. https://doi.org/10.1093/ntr/ntu192 | Did not analyse mental health outcomes by exposure of interest |
| Cropsey, K. L., Bean, M. C., Haynes, L., Carpenter, M. J., Richey, L. E., & Ande, B. (2020). Delivery and implementation of an algorithm for smoking cessation treatment for people living with HIV and AIDS. AIDS Care, 32(2), 223–229. https://doi.org/10.1080/09540121.2019.1626340 | Mental health measured at baseline only, no follow up |
| Cui, M., Kimura, T., Ikehara, S., Dong, J. Y., Ueda, K., Kawanishi, Y., Iso, H., Japan, E., & Children’s Study, G. (2019). Prenatal tobacco smoking is associated with postpartum depression in Japanese pregnant women: The japan environment and children’s study. *Journal of Affective Disorders*, *264*, 76–81. | Wrong outcome |
| Dahal, R., Adhikari, K., Patten, S. B., & Kroenke, P., Tyler. (2020). Smoking cessation and improvement in mental health outcomes: Do people who quit smoking by switching to electronic cigarettes experience improvement in mental health? The Canadian Journal of Psychiatry / La Revue Canadienne de Psychiatrie, 65(7), 512–514. https://doi.org/10.1177/0706743720917775 | Wrong study design |
| Dahne, J., Wahlquist, A. E., Kustanowitz, J., Natale, N., Fahey, M., Graboyes, E. M., Diaz, V. A., & Carpenter, M. J. (2023). Behavioral activation-based digital smoking cessation intervention for individuals with depressive symptoms: Randomized clinical trial. *Journal of Medical Internet Research*, *25*. https://doi.org/10.2196/49809 | Wrong exposure |
| Dai S., Chan M.H.M., Kam R.K.T., Li A.M., Au C.T., & Chan K.C.-C. (2022). Monthly Motivational Interview Counseling and Nicotine Replacement Therapy for Smoking Parents of Pediatric Patients: A Randomized Controlled Trial. Frontiers in Pediatrics, 10, 798351. https://doi.org/10.3389/fped.2022.798351 | Wrong outcome |
| Daly A., McDonnell B., & Regan C. (2021). 438 Pregnant smokers’ confidence in quitting is related to their level of nicotine addiction. American Journal of Obstetrics and Gynecology, 224(2 Supplement), S278. https://doi.org/10.1016/j.ajog.2020.12.459 | Wrong study design |
| Darmon, S., Park, A., Lovejoy, L. A., Shriver, C. D., Zhu, K., & Ellsworth, R. E. (2022). Relationship between Cigarette Smoking and Cancer Characteristics and Survival among Breast Cancer Patients. International Journal of Environmental Research and Public Health, 19(7). https://doi.org/10.3390/ijerph19074084 | Wrong study design |
| Daumit G., Cather C., Dalcin A., Dickerson F., Wang N.-Y., Jerome G., Miller E., Appel L., McCann U., Gennusa J., Goldsholl S., Cook C., & Evins A.E. (2019). Trial of integrated tobacco smoking cessation, exercise and weight management in persons with serious mental illness. Schizophrenia Bulletin, 45(Supplement 2), S96–S97. https://doi.org/10.1093/schbul/sbz022.021 | Not primary research |
| Daumit, G. L., Evins, A. E., Cather, C., Dalcin, A. T., Dickerson, F. B., Miller, E. R. I., Appel, L. J., Jerome, G. J., McCann, U., Ford, D. E., Charleston, J. B., Young, D. R., Gennusa, J. V. I., Goldsholl, S., Cook, C., Fink, T., & Wang, N.-Y. (2023). Effect of a tobacco cessation intervention incorporating weight management for adults with serious mental illness: A randomized clinical trial. *JAMA Psychiatry*, *80*(9), 895–904. https://doi.org/10.1001/jamapsychiatry.2023.1691 | Wrong outcome |
| Davies, N. M., Taylor, A. E., Taylor, G. M., Itani, T., Jones, T., Martin, R. M., Munafo, M. R., Windmeijer, F., & Thomas, K. H. (2020). Varenicline versus nicotine replacement therapy for long-term smoking cessation: An observational study using the Clinical Practice Research Datalink. Health Technology Assessment (Winchester, England), 24(9), 1–46. https://doi.org/10.3310/hta24090 | Mental health measured at baseline only, no follow up |
| Davis A., Ngo H., & Coleman M. (2019). An evaluation of a pilot specialist smoking cessation clinic in a mental health setting. Australasian Psychiatry, 27(3), 275–278. https://doi.org/10.1177/1039856218816372 | Wrong outcome |
| Davis, J. M., Manley, A. R., Goldberg, S. B., Smith, S. S., & Jorenby, D. E. (2014). Randomized trial comparing mindfulness training for smokers to a matched control. Journal of Substance Abuse Treatment, 47(3), 213–221. https://doi.org/10.1016/j.jsat.2014.04.005 | Wrong outcome |
| Davoudi M, Omidi A, Sehat M, & Sepehrmanesh Z. (2017). The Effects of acceptance and commitment therapy on man smokers’ comorbid depression and anxiety symptoms and smoking cessation: A randomized controlled trial. *Addict Health*, *9*(3), 129–138. | Did not analyse mental health outcomes by exposure of interest |
| Dawkins, L., Powell, J. H., Pickering, A., Powell, J., & West, R. (2009). Patterns of change in withdrawal symptoms, desire to smoke, reward motivation and response inhibition across 3 months of smoking abstinence. Addiction, 104(5), 850–858. https://doi.org/10.1111/j.1360-0443.2009.02522.x | Did not distinguish between unsuccessful quit attempt and no attempt to quit |
| Dawson-Rose, C., Shehadeh, D., Hao, J., Barnard, J., Khoddam-Khorasani, L. L., Leonard, A., Clark, K., Kersey, E., Mousseau, H., Frank, J., Miller, A., Carrico, A., Schustack, A., & Cuca, Y. P. (2020). Trauma, substance use, and mental health symptoms in transitional age youth experiencing homelessness. Public Health Nursing (Boston, Mass.), 37(3), 363–370. https://doi.org/10.1111/phn.12727 | Wrong study design |
| De Bacquer, D., Jennings, C. S., Mirrakhimov, E., Lovic, D., Bruthans, J., De Smedt, D., Gotcheva, N., Dolzhenko, M., Fras, Z., Pogosova, N., Lehto, S., Hasan-Ali, H., Jankowski, P., Kotseva, K., De Backer, G., Wood, D., & Ryden, L. (2022). Potential for optimizing management of obesity in the secondary prevention of coronary heart disease. European Heart Journal. Quality of Care & Clinical Outcomes, 8(5), 568–576. https://doi.org/10.1093/ehjqcco/qcab043 | Wrong exposure |
| De Genna N.M., Qu Y., Cheng Y., Emery Tavernier R.L., Kolko R.P., & Levine M.D. (2022). Trajectories of return to cigarette smoking up to one year postpartum among people who quit smoking during pregnancy. Nicotine & Tobacco Research. https://doi.org/10.1093/ntr/ntac263 | Mental health measured at baseline only, no follow up |
| de Jesus, S. (2023). Smoking and exercise: Mechanisms and effects during simulated and genuine quit attempts. *Dissertation Abstracts International: Section B: The Sciences and Engineering*, *84*(2-B). | Wrong outcome |
| de Ruijter D., Hoving C., Evers S., Hudales R., de Vries H., & Smit E. (2019). An economic evaluation of a computer-tailored e-learning program to promote smoking cessation counseling guideline adherence among practice nurses. Patient Education and Counseling, 102(10), 1802–1811. https://doi.org/10.1016/j.pec.2019.07.015 | Wrong exposure |
| de-Arriba-Palomero, P., Sales-Sanz, M., Fuentemilla, E., Won-Kim, H. R., de-Arriba-Palomero, F., & Munoz-Negrete, F. J. (2019). Effectiveness of oral counselling for smoke cessation in Graves orbitopathy patients. Efectividad Del Consejo Medico Para Dejar de Fumar En Pacientes Con Orbitopatia de Graves., 94(7), 323–330. https://doi.org/10.1016/j.oftal.2019.03.008 | Wrong study design |
| DeAtley, T., Denlinger-Apte, R. L., Cioe, P. A., Colby, S. M., Cassidy, R. N., Clark, M. A., Donny, E. C., Tidey, J. W., & Anthenelli, B. (2020). Biopsychosocial mechanisms associated with tobacco use in smokers with and without serious mental illness. Preventive Medicine, 140. https://doi.org/10.1016/j.ypmed.2020.106190 | Wrong study design |
| Deeks, L. S., Kosari, S., Develin, A., Peterson, G. M., Naunton, M., & Bittoun, C. (2019). Smoking cessation and the general practice pharmacist. Journal of Smoking Cessation, 14(3), 186–189. https://doi.org/10.1017/jsc.2019.3 | Wrong outcome |
| Deilhes F., Rouquet R.M., Gall Y., Aquilina C., Paul C., & Konstantinou M.P. (2020). Profile of smoking dependency in hidradenitis suppurativa patients and smoking cessation outcomes. Journal of the European Academy of Dermatology and Venereology. https://doi.org/10.1111/jdv.16494 | Wrong outcome |
| Deliu Z., Zhang J., Pasquinelli M., Wang H., Weldon C., Liu L., Feldman L., & Huber M. (2019). P2.10-08 Association Between Smoking and Anxiety/Depression in Respiratory Tract Cancers. Journal of Thoracic Oncology, 14(10 Supplement), S787–S788. https://doi.org/10.1016/j.jtho.2019.08.1692 | Wrong exposure |
| Denlinger-Apte, R. L., Tidey, J. W., Koopmeiners, J. S., Hatsukami, D. K., Smith, T. T., Pacek, L. R., McClernon, F. J., & Donny, E. C. (2019). Correlates of support for a nicotine-reduction policy in smokers with 6-week exposure to very low nicotine cigarettes. Tobacco Control, 28(3), 352–355. https://doi.org/10.1136/tobaccocontrol-2018-054622 | Wrong study design |
| Dennis PA, Kimbrel NA, Dedert EA, Beckham JC, Dennis MF, & Calhoun PS. (2016). Supplemental nicotine preloading for smoking cessation in posttraumatic stress disorder: Results from a randomized controlled trial. Addictive Behaviors. 59:24-9, 2016 08, 59, 24–29. https://doi.org/10.1016/j.addbeh.2016.03.004 | Wrong outcome |
| Deutsch, C., Bock, B. C., Lantini, R., Walaska, K., Rosen, R. K., Fava, J. L., Jennings, E. G., Foster, R., & Flanagan, W. (2019). A text message delivered smoking cessation intervention: Design and rationale of the Text My Quit Study. Contemporary Clinical Trials, 81(101242342), 19–27. https://doi.org/10.1016/j.cct.2019.04.010 | Wrong study design |
| Deveci, B., Ozeke, O., Gul, M., Acar, B., Cetin, E. H. O., Burak, C., Cay, S., Topaloglu, S., Aras, D., & Ilkay, E. (2018). Impact of the radial versus femoral access for primary percutaneous intervention on smoking cessation rates: A paradoxus between the health related quality of life and smoking quitting? Cor et Vasa, 60(4), e381–e386. https://doi.org/10.1016/j.crvasa.2018.03.006 | Mental health measured at baseline only, no follow up |
| Di Meglio, A., Gbenou, A. S., Martin, E., Pistilli, B., Ligibel, J. A., Crane, T. E., Flaysakier, J.-D., Minvielle, E., Vanlemmens, L., Guenancia, C., Rigal, O., Fournier, M., Soulie, P., Mouret-Reynier, M.-A., Tarpin, C., Boiffard, F., Guillermet, S., Everhard, S., Martin, A.-L., … Vaz-Luis, I. (2021). Unhealthy behaviors after breast cancer: Capitalizing on a teachable moment to promote lifestyle improvements. Cancer, 127(15), 2774–2787. https://doi.org/10.1002/cncr.33565 | Wrong exposure |
| Diaz, M., Garcia, M., Vidal, C., Santiago, A., Gnutti, G., Gomez, D., Trapero-Bertran, M., Fu, M., & Lung Cancer Prevention LUCAPREV research group. (2021). Health and economic impact at a population level of both primary and secondary preventive lung cancer interventions: A model-based cost-effectiveness analysis. Lung Cancer, 159(b3u, 8800805), 153–161. https://doi.org/10.1016/j.lungcan.2021.06.027 | Wrong study design |
| Dickreuter, J., Schmoor, C., Bengel, J., Jahne, A., & Leifert, J. A. (2020). Efficacy of a short-term residential smoking cessation therapy versus standard outpatient group therapy ('START-Study’): Study protocol of a randomized controlled trial. Trials, 21(1), 562. https://doi.org/10.1186/s13063-020-04253-x | Wrong study design |
| Dickson-Spillmann M, Kraemer T, Rust K, & Schaub M. (2012). Group hypnotherapy versus group relaxation for smoking cessation: An RCT study protocol. BMC Public Health, 12, 271. https://doi.org/10.1186/1471-2458-12-271 | Did not analyse mental health outcomes by exposure of interest |
| Ding, H., Karunanithi, M., Ireland, D., McCarthy, L., Hakim, R., Phillips, K., Pradhan, R., Seah, E.-H., Bowman, R. V., Fong, K., Masel, P., & Yang, I. A. (2019). Evaluation of an innovative mobile health programme for the self-management of chronic obstructive pulmonary disease (MH-COPD): Protocol of a randomised controlled trial. BMJ Open, 9(4), e025381. https://doi.org/10.1136/bmjopen-2018-025381 | Wrong outcome |
| Dobbie, F., Miller, M., Kam, M. H. M., McKenna, A., Glen, C., & McCallum, A. (2022). DASHES Protocol: Development and Feasibility Testing of a Tailored Community Programme to Support People in Recovery from Problematic Alcohol and Drug Use to Cut Down or Stop Smoking Using Co-Creation. International Journal of Environmental Research and Public Health, 19(20). https://doi.org/10.3390/ijerph192013709 | Wrong study design |
| Doherty, L. K. (2022). Electronic cigarette use among pregnant cigarette smokers in the Quit4Baby trial: Predictive factors of use, the efficacy of e-cigarettes for smoking cessation, and associations between combined cigarette and e-cigarette use and the risk of adverse neonatal outcomes. *Dissertation Abstracts International: Section B: The Sciences and Engineering*, *83*(10-B), No-Specified. | Wrong study design |
| Doran, N., Dubrava, S., & Anthenelli, R. M. (2019). Effects of varenicline, depressive symptoms, and region of enrollment on smoking cessation in depressed smokers. Nicotine and Tobacco Research, 21(2), 156–162. https://doi.org/10.1093/ntr/nty033 | Mental health measured at baseline only, no follow up |
| Drake, L. A., Suresh, K., Chrastil, H., Lewis, C. L., & Altman, R. L. (2022). Improving Tobacco Cessation Rates Using Inline Clinical Decision Support. Applied Clinical Informatics, 13(5), 1116–1122. https://doi.org/10.1055/a-1961-9800 | Wrong study design |
| DRKS00016860. (2019). Effectiveness of a self-help approach (Imaginal Retraining) to reduce cigarette consumption. *Http://Www.Who.Int/Trialsearch/Trial2.Aspx?TrialID=DRKS00016860*. | Did not analyse mental health outcomes by exposure of interest |
| Druckrey-Fiskaaen, K. T., Furulund, E., Daltveit, J. T., Vold, J. H., Lid, T. G., Madebo, T., Fadnes, L. T., ATLAS4LAR Study Group, & Buljovcic VB, F. T. (2022). Integration of smoking cessation into standard treatment for patients receiving opioid agonist therapy who are smoking tobacco: Protocol for a randomised controlled trial (ATLAS4LAR). Trials, 23(1), 663. https://doi.org/10.1186/s13063-022-06560-x | Wrong outcome |
| Du, X., Wu, R., Kang, L., Zhao, L., & Li, C. (2022). Tobacco smoking and depressive symptoms in Chinese middle-aged and older adults: Handling missing values in panel data with multiple imputation. Frontiers in Public Health, 10(101616579), 913636. https://doi.org/10.3389/fpubh.2022.913636 | Mental health not measured before quit attempt |
| Duffy, S. A., Ronis, D. L., Karvonen-Gutierrez, C. A., Ewing, L. A., Dalack, G. W., Smith, P. M., Carmody, T. P., Hicks, T., Hermann, C., Reeves, P., & Flanagan, P. (2014). Effectiveness of the tobacco tactics program in the Department of Veterans Affairs. Annals of Behavioral Medicine, 48(2), 265–274. https://doi.org/10.1007/s12160-014-9605-z | Did not analyse mental health outcomes by exposure of interest |
| Dulger S., Dilektasli E., & Sayan H.E. (2019). Evaluation of smoking cessation program results in obesity surgery patients. Turkish Thoracic Journal, 20(Supplement 1), S111. https://doi.org/10.5152/TurkThoracJ.2019.111 | Wrong study design |
| Dulger, S., Aykurt Karlibel, I., Kasapoglu Aksoy, M., Altan, L., Sengoren Dikis, O., & Yildiz, T. (2019). How Does Smoking Cessation Affect Disease Activity, Function Loss, and Quality of Life in Smokers With Ankylosing Spondylitis? *JCR: Journal of Clinical Rheumatology*, *25*(7), 288–296. | Did not distinguish between unsuccessful quit attempt and no attempt to quit |
| Durazzo T.C. & Meyerhoff D.J. (2020). Cigarette smoking history is associated with poorer recovery in multiple neurocognitive domains following treatment for an alcohol use disorder. Alcohol, 85, 135–143. https://doi.org/10.1016/j.alcohol.2019.12.003 | Wrong study design |
| Ebbert JO, Hatsukami DK, Croghan IT, Schroeder DR, Allen SS, Hays JT, & Hurt RD. (2014). Combination varenicline and bupropion SR for tobacco-dependence treatment in cigarette smokers: A randomized trial. JAMA, 311(2), 155–163. https://doi.org/10.1001/jama.2013.283185 | Did not analyse mental health outcomes by exposure of interest |
| Ebbert, J., Jimenez-Ruiz, C., Dutro, M. P., Fisher, M., Li, J., & Hays, J. T. (2021). Frequently Reported Adverse Events With Smoking Cessation Medications: Post Hoc Analysis of a Randomized Trial. Mayo Clinic Proceedings, 96(7), 1801–1811. https://doi.org/10.1016/j.mayocp.2020.10.046 | Mental health measured at baseline only, no follow up |
| Efraimsson, E. O. (2022). Communication in smoking cessation and self-management: A study at nurse-led COPD-clinics in primary health care. *Dissertation Abstracts International: Section B: The Sciences and Engineering*, *83*(5-B), No-Specified. | Wrong study design |
| Eiden, R. D., Homish, G. G., Colder, C. R., Schuetze, P., Gray, T. R., & Huestis, M. A. (2013). Changes in smoking patterns during pregnancy. *Substance Use & Misuse*, *48*(7), 513–522. | Wrong exposure |
| Eke, G. (2018). Evaluating the impact of a smoking cessation program. *Dissertation Abstracts International: Section B: The Sciences and Engineering*, *79*(4-B(E)), No-Pagination Specified. | Wrong outcome |
| El-Mohandes, A. A., El-Khorazaty, M. N., Kiely, M., & Gantz, M. G. (2011). Smoking cessation and relapse among pregnant African-American smokers in Washington, DC. *Maternal & Child Health Journal*, *15 Suppl 1*, S96-105. | Did not analyse mental health outcomes by exposure of interest |
| Elwany S., Shewel Y., Bazak R., Talaat I., & Elwany M. (2020). Quitting smoking reverses nasal mucosal changes. European Archives of Oto-Rhino-Laryngology, 277(6), 1691–1698. https://doi.org/10.1007/s00405-020-05896-x | Wrong outcome |
| Elzorkany, B., Mokbel, A., Gamal, S. M., Hmamouchi, I., & Dougados, M. (2021). Does smoking affect level of seropositivity in RA? A post-HOC global and inter-country analysis of COMORA cohort. Rheumatology International, 41(4), 699–705. https://doi.org/10.1007/s00296-021-04791-w | Wrong study design |
| Endrighi Romano, McQuaid Elizabeth L, Bartlett Yvonne Kiera, Clawson Ashley H, & Borrelli Belinda. (2018). Parental depression is prospectively associated with lower smoking cessation rates and poor child asthma outcomes. Annals of Behavioral Medicine, 52(3), 195–203. https://doi.org/10.1093/abm/kax011 | Mental health measured at baseline only, no follow up |
| Endrighi, R., Zhao, Y., Hughes, R. B., Kumar, D., & Borrelli, B. (2022). Associations Between Smoking Status and Physical and Mental Health-Related Quality of Life Among Individuals With Mobility Impairments. Annals of Behavioral Medicine : A Publication of the Society of Behavioral Medicine, 56(9), 890–899. https://doi.org/10.1093/abm/kaab077 | Mental health measured at baseline only, no follow up |
| Eng, L., Alton, D., Song, Y., Su, J., Zhang, Q., Che, J., Farzanfar, D., Mohan, R., Krys, O., Mattina, K., Harper, C., Liu, S., Yoannidis, T., Milne, R., Abdelmutti, N., Brown, M. C., Vennettilli, A., Hope, A. J., Howell, D., … Liu, G. (2020). Awareness of the Harms of Continued Smoking Among Cancer Survivors. Supportive Care in Cancer, 28(7), 3409–3419. https://doi.org/10.1007/s00520-019-05175-4 | Wrong outcome |
| Ercan E. & Ercan P. (2021). Retrospective analysis of smoking cessation campaign in aviation personnel. Annals of Clinical and Analytical Medicine, 12(2), 157–161. https://doi.org/10.4328/ACAM.20228 | Mental health measured at baseline only, no follow up |
| Ernst J. (2024). Quitting smoking after cancer treatment improves mental health. *Psychotherapie Psychosomatik Medizinische Psychologie*, *74*(3–4), 99. https://doi.org/10.1055/a-2230-7537 | Wrong exposure |
| Erwin M.C., Dennis P.A., Coughlin L.N., Calhoun P.S., & Beckham J.C. (2019). Examining the relationship between negative affect and posttraumatic stress disorder symptoms among smokers using ecological momentary assessment. Journal of Affective Disorders, 253, 285–291. https://doi.org/10.1016/j.jad.2019.04.035 | Wrong study design |
| Esmer, B., Sengezer, T., Aksu, F., Ozkara, A., & Aksu, K. (2019). Clinical, sociodemographic and tobacco-use factors associated with smoking cessation rates at three years follow-up, Ankara, Turkey. Tobacco Prevention & Cessation, 5(101693412), 47. https://doi.org/10.18332/tpc/114082 | Wrong study design |
| Etcheverry, P. E., Waters, A. J., Lam, C., Correa-Fernandez, V., Vidrine, J. I., Cinciripini, P. M., & Wetter, D. W. (2016). Attentional bias to negative affect moderates negative affect’s relationship with smoking abstinence. *Health Psychology*, *35*(8), 881–890. | Follow up < 6 weeks |
| Etter, J.-F., Khazaal, Y., & Abroms, A. (2022). The Stop-tabac smartphone application for smoking cessation: A randomized controlled trial. Addiction, 117(5), 1406–1415. https://doi.org/10.1111/add.15738 | Mental health measured at baseline only, no follow up |
| EUCTR2010-022914-15-SK. (2012). This study investigates the effects of various smoking cessation therapies on people with and without a history of psychiatric conditions. *Http://Www.Who.Int/Trialsearch/Trial2.Aspx?TrialID=EUCTR2010-022914-15-SK*. | Did not analyse mental health outcomes by exposure of interest |
| Evins AE, Benowitz NL, West R, Russ C, McRae T, Lawrence D, Krishen A, St Aubin L, Maravic MC, & Anthenelli RM. (2019). Neuropsychiatric Safety and Efficacy of Varenicline, Bupropion, and Nicotine Patch in Smokers With Psychotic, Anxiety, and Mood Disorders in the EAGLES Trial. Journal of Clinical Psychopharmacology, 39(2), 108–116. https://doi.org/10.1097/JCP.0000000000001015 | Did not analyse mental health outcomes by exposure of interest |
| Evins AE, Cather C, Pratt SA, Pachas GN, Hoeppner SS, Goff DC, Achtyes ED, Ayer D, & Schoenfeld DA. (2014). Maintenance treatment with varenicline for smoking cessation in patients with schizophrenia and bipolar disorder: A randomized clinical trial. JAMA, 311(2), 145–154. https://doi.org/10.1001/jama.2013.285113 | Did not analyse mental health outcomes by exposure of interest |
| Fakhoury, M. Q., Ghorayeb, A. M., Houlihan, M. D., Powers, R. J., Hurley, S., Wille, M. A., Freeman, V. L., Kim, K., Psutka, S. P., Vidal, P., & Hollowell, C. M. (2021). Predictive Risk Factors for Continued Smoking after the Diagnosis of a Genitourinary Malignancy. Urology, 147(wsy, 0366151), 178–185. https://doi.org/10.1016/j.urology.2020.05.089 | Wrong exposure |
| Falcone M., Bernardo L., Wileyto E.P., Allenby C., Burke A.M., Hamilton R., Cristancho M., Ashare R.L., Loughead J., & Lerman C. (2019). Lack of effect of transcranial direct current stimulation (tDCS) on short-term smoking cessation: Results of a randomized, sham-controlled clinical trial. Drug and Alcohol Dependence, 194, 244–251. https://doi.org/10.1016/j.drugalcdep.2018.10.016 | Wrong study design |
| Fallin-Bennett, A., Rademacher, K., Dye, H., Elswick, A., Ashford, K., & Goodin, A. (2019). Perinatal navigator approach to smoking cessation for women with prevalent opioid dependence. *Western Journal of Nursing Research*, *41*(8), 1103–1120. | Did not analyse mental health outcomes by exposure of interest |
| Fankhauser, C. D., Affentranger, A., Cortonesi, B., Jeker, U., Gass, M., Minervini, F., Jung, G., Christmann, C., Brambs, C., Puhan, M. A., & Held, U. (2022). Preoperative smoking cessation program in patients undergoing intermediate to high-risk surgery: A randomized, single-blinded, controlled, superiority trial. Trials, 23(1), 717. https://doi.org/10.1186/s13063-022-06628-8 | Did not analyse mental health outcomes by exposure of interest |
| Farris S.G., Legasse A.J., Uebelacker L.A., Brown R.A., Price L.H., & Abrantes A.M. (2019). Anxiety Sensitivity is Associated with Lower Enjoyment and an Anxiogenic Response to Physical Activity in Smokers. Cognitive Therapy and Research, 43(1), 78–87. https://doi.org/10.1007/s10608-018-9948-z | Only participants who successfully quit were included |
| Farris, S. G., Matsko, S. V., Uebelacker, L. A., Brown, R. A., Price, L. H., Abrantes, A. M., & Alvaro, B. (2020). Anxiety sensitivity and daily cigarette smoking in relation to sleep disturbances in treatment-seeking smokers. Cognitive Behaviour Therapy, 49(2), 137–148. https://doi.org/10.1080/16506073.2019.1583277 | Wrong exposure |
| Farris, S. G., Zvolensky, M. J., & Schmidt, N. B. (2015). Smoking-specific experiential avoidance cognition: Explanatory relevance to pre- and post-cessation nicotine withdrawal, craving, and negative affect. Addictive Behaviors, 44, 58–64. https://doi.org/10.1016/j.addbeh.2014.07.026 | Follow up < 6 weeks |
| Fatemi, S., Yousefi, M. K., Kneeland, R. E., Liesch, S. B., Folsom, T. D., & Thuras, P. D. (2013). Antismoking and potential antipsychotic effects of varenicline in subjects with schizophrenia or schizoaffective disorder: A double-blind placebo and bupropion-controlled study. Schizophrenia Research, 146(1–3), 376–378. https://doi.org/10.1016/j.schres.2013.02.015 | Did not analyse mental health outcomes by exposure of interest |
| Feldman, I., Helgason, A. R., Johansson, P., Tegelberg, A., & Nohlert, E. (2019). Cost-effectiveness of a high-intensity versus a low-intensity smoking cessation intervention in a dental setting: Long-term follow-up. BMJ Open, 9(8), e030934. https://doi.org/10.1136/bmjopen-2019-030934 | Wrong study design |
| Felicitas-Perkins, J. Q. (2019). An exploration of the potential consequences of tobacco harm reduction: Examining the associations between switching behavior, tobacco use status, nicotine dependence, and cessation outcomes. *Dissertation Abstracts International: Section B: The Sciences and Engineering*, *80*(5-B(E)), No-Specified. | Wrong study design |
| Fernandez Arias, I. G., Garcia-Vera, M. P., & Sanz, J. (2014). The more psychology, the better: The efficacy of smoking cessation treatment using intensive cognitive-behavioral therapy versus a combination of nicotine patches plus intensive or less intensive cognitive-behavioral therapy: First prize of the 20th "Rafa. *Clinica y Salud*, *25*(1), 1–10. | Did not analyse mental health outcomes by exposure of interest |
| Ferra J.R.M., Vieira A.C., Oliveira I., Carvalho J., Matos C., & Nogueira F. (2020). Smoking cessation according to nicotine dependence: Experience of a smoking cessation program. European Respiratory Journal, 56(Supplement 64). https://doi.org/10.1183/13993003.congress-2020.3052 | Wrong study design |
| Ferron, J. C., Devitt, T., McHugo, G. J., Jonikas, J., Cook, J. A., & Brunette, M. F. (2016). Abstinence and use of community-based cessation treatment after a motivational intervention among smokers with severe mental illness. Community Mental Health Journal, 52(4), 446–456. https://doi.org/10.1007/s10597-016-9998-1 | Wrong outcome |
| Fibbins, H., Ward, P. B., Morell, R., Lederman, O., Teasdale, S., Davies, K., McGuigan, B., & Curtis, J. (2024). Evaluation of a smoking cessation program for adults with severe mental illness in a public mental health service. *Journal of Psychiatric and Mental Health Nursing*, *22(7), 547-562.* https://doi.org/10.1111/jpm.13052 | Mental health measured at baseline only, no follow up |
| Fidanci I., Aksoy H., Yengil Taci D., Ayhan Baser D., & Cankurtaran M. (2021). Evaluation of the effect of the Covid-19 pandemic on smoking addiction levels. International Journal of Clinical Practice, 75(5), e14012. https://doi.org/10.1111/ijcp.14012 | Wrong study design |
| Fillo, J., Kamper-DeMarco, K. E., Brown, W. C., Stasiewicz, P. R., Bradizza, C. M., & Allen, A. (2019). Emotion regulation difficulties and social control correlates of smoking among pregnant women trying to quit. Addictive Behaviors, 89, 104–112. https://doi.org/10.1016/j.addbeh.2018.09.033 | Wrong study design |
| Firat, M., Demir Gokmen, B., & Karakurt, P. (2022). An investigation of smoking habits and mental well-being in healthcare personnel during COVID-19. Perspectives in Psychiatric Care, 58(1), 108–113. https://doi.org/10.1111/ppc.12819 | Wrong study design |
| Foa Edna B, Asnaani Anu, Rosenfield David, Zandberg Laurie J, Gariti Peter, & Imms Patricia. (2017). Concurrent varenicline and prolonged exposure for patients with nicotine dependence and PTSD: a randomized controlled trial. Journal of Consulting and Clinical Psychology, 85(9), 862–872. https://doi.org/10.1037/ccp0000213 | Did not analyse mental health outcomes by exposure of interest |
| Fobian, A. D., Schiavon, S., Elliott, L., Stager, L., & Cropsey, K. L. (2020). Body Mass Index Changes Associated with Smoking are Moderated by Race and Depression. Journal of Health Care for the Poor and Underserved, 31(3), 1115–1123. https://doi.org/10.1353/hpu.2020.0084 | Wrong study design |
| Forrest PE, Brinson AJ, Gannon JM, George TP, Perkins KA, & Chengappa KN. (2015). An association between the use of hypnotics and quit status in the treatment of nicotine dependence with varenicline in bipolar disorder. Journal of Clinical Psychopharmacology, 35(2), 199–200. https://doi.org/10.1097/JCP.0000000000000272 | Wrong outcome |
| Foulds J, Russ C, Yu CR, Zou KH, Galaznik A, Franzon M, Berg A, & Hughes JR. (2013). Effect of varenicline on individual nicotine withdrawal symptoms: A combined analysis of eight randomized, placebo-controlled trials. Nicotine & Tobacco Research, 15(11), 1849–1857. https://doi.org/10.1093/ntr/ntt066 | Not primary research |
| Foulds J., Veldheer S., Pachas G., Hrabovsky S., Hameed A., Allen S.I., Cather C., Azzouz N., Yingst J., Hammett E., Modesto J., Krebs N.M., Lester C., Trushin N., Reinhart L., Wasserman E., Zhu J., Liao J., Muscat J.E., … Evins A.E. (2022). The effects of reduced nicotine content cigarettes in smokers with mood or anxiety disorders: A double-blind randomized trial. medRxiv. https://doi.org/10.1101/2022.05.24.22275536 | Wrong study design |
| Foulds, J., Veldheer, S., Pachas, G., Hrabovsky, S., Hameed, A., Allen, S. I., Cather, C., Azzouz, N., Yingst, J., Hammett, E., Modesto, J., Krebs, N. M., Lester, C., Trushin, N., Reinhart, L., Wasserman, E., Zhu, J., Liao, J., Muscat, J. E., … Evins, A. E. (2022). The effects of reduced nicotine content cigarettes on biomarkers of nicotine and toxicant exposure, smoking behavior and psychiatric symptoms in smokers with mood or anxiety disorders: A double-blind randomized trial. PloS One, 17(11), e0275522. https://doi.org/10.1371/journal.pone.0275522 | Mental health measured at baseline only, no follow up |
| Frank, D. W., Cinciripini, P. M., Deweese, M. M., Karam-Hage, M., Kypriotakis, G., Lerman, C., Robinson, J. D., Tyndale, R. F., Vidrine, D. J., Versace, F., & Babb, B. (2020). Toward precision medicine for smoking cessation: Developing a neuroimaging-based classification algorithm to identify smokers at higher risk for relapse. Nicotine & Tobacco Research, 22(8), 1277–1284. https://doi.org/10.1093/ntr/ntz211 | Wrong study design |
| Fredman Stein, K., Sawyer, K., Daryan, S., Allen, J., & Taylor, G. (2023). Service-user experiences of an integrated psychological intervention for depression or anxiety and tobacco smoking in improving access to psychological therapies services: A qualitative investigation into mechanisms of change in quitting smoking. *Health Expectations, 26*(1), 498–509. https://doi.org/10.1111/hex.13684 | Wrong study design |
| Frischknecht, U., Patz, T., Reinhard, I., Dinter, C., Kiefer, F., & Weber, T. (2021). Predicting Participation in and Success of a Concurrent Smoking Cessation Program during Inpatient Treatment for Alcohol Dependence. *Psychiatria Danubina*, *33*(Suppl 10), 76–88. | Wrong study design |
| Fujita, T., Babazono, A., Harano, Y., & Jiang, P. (2019). Risk of depressive disorders after tobacco smoking cessation: A retrospective cohort study in Fukuoka, Japan. BMJ Open, 9(3). https://doi.org/10.1136/bmjopen-2018-025124 | Wrong outcome |
| Gajos, J. M., Hawes, E. S., Chana, S. M., Mrug, S., Wolford-Clevenger, C., Businelle, M. S., Carpenter, M. J., & Cropsey, K. L. (2023). Daily adherence to nicotine replacement therapy in low-income smokers: The role of gender, negative mood, motivation, and self-efficacy. *Addictive Behaviors, 138*, 107543. https://doi.org/10.1016/j.addbeh.2022.107543 | Wrong outcome |
| Galiatsatos, P., Oluyinka, M., Min, J., Schreiber, R., Lansey, D. G., Ikpe, R., Pacheco, M. C., DeJaco, V., Ellison-Barnes, A., Neptune, E., Kanarek, N. F., & Cudjoe, T. K. M. (2022). Prevalence of Mental Health and Social Connection among Patients Seeking Tobacco Dependence Management: A Pilot Study. International Journal of Environmental Research and Public Health, 19(18). https://doi.org/10.3390/ijerph191811755 | Wrong study design |
| Galil A.G.D.S., Andrade B.A.B.B., Lamas M.F.M., Bastos M.G., & Banhato E.F.C. (2020). Abdominal obesity in smokers: Is this association real? Atherosclerosis, 315, e169. https://doi.org/10.1016/j.atherosclerosis.2020.10.521 | Wrong study design |
| Galil A.G.D.S., Banhato E.F.C., Andrade B.A.B.B., Lamas M.F.M., & Bastos M.G. (2020). Cognition and biopsychological profile among elderly smokers undergoing smoking cessation. Atherosclerosis, 315, e231. https://doi.org/10.1016/j.atherosclerosis.2020.10.728 | Wrong outcome |
| Garcia-Perez, A., Vallejo-Seco, G., Weidberg, S., Gonzalez-Roz, A., Secades-Villa, R., & Aguirre, A. (2020). Long-term changes in delay discounting following a smoking cessation treatment for patients with depression. Drug and Alcohol Dependence, 212. https://doi.org/10.1016/j.drugalcdep.2020.108007 | Wrong study design |
| Garcia-Portilla, M. P., Garcia-Alvarez, L., Saiz, P. A., Diaz-Mesa, E., Galvan, G., Sarramea, F., Garcia-Blanco, J., Elizagarate, E., & Bobes, J. (2014). Effectiveness of a multi-component smoking cessation support programme (McSCSP) for patients with severe mental disorders: Study design. International Journal of Environmental Research and Public Health, 11(1), 373–389. https://doi.org/10.3390/ijerph110100373 | Did not analyse mental health outcomes by exposure of interest |
| Garey L., Hebert E.T., Mayorga N.A., Chavez J., Shepherd J.M., Businelle M.S., & Zvolensky M.J. (2022). Evaluating the feasibility and acceptability of a mobile-based health technology for smoking cessation: Mobile Anxiety Sensitivity Program. The British Journal of Clinical Psychology, 61(Supplement 1), 111–129. https://doi.org/10.1111/bjc.12294 | Wrong study design |
| Garey, L., Kauffman, B. Y., Manning, K. F., Taha, S. A., Schmidt, N. B., Neighbors, C., & Zvolensky, M. J. (2019). The Effect of Positive and Negative Affect on Early Treatment Milestones in the Context of Integrated Smoking Treatment. Journal of Addiction Medicine, 13(1), 47–54. https://doi.org/10.1097/ADM.0000000000000445 | Wrong study design |
| Garey, L., Robison, J. H., Matoska, C. T., Montgomery, A., Jones, A., Hebert, E. T., Vujanovic, A. A., Kezbers, K. M., Cheney, M. K., Gallagher, M. W., Obasi, E. M., Zvolensky, M. J., & Businelle, M. S. (2024). A proof-of-concept trial of a smoking cessation and anxiety sensitivity reduction smartphone application for black adults. *Cognitive Behaviour Therapy*, 1-26. https://doi.org/10.1080/16506073.2024.2431555 | Did not analyse mental health outcomes by exposure of interest |
| Garey, L., Rogers, A. H., Manning, K., Smit, T., Derrick, J. L., Viana, A. G., Schmidt, N. B., Zvolensky, M. J., & Akaike, A. (2020). Effects of smoking cessation treatment attendance on abstinence: The moderating role of psychologically based behavioral health conditions. *Journal of Substance Abuse Treatment, 109*. https://doi.org/10.1016/j.jsat.2019.10.006 | Wrong outcome |
| Garey, L., Senger, A. R., Smit, T., Nizio, P., Matoska, C. T., Kauffman, B., Businelle, M. S., Gallagher, M. W., & Zvolensky, M. J. (2023). Anxiety sensitivity and reasons for smoking among Black smokers. *Addictive Behaviors, 139*, 107593. https://doi.org/10.1016/j.addbeh.2022.107593 | Wrong study design |
| Garey, L., Wirtz, M. R., Labbe, A. K., Zvolensky, M. J., Smits, J. A. J., Giordano, T. P., Rosenfield, D., Robbins, G. K., Levy, D. E., McKetchnie, S. M., Bell, T., & O’Cleirigh, C. (2021). Evaluation of an integrated treatment to address smoking cessation and anxiety/depressive symptoms among people living with HIV: Study protocol for a randomized controlled trial. Contemporary Clinical Trials, 106(101242342), 106420. https://doi.org/10.1016/j.cct.2021.106420 | Wrong study design |
| Garrison, K. A., Pal, P., O’Malley, S. S., Pittman, B. P., Gueorguieva, R., Rojiani, R., Scheinost, D., Dallery, J., & Brewer, J. A. (2020). Craving to Quit: A Randomized Controlled Trial of Smartphone App–Based Mindfulness Training for Smoking Cessation. Nicotine & Tobacco Research, 22(3), 324–331. https://doi.org/10.1093/ntr/nty126 | Wrong study design |
| Garza, D., Murphy, M., Tseng, L. J., Riordan, H. J., & Chatterjee, A. (2011). A double-blind randomized placebo-controlled pilot study of neuropsychiatric adverse events in abstinent smokers treated with varenicline or placebo. *Biological Psychiatry*, *69*(11), 1075–1082. | Did not analyse mental health outcomes by exposure of interest |
| Gates J., Kaklamanou D., Rupani H., Brown T.P., Pilkington K., Longstaff J., & Chauhan A.J. (2021). Stop (the staff smoking project): Designing a sustainable smoking cessation programme for nhs staff. Thorax, 76(SUPPL 1), A64. https://doi.org/10.1136/thorax-2020-BTSabstracts.110 | Wrong study design |
| Gathright, E. C., Vickery, K. D., Ayenew, W., Whited, M. C., Adkins-Hempel, M., Chrastek, M., Carter, J. K., Rosen, R. K., Wu, W.-C., Busch, A. M., & Ali, A. (2022). The development and pilot testing of a behavioral activation-based treatment for depressed mood and multiple health behavior change in patients with recent acute coronary syndrome. PLoS ONE, 17(2). https://doi.org/10.1371/journal.pone.0261490 | Wrong study design |
| Gelkopf M, Noam S, Rudinski D, Lerner A, Behrbalk P, Bleich A, & Melamed Y. (2012). Nonmedication smoking reduction program for inpatients with chronic schizophrenia: A randomized control design study. Journal of Nervous and Mental Disease, 200(2), 142–146. https://doi.org/10.1097/NMD.0b013e3182438e92 | Wrong exposure |
| Gemine R., Ghosal R., Collier G., & Lewis K. (2019). Effect of quitting smoking on quality of life following a diagnosis of lung cancer. European Respiratory Journal, 54(Supplement 63). https://doi.org/10.1183/13993003.congress-2019.OA5133 | Wrong exposure |
| Germeroth L.J., Wang Z., Emery R.L., Cheng Y., & Levine M.D. (2019). The Role of Self-Efficacy and Motivation in Postpartum Sustained Smoking Abstinence. Women’s Health Issues, 29(3), 259–266. https://doi.org/10.1016/j.whi.2019.03.006 | Mental health not measured before quit attempt |
| Ghatak, A., Gilman, S., Carney, S., Gonzalez, A. V., Benedetti, A., & Ezer, N. (2022). Smoking Cessation by Phone Counselling in a Lung Cancer Screening Program: A Retrospective Comparative Cohort Study. Canadian Respiratory Journal, 2022(c1w, 9433332), 5446751. https://doi.org/10.1155/2022/5446751 | Wrong study design |
| Gibbons RD & Mann JJ. (2013). Varenicline, smoking cessation, and neuropsychiatric adverse events. American Journal of Psychiatry, 170(12), 1460–1467. https://doi.org/10.1176/appi.ajp.2013.12121599 | Not primary research |
| Gibson, I., Flaherty, G., Cormican, S., Jones, J., Kerins, C., Walsh, A. M., Costello, C., Windle, J., Connolly, S., & Crowley, J. (2014). Translating guidelines to practice: Findings from a multidisciplinary preventive cardiology programme in the west of Ireland. European Journal of Preventive Cardiology, 21(3), 366–376. https://doi.org/10.1177/2047487313498831 | Did not analyse mental health outcomes by exposure of interest |
| Gilbert DG, Rabinovich NE, Gilbert-Matuskowitz EA, Klein KP, & Pergadia ML. (2019). Smoking abstinence symptoms across 67 days compared with randomized controls-moderation by nicotine replacement therapy, bupropion, and negative-affect traits. *Experimental and Clinical Psychopharmacology*. | Did not analyse mental health outcomes by exposure of interest |
| Gilbody, S., Coleman, I., Peckham, E., Bailey, D., Arundel, C., Heron ,P., Crosland, S., Fairhurst, C., Li, J., Hewitt, C., Parrott, S., Bradshaw, T., Horspool, M., Hughes, L., Hughes, T., Ker, S., Leahy, M., McCloud, T., Osborn, D., … Vickers, C. (2024). Long term smoking and quitting among people with severe mental illness: 3-year follow-up of the SCIMITAR+ Trial. *medRxiv.* https://doi.org/10.1101/2024.06.03.24308386 | Did not analyse mental health outcomes by exposure of interest |
| Gilbody S., Peckham E., Bailey D., Arundel C., Heron P., Crosland S., Fairhurst C., Hewitt C., & Li J. (2021). Smoking cessation in severe mental illness: Combined long-term quit rates from the UK SCIMITAR trials programme. British Journal of Psychiatry, 218(2), 95–97. https://doi.org/10.1192/bjp.2019.192 | Wrong outcome |
| Gilbody, S., Peckham, E., Bailey, D., Arundel, C., Heron, P., Crosland, S., Fairhurst, C., Hewitt, C., Li, J., Parrott, S., Bradshaw, T., Horspool, M., Hughes, E., Hughes, T., Ker, S., Leahy, M., McCloud, T., Osborn, D., Reilly, J., … Vickers, C. (2019). Smoking cessation for people with severe mental illness (SCIMITAR+): A pragmatic randomised controlled trial. The Lancet Psychiatry, 6(5), 379–390. https://doi.org/10.1016/S2215-0366%2819%2930047-1 | Did not analyse mental health outcomes by exposure of interest |
| Ginsberg, D., Hall, S. M., Reus, V. I., & Muñoz, R. F. (1995). Mood and depression diagnosis in smoking cessation. Experimental and Clinical Psychopharmacology, 3(4), 389–395. https://doi.org/10.1037/1064-1297.3.4.389 | Did not analyse mental health outcomes by exposure of interest |
| Giovancarli C, Malbos E, Baumstarck K, Parola N, Pelissier M-F, Lancon C, Auquier P, & Boyer L. (2016). Virtual reality cue exposure for the relapse prevention of tobacco consumption: A study protocol for a randomized controlled trial. Trials, 17(1) (no pagination), 96. https://doi.org/10.1186/s13063-016-1224-5 | Did not analyse mental health outcomes by exposure of interest |
| Gjorgjievski D., Ristovska R., Stavrikj K., Farley A., Adab P., Adams R., Dickens A., Enocson A., Stanoevski G., Gale N., Jowett S., Rai K., Sitch A., Stamenova A., Krstevska E., & Jordan R. (2020). Effectiveness of combining feedback about lung age or exhaled carbon monoxide levels with very brief advice (VBA) and support for smoking cessation in primary care compared to giving vba and support alone—Protocol for a randomized controlled trial within the breathe well research program. Open Access Macedonian Journal of Medical Sciences, 8(E), 28–36. https://doi.org/10.3889/oamjms.2020.4519 | Wrong outcome |
| Glassman, A. H., Covey, L. S., Stetner, F., & Rivelli, S. (2001). Smoking cessation and the course of major depression: A follow-up study. Lancet (London, England), 357(9272), 1929–1932. https://doi.org/10.1016/S0140-6736(00)05064-9 | Wrong outcome |
| Gobarani, R. K., Ilomaki, J., Wood, S., Abramson, M. J., Bonevski, B., George, J., & Anthenelli, B. (2022). Characterising the use of varenicline: An analysis of the Australian dispensing claims data. Addiction, 117(10), 2683–2694. https://doi.org/10.1111/add.15949 | Wrong study design |
| Goettler D., Wagner M., Faller H., Kotseva K., Wood D., Leyh R., Ertl G., Karmann W., Heuschmann P.U., Stork S., Nolte K., Schich M., Wahl V., Breunig M., Eichstadt K., Gerhardt A., Ludwig T., Memmel Y., Quilitzsch A., … Tiffe T. (2020). Factors associated with smoking cessation in patients with coronary heart disease: A cohort analysis of the German subset of EuroAspire IV survey. BMC Cardiovascular Disorders, 20(1), 152. https://doi.org/10.1186/s12872-020-01429-w | Wrong exposure |
| Golden, S. E. (2021). Smoking behaviors in patients offered lung cancer screening. *Dissertation Abstracts International: Section B: The Sciences and Engineering*, *82*(7-B), No-Specified. | Wrong study design |
| Gomajee R., El-Khoury F., Goldberg M., Zins M., Lemogne C., Wiernik E., Lequy-Flahault E., Romanello L., Kousignian I., & Melchior M. (2019). Association between Electronic Cigarette Use and Smoking Reduction in France. JAMA Internal Medicine, 179(9), 1193–1200. https://doi.org/10.1001/jamainternmed.2019.1483 | Wrong exposure |
| Gomez Moreno C., Carrion Exposito L., Del Pozo Sesena G.I., Chauca Chauca G.M., Alcala Partera J.A., Sanchez M.D., Jaen-Moreno M.J., & Sarramea Crespo F. (2020). Readiness to change versus stage of change to predict the efficacy of tobacco reduction in severe mental illness. European Psychiatry, 63(Supplement 1), S533. https://doi.org/10.1192/j.eurpsy.2020.6 | Wrong study design |
| Gonzaga-Carvalho C., Kochen D., & Abramson B. (2019). Effectiveness and predictors of a smoking cessation program in patients with cardiovascular disease: sex and affordability matter. Canadian Journal of Cardiology, 35(10 Supplement), S186. https://doi.org/10.1016/j.cjca.2019.07.601 | Wrong exposure |
| Gonzalez-Roz, A., Secades-Villa, R., & Alonso-Perez, F. (2019). Effects of combining contingency management with behavioral activation for smokers with depression. Addiction Research & Theory, 27(2), 114–121. https://doi.org/10.1080/16066359.2018.1463371 | Did not analyse mental health outcomes by exposure of interest |
| Gonzalez-Roz, A., Secades-Villa, R., & Alpi, A. (2022). Contingency management for smokers with mental health disorders and smoking-sensitive conditions caused or exacerbated by tobacco use: A review of existing studies, intervention parameters, and research priorities. Experimental and Clinical Psychopharmacology. https://doi.org/10.1037/pha0000585 | Wrong study design |
| Gonzalez-Roz, A., Secades-Villa, R., Garcia-Fernandez, G., Martinez-Loredo, V., Alonso-Perez, F., & Baker, B. (2021). Depression symptom profiles and long-term response to cognitive behavioral therapy plus contingency management for smoking cessation. Drug and Alcohol Dependence, 225. https://doi.org/10.1016/j.drugalcdep.2021.108808 | Wrong study design |
| Gonzalez-Roz, A., Secades-Villa, R., Pericot-Valverde, I., Weidberg, S., Alonso-Perez, F., & Aguirre, A. F. (2019). Effects of delay discounting and other predictors on smoking relapse. The Spanish Journal of Psychology, 22. https://doi.org/10.1017/sjp.2019.11 | Wrong study design |
| Gonzalez-Roz, A., Secades-Villa, R., Weidberg, S., Garcia-Perez, A., Reed, D. D., & Amlung, A.-M. (2020). Latent structure of the cigarette purchase task among treatment-seeking smokers with depression and its predictive validity on smoking abstinence. Nicotine & Tobacco Research, 22(1), 74–80. https://doi.org/10.1093/ntr/nty236 | Mental health measured at baseline only, no follow up |
| Gonzalez-Roz, A., Weidberg, S., Garcia-Perez, A., Martinez-Loredo, V., Secades-Villa, R., Addictive Behaviors Research Group (GCA), & Audrain-McGovern, B. (2021). One-year efficacy and incremental cost-effectiveness of contingency management for cigarette smokers with depression. Nicotine & Tobacco Research, 23(2), 320–326. https://doi.org/10.1093/ntr/ntaa146 | Wrong study design |
| Gorniak, B., Yong, H.-H., Borland, R., Cummings, K. M., Thrasher, J. F., McNeill, A., Hyland, A., & Fong, G. T. (2022). Do post-quitting experiences predict smoking relapse among former smokers in Australia and the United Kingdom? Findings from the International Tobacco Control Surveys. Drug and Alcohol Review, 41(4), 883–889. https://doi.org/10.1111/dar.13419 | Wrong outcome |
| Graham, A. L., Papandonatos, G. D., Cha, S., Amato, M. S., Jacobs, M. A., Cohn, A. M., Abroms, L. C., Whittaker, R., & Alkhaldi, A. (2022). Effectiveness of an optimized text message and internet intervention for smoking cessation: A randomized controlled trial. Addiction, 117(4), 1035–1046. https://doi.org/10.1111/add.15677 | Wrong study design |
| Grassi, M. C., Enea, D., Ferketich, A. K., Lu, B., Pasquariello, S., & Nencini, P. (2011). Effectiveness of varenicline for smoking cessation: A 1-year follow-up study. J Subst Abuse Treat, 41(1), 64–70. https://doi.org/10.1016/j.jsat.2011.01.014 | Mental health measured at baseline only, no follow up |
| Greaves, L., Poole, N., Hemsing, N., & Amstadter, B. (2019). Tailored intervention for smoking reduction and cessation for young and socially disadvantaged women during pregnancy. Journal of Obstetric, Gynecologic, & Neonatal Nursing, 48(1), 90–98. https://doi.org/10.1016/j.jogn.2018.10.007 | Not primary research |
| Gu, D., Rafferty, H., & Vijayaraghavan, M. (2024). Factors Associated with Smoking Cessation and the Receipt of Cessation Services in a Public, Safety-Net Primary Care System. *Journal of General Internal Medicine*, *39*(9), 1657-1665. https://doi.org/10.1007/s11606-024-08664-3 | Wrong study design |
| Guimaraes-Pereira, B. B. S., da Silva, A. S. M., Lima, D. R., Carvalho, C. F. C., Loreto, A. R., Galvao, L. P., Frallonardo, F. P., Ismael, F., Torales, J., Ventriglio, A., de Andrade, A. G., & Castaldelli-Maia, J. M. (2021). Sex differences in smoking cessation: A Retrospective Cohort Study in a Psychosocial Care Unit in Brazil. Trends in Psychiatry and Psychotherapy, 101610695. https://doi.org/10.47626/2237-6089-2021-0217 | Wrong outcome |
| Guimond, A. J., Croteau, V. A., Savard, M. H., Bernard, P., Ivers, H., & Savard, J. (2017). Predictors of Smoking Cessation and Relapse in Cancer Patients and Effect on Psychological Variables: An 18-Month Observational Study. *Annals of Behavioral Medicine*, *51*(1), 117–127. | Did not analyse mental health outcomes by exposure of interest |
| Gunge, D., Marganski, J., Advani, I., Boddu, S., Chen, Y. J. E., Mehta, S., Merz, W., Fuentes, A. L., Malhotra, A., Banks, S. J., & Crotty Alexander, L. E. (2021). Deleterious Association of Inhalant Use on Sleep Quality during the COVID-19 Pandemic. International Journal of Environmental Research and Public Health, 18(24). https://doi.org/10.3390/ijerph182413203 | Wrong outcome |
| Gungen, A. C., Tekesin, A., Koc, A. S., Gungen, B. D., Tunc, A., Yildirim, A., Ceyran, O., & Memis, I. (2022). The effects of cognitive and emotional status on smoking cessation. European Review for Medical and Pharmacological Sciences, 26(14), 5092–5097. https://doi.org/10.26355/eurrev_202207_29295 | Mental health measured at baseline only, no follow up |
| Gyeong Son, H., & Akaike, B. (2021). Effects of smoking behaviour changes on depression in older people: A retrospective study. Australasian Journal on Ageing, 40(1), e37–e43. https://doi.org/10.1111/ajag.12842 | Did not distinguish between unsuccessful quit attempt and no attempt to quit |
| Habibagahi, R., Navabi, N., Alsadat Hashemipour, M., & Hashemzehi, A. (2020). Does Smoking Cessation Improve Oral Health-related Quality of Life? A Pilot Study. Addiction & Health, 12(3), 167–174. https://doi.org/10.22122/ahj.v12i3.273 | Wrong study design |
| Hacker, K. A., Kang, J. Y., & Ahluwalia, A., Baker, Creamer, Fiore, Glover-Kudon, Keller, Morris, Prutzman, Vickerman, Xu, Zhang. (2021). Tobacco cessation quitlines: An evolving mainstay for an enduring cessation support infrastructure. Special Issue, 60(3, Suppl 2), S185–S187. https://doi.org/10.1016/j.amepre.2020.11.001 | Not primary research |
| Hage M.K., Seoudy K., Kypriotakis G., Beneventi D., Minnix J., Robinson J., Blalock J., Cui Y., & Cinciripini P. (2020). The effect of baseline insomnia and its treatment on the ability to quit smoking. American Journal on Addictions, 29(3), 222. https://doi.org/10.1111/ajad.13032 | Wrong study design |
| Hagens P, Pieterse M, van der Valk P, & van der Palen J. (2017). Effectiveness of intensive smoking reduction counselling plus combination nicotine replacement therapy in promoting long-term abstinence in patients with chronic obstructive pulmonary disease not ready to quit smoking: Protocol of the REDUQ trial. Contemporary Clinical Trials Communications, 8, 248–257. https://doi.org/10.1016/j.conctc.2017.08.014 | Did not analyse mental health outcomes by exposure of interest |
| Hahad, O., Beutel, M., Gilan, D. A., Michal, M., Schulz, A., Pfeiffer, N., Konig, J., Lackner, K., Wild, P., Daiber, A., & Munzel, T. (2022). The association of smoking and smoking cessation with prevalent and incident symptoms of depression, anxiety, and sleep disturbance in the general population. Journal of Affective Disorders, 313(h3v, 7906073), 100–109. https://doi.org/10.1016/j.jad.2022.06.083 | Wrong outcome |
| Haile, S., Linne, A., Johansson, U.-B., & Joelsson-Alm, E. (2020). Follow-up after surgical treatment for intermittent claudication (FASTIC): A study protocol for a multicentre randomised controlled clinical trial. BMC Nursing, 19(101088683), 45. https://doi.org/10.1186/s12912-020-00437-7 | Wrong study design |
| Hale, J. W., Pacheco, J. A., Lewis, C. S., Swimmer, L., Daley, S. M., Nazir, N., Daley, C. M., Choi, W. S., & Adler, B. H. (2021). Everyday discrimination for american indian tribal college students enrolled in the internet all nations breath of life program. Journal of American College Health. https://doi.org/10.1080/07448481.2021.1987246 | Wrong study design |
| Hall, S. M., Bachman, J., Henderson, J. B., Barstow, R., & Jones, R. T. (1983). Smoking cessation in patients with cardiopulmonary disease: An initial study. Addictive Behaviors, 8(1), 33–42. https://doi.org/10.1016/0306-4603(83)90053-9 | Did not analyse mental health outcomes by exposure of interest |
| Hallford, G., Bakhshaie, J., Rodriguez-Cano, R., Shepherd, J. M., Schmidt, N. B., Zvolensky, M. J., & Allan, B. (2020). A multiple mediation model of trait worry and hazardous drinking among treatment-seeking smokers: The roles of coping and conformity drinking motives. Cognitive Behaviour Therapy, 49(5), 398–411. https://doi.org/10.1080/16506073.2020.1740315 | Wrong exposure |
| Hamieh, N., Airagnes, G., Descatha, A., Goldberg, M., Limosin, F., Roquelaure, Y., Lemogne, C., Zins, M., & Matta, J. (2022). Atypical working hours are associated with tobacco, cannabis and alcohol use: Longitudinal analyses from the CONSTANCES cohort. BMC Public Health, 22(1), 1834. https://doi.org/10.1186/s12889-022-14246-x | Wrong outcome |
| Hammett PJ, Lando HA, Taylor BC, Widome R, Erickson DJ, Joseph AM, Clothier B, & Fu SS. (2019). The relationship between smoking cessation and binge drinking, depression, and anxiety symptoms among smokers with serious mental illness. Drug and Alcohol Dependence, 194, 128–135. https://doi.org/10.1016/j.drugalcdep.2018.08.043 | Did not distinguish between unsuccessful quit attempt and no attempt to quit |
| Hammett, P. J., Businelle, M. S., Taylor, B. C., Erbes, C. R., Bastian, L., Doran, N., Sherman, S. E., Rogers, E. S., Burgess, D. J., Fu, S. S., & Al-Delaimy, B. (2021). The association between smoking abstinence and pain trajectory among veterans engaged in U.S. Department of Veterans Affairs mental health care. Pain Medicine, 22(8), 1793–1803. https://doi.org/10.1093/pm/pnab009 | Wrong study design |
| Han D.-H., Lee S.H., & Seo D.-C. (2022). Within-Person Longitudinal Associations between Electronic Nicotine Delivery Systems Use and Smoking Cessation Efforts among US Continuing Adult Cigarette Smokers. Nicotine and Tobacco Research, 24(4), 590–597. https://doi.org/10.1093/ntr/ntab232 | Wrong outcome |
| Han S, Kheder J, Bocelli L, Fahed J, Wachholtz A, Seward G, & Wassef W. (2016). Smoking cessation in a chronic pancreatitis population. Pancreas, 45(9), 1303–1308. https://doi.org/10.1097/MPA.0000000000000641 | Did not analyse mental health outcomes by exposure of interest |
| Han, J.-W., & Lee, H. (2020). The effects of hardcore smokers’ depression and self-esteem on daily smoking amount. Archives of Psychiatric Nursing, 34(3), 149–158. https://doi.org/10.1016/j.apnu.2020.02.006 | Wrong study design |
| Hansoti E., Pollio D.E., & North C.S. (2022). A prospective longitudinal study of tobacco use and tobacco use disorder in a homeless population. Annals of Clinical Psychiatry, 34(3), 167–175. https://doi.org/10.12788/acp.0061 | Wrong outcome |
| Harris, T., Winetrobe, H., Rhoades, H., Wenzel, S., & Al-Tayyib, A. (2019). The role of mental health and substance use in homeless adults’ tobacco use and cessation attempts. Journal of Dual Diagnosis, 15(2), 76–87. https://doi.org/10.1080/15504263.2019.1579947 | Wrong study design |
| Harrison, K., Nicole, N., Friedrichsen Samantha, Tosun Nicole, Oyenuga Abayomi, & Allen Sharon. (2019). Self-reported leisure time exercise change during smoking cessation in men and women. Addictive Behaviors, 99(no pagination), 106025. https://doi.org/10.1016/j.addbeh.2019.06.014 | Did not analyse mental health outcomes by exposure of interest |
| Hart A., Rainer W.G., Taunton M.J., Mabry T.M., Berry D.J., & Abdel M.P. (2019). Smoking Cessation Before and After Total Joint Arthroplasty-An Uphill Battle. Journal of Arthroplasty, 34(7 Supplement), S140–S143. https://doi.org/10.1016/j.arth.2019.01.073 | Wrong study design |
| Hartog, J., Blokzijl, F., Dijkstra, S., DeJongste, M. J. L., Reneman, M. F., Dieperink, W., van der Horst, I. C. C., Fleer, J., van der Woude, L. H. V., van der Harst, P., & Mariani, M. A. (2019). Heart Rehabilitation in patients awaiting Open heart surgery targeting to prevent Complications and to improve Quality of life (Heart-ROCQ): Study protocol for a prospective, randomised, open, blinded endpoint (PROBE) trial. BMJ Open, 9(9), e031738. https://doi.org/10.1136/bmjopen-2019-031738 | Did not analyse mental health outcomes by exposure of interest |
| Hashimoto, R., Tomioka, H., Wada, T., & Yoshizumi, Y. (2020). Outcomes and predictive factors for successful smoking cessation therapy in COPD patients with nicotine dependence. Respiratory Investigation, 58(5), 387–394. https://doi.org/10.1016/j.resinv.2020.03.007 | Wrong study design |
| Hata, A., Komiyama, M., Yasoda, A., Wada, H., Yamakage, H., Satoh-Asahara, N., Morimoto, T., Takahashi, Y., & Hasegawa, K. (2022). Psychological Effects of Aromatherapy on Smokers With Depressive Tendencies During Smoking Cessation Treatment: Protocol for a Pre-Post Single-Arm Clinical Trial. JMIR Research Protocols, 11(7), e38626. https://doi.org/10.2196/38626 | Did not analyse mental health outcomes by exposure of interest |
| Hatsukami, D. K., Meier, E., Lindgren, B. R., Anderson, A., Reisinger, S. A., Norton, K. J., Strayer, L., Jensen, J. A., Dick, L., Murphy, S. E., Carmella, S. G., Tang, M.-K., Chen, M., Hecht, S. S., O’connor, R. J., Shields, P. G., & Brown, B. (2020). A randomized clinical trial examining the effects of instructions for electronic cigarette use on smoking-related behaviors and biomarkers of exposure. Nicotine & Tobacco Research, 22(9), 1524–1532. https://doi.org/10.1093/ntr/ntz233 | Wrong exposure |
| Hattatoglu D.G. & Yildiz B.P. (2021). Long-term relapse outcomes of smoking cessation in older smokers. Haseki Tip Bulteni, 59(5), 405–410. https://doi.org/10.4274/haseki.galenos.2021.7376 | Wrong outcome |
| Hayes, L., McParlin, C., Azevedo, L. B., Jones, D., Newham, J., Olajide, J., McCleman, L., & Heslehurst, N. (2021). The Effectiveness of Smoking Cessation, Alcohol Reduction, Diet and Physical Activity Interventions in Improving Maternal and Infant Health Outcomes: A Systematic Review of Meta-Analyses. Nutrients, 13(3). https://doi.org/10.3390/nu13031036 | Wrong study design |
| Hayes, R. B., Dunsiger, S., & Borrelli, B. (2010). The influence of quality of life and depressed mood on smoking cessation among medically ill smokers. J Behav Med, 33(3), 209–218. https://doi.org/10.1007/s10865-010-9254-z | Mental health measured at baseline only, no follow up |
| Hayford, K. E., Patten, C. A., Rummans, T. A., Schroeder, D. R., Offord, K. P., Croghan, I. T., Glover, E. D., Sachs, D. P., & Hurt, R. D. (1999). Efficacy of bupropion for smoking cessation in smokers with a former history of major depression or alcoholism. Br J Psychiatry, 174, 173–178. https://doi.org/10.1192/bjp.174.2.173 | Did not analyse mental health outcomes by exposure of interest |
| Hays JT, Croghan IT, Baker CL, Cappelleri JC, & Bushmakin AG. (2012). Changes in health-related quality of life with smoking cessation treatment. European Journal of Public Health, 22(2), 224–229. https://doi.org/10.1093/eurpub/ckq137 | Wrong outcome |
| Heffner J.L., Kelly M.M., Waxmonsky J., Mattocks K., Serfozo E., Bricker J.B., Mull K.E., Watson N.L., & Ostacher M. (2020). Pilot randomized controlled trial of web-delivered acceptance and commitment therapy versus smokefree.gov for smokers with bipolar disorder. Nicotine and Tobacco Research, 22(9), 1543–1552. https://doi.org/10.1093/ntr/ntz242 | Did not analyse mental health outcomes by exposure of interest |
| Heffner, J. L., Anthenelli, R. M., DelBello, M. P., L.S, D. P., & Strakowski, S. M. (2013). Mood management and nicotine patch for smoking cessation in adults with bipolar disorder. Nicotine and Tobacco Research, 15(11), 1805–1806. https://doi.org/10.1093/ntr/ntt076 | Did not analyse mental health outcomes by exposure of interest |
| Heffner, J. L., Evins, A. E., Russ, C., Lawrence, D., Ayers, C. R., McRae, T., Aubin, L. St., Krishen, A., West, R., & Anthenelli, R. M. (2019). Safety and efficacy of first-line smoking cessation pharmacotherapies in bipolar disorders: Subgroup analysis of a randomized clinical trial. Journal of Affective Disorders, 256, 267–277. https://doi.org/10.1016/j.jad.2019.06.008 | Did not analyse mental health outcomes by exposure of interest |
| Heffner, J. L., Mull, K. E., Watson, N. L., McClure, J. B., Bricker, J. B., & Babor, B. (2020). Long-term smoking cessation outcomes for sexual minority versus nonminority smokers in a large randomized controlled trial of two web-based interventions. Nicotine & Tobacco Research, 22(9), 1596–1604. https://doi.org/10.1093/ntr/ntz112 | Wrong study design |
| Heffner, J. L., Watson, N. L., Serfozo, E., Mull, K. E., MacPherson, L., Gasser, M., & Bricker, J. B. (2019). A behavioral activation mobile health app for smokers with depression: Development and pilot evaluation in a single-arm trial. *JMIR Formative Research*, *3*(4), e13728. | Did not analyse mental health outcomes by exposure of interest |
| Heggen, E., Omland, T., & Tonstad, S. (2022). Effect of smoking cessation on cardiac troponin I concentrations. Scandinavian Journal of Clinical and Laboratory Investigation, 82(2), 104–107. https://doi.org/10.1080/00365513.2022.2031277 | Wrong outcome |
| Herbec, A., Shahab, L., Brown, J., Ubhi, H. K., Beard, E., Matei, A., & West, R. (2021). Does addition of craving management tools in a stop smoking app improve quit rates among adult smokers? Results from BupaQuit pragmatic pilot randomised controlled trial. Digital Health, 7(101690863), 20552076211058935. https://doi.org/10.1177/20552076211058935 | Wrong study design |
| Hermle, L., Bessey, C., Vasic, N., Uwe Petersen, K., & Batra, A. (2019). Tabakentwohnungsbehandlung bei Heimbewohnern mit schweren psychischen Storungen (SMI). [German]. Sucht, 65(1), 13–21. https://doi.org/10.1024/0939-5911/a000575 | Wrong study design |
| Higuchi, Y., Fujiwara, M., Nakaya, N., Fujimori, M., Hayashibara, C., So, R., Shinkawa, I., Sato, K., Yada, Y., Kodama, M., Takenaka, H., Kishi, Y., Kakeda, K., Uchitomi, Y., Yamada, N., Inagaki, M., & Ahmed, B. (2019). Change in smoking cessation stage over 1 year in patients with schizophrenia: A follow up study in Japan. BMC Psychiatry, 19. https://doi.org/10.1186/s12888-019-2351-9 | Wrong exposure |
| Hillebrandt, M.-A. (2022). Impact of changes in relationship status on smoking behavior and body weight. Economics and Human Biology, 44(101166135), 101077. https://doi.org/10.1016/j.ehb.2021.101077 | Wrong exposure |
| Hills A., Allison C., & Allison A. (2022). 80 The cost of smoking in pregnancy—A retrospective study. European Journal of Obstetrics and Gynecology and Reproductive Biology, 270, e56. https://doi.org/10.1016/j.ejogrb.2021.11.198 | Wrong exposure |
| Hintz, F., Geiser, C., & Shiffman, S. (2019). A latent state-trait model for analyzing states, traits, situations, method effects, and their interactions. Journal of Personality, 87(3), 434–454. https://doi.org/10.1111/jopy.12400 | Did not analyse mental health outcomes by exposure of interest |
| Hirvonen E., Stepanov M., Kilpelainen M., Lindqvist A., & Laitinen T. (2019). Consistency and reliability of smoking-related variables: Longitudinal study design in asthma and COPD. European Clinical Respiratory Journal, 6(1), 1591842. https://doi.org/10.1080/20018525.2019.1591842 | Wrong outcome |
| Hitsman, B., Papandonatos, G. D., Gollan, J. K., Huffman, M. D., Niaura, R., Mohr, D. C., Veluz-Wilkins, A. K., Lubitz, S. F., Hole, A., Leone, F. T., Khan, S. S., Fox, E. N., Bauer, A.-M., Wileyto, E. P., Bastian, J., & Schnoll, R. A. (2023). Efficacy and safety of combination behavioral activation for smoking cessation and varenicline for treating tobacco dependence among individuals with current or past major depressive disorder: A 2 x 2 factorial, randomized, placebo-controlled trial. *Addiction*, *118*(9), 1710–1725. https://doi.org/10.1111/add.16209 | Did not analyse mental health outcomes by exposure of interest |
| Ho, L. F., Ho, W. K., Wong, L. L., Chiu, S. W., Tang, S. Y., Wong, C. M., Chan, K. S., Lam, C. L., Chen, M., Chan, K. L., Lin, G., Ng, B. F.-L., & Lin, Z. X. (2022). Acupuncture combined with auricular acupressure for smoking cessation and its effects on tobacco dependence and smoking behavior among Hong Kong smokers: A multicenter pilot clinical study. Chinese Medicine, 17(1), 92. https://doi.org/10.1186/s13020-022-00649-w | Wrong study design |
| Hoepper, B. B., Siegel, K. R., Carlon, H. A., Kahler, C. W., Park, E. R., Taylor, S. T., Simpson, H. V., & Hoeppner, S. S. (2022). Feature-Level Analysis of a Smoking Cessation Smartphone App Based on a Positive Psychology Approach: Prospective Observational Study. JMIR Formative Research, 6(7), e38234. https://doi.org/10.2196/38234 | Wrong study design |
| Hoeppner, B. B., Hoeppner, S. S., Schick, M. R., Milligan, C. M., Helmuth, E., Bergman, B. G., Abroms, L. C., Kelly, J. F., & Baker, B. (2019). Using the text-messaging program SmokefreeTXT to support smoking cessation for nondaily smokers. Substance Use & Misuse, 54(8), 1260–1271. https://doi.org/10.1080/10826084.2018.1552300 | Wrong outcome |
| Hoeppner, S. S., Carlon, H. A., Kahler, C. W., Park, E. R., Darville, A., Rohsenow, D. J., & Hoeppner, B. B. (2021). COVID-19 Impact on Smokers Participating in Smoking Cessation Trials: The Experience of Nondaily Smokers Participating in a Smartphone App Study. Telemedicine Reports, 2(1), 179–187. https://doi.org/10.1089/tmr.2021.0008 | Wrong outcome |
| Hofmeyr, A., Kincaid, H., Rusch, O., & Alessi, C. (2020). Incentivizing university students to quit smoking: A randomized controlled trial of a contingency management intervention in a developing country. The American Journal of Drug and Alcohol Abuse, 46(1), 109–119. https://doi.org/10.1080/00952990.2019.1622130 | Wrong outcome |
| Hollis E.S., Ruebush E., Davies E., Paraghamian S., & Weiner A.A. (2021). Smoking Cessation Interventions During Chemoradiotherapy for Locally Advanced Cervical Cancer: A Missed Opportunity? International Journal of Radiation Oncology Biology Physics, 111(3 Supplement), e613–e614. https://doi.org/10.1016/j.ijrobp.2021.07.1634 | Not primary research |
| Holstein, J. A., O’Hara, K., Moss, A., Lowary, M., Kerby, G., Hovell, M., Klein, J. D., Winickoff, J. P., & Wilson, K. (2022). Barriers and Motivators for Smoking Cessation Among Caregivers of Inpatient Pediatric Patients. Hospital Pediatrics, 12(2), 220–228. https://doi.org/10.1542/hpeds.2021-005984 | Wrong outcome |
| Hong AS, Elrashidi MY, Schroeder DR, & Ebbert JO. (2015). Depressive symptoms among patients receiving varenicline and bupropion for smoking cessation. Journal of Substance Abuse Treatment, 52, 78–81. https://doi.org/10.1016/j.jsat.2014.11.010 | Did not analyse mental health outcomes by exposure of interest |
| Hong H// Wang W// Chen L. (2015). Study on effect of 5A intervention method combined with varenicline for smoking cessation of COPD patients in stable stage. Chinese Nursing Research, 29(2C), 667–670. https://doi.org/10.3969/j.issn.1009-6493.2015.06.009 | Wrong outcome |
| Hood NE, Ferketich AK, Paskett ED, & Wewers ME. (2013). Treatment adherence in a lay health adviser intervention to treat tobacco dependence. Health Education Research, 28(1), 72–82. https://doi.org/10.1093/her/cys081 | Mental health measured at baseline only, no follow up |
| Hoogwegt, M. T., Hoeks, S. E., Pedersen, S. S., Scholte op Reimer, W. J. M., van Gestel, Y. R. B. M., Verhagen, H. J. M., & Poldermans, D. (2010). Smoking cessation has no influence on quality of life in patients with peripheral arterial disease 5 years post-vascular surgery. Eur J Vasc Endovasc Surg, 40(3), 355–362. https://doi.org/10.1016/j.ejvs.2010.05.013 | Wrong outcome |
| Horinouchi, T., Yoshida, N., Toihata, T., Harada, K., Eto, K., Ogawa, K., Sawayama, H., Iwatsuki, M., Baba, Y., Miyamoto, Y., & Baba, H. (2022). Postoperative respiratory morbidity can adversely affect prognosis in thoracoscopic esophagectomy for esophageal cancer: A retrospective study. Surgical Endoscopy, vbf, 8806653. https://doi.org/10.1007/s00464-022-09711-y | Wrong exposure |
| Hovhannisyan, K., Rasmussen, M., Adami, J., Wikstrom, M., Tonnesen, H., & Apollonio, B. (2020). Evaluation of Very Integrated Program: Health promotion for patients with alcohol and drug addiction-A randomized trial. Alcoholism: Clinical and Experimental Research, 44(7), 1456–1467. https://doi.org/10.1111/acer.14364 | Wrong study design |
| Hu, M., Yin, H., Shu, X., Jia, Y., Leng, M., Chen, L., & Almeida, A. (2019). Multi-angles of smoking and mild cognitive impairment: Is the association mediated by sleep duration? Neurological Sciences, 40(5), 1019–1027. https://doi.org/10.1007/s10072-019-03750-5 | Mental health measured at baseline only, no follow up |
| Hu, N., Yu, Z., Du, Y., & Li, J. (2022). Risk Factors of Relapse After Smoking Cessation: Results in China Family Panel Studies From 2010 to 2018. Frontiers in Public Health, 10(101616579), 849647. https://doi.org/10.3389/fpubh.2022.849647 | Wrong study design |
| Huang, S., Chen, Q., Griffin, P., Liu, G., & Azagba, S. (2023). Longitudinal transitions in tobacco use in youth and young adults: A latent transition analysis of the population assessment of tobacco and health study from Wave 1 to 5. Addictive Behaviors, 138(2gw, 7603486), 107548. https://doi.org/10.1016/j.addbeh.2022.107548 | Not adult population |
| Huang, S., Wahlquist, A., & Dahne, J. (2024). Individual predictors of response to a behavioral activation-based digital smoking cessation intervention: A machine learning approach. *Substance Use & Misuse*, *59*(11), 1620–1628. https://doi.org/10.1080/10826084.2024.2369155 | Wrong study design |
| Huang, Y., Emery, J., Naughton, F., Cooper, S., McDaid, L., Dickinson, A., Clark, M., Kinahan-Goodwin, D., Thomson, R., Phillips, L., Lewis, S., Orton, S., & Coleman, T. (2022). The development and acceptability testing of an app-based smart survey system to record smoking behaviour, use of nicotine replacement therapy (NRT) and e-cigarettes. BMC Research Notes, 15(1), 100. https://doi.org/10.1186/s13104-022-05983-8 | Wrong study design |
| Huang, Z., Chang, X., Wang, L., Liu, J., Heng, C.-K., Khor, C.-C., Yuan, J.-M., Koh, W.-P., & Dorajoo, R. (2022). Interaction between cigarette smoking and genetic polymorphisms on the associations with age of natural menopause and reproductive lifespan: The Singapore Chinese Health Study. Human Reproduction, 37(6), 1351–1359. https://doi.org/10.1093/humrep/deac075 | Wrong exposure |
| Huffman, A. L., Bromberg, J. E., & Augustson, E. M. (2018). Lifetime Depression, Other Mental Illness, and Smoking Cessation. *American Journal of Health Behavior*, *42*(4), 90–101. | Wrong outcome |
| Hughes J.R., Klemperer E.M., & Peasley-Miklus C. (2020). Possible new symptoms of tobacco withdrawal II: Anhedonia—A systematic review. Nicotine and Tobacco Research, 22(1), 11–17. https://doi.org/10.1093/ntr/nty171 | Wrong study design |
| Hughes, J. R. (1992). Tobacco withdrawal in self-quitters. J Consult Clin Psychol, 60(5), 689–697. https://doi.org/10.1037//0022-006x.60.5.689 | Follow up < 6 weeks |
| Humfleet, G. L., Hall, S. M., Delucchi, K. L., & Dilley, J. W. (2013). A randomized clinical trial of smoking cessation treatments provided in HIV clinical care settings. Nicotine and Tobacco Research, 15(8), 1436–1445. https://doi.org/10.1093/ntr/ntt005 | Mental health measured at baseline only, no follow up |
| Hunt L., Covinsky K., Stijacic Cenzer I., Boscardin J., Leutwyler H., Lee A., & Cataldo J. (2021). Epidemiology of smoking in U.S. older adults, 1998-2016. Journal of the American Geriatrics Society, 69(SUPPL 1), S99–S100. https://doi.org/10.1111/jgs.17115 | Wrong study design |
| Hurt, R. D., Sachs, D. P., Glover, E. D., Offord, K. P., Johnston, J. A., Dale, L. C., Khayrallah, M. A., Schroeder, D. R., Glover, P. N., Sullivan, C. R., Croghan, I. T., & Sullivan, P. M. (1997). A comparison of sustained-release bupropion and placebo for smoking cessation. N Engl J Med, 337(17), 1195–1202. https://doi.org/10.1056/NEJM199710233371703 | Did not analyse mental health outcomes by exposure of interest |
| Husted, K. L. S., Dandanell, S., Petersen, J., Dela, F., Helge, J. W., & Allegrante, B. (2020). The effectiveness of body age-based intervention in workplace health promotion: Results of a cohort study on 9851 Danish employees. PLoS ONE, 15(9). https://doi.org/10.1371/journal.pone.0239337 | Wrong study design |
| Hwong, A. R., Schmittdiel, J., Schillinger, D., Newcomer, J. W., Essock, S., Zhu, Z., Dyer, W., Young-Wolff, K. C., Mangurian, C., & Bennett, D. (2021). Smoking cessation treatment for individuals with comorbid diabetes and serious mental illness in an integrated health care delivery system. Addictive Behaviors, 114. https://doi.org/10.1016/j.addbeh.2020.106697 | Wrong exposure |
| Ikeda, T., Cooray, U., Murakami, M., & Osaka, K. (2023). Assessing the impacts of smoking cessation and resumption on back pain risk in later life. *European Journal of Pain*, *27*(8), 973–980. https://doi.org/10.1002/ejp.2139 | Did not analyse mental health outcomes by exposure of interest |
| Ikonomidis I., Thymis J., Kourea K., Kostelli G., Neocleous A., Katogiannis K., Makavos G., Polyzogopoulou E., Plotas P., Lambadiari V., & Parissis J. (2022). Fagerstrom score predicts smoking status six months after hospitalization for acute myocardial infarction: A prospective study. Hellenic Journal of Cardiology, 67, 28–35. https://doi.org/10.1016/j.hjc.2022.05.007 | Wrong study design |
| Inoue, Y., Katoh, T., Masuda, S., Lu, X., Koga, T., Sadohara, T., Sadanaga, M., & Tanaka, E. (2020). Perioperative complications of abdominal surgery in smokers. Journal of Anesthesia, 34(5), 712–718. https://doi.org/10.1007/s00540-020-02815-6 | Wrong study design |
| IRCT2017011932057N1. (2017). Effectiveness Of acceptance and commitment therapy on male smokers. *Http://Www.Who.Int/Trialsearch/Trial2.Aspx?TrialID=IRCT2017011932057N1*. | Did not analyse mental health outcomes by exposure of interest |
| ISRCTN11214785. (2018). The STOP project—A trial of a ‘quit smoking’ intervention in pregnancy to achieve better outcomes for mother and baby. *Http://Www.Who.Int/Trialsearch/Trial2.Aspx?TrialID=ISRCTN11214785*. | Wrong outcome |
| ISRCTN16022919. (2016). Mobile health interventions for smoking cessation services uptake and smoking cessation: A factorial randomised trial in Thailand. *Http://Www.Who.Int/Trialsearch/Trial2.Aspx?TrialID=ISRCTN16022919*. | Wrong outcome |
| ISRCTN47776579. (2017). A trial of physical activity assisted reduction of smoking. *Http://Www.Who.Int/Trialsearch/Trial2.Aspx?TrialID=ISRCTN47776579*. | Wrong outcome |
| ISRCTN65853476. (2018). Is a mobile phone app delivering cognitive behavioural therapy effective at helping people quit smoking? *Http://Www.Who.Int/Trialsearch/Trial2.Aspx?TrialID=ISRCTN65853476*. | Wrong outcome |
| ISRCTN99531779. (2018). The ESCAPE study: Offering smoking cessation treatment as part of routine psychological care. *Http://Www.Who.Int/Trialsearch/Trial2.Aspx?TrialID=ISRCTN99531779*. | Did not analyse mental health outcomes by exposure of interest |
| Itani, T., Rai, D., Jones, T., Taylor, G. M. J., Thomas, K. H., Martin, R. M., Munafo, M. R., Davies, N. M., & Taylor, A. E. (2019). Long-term effectiveness and safety of varenicline and nicotine replacement therapy in people with neurodevelopmental disorders: A prospective cohort study. *Scientific Reports*, *9*(1), 19488. | Wrong outcome |
| Jaber Filho J.A., Verissimo J., Hollanda A., Tome A., & Geraldes P.C. (2019). A non smoking program in the psychiatric clinic: Eradicating tobacco from the therapeutic space. European Psychiatry, 56(Supplement 1), S185. https://doi.org/10.1016/j.eurpsy.2019.01.003 | Wrong study design |
| Jaen-Moreno M.J., Feu N., Redondo-Ecija J., Montiel F.J., Gomez C., Del Pozo G.I., Alcala J.A., Gutierrez-Rojas L., Balanza-Martinez V., Chauca G.M., Carrion L., Osuna M.I., Sanchez M.D., Caro I., Ayora M., Valdivia F., Lopez M.S., Poyato J.M., & Sarramea F. (2019). Smoking cessation opportunities in severe mental illness (tobacco intensive motivational and estimate risk—TIMER -): Study protocol for a randomized controlled trial. Trials, 20(1), 47. https://doi.org/10.1186/s13063-018-3139-9 | Wrong study design |
| Jaen-Moreno M.J., Ruiz Rull C., Valdivia F., Valverde M., Osuna M.I., Caro I., Martin D., Montiel F.J., & Sarramea Crespo F. (2020). Tobacco intensive motivational and estimate risk (timer). Study protocol for a randomized controlled trial. European Psychiatry, 63(Supplement 1), S532–S533. https://doi.org/10.1192/j.eurpsy.2020.6 | Wrong study design |
| Japuntich, S. J., Dunne, E. M., Krieger, N. H., Ryan, P. M., Rogers, E., Sherman, S. E., & Fu, S. S. (2019). Proactive tobacco treatment in a behavioral health home. *Community Mental Health Journal*, *13*, 13. | Wrong outcome |
| Japuntich, S. J., Lee, L. O., Pineles, S. L., Gregor, K., Joos, C. M., Patton, S. C., Krishnan-Sarin, S., & Rasmusson, A. M. (2019). Contingency management and cognitive behavioral therapy for trauma-exposed smokers with and without posttraumatic stress disorder. Addictive Behaviors, 90, 136–142. https://doi.org/10.1016/j.addbeh.2018.10.042 | Mental health measured at baseline only, no follow up |
| Japuntich, S. J., Smith, S. S., Jorenby, D. E., Piper, M. E., Fiore, M. C., & Baker, T. B. (2007). Depression predicts smoking early but not late in a quit attempt. Nicotine Tob Res, 9(6), 677–686. https://doi.org/10.1080/14622200701365301 | Mental health measured at baseline only, no follow up |
| Jardin C, Bakhshaie J, Schmidt NB, Sharp C, & Zvolensky M. (2015). Examination of smoking inflexibility AS a mechanism linking anxiety sensitivity and severity of smoking behavior. American Journal on Addictions, 24(4), 374–381. https://doi.org/10.1111/ajad.12203 | Mental health measured at baseline only, no follow up |
| Javitz, H. S., Bush, T. M., Lovejoy, J. C., Torres, A. J., Wetzel, T., Wassum, K. P., Tan, M. M., Alshurafa, N., Spring, B., & Adams, B. (2019). Six month abstinence heterogeneity in the Best Quit Study. Annals of Behavioral Medicine, 53(12), 1032–1044. https://doi.org/10.1093/abm/kaz014 | Wrong outcome |
| Jeemon P., Harikrishnan S., Ganapathi S., Sivasankaran S., Binukumar B., Padmanabhan S., Tandon N., & Prabhakaran D. (2021). Efficacy of a family-based cardiovascular risk reduction intervention in individuals with a family history of premature coronary heart disease in India (PROLIFIC): An open-label, single-centre, cluster randomised controlled trial. The Lancet Global Health, 9(10), e1442–e1450. https://doi.org/10.1016/S2214-109X%2821%2900319-3 | Wrong study design |
| Jeffries, E. R. (2022). A randomized controlled trial of a hatha yoga intervention for smokers. *Dissertation Abstracts International: Section B: The Sciences and Engineering*, *83*(11-B), No-Specified. | Wrong study design |
| Jeon, H. G., Lee, K. C., & Abroms, B. (2020). Relationship between the use of a smoking cessation mobile health application and emotional attitudes. Social Behavior and Personality: An International Journal, 48(1), 1–14. https://doi.org/10.2224/sbp.8554 | Wrong study design |
| Jeong, B.-Y., Lim, M.-K., Shin, S.-H., Han, Y.-R., Oh, J.-K., & Lee, H.-J. (2021). Factors Associated with the 30-Day and 1-Year Smoking Abstinence of Women in Korea: The Effect of Nicotine Dependency, Self-Efficacy, and Mental Illness. International Journal of Environmental Research and Public Health, 18(21). https://doi.org/10.3390/ijerph182111171 | Wrong outcome |
| Jeong, K., Son, H., Kim, S., Kim, J., & Cho, H. J. (2024). The longitudinal relationship between changes in smoking and depression in older male adults. *Journal of Men’s Health*, *20*(3), 106-112. https://doi.org/10.22514/jomh.2024.044 | Did not distinguish between unsuccessful quit attempt and no attempt to quit |
| Jimenez Ruiz, C. A., Pinedo, A. R., Guerrero, A. C., Ulibarri, M. M., Fernandez, M. C., & Gonzalez, G. L. (2012). Characteristics of COPD smokers and effectiveness and safety of smoking cessation medications. Nicotine and Tobacco Research, 14(9), 1035–1039. https://doi.org/10.1093/ntr/nts001 | Mental health not measured before quit attempt |
| Jimenez-Ruiz, C. A., Pascual-Lledo, J. F., Cicero-Guerrero, A., Cristobal-Fernandez, M., Mayayo-Ulibarri, M., & Villar-Laguna, C. (2018). Effectiveness and safety of varenicline and nicotine replacement therapy among mental health patients: A retrospective cohort study. Pulmonology, 24(1), 10–15. https://doi.org/10.1016/j.rppnen.2017.10.008 | Mental health measured at baseline only, no follow up |
| Jizzini M., Raghavendra A.S., Ibrahim N.K., Kypriotakis G., Cinciripini P.M., Seoudy K., & Karam-Hage M.A. (2019). The impact of smoking cessation on breast cancer patients’ survival. Journal of Clinical Oncology, 37(Supplement 15). https://doi.org/10.1200/JCO.2019.37.15-suppl.542 | Wrong study design |
| Johnson A.C., Simmens S.J., Turner M.M., Evans W.D., Strasser A.A., & Mays D. (2022). Longitudinal effects of cigarette pictorial warning labels among young adults. Journal of Behavioral Medicine, 45(1), 124–132. https://doi.org/10.1007/s10865-021-00258-2 | Wrong study design |
| Johnson KA, Farris SG, Schmidt NB, & Zvolensky MJ. (2012). Anxiety sensitivity and cognitive-based smoking processes: Testing the mediating role of emotion dysregulation among treatment-seeking daily smokers. Journal of Addictive Diseases, 31(2), 143–157. https://doi.org/10.1080/10550887.2012.665695 | Mental health measured at baseline only, no follow up |
| Johnson KA, Farris SG, Schmidt NB, Smits JA, & Zvolensky MJ. (2013). Panic attack history and anxiety sensitivity in relation to cognitive-based smoking processes among treatment-seeking daily smokers. Nicotine & Tobacco Research, 15(1), 1–10. https://doi.org/10.1093/ntr/ntr332 | Mental health measured at baseline only, no follow up |
| Johnson, A. L., Kaye, J., Baker, T. B., Fiore, M. C., Cook, J. W., & Piper, M. E. (2019). Psychiatric comorbidities in a comparative effectiveness smoking cessation trial: Relations with cessation success, treatment response, and relapse risk factors. *Drug & Alcohol Dependence*, *207*, 107796. | Mental health measured at baseline only, no follow up |
| Johnson, A. L., O’Bryan, E. M., Kraemer, K. M., McLeish, A. C., Zvolensky, M. J., Bernstein, J. A., Horning, D. R., & Althuis, A. (2019). The role of anxiety sensitivity-physical concerns in terms of quit day withdrawal symptoms and cravings: A pilot test among smokers with asthma. Journal of Asthma, 56(2), 173–178. https://doi.org/10.1080/02770903.2018.1437175 | Follow up < 6 weeks |
| Johnson, E. O., & Breslau, N. (2006). Is the association of smoking and depression a recent phenomenon? Nicotine Tob Res, 8(2), 257–262. https://doi.org/10.1080/14622200600576644 | Wrong exposure |
| Johnson, K. M., Sadatsafavi, M., Adibi, A., Lynd, L., Harrison, M., Tavakoli, H., Sin, D. D., & Bryan, S. (2021). Cost Effectiveness of Case Detection Strategies for the Early Detection of COPD. Applied Health Economics and Health Policy, 19(2), 203–215. https://doi.org/10.1007/s40258-020-00616-2 | Did not analyse mental health outcomes by exposure of interest |
| Johnson, S. E., Mitrou, F., Lawrence, D., Zubrick, S. R., Wolstencroft, K., Ennals, P., Hall, C., McNaught, E., & Annamalai, A. (2020). Feasibility of a Consumer Centred Tobacco Management intervention in community mental health services in Australia. Community Mental Health Journal, 56(7), 1354–1365. https://doi.org/10.1007/s10597-020-00573-z | Wrong study design |
| Jolly K, Sidhu M, Hewitt C, Daley A, Jordan R, Coventry P, Heneghan C, Jowett S, Singh S, Ives N, Adab P, Varghese J, Nunan D, Ahmed K, & Fitzmaurice D. (2017). Telephone health coaching in primary care patients with MRC I/II COPD: randomised controlled trial. European Respiratory Journal, 50(suppl 61), OA2914. https://doi.org/10.1183/1393003.congress-2017.OA2914 | Did not distinguish between unsuccessful quit attempt and no attempt to quit |
| Jones A.M., Carter-Harris L., Stiffler D., Macy J.T., Staten L.K., & Shieh C. (2020). Smoking Status and Symptoms of Depression During and After Pregnancy Among Low-Income Women. Journal of Obstetric, Gynecologic, and Neonatal Nursing : JOGNN, 49(4), 361–372. https://doi.org/10.1016/j.jogn.2020.05.006 | Wrong exposure |
| Jones M., Smith M., Lewis S., Parrott S., & Coleman T. (2019). A dynamic, modifiable model for estimating cost-effectiveness of smoking cessation interventions in pregnancy: Application to an RCT of self-help delivered by text message. Addiction, 114(2), 353–365. https://doi.org/10.1111/add.14476 | Wrong study design |
| Jones, S. (2024). Perceived ethnic discrimination and cigarette smoking cessation among a sample of people with HIV. *Dissertation Abstracts International Section A: Humanities and Social Sciences*, *85*(9-A). | Did not analyse mental health outcomes by exposure of interest |
| Jung, J. Y., & Yun, Y. H. (2022). Different effects between the current capability of and changes in self-management strategies on improving health behavior and psychological health after 6 months: Evidence from a prospective cohort study of patients with cancer. Quality of Life Research, 9210257, bqm. https://doi.org/10.1007/s11136-022-03320-x | Wrong study design |
| Jung, Y., Tagele, S. B., Son, H., Ibal, J. C., Kerfahi, D., Yun, H., Lee, B., Park, C. Y., Kim, E. S., Kim, S.-J., & Shin, J.-H. (2020). Modulation of Gut Microbiota in Korean Navy Trainees following a Healthy Lifestyle Change. Microorganisms, 8(9). https://doi.org/10.3390/microorganisms8091265 | Wrong study design |
| Kaasgaard, D. M., Sorensen, M. K., Christiansen, R. B., Breum, U. N., Asiamah, N., Friis, L. B. T., & Hjorth, P. (2024). Video consultation and treatment in the community smoking cessation therapy success rates in patients with mental illness: A randomized controlled trial. *Nordic Journal of Psychiatry*, *78*(4), 272-280. https://doi.org/10.1080/08039488.2024.2318305 | Wrong outcome |
| Kahler CW, Surace A, Gordon REF, Cioe PA, Spillane NS, Parks A, Bock BC, & Brown RA. (2018). Positive psychotherapy for smoking cessation enhanced with text messaging: Protocol for a randomized controlled trial. Contemporary Clinical Trials, 71(pp 146-153), 146–153. https://doi.org/10.1016/j.cct.2018.06.013 | Wrong outcome |
| Kahler, C. W., Spillane, N. S., & Metrik, J. (2010). Alcohol use and initial smoking lapses among heavy drinkers in smoking cessation treatment. Nicotine Tob Res, 12(7), 781–785. https://doi.org/10.1093/ntr/ntq083 | Wrong outcome |
| Kallio, A., Suominen, A., Tolvanen, M., Rantavuori, K., Jussila, H., Karlsson, L., Karlsson, H., & Lahti, S. (2023). Concurrent changes in dental anxiety and smoking in parents of the FinnBrain Birth Cohort Study. *European Journal of Oral Sciences,* *9504563*, e12912. https://doi.org/10.1111/eos.12912 | Wrong study design |
| Kamonkraingkrai S., Kaewpan W., Kalampakorn S., Sillabutra J., & Rungruanghiranya S. (2021). Effectiveness of Nicotine Withdrawal Symptoms Management Program Using LINE Application to Increase Self-Efficacy for Industrial Workers. Journal of the Medical Association of Thailand, 104(12), 1920–1929. https://doi.org/10.35755/jmedassocthai.2021.12.13025 | Wrong study design |
| Kang, S. H., Jeong, W., Jang, S.-I., & Park, E.-C. (2021). The effect of depression status change on daily cigarette smoking amount according to sex: An eleven-year follow up study of the Korea Welfare Panel Study. BMC Public Health, 21(1), 1304. https://doi.org/10.1186/s12889-021-11362-y | Wrong study design |
| Kaplan, B., Thrul, J., & Cohen, J. E. (2021). Association of cigarette and electronic nicotine delivery systems use with internalizing and externalizing problems among US adults: Findings from wave 3 (2015-2016) of the PATH study. PloS One, 16(6), e0253061. https://doi.org/10.1371/journal.pone.0253061 | Wrong study design |
| Karadogan, D., Onal, O., & Kanbay, Y. (2019a). Corrigendum: How does reimbursement status affect smoking cessation interventions? A real-life experience from the Eastern Black Sea region of Turkey. Tobacco Induced Diseases, 17(101201591), 67. https://doi.org/10.18332/tid/112274 | Mental health measured at baseline only, no follow up |
| Karadogan, D., Onal, O., & Kanbay, Y. (2019b). How does reimbursement status affect smoking cessation interventions? A real-life experience from the Eastern Black Sea region of Turkey. Tobacco Induced Diseases, 17(101201591), 05. https://doi.org/10.18332/tid/100412 | Wrong outcome |
| Karch, S., Paolini, M., Gschwendtner, S., Jeanty, H., Reckenfelderbaumer, A., Yaseen, O., Maywald, M., Fuchs, C., Rauchmann, B.-S., Chrobok, A., Rabenstein, A., Ertl-Wagner, B., Pogarell, O., Keeser, D., Ruther, T., & Addicott, A. (2019). Real-time fMRI neurofeedback in patients with tobacco use disorder during smoking cessation: Functional differences and implications of the first training session in regard to future abstinence or relapse. Frontiers in Human Neuroscience, 13. https://doi.org/10.3389/fnhum.2019.00065 | Wrong exposure |
| Karl, D., Wieland, A., Shevchenko, Y., Grundinger, N., Machunze, N., Gerhardt, S., Flor, H., & Vollstadt-Klein, S. (2023). Using computer-based habit versus chess-based cognitive remediation training as add-on therapy to modify the imbalance between habitual behavior and cognitive control in tobacco use disorder: Protocol of a randomized controlled, fMRI study. *BMC Psychology, 11*(1), 24. https://doi.org/10.1186/s40359-023-01055-z | Wrong study design |
| Karlsson M.L., Hertzberg-Nyquist K., Saevarsdottir S., Lundberg I.E., Demmelmaie I., Pettersson S., & Chatzidionysiou K. (2021). Hpr the effect of a person-centered smoking cessation program in rheumatoid arthritis patients in a rheumatology outpatient clinic setting -Results of an interventional feasibility study. Annals of the Rheumatic Diseases, 80(SUPPL 1), 93–94. https://doi.org/10.1136/annrheumdis-2021-eular.1310 | Wrong study design |
| Katara P. (2021). Tobacco cessation services and medications to quit tobacco for NCD patients. Tobacco Induced Diseases, 19(SUPPL 1). https://doi.org/10.18332/tid/141417 | Wrong study design |
| Katsaounou P., Korkotzelou A., Driva M., Schoretsaniti S., Barbaressou Z., Osarogue A., Saltiagianni V., Vasileiou V., Gyftopoulos S., Tentolouris N., & Tonstad S. (2019). Smoking cessation in diabetic patients. Tobacco Induced Diseases, 17(SUPPL 1). https://doi.org/10.18332/tid/111604 | Wrong study design |
| Kaufmann, A. (Lily). (2022). Does changing outcome expectancies and behavioral reinforcers affect smoking-related outcomes among young adults? *Dissertation Abstracts International: Section B: The Sciences and Engineering*, *83*(2-B), No-Specified. | Wrong study design |
| Kay, H., Silver, S. N., Smith, A. B., Basak, R., Shoenbill, K., Usinger, D., Goldstein, A. O., Matulewicz, R. S., Chen, R., & Bjurlin, M.A. (2024). Bladder Cancer Survivors Who Do Not Smoke Have Better Longitudinal Health-Related Quality of Life Measures: An Assessment of the Comparative Effectiveness and Survivorship Health in Bladder Cancer (CEASE-BC) Study. *Journal of Urology*, *212*(1), 87-94. https://doi.org/10.1097/JU.0000000000003964 | Wrong outcome |
| Kaye, J. T., Johnson, A. L., Baker, T. B., Piper, M. E., Cook, J. W., & Baker, C. (2020). Searching for personalized medicine for binge drinking smokers: Smoking cessation using varenicline, nicotine patch, or combination nicotine replacement therapy. Journal of Studies on Alcohol and Drugs, 81(4), 426–435. https://doi.org/10.15288/jsad.2020.81.426 | Wrong study design |
| Keith, D. R., Skelly, J., Tang, K. J., Kurti, A. N., Higgins, S. T., & Anesi, A. (2021). Household-smoking bans are associated with reduced nicotine exposure, increased smoking abstinence, and improved birth outcomes among pregnant women enrolled in smoking-cessation treatment. Experimental and Clinical Psychopharmacology, 29(4), 366–374. https://doi.org/10.1037/pha0000426 | Mental health measured at baseline only, no follow up |
| Kelly MM, Grant C, Cooper S, & Cooney JL. (2013). Anxiety and smoking cessation outcomes in alcohol-dependent smokers. Nicotine & Tobacco Research, 15(2), 364–375. https://doi.org/10.1093/ntr/nts132 | Mental health measured at baseline only, no follow up |
| Kelly PJ, Baker AL, Townsend CJ, Deane FP, Callister R, Collins CE, Ingram I, Keane C, & Beck AK. (2019). Healthy recovery: A pilot study of a smoking and other health behavior change intervention for people attending residential alcohol and other substance dependence treatment. Journal of Dual Diagnosis, 15(3), 207–216. https://doi.org/10.1080/15504263.2019.1612537 | Did not analyse mental health outcomes by exposure of interest |
| Kelly, M. E., Guillot, C. R., Quinn, E. N., Lucke, H. R., Bello, M. S., Pang, R. D., & Leventhal, A. M. (2020). Anxiety sensitivity in relation to cigarette smoking and other substance use in African American smokers. Psychology of Addictive Behaviors : Journal of the Society of Psychologists in Addictive Behaviors, 34(6), 669–679. https://doi.org/10.1037/adb0000573 | Wrong study design |
| Kelly, P. J., Baker, A. L., Fagan, N. L., Turner, A., Deane, F., McKetin, R., Callister, R., Collins, C., Ingram, I., Wolstencroft, K., Townsend, C., Osborne, B. A., Zimmermann, A., & Attkisson, B. (2020). Better Health Choices: Feasability and preliminary effectiveness of a peer delivered healthy lifestyle intervention in a community mental health setting. Addictive Behaviors, 103. https://doi.org/10.1016/j.addbeh.2019.106249 | Wrong study design |
| Kendzor D.E., Businelle M.S., Vidrine D.J., Frank-Pearce S.G., Shih Y.-C.T., Dallery J., Alexander A.C., Boozary L.K., Waring J.J.C., & Ehlke S.J. (2022). Mobile contingency management for smoking cessation among socioeconomically disadvantaged adults: Protocol for a randomized trial. Contemporary Clinical Trials, 114, 106701. https://doi.org/10.1016/j.cct.2022.106701 | Wrong study design |
| Kenfield, S. A., Van Blarigan, E. L., Ameli, N., Lavaki, E., Cedars, B., Paciorek, A. T., Monroy, C., Tantum, L. K., Newton, R. U., Signorell, C., Suh, J. H., Zhang, L., Cooperberg, M. R., Carroll, P. R., & Chan, J. M. (2019). Feasibility, Acceptability, and Behavioral Outcomes from a Technology-enhanced Behavioral Change Intervention (Prostate 8): A Pilot Randomized Controlled Trial in Men with Prostate Cancer. European Urology, 75(6), 950–958. https://doi.org/10.1016/j.eururo.2018.12.040 | Wrong exposure |
| Kenney, B. A., Holahan, C. J., Holahan, C. K., Brennan, P. L., Schutte, K. K., & Moos, R. H. (2009). Depressive symptoms, drinking problems, and smoking cessation in older smokers. Addict Behav, 34(6–7), 548–553. https://doi.org/10.1016/j.addbeh.2009.03.020 | Did not analyse mental health outcomes by exposure of interest |
| Kerkvliet, J. L., Wey, H., & Fahrenwald, N. L. (2015). Cessation among state quitline participants with a mental health condition. Nicotine and Tobacco Research, 17(6), 735–741. https://doi.org/10.1093/ntr/ntu239 | Mental health measured at baseline only, no follow up |
| Kertes J., Stein Reisner O., Grunhaus L., Nezry R., Alcalay T., Azuri J., & Neumark Y. (2022). Comparison of Smoking Cessation Program Registration, Participation, Smoking Cessation Medication Utilization, and Abstinence Rates Between Smokers With and Without Schizophrenia, Schizo-affective Disorder, or Bipolar Disorder. Nicotine and Tobacco Research, 24(5), 670–678. https://doi.org/10.1093/ntr/ntab202 | Wrong outcome |
| Khaled, S. M., Bulloch, A. G., Williams, J. V. A., Hill, J. C., Lavorato, D. H., & Patten, S. B. (2012). Persistent heavy smoking as risk factor for major depression (MD) incidence—Evidence from a longitudinal Canadian cohort of the National Population Health Survey. Journal of Psychiatric Research, 46(4), 436–443. https://doi.org/10.1016/j.jpsychires.2011.11.011 | Wrong exposure |
| Khaled, S. M., Bulloch, A., Exner, D. V., & Patten, S. B. (2009). Cigarette smoking, stages of change, and major depression in the Canadian population. Can J Psychiatry, 54(3), 204–208. https://doi.org/10.1177/070674370905400309 | Wrong study design |
| Khan, J. S., Hah, J. M., Mackey, S. C., & Austin, A. (2019). Effects of smoking on patients with chronic pain: A propensity-weighted analysis on the Collaborative Health Outcomes Information Registry. Pain, 160(10), 2374–2379. https://doi.org/10.1097/j.pain.0000000000001631 | Wrong outcome |
| Khomenya A., Vershinina M., & Foronosova O. (2020). Does Smoking Cessation affect symptoms and FEV1 in smokers with mild COPD? European Respiratory Journal, 56(Supplement 64). https://doi.org/10.1183/13993003.congress-2020.1313 | Wrong study design |
| Killi, A. E., Baspinar, M. M., & Basat, O. (2020). Association between post-cessation weight gain and eating behavior changes. Northern Clinics of Istanbul, 7(2), 153–160. https://doi.org/10.14744/nci.2019.58712 | Wrong outcome |
| Kim E., Williams R.M., Eyestone E., Cordon M., Smith L., Davis K., Luta G., Anderson E.D., McKee B., Batlle J., Ramsaier M., Howell J., Parikh V., Geronimo M., Stanton C., Niaura R., Abrams D., & Taylor K.L. (2021). Predictors of attrition in a smoking cessation trial conducted in the lung cancer screening setting. Contemporary Clinical Trials, 106, 106429. https://doi.org/10.1016/j.cct.2021.106429 | Wrong exposure |
| Kim E.G., Park S.K., Lee Y.-M., Hyun M.Y., & Narapareddy L.R. (2020). Factors associated with maintenance of smoking cessation in adolescents after implementation of tobacco pricing policy in South Korea: Evidence from the 11th Youth Health Behavior Survey. Research in Nursing & Health, 43(1), 40–47. https://doi.org/10.1002/nur.21996 | Wrong study design |
| Kim G.E. & Kim S.-I. (2021). The relationship between smoking and depression in Korean men: A population-based cohort study. Asia-Pacific Psychiatry, 13(SUPPL 1). https://doi.org/10.1111/appy.12462 | Wrong study design |
| Kim, C.-Y., Lee, C. M., Lee, S., Yoo, J. E., Lee, H., Park, H. E., Han, K., & Choi, S.-Y. (2023). The Association of Smoking Status and Clustering of Obesity and Depression on the Risk of Early-Onset Cardiovascular Disease in Young Adults: A Nationwide Cohort Study. *Korean Circulation Journal, 53*(1), 17–30. https://doi.org/10.4070/kcj.2022.0179 | Wrong study design |
| Kim, D., Park, H., & Blackburn, B. (2021). Does childbirth affect paternal smoking? Addiction Research & Theory. https://doi.org/10.1080/16066359.2021.1995369 | Wrong outcome |
| Kim, E. S., Shiba, K., Boehm, J. K., & Kubzansky, L. D. (2020). Sense of purpose in life and five health behaviors in older adults. Preventive Medicine, 139(pm4, 0322116), 106172. https://doi.org/10.1016/j.ypmed.2020.106172 | Wrong study design |
| Kim, G. E., Kim, M.-H., Lim, W.-J., & Kim, S. I. (2022). The effects of smoking habit change on the risk of depression-Analysis of data from the Korean National Health Insurance Service. Journal of Affective Disorders, 302(h3v, 7906073), 293–301. https://doi.org/10.1016/j.jad.2022.01.095 | Wrong outcome |
| Kim, H., & Lee, K.-S. (2021). Association of Stress Management with Success of Smoking Cessation in Korean Female Emotional Labor Workers for Service and Sales. International Journal of Environmental Research and Public Health, 18(6). https://doi.org/10.3390/ijerph18063023 | Wrong outcome |
| Kim, M. K., Han, K., Kim, B., Kim, J., & Kwon, H.-S. (2022). Effects of exercise initiation and smoking cessation after new-onset type 2 diabetes mellitus on risk of mortality and cardiovascular outcomes. Scientific Reports, 12(1), 10656. https://doi.org/10.1038/s41598-022-14603-1 | Wrong study design |
| Kim, N., McCarthy, D. E., Cook, J. W., Piper, M. E., Schlam, T. R., Baker, T. B., & Baker, B. (2021). Time-varying effects of ‘optimized smoking treatment’ on craving, negative affect and anhedonia. Addiction, 116(3), 608–617. https://doi.org/10.1111/add.15232 | Wrong outcome |
| Kim, S. S., Cooley, M. E., Lee, S. A., DeMarco, R. F., & Allen, A. (2020). Prediction of smoking abstinence in women living with human immunodeficiency virus infection. Nursing Research, 69(3), 167–175. https://doi.org/10.1097/NNR.0000000000000421 | Mental health measured at baseline only, no follow up |
| Kinnunen, T., Haukkala, A., Korhonen, T., Quiles, Z. N., Spiro, A., & Garvey, A. J. (2006). Depression and Smoking across 25 Years of the Normative Aging Study. The International Journal of Psychiatry in Medicine, 36(4), 413–426. https://doi.org/10.2190/G652-T403-73H7-2X28 | Did not distinguish between unsuccessful quit attempt and no attempt to quit |
| Kirchner, T. R. (2021). Relapse dynamics during smoking cessation: Recurrent abstinence violation effects and lapse-relapse progression. *Dissertation Abstracts International: Section B: The Sciences and Engineering*, *82*(11-B), No-Specified. | Wrong study design |
| Kiviruusu O., Berg N., Piirtola M., Viertio S., Suvisaari J., Korhonen T., & Marttunen M. (2024). Life-Course Associations Between Smoking and Depressive Symptoms. A 30-Year Finnish Follow-up Study. *Nicotine and Tobacco Research*, *26*(7), 843-851. https://doi.org/10.1093/ntr/ntae012 | Did not distinguish between unsuccessful quit attempt and no attempt to quit |
| Kiviruusu, O., Berg, N., Piirtola, M., Viertio, S., Suvisaari, J., Korhonen, T., & Marttunen, M. (2022). Life course associations between smoking and depressive symptoms. A 30-year Finnish follow-up study. *Nordic Journal of Psychiatry*, 100927567. https://doi.org/10.1080/08039488.2021.2019912 | Wrong exposure |
| Klemperer, E. M., Mermelstein, R., Baker, T. B., Hughes, J. R., Fiore, M. C., Piper, M. E., Schlam, T. R., Jorenby, D. E., Collins, L. M., Cook, J. W., & Al’Absi, B. (2020). Predictors of smoking cessation attempts and success following motivation-phase interventions among people initially unwilling to quit smoking. Nicotine & Tobacco Research, 22(9), 1446–1452. https://doi.org/10.1093/ntr/ntaa051 | Wrong outcome |
| Klinsophon, T., Thaveeratitham, P., & Janwantanakul, P. (2022). The effect of three-part breathing exercise on smoking cessation: A 6-month cluster-randomized clinical trial. Journal of Bodywork and Movement Therapies, 32(9700068), 156–162. https://doi.org/10.1016/j.jbmt.2022.04.015 | Wrong study design |
| Klugman, M., Hosgood, H. D. 3rd, Hua, S., Xue, X., Vu, T.-H. T., Perreira, K. M., Castaneda, S. F., Cai, J., Pike, J. R., Daviglus, M., Kaplan, R. C., & Isasi, C. R. (2020). A longitudinal analysis of nondaily smokers: The Hispanic Community Health Study/Study of Latinos (HCHS/SOL). Annals of Epidemiology, 49(9100013, bx8), 61–67. https://doi.org/10.1016/j.annepidem.2020.06.007 | Wrong exposure |
| Knerich, V., Jones, A. A., Seyedin, S., Siu, C., Dinh, L., Mostafavi, S., Barr, A. M., Panenka, W. J., Thornton, A. E., Honer, W. G., Rutherford, A. R., & Aldridge, B.-A. (2019). Social and structural factors associated with substance use within the support network of adults living in precarious housing in a socially marginalized neighborhood of Vancouver, Canada. PLoS ONE, 14(9). https://doi.org/10.1371/journal.pone.0222611 | Wrong outcome |
| Kollins, S. H., English, J. S., Itchon-Ramos, N., Chrisman, A. K., Dew, R., O’Brien, B., & McClernon, F. (2014). A pilot study of lis-dexamfetamine dimesylate (LDX/SPD489) to facilitate smoking cessation in nicotine-dependent adults with ADHD. Journal of Attention Disorders, 18(2), 158–168. https://doi.org/10.1177/1087054712440320 | Wrong outcome |
| Komiyama, M., Ozaki, Y., Miyazaki, Y., Yasoda, A., Wada, H., Yamakage, H., Satoh-Asahara, N., Morimoto, T., Shimatsu, A., Takahashi, Y., & Hasegawa, K. (2021). Short-term Changes in Self-rating Depression Scale Scores after Smoking Cessation in Neurotic Patients. Internal Medicine (Tokyo, Japan), 60(8), 1175–1181. https://doi.org/10.2169/internalmedicine.4868-20 | Only participants who successfully quit were included |
| Komiyama, M., Ozaki, Y., Wada, H., Yamakage, H., Satoh-Asahara, N., Yasoda, A., Sunagawa, Y., Morimoto, T., Tamaki, S., Masahiro Suzuki, Shibayama, T., Kato, T., Okada, Y., Kita, T., Takahashi, Y., & Hasegawa, K. (2022). Randomized double-blind placebo-controlled multicenter trial for the effects of a polyherbal remedy, Yokukansan (YiganSan), in smokers with depressive tendencies. BMC Complementary Medicine and Therapies, 22(1), 311. https://doi.org/10.1186/s12906-022-03788-7 | Wrong study design |
| Koo, H.-K., Hoth, K. F., Make, B. J., Regan, E. A., Crapo, J. D., Silverman, E. K., & DeMeo, D. L. (2022). Optimism is associated with respiratory symptoms and functional status in chronic obstructive pulmonary disease. Respiratory Research, 23(1), 19. https://doi.org/10.1186/s12931-021-01922-6 | Mental health measured at baseline only, no follow up |
| Korchia, T., Faugere, M., Suc, N., Garosi, A., Andrieu-Haller, C., Breyton, M., Godin, O., Aouizerate, B., Arbus, C., Bennabi, D., Bellivier, F., Bougerol, T., Camus, V., Courtet, P., Doumy, O., El-Hage, W., Genty, J.-B., Haffen, E., Holtzmann, J., … Airagnes, B. (2022). Recommendations of the treatment-resistant depression expert center network for promoting tobacco smoking cessation based on the results from the real-world FACE-TRD national cohort. Progress in Neuro-Psychopharmacology & Biological Psychiatry, 114, 1–10. https://doi.org/10.1016/j.pnpbp.2021.110479 | Mental health measured at baseline only, no follow up |
| Korzh, O., Titkova, A., Fylenko, Y., & Lavrova, Y. (2022). Evaluation of health-promoting self-care behaviors in hypertensive patients with concomitant chronic kidney disease in primary care. Primary Health Care Research & Development, 23(100897390), e48. https://doi.org/10.1017/S1463423622000299 | Wrong outcome |
| Kosterman, R., Epstein, M., Bailey, J. A., Furlong, M., Hawkins, J. D., & Babb, B. (2021). The role of electronic cigarette use for quitting or reducing combustible cigarette use in the 30s: Longitudinal changes and moderated relationships. Drug and Alcohol Dependence, 227. https://doi.org/10.1016/j.drugalcdep.2021.108940 | Wrong outcome |
| Kotlyar, M., Shanley, R., Dufresne, S. R., Corcoran, G. A., & Hatsukami, D. K. (2022). Effect of restricting menthol flavored cigarettes or E-cigarettes on smoking behavior in menthol smokers. Preventive Medicine, 165(Pt B), 107243. https://doi.org/10.1016/j.ypmed.2022.107243 | Wrong exposure |
| Kotoulas S.-C., Stefanidou A., Chatzopoulos E., Fekete-Passa K., Domvri K., Grigoriou I., Argyropoulou-Pataka P., & Pataka A. (2019). Prognostic factors affecting smoking cessation: A real-life study in a population of greek smokers visited a smoking cessation clinic. *Pneumon*, *32*(1–2), 12–22. | Wrong study design |
| Kranzler H.R., Washio Y., Zindel L.R., Lynch K.G., Hand D., Tyndale R.F., Oncken C., & Schnoll R. (2021). Pregnant smokers receiving opioid agonist therapy have an elevated nicotine metabolite ratio: A replication study. Nicotine and Tobacco Research, 22(10), 1923–1927. https://doi.org/10.1093/NTR/NTAA066 | Wrong study design |
| Kranzler, H. R., Washio, Y., Zindel, L. R., Wileyto, E. P., Srinivas, S., Hand, D. J., Hoffman, M., Oncken, C., & Schnoll, R. A. (2021). Placebo-controlled trial of bupropion for smoking cessation in pregnant women. American Journal of Obstetrics & Gynecology MFM, 3(6), 100315. https://doi.org/10.1016/j.ajogmf.2021.100315 | Mental health measured at baseline only, no follow up |
| Krebs, J., Konrad, N., Opitz-Welke, A., & Amato, B. (2020). Addictionmedicine in correctional settings exemplified by the Berlin penal institution. Suchtmedizin Unter Kustodialen Bedingungen Am Beispiel Des Berliner Justizvollzuges., 14(1), 85–94. https://doi.org/10.1007/s11757-019-00573-0 | Wrong study design |
| Kruse G.R., Park E., Chang Y., Haberer J., Abroms L., Shahid N.N., Howard S., Haas J., & Rigotti N.A. (2019). A proactive text messaging intervention with medication adherence support tailored to primary care populations: A pilot randomized trial of getready2quit. Journal of General Internal Medicine, 34(2 Supplement), S114–S115. https://doi.org/10.1007/11606.1525-1497 | Wrong study design |
| Kruse, G. R., Thawal, V., Gupte, H. A., Chaudhuri, L., Pradhan, S., Howard, S., Rigotti, N. A., & Boardman, B. (2020). Tobacco use and subsequent cessation among hospitalized patients in Mumbai, India: A longitudinal study. Nicotine & Tobacco Research, 22(3), 363–370. https://doi.org/10.1093/ntr/ntz026 | Wrong study design |
| Kulhanek, A., Lukavska, K., Gabrhelik, R., Novak, D., Burda, V., Prokop, J., Holter, M. T. S., & Brendryen, H. (2022). Comparing Reminders Sent via SMS Text Messaging and Email for Improving Adherence to an Electronic Health Program: Randomized Controlled Trial. JMIR mHealth and uHealth, 10(3), e31040. https://doi.org/10.2196/31040 | Wrong study design |
| Kunicki, Z. J., Hallgren, M., Uebelacker, L. A., Brown, R. A., Price, L. H., Abrantes, A. M., & Abrantes, A.-S. (2022). Examining the effect of exercise on the relationship between affect and cravings among smokers engaged in cessation treatment. Addictive Behaviors, 125. https://doi.org/10.1016/j.addbeh.2021.107156 | Wrong study design |
| Kuo, C.-W., Lin, C.-F., Chen, C.-Y., Wang, R.-H., Chou, C.-Y., Cheng, H.-J., Wu, J.-S., Chen, C.-W., Shieh, C.-C., & Yu, T. (2022). Body-Weight Gain in Women During Smoking Cessation Is a Sex-Specific Predictor of 6-Month Abstinence: A Retrospective Cohort Study. Frontiers in Public Health, 10(101616579), 872220. https://doi.org/10.3389/fpubh.2022.872220 | Wrong study design |
| Kushnir, V. (2019). Factors influencing the effectiveness of mass distribution of free nicotine replacement therapy. *Dissertation Abstracts International: Section B: The Sciences and Engineering*, *80*(4-B(E)), No-Specified. | Wrong study design |
| Kwon, D. M., Santiago-Torres, M., Mull, K. E., Sullivan, B. M., Bricker, J. B., & Al-Zahrani, A. (2022). Older adults who smoke: Do they engage with and benefit from web-based smoking cessation interventions? Preventive Medicine: An International Journal Devoted to Practice and Theory, 161, 1–7. https://doi.org/10.1016/j.ypmed.2022.107118 | Wrong study design |
| Lal, A., Mohebi, M., White, S. L., Scollo, M., & McCaffrey, N. (2022). Household expenditure of smokers and ex-smokers across socioeconomic groups: Results from a large nationwide Australian longitudinal survey. BMC Public Health, 22(1), 1706. https://doi.org/10.1186/s12889-022-14083-y | Wrong study design |
| Lam, J. O., Levine-Hall, T., Hood, N., Alexeeff, S. E., Horberg, M. A., Young-Wolff, K. C., Sterling, S. A., Williams, A., Weisner, C., Satre, D. D., Silverberg, M. J., & Altekruse, B. (2020). Smoking and cessation treatment among persons with and without HIV in a U.S. Integrated health system. Drug and Alcohol Dependence, 213. https://doi.org/10.1016/j.drugalcdep.2020.108128 | Wrong outcome |
| Landais, L. L., van Wijk, E. C., & Harting, J. (2021). Smoking Cessation in Lower Socioeconomic Groups: Adaptation and Pilot Test of a Rolling Group Intervention. BioMed Research International, 2021(101600173), 8830912. https://doi.org/10.1155/2021/8830912 | Wrong study design |
| Lappan S., Thorne C.B., Long D., & Hendricks P.S. (2020). Longitudinal and reciprocal relationships between psychological well-being and smoking. Nicotine and Tobacco Research, 22(1), 18–23. https://doi.org/10.1093/ntr/nty185 | Wrong outcome |
| Lappin, J. M., Thomas, D., Curtis, J., Blowfield, S., Gatsi, M., Marr, G., & Courtney, R. (2020). Targeted Intervention to Reduce Smoking among People with Severe Mental Illness: Implementation of a Smoking Cessation Intervention in an Inpatient Mental Health Setting. Medicina, 56(4). https://doi.org/10.3390/medicina56040204 | Wrong outcome |
| Larsen, A. S. F., Reiersen, A. T., Nadland, I. H., Wesche, J., & Aboyans, de A. (2020). Self-reported health status and disease-specific quality of life one year after treatment for peripheral arterial disease in clinical practice. Health and Quality of Life Outcomes, 18. https://doi.org/10.1186/s12955-020-01477-y | Wrong study design |
| Laude JR, Bailey SR, Crew E, Varady A, Lembke A, McFall D, Jeon A, Killen D, Killen JD, & David SP. (2017). Extended treatment for cigarette smoking cessation: A randomized control trial. Addiction, 112(8), 1451–1459. https://doi.org/10.1111/add.13806 | Did not analyse mental health outcomes by exposure of interest |
| Lauridsen S.V., Thomsen T., Jensen J.B., Kallemose T., Schmidt Behrend M., Steffensen K., Poulsen A.M., Jacobsen A., Walther L., Isaksson A., Thind P., & Tonnesen H. (2022). Effect of a Smoking and Alcohol Cessation Intervention Initiated Shortly Before Radical Cystectomy-the STOP-OP Study: A Randomised Clinical Trial. European Urology Focus, 8(6), 1650–1658. https://doi.org/10.1016/j.euf.2022.02.005 | Wrong study design |
| Le Faou A.-L., Allagbe I., Airagnes G., Baha M., Boussadi A., & Limosin F. (2020). Predictors of Smoking Cessation Attempt and Continued Abstinence among Low-Income Disabled Smokers: Evidence from the French National Smoking Cessation Cohort CDT-Net. Substance Use & Misuse, 55(10), 1724–1731. https://doi.org/10.1080/10826084.2020.1759644 | Wrong study design |
| Le Mao R., Tromeur C., Paleiron N., Sanchez O., Gagnadoux F., Jouneau S., Magnan A., Hayem-Vannimenus C., Dansou A., Proust A., Dion A., Larhantec G., Brestec A.L., Dewitte J.-D., Roche N., Leroyer C., & Couturaud F. (2020). Effect of Early Initiation of Varenicline on Smoking Cessation in COPD Patients Admitted for Exacerbation: The Save Randomized Clinical Trial. COPD: Journal of Chronic Obstructive Pulmonary Disease, 17(1), 7–14. https://doi.org/10.1080/15412555.2019.1703928 | Wrong study design |
| Le, T. T. T., & Jaffri, M. A. (2022). The association between smoking behaviors and prices and taxes per cigarette pack in the United States from 2000 through 2019. BMC Public Health, 22(1), 856. https://doi.org/10.1186/s12889-022-13242-5 | Wrong study design |
| Lee J., Cheong J., Markham M.J., Lam J., Warren G.W., & Salloum R.G. (2021). Negative affect and the utilization of tobacco treatment among adult smokers with cancer. Psycho-Oncology, 30(1), 93–102. https://doi.org/10.1002/pon.5543 | Wrong study design |
| Lee S., Goodall C., Egbert N., & Chung D. (2021). The Moderating Role of Self-construal in Culturally Reflected Fear Appeals. Journal of Health Communication, 26(2), 65–75. https://doi.org/10.1080/10810730.2021.1878309 | Wrong outcome |
| Lee Westmaas, J., Chantaprasopsuk, S., Bontemps-Jones, J., Stephens, R. L., Thorne, C., & Abroms, L. C. (2022). Longitudinal analysis of peer social support and quitting Smoking: Moderation by sex and implications for cessation interventions. Preventive Medicine Reports, 30(101643766), 102059. https://doi.org/10.1016/j.pmedr.2022.102059 | Mental health measured at baseline only, no follow up |
| Lee, A., Lee, K.-S., Lee, D., Ahn, H., Lee, H.-K., Kim, H., Lee, J., & Seo, H.-G. (2021). The Utilization of National Tobacco Cessation Services among Female Smokers and the Need for a Gender-Responsive Approach. International Journal of Environmental Research and Public Health, 18(10). https://doi.org/10.3390/ijerph18105313 | Mental health measured at baseline only, no follow up |
| Lee, B., & Seo, D.-C. (2021). Effects of an 80% cigarette price increase on quit attempts, successful quitting and smoking intensity among Korean adult smokers: Results from nationally representative longitudinal panel data. Tobacco Control, 30(3), 336–343. https://doi.org/10.1136/tobaccocontrol-2019-055518 | Wrong outcome |
| Lee, C., Harari, L., & Park, S. (2020). Early-Life Adversities and Recalcitrant Smoking in Midlife: An Examination of Gender and Life-Course Pathways. Annals of Behavioral Medicine : A Publication of the Society of Behavioral Medicine, 54(11), 867–879. https://doi.org/10.1093/abm/kaaa023 | Wrong study design |
| Lee, D., Lee, K.-S., Lee, A., Ahn, H., Lee, H.-K., Kim, H., Lee, J., & Seo, H.-G. (2021). Successful Smoking Cessation among Women Smokers Based on Utilizing National Smoking Cessation Service Type in Korea. International Journal of Environmental Research and Public Health, 18(12). https://doi.org/10.3390/ijerph18126578 | Wrong outcome |
| Lee, E. J. (2020a). Factors Predicting 6-Month Smoking Cessation in Korean Adults. The Psychiatric Quarterly, 91(3), 703–714. https://doi.org/10.1007/s11126-020-09730-9 | Wrong study design |
| Lee, M., Bastian, L. A., LaRowe, L., DeRycke, E. C., Relyea, M., Becker, W. C., & Ditre, J. W. (2022). Perceived Pain and Smoking Interrelations Among Veterans with Chronic Pain Enrolled in a Smoking Cessation Trial. Pain Medicine (Malden, Mass.), 23(11), 1820–1827. https://doi.org/10.1093/pm/pnac082 | Mental health measured at baseline only, no follow up |
| Lee, M., Miller, S. M., Wen, K.-Y., Hui, S. A., Roussi, P., & Hernandez, E. (2015). Cognitive-behavioral intervention to promote smoking cessation for pregnant and postpartum inner city women. Journal of Behavioral Medicine, 38(6), 932–943. https://doi.org/10.1007/s10865-015-9669-7 | Wrong outcome |
| Lee, S. M., Chun, S., & Lee, J. S. (2020). The Role of Negative Emotions Pre- and Post-Implementation of Graphic Health Warnings: Longitudinal Evidence from South Korea. International Journal of Environmental Research and Public Health, 17(15). https://doi.org/10.3390/ijerph17155393 | Wrong study design |
| Leonelli B.R., Kuhn T., Neifert H.Y., Josephson R., & Hughes J.W. (2020). Behavioral activation for depression in cardiovascular rehabilitation: A case study. Journal of Cardiopulmonary Rehabilitation and Prevention, 40(5), E41. https://doi.org/10.1097/HCR.0000000000000556 | Wrong study design |
| Lepore, S. J., Collins, B. N., Sosnowski, D. W., & Andresen, B. (2019). Self-efficacy as a pathway to long-term smoking cessation among low-income parents in the multilevel Kids Safe and Smokefree intervention. Drug and Alcohol Dependence, 204. https://doi.org/10.1016/j.drugalcdep.2019.05.027 | Wrong study design |
| Leppanen, A. (2021). Tobacco cessation on prescription: A primary healthcare intervention targeting socioeconomically disadvantaged areas in Stockholm. *Dissertation Abstracts International: Section B: The Sciences and Engineering*, *82*(9-B), No-Specified. | Wrong study design |
| Leszko, M., Keenan-Devlin, L., Adam, E. K., Buss, C., Grobman, W., Simhan, H., Wadhwa, P., Mroczek, D. K., & Borders, A. (2020). Are personality traits associated with smoking and alcohol use prior to and during pregnancy?. PloS One, 15(5), e0232668. https://doi.org/10.1371/journal.pone.0232668 | Wrong exposure |
| Leung L, Neufeld T, & Marin S. (2012). Effect of self-administered auricular acupressure on smoking cessation—A pilot study. BMC Complementary and Alternative Medicine, 12, 11. https://doi.org/10.1186/1472-6882-12-11 | Wrong outcome |
| Leutwyler, H., & Hubbard, E. (2021). Telephone based smoking cessation intervention for adults with serious mental illness during the COVID-19 pandemic. Tobacco Use Insights, 14(101608659), 1179173X211065989. https://doi.org/10.1177/1179173X211065989 | Wrong study design |
| Leutwyler, H., Hubbard, E., Bussell, T., Balestra, D., Cooper, B., Souza, R. B., & Humfleet, G. (2024). A Pilot Randomized Controlled Trial of a Multicomponent Smoking Cessation Intervention for Adults with Serious Mental Illness. *Games for Health Journal*. https://doi.org/10.1089/g4h.2023.0160 | Did not analyse mental health outcomes by exposure of interest |
| Levine, M. D., Cheng, Y., Marcus, M. D., Kalarchian, M. A., & Emery, R. L. (2016). Preventing postpartum smoking relapse: A randomized clinical trial. JAMA Internal Medicine, 176(4), 443–452. https://doi.org/10.1001/jamainternmed.2016.0248 | Mental health not measured before quit attempt |
| Levine, M. D., Emery, R. L., Kolko Conlon, R. P., Marcus, M. D., Germeroth, L. J., Salk, R. H., Cheng, Y., & Allen, B. (2020). Depressive symptoms assessed near the end of pregnancy predict differential response to postpartum smoking relapse prevention intervention. Annals of Behavioral Medicine, 54(2), 119–124. https://doi.org/10.1093/abm/kaz026 | Wrong study design |
| Levy D. (2020). Vulnerable populations, disparities, and the u.s. Tobacco endgame. Circulation, 141(SUPPL 1). https://doi.org/10.1161/circ.141.suppl-1.57 | Wrong study design |
| Levy, D. T., Yuan, Z., Li, Y., Alberg, A. J., Cummings, K. M., & Abrams, B. (2019). A modeling approach to gauging the effects of nicotine vaping product use on cessation from cigarettes: What do we know, what do we need to know? Addiction, 114(Suppl 1), 86–96. https://doi.org/10.1111/add.14530 | Wrong outcome |
| Lewis, E. M., Jeffries, E. R., Zvolensky, M. J., Buckner, J. D., & Aiken, A. (2020). Anxiety sensitivity among smokers during a reduction attempt: The impact of hatha yoga. Cognitive Therapy and Research, 44(3), 709–714. https://doi.org/10.1007/s10608-020-10087-3 | Wrong outcome |
| Li WHC, Chan SSC, Wang KMP, & Lam TH. (2015). Helping cancer patients quit smoking by increasing their risk perception: A study protocol of a cluster randomized controlled trial. BMC Cancer, 15(1), 490. https://doi.org/10.1186/s12885-015-1496-2 | Wrong outcome |
| Li, J., Fairhurst, C., Peckham, E., Bailey, D., Arundel, C., Hewitt, C., Heron, P., Crosland, S., Parrott, S., Gilbody, S., SCIMITAR+ collaborative, & Bradshaw, B. (2020). Cost-effectiveness of a specialist smoking cessation package compared with standard smoking cessation services for people with severe mental illness in England: A trial-based economic evaluation from the SCIMITAR+ study. Addiction, 115(11), 2113–2122. https://doi.org/10.1111/add.15086 | Wrong study design |
| Li, J., Hajek, P., Pesola, F., Wu, Q., Phillips-Waller, A., Przulj, D., Myers Smith, K., Bisal, N., Sasieni, P., Dawkins, L., Ross, L., Goniewicz, M. L., McRobbie, H., Parrott, S., & Athanasakis, B. (2020). Cost-effectiveness of e-cigarettes compared with nicotine replacement therapy in stop smoking services in England (TEC study): A randomized controlled trial. Addiction, 115(3), 507–517. https://doi.org/10.1111/add.14829 | Wrong outcome |
| Li, J., Parrott, S., Keding, A., Dogar, O., Gabe, R., Marshall, A.-M., Huque, R., Barua, D., Fatima, R., Khan, A., Zahid, R., Mansoor, S., Kotz, D., Boeckmann, M., Elsey, H., Kralikova, E., Readshaw, A., Sheikh, A., Siddiqi, K., & TB & Tobacco Consortium. (2022). Cost-utility of cytisine for smoking cessation over and above behavioural support in people with newly diagnosed pulmonary tuberculosis: An economic evaluation of a multicentre randomised controlled trial. BMJ Open, 12(8), e049644. https://doi.org/10.1136/bmjopen-2021-049644 | Wrong study design |
| Li, L., Borland, R., Cummings, K. M., McNeill, A., Heckman, B. W., Fong, G. T., O’Connor, R. J., & Driezen, P. (2020). Are health conditions and concerns about health effects of smoking predictive of quitting? Findings from the ITC 4CV Survey ( 2016-2018 ). Tobacco Prevention & Cessation, 6(101693412), 60. https://doi.org/10.18332/tpc/127471 | Wrong exposure |
| Li, L., Borland, R., O’Connor, R. J., Fong, G. T., McNeill, A., Driezen, P., & Cummings, K. M. (2019). How Are Self-Reported Physical and Mental Health Conditions Related to Vaping Activities among Smokers and Quitters: Findings from the ITC Four Country Smoking and Vaping Wave 1 Survey. International Journal of Environmental Research and Public Health, 16(8). https://doi.org/10.3390/ijerph16081412 | Wrong exposure |
| Li, L., Borland, R., Yong, H.-H., Gravely, S., Fong, G. T., Cummings, K. M., East, K., & Le Grande, M. (2022). Experienced Effects on Well-Being following Smoking Cessation: Findings from the 2020 ITC Four Country Smoking and Vaping Survey. International Journal of Environmental Research and Public Health, 19(16). https://doi.org/10.3390/ijerph191610037 | Wrong study design |
| Li, L., Lee, J.-H., Sutton, S. K., Simmons, V. N., & Brandon, T. H. (2020). A Bayesian transition model for missing longitudinal binary outcomes and an application to a smoking cessation study. Statistical Modelling, 20(3), 310–338. https://doi.org/10.1177/1471082x18821489 | Wrong outcome |
| Li, M., Okamoto, R., & Shirai, F. (2019). Factors associated with smoking cessation and relapse in the Japanese smoking cessation treatment program: A prospective cohort study based on financial support in Suita City, Japan. Tobacco Induced Diseases, 17(101201591), 71. https://doi.org/10.18332/tid/112154 | Wrong outcome |
| Li, W. H. C., Ho, K. Y., Wang, M. P., Cheung, D. Y. T., Lam, K. K. W., Xia, W., Cheung, K. Y., Wong, C. K. H., Chan, S. S. C., & Lam, T. H. (2020). Effectiveness of a Brief Self-determination Theory-Based Smoking Cessation Intervention for Smokers at Emergency Departments in Hong Kong: A Randomized Clinical Trial. JAMA Internal Medicine, 180(2), 206–214. https://doi.org/10.1001/jamainternmed.2019.5176 | Wrong study design |
| Liang, M., Koslovsky, M. D., Hebert, E. T., Kendzor, D. E., Businelle, M. S., Vannucci, M., & Bandalos, B. (2021). Bayesian continuous-time hidden Markov models with covariate selection for intensive longitudinal data with measurement error. Psychological Methods. https://doi.org/10.1037/met0000433 | Wrong exposure |
| Liao, Y., & Tang, J. (2021). Efficacy of cognitive behavioural therapy-based smartphone app for smoking cessation in China: A study protocol of a randomised controlled trial. BMJ Open, 11(1), e041985. https://doi.org/10.1136/bmjopen-2020-041985 | Wrong study design |
| Lien, L., Bolstad, I., Bramness, J. G., & Al-Rubaye, B. (2021). Smoking among inpatients in treatment for substance use disorders: Prevalence and effect on mental health and quality of life. *BMC Psychiatry, 21*. https://doi.org/10.1186/s12888-021-03252-9 | Wrong outcome |
| Lima, D. R., Carvalho, C. F. C., Guimaraes-Pereira, B. B. S., Loreto, A. R., Frallonardo, F. P., Ismael, F., de Andrade, A. G., Castaldelli-Maia, J. M., & Benowitz, C. (2020). Abstinence and retention outcomes in a smoking cessation program among individuals with co-morbid substance use and mental disorders. Journal of Psychiatric Research, 125, 121–128. https://doi.org/10.1016/j.jpsychires.2020.03.014 | Wrong outcome |
| Lima, D. R., Guimaraes-Pereira, B. B. S., Mannes, Z. L., Carvalho, C. F. C., Loreto, A. R., Davanso, L. C., Frallonardo, F. P., Ismael, F., de Andrade, A. G., & Castaldelli-Maia, J. M. (2022). The effect of a real-world intervention for smoking cessation in Adults with and without comorbid psychiatric and substance use disorders: A one-year follow-up study. Psychiatry Research, 315(qc4, 7911385), 114722. https://doi.org/10.1016/j.psychres.2022.114722 | Wrong study design |
| Lin, H., Lin, Y., Xiao, L., Chen, Y., Zeng, X., & Chang, C. (2021). How Do Smoking Status and Smoking Cessation Efforts Affect TB Recurrence After Successful Completion of Anti-TB Treatment? A Multicenter, Prospective Cohort Study With a 7-Year Follow-up in China. Nicotine & Tobacco Research : Official Journal of the Society for Research on Nicotine and Tobacco, 23(12), 1995–2002. https://doi.org/10.1093/ntr/ntab117 | Did not distinguish between unsuccessful quit attempt and no attempt to quit |
| Lin, W., Hobkirk, A. L., Zhu, J., Krebs, N. M., Hayes, J. E., Richie, J. P. Jr., Liao, J., Horn, K., Foulds, J., Muscat, J. E., & Ahijevych, A. (2022). Effect of menthol on nicotine reduction: Pooled results from two double-blind randomized controlled trials. Brain Research Bulletin, 189, 131–138. https://doi.org/10.1016/j.brainresbull.2022.08.019 | Wrong study design |
| Lin, Y., Tudor-Sfetea, C., Siddiqui, S., Sherwani, Y., Ahmed, M., & Eisingerich, A. B. (2018). Effective behavioral changes through a digital mHealth app: Exploring the impact of hedonic well-being, psychological empowerment and inspiration. *JMIR MHealth and UHealth*, *6*(6), e10024. | Wrong outcome |
| Linn, B. K., Stasiewicz, P. R., Fillo, J., Bradizza, C. M., & Abrams, A. (2020). The great disrupter: Relationship of alexithymia to emotion regulation processes and smoking among pregnant women. Substance Use & Misuse, 55(7), 1113–1121. https://doi.org/10.1080/10826084.2020.1729198 | Wrong study design |
| Listabarth, S., Groemer, M., Waldhoer, T., Vyssoki, B., Pruckner, N., Vyssoki, S., Glahn, A., Konig-Castillo, D. M., & Konig, D. (2022). Cognitive decline and alcohol consumption in the aging population-A longitudinal analysis of the Survey of Health, Ageing and Retirement in Europe. European Psychiatry : The Journal of the Association of European Psychiatrists, 65(1), e83. https://doi.org/10.1192/j.eurpsy.2022.2344 | Wrong study design |
| Littlewood Rae A, Claus Eric D, Wilcox Claire E, Mickey Jessica, Arenella Pamela B, Bryan Angela D, & Hutchison Kent E. (2017). Moderators of smoking cessation outcomes in a randomized-controlled trial of varenicline versus placebo. Psychopharmacology, 234(23–24), 3417–3429. https://doi.org/10.1007/s00213-017-4721-7 | Did not analyse mental health outcomes by exposure of interest |
| Liu, M. E., Tsai, S. J., Jeang, S. Y., Peng, S. L., Wu, S. L., Chen, M. C., Tsai, Y. L., & Yang, S. T. (2011). Varenicline prevents affective and cognitive exacerbation during smoking abstinence in male patients with schizophrenia. *Psychiatry Research*, *190*(1), 79–84. | Did not analyse mental health outcomes by exposure of interest |
| Liu, N. H., Wu, C., Perez-Stable, E. J., Munoz, R. F., & Alegria, A. (2021). Longitudinal association between smoking abstinence and depression severity in those with baseline current, past, and no history of major depressive episode in an international online tobacco cessation study. Nicotine & Tobacco Research, 23(2), 267–275. https://doi.org/10.1093/ntr/ntaa036 | Wrong exposure |
| Liu, S., Jiang, H., Zhang, D., Luo, J., & Zhang, H. (2022). The Association between Smoking Cessation and Depressive Symptoms: Diet Quality Plays a Mediating Role. Nutrients, 14(15). https://doi.org/10.3390/nu14153047 | Wrong exposure |
| Liu, Y., Greenlund, K. J., VanFrank, B., Xu, F., Lu, H., Croft, J. B., & Anthonisen, A. (2022). Smoking cessation among U.S. adult smokers with and without chronic obstructive pulmonary disease, 2018. American Journal of Preventive Medicine, 62(4), 492–502. https://doi.org/10.1016/j.amepre.2021.12.001 | Mental health measured at baseline only, no follow up |
| Lodge, S., Bartlem, K., Gibson, L., Fehily, C., Bradley, T., McKeon, E., Reakes, K., Rickards, S., Hastings, P., & Bowman, J. (2022). Characteristics and service use of NSW Quitline callers with and without mental health conditions. Frontiers in Psychiatry, 13(101545006), 868084. https://doi.org/10.3389/fpsyt.2022.868084 | Did not analyse mental health outcomes by exposure of interest |
| Lombardero Anayansi. (2017). Smoking cessation for patients in multidisciplinary pain treatment settings: A preliminary test of acceptance and commitment therapy. *Dissertation Abstracts International*, *77*(11-B(E)), No-Pagination Specified. | Did not analyse mental health outcomes by exposure of interest |
| Lopez-Lazcano A.I., Gual A., Colmenero J., Caballeria E., Lligona A., Navasa M., Crespo G., Lopez E., & Lopez-Pelayo H. (2020). Active smoking before liver transplantation in patients with alcohol use disorder: Risk factors and outcomes. Journal of Clinical Medicine, 9(9), 1–10. https://doi.org/10.3390/jcm9092710 | Wrong study design |
| Lotfalian, S., Spears, C. A., & Juliano, L. M. (2020). The effects of mindfulness-based yogic breathing on craving, affect, and smoking behavior. Psychology of Addictive Behaviors : Journal of the Society of Psychologists in Addictive Behaviors, 34(2), 351–359. https://doi.org/10.1037/adb0000536 | Wrong exposure |
| Loth, F. G., Zeschke, M., Bickhardt, J., Heindl, T., & Muhlig, S. (2019). Tobacco dependence, smoking behavior and depressiveness among COPD patients: Partial results of a naturalistic study on a disease-specific smoking cessation in pulmonary practices (ATEMM-Study). Sucht: Zeitschrift Fur Wissenschaft Und Praxis, 65(1), 23–33. https://doi.org/10.1024/0939-5911/a000577 | Mental health measured at baseline only, no follow up |
| Lou P, Chen P, Zhang P, Yu J, Wang Y, Chen N, Zhang L, Wu H, & Zhao J. (2015). A COPD health management program in a community-based primary care setting: A randomized controlled trial. Respiratory Care, 60(1), 102–112. https://doi.org/10.4187/respcare.03420 | Wrong exposure |
| Loukas, A., Marti, C. N., & Harrell, M. B. (2022). Electronic nicotine delivery systems use predicts transitions in cigarette smoking among young adults. Drug and Alcohol Dependence, 231(ebs, 7513587), 109251. https://doi.org/10.1016/j.drugalcdep.2021.109251 | Wrong study design |
| Louwagie G., Kanaan M., Morojele N.K., Van Zyl A., Moriarty A.S., Li J., Siddiqi K., Turner A., Mdege N.D., Omole O.B., Tumbo J., Bachmann M., Parrott S., & Ayo-Yusuf O.A. (2022). Effect of a brief motivational interview and text message intervention targeting tobacco smoking, alcohol use and medication adherence to improve tuberculosis treatment outcomes in adult patients with tuberculosis: A multicentre, randomised controlled trial of the ProLife programme in South Africa. BMJ Open, 12(2), e056496. https://doi.org/10.1136/bmjopen-2021-056496 | Wrong study design |
| Lubitz S.F., Flitter A., Wileyto E.P., Ziedonis D., Stevens N., Leone F., Mandell D., Kimberly J., Beidas R., & Schnoll R.A. (2020). History and correlates of smoking cessation behaviors among smokers with serious mental illness. Nicotine and Tobacco Research, 22(9), 1492–1499. https://doi.org/10.1093/ntr/ntz229 | Wrong study design |
| Lucchiari, C., Masiero, M., Mazzocco, K., Veronesi, G., Maisonneuve, P., Jemos, C., Sale, E. O., Spina, S., Bertolotti, R., Pravettoni, G., & Berkhof, B. (2020). Benefits of e-cigarettes in smoking reduction and in pulmonary health among chronic smokers undergoing a lung cancer screening program at 6 months. Addictive Behaviors, 103. https://doi.org/10.1016/j.addbeh.2019.106222 | Mental health measured at baseline only, no follow up |
| Ludman, E. J., McBride, C. M., Nelson, J. C., Curry, S. J., Grothaus, L. C., Lando, H. A., & Pirie, P. L. (2000). Stress, depressive symptoms, and smoking cessation among pregnant women. Health Psychol, 19(1), 21–27. https://doi.org/10.1037//0278-6133.19.1.21 | Mental health measured at baseline only, no follow up |
| Ma, Y., Chen, Y., Zhang, N., Xu, G., Wang, Y., Sun, Y., Bai, C., & Zuo, Z. (2022). Efficacy and safety of pulmonary rehabilitation training on lung function, quality of life, and T cell immune function in patients with stable chronic obstructive pulmonary disease: A randomized controlled trial. Annals of Palliative Medicine, 11(5), 1774–1785. https://doi.org/10.21037/apm-22-451 | Wrong study design |
| Machado, R. C. B. R., Vargas, H. O., Baracat, M. M., Urbano, M. R., Verri, W. A. Jr., Porcu, M., Nunes, S. O. V., & Aldaham, A. (2020). N-acetylcysteine as an adjunctive treatment for smoking cessation: A randomized clinical trial. Brazilian Journal of Psychiatry, 42(5), 519–526. https://doi.org/10.1590/1516-4446-2019-0753 | Did not analyse mental health outcomes by exposure of interest |
| Machin M., Hayward L., Harris L., Gadhvi V., & Thapar A. (2021). Feasibility of a remotely supervised exercise programme. British Journal of Surgery, 108(SUPPL 7), vii130. https://doi.org/10.1093/bjs/znab308.026 | Wrong study design |
| Machulska, A., Rinck, M., Klucken, T., Kleinke, K., Wunder, J.-C., Remeniuk, O., Margraf, J., & Adams, A. (2022). ‘Push it!’ or ‘hold it!’? A comparison of nicotine-avoidance training and nicotine-inhibition training in smokers motivated to quit. Psychopharmacology, 239(1), 105–121. https://doi.org/10.1007/s00213-021-06058-5 | Did not analyse mental health outcomes by exposure of interest |
| Mackay, D., Mollard, R. C., Granger, M., Bruce, S., Blewett, H., Carlberg, J., Duhamel, T., Eck, P., Faucher, P., Hamm, N. C., Khafipour, E., Lix, L., McMillan, D., Myrie, S., Ravandi, A., Tangri, N., Azad, M., & Jones, P. J. (2019). The Manitoba Personalized Lifestyle Research (TMPLR) study protocol: A multicentre bidirectional observational cohort study with administrative health record linkage investigating the interactions between lifestyle and health in Manitoba, Canada. BMJ Open, 9(10), e023318. https://doi.org/10.1136/bmjopen-2018-023318 | Wrong study design |
| Magee, W., & Clarke, P. (2021). The effect of smoking on depressive symptoms. Addictive Behaviors, 112(2gw, 7603486), 106641. https://doi.org/10.1016/j.addbeh.2020.106641 | Did not distinguish between unsuccessful quit attempt and no attempt to quit |
| Mak, Y.-W., Loke, A.-Y., & Leung, D. Y. P. (2021). Acceptance and Commitment Therapy versus Social Support for Smoking Cessation for People with Schizophrenia: A Randomised Controlled Trial. Journal of Clinical Medicine, 10(19). https://doi.org/10.3390/jcm10194304 | Wrong study design |
| Malburg, C. M., Fucinari, J., Ruterbusch, J. J., Ledgerwood, D. M., Beebe-Dimmer, J. L., Schwartz, A. G., & Cote, M. L. (2020). Continued smoking in African American cancer survivors: The Detroit Research on Cancer Survivors Cohort. Cancer Medicine, 9(20), 7763–7771. https://doi.org/10.1002/cam4.3368 | Wrong study design |
| Marin, D. B., Karol, A. B., Sharma, V., Wetmore, J., Costello, Z., Henry, B., Robinson, M., Thompson, L., Pena, I., & Jandorf, L. (2022). M.I.C.A.H. Project HEAL: Sustainability of a Faith-Based Community Health Advisor Training Program in Urban Underserved Communities in the USA. Journal of Religion and Health, 61(3), 2527–2538. https://doi.org/10.1007/s10943-021-01453-w | Wrong study design |
| Marqueta, A., Jiménez-Muro, A., Beamonte, A., Gargallo, P., & Nerín, I. (2010). Evolución de la ansiedad en el proceso de dejar de fumar en fumadores que acuden a una Unidad de Tabaquismo. Adicciones, 22(4), 317–324. https://doi.org/10.20882/adicciones.173 | Did not analyse mental health outcomes by exposure of interest |
| Martinez, C., Feliu, A., Saura, J., Nieva, G., Pinet, C., Raich, A., Mondon, S., Barrio, P., Andreu, M., Hernandez-Ribas, R., Costa, S., Suelves, J. M., Vilaplana, J., Enriquez, M., Alaustre, L., Vilalta, E., Alvarez, J. B., Guydish, J., Fernandez, E., & Ballbe, M. (2024). Effectiveness of a post-discharge phone-based smoking cessation intervention for patients with severe mental health disorders: The 061 quitmental randomized controlled clinical trial. *International Journal of Mental Health and Addiction*. https://doi.org/10.1007/s11469-024-01254-8 | Wrong outcome |
| Martinez, C., Feliu, A., Torres, N., Nieva, G., Pinet, C., Raich, A., Mondon, S., Barrio, P., Andreu, M., Hernandez-Ribas, R., Vicens, J., Costa, S., Suelves, J. M., Vilaplana, J., Enriquez, M., Alaustre, L., Vilalta, E., Subira, S., Bruguera, E., …, Zwarenstein. (2022). Acceptability and participation predictors for a pragmatic randomized controlled trial to test a smoking cessation intervention after discharge from mental health wards. *Drug and Alcohol Dependence, 234,* 1–6. https://doi.org/10.1016/j.drugalcdep.2022.109390 | Wrong study design |
| Martinez, U., Brandon, K. O., Sutton, S. K., Brandon, T. H., Simmons, V. N., & Arbuckle, B. (2019). Does smoking abstinence predict cancer patients’ quality of life over time? Psycho-Oncology, 28(8), 1702–1711. https://doi.org/10.1002/pon.5145 | Follow up < 6 weeks |
| Martinez, U., Martinez-Loredo, V., Simmons, V. N., Meltzer, L. R., Drobes, D. J., Brandon, K. O., Palmer, A. M., Eissenberg, T., Bullen, C. R., Harrell, P. T., Brandon, T. H., & Abrams, B. (2020). How does smoking and nicotine dependence change after onset of vaping? A retrospective analysis of dual users. Nicotine & Tobacco Research, 22(5), 764–770. https://doi.org/10.1093/ntr/ntz043 | Wrong outcome |
| Martinez, U., Simmons, V. N., Brandon, K. O., Quinn, G. P., Brandon, T. H., & Al Rifai, B. (2022). Examining smoking and vaping behaviors, expectancies, and cessation outcomes between bisexual and heterosexual individuals. Behavioral Medicine. https://doi.org/10.1080/08964289.2022.2077295 | Wrong outcome |
| Martinez, U., Simmons, V. N., Sutton, S. K., Drobes, D. J., Meltzer, L. R., Brandon, K. O., Byrne, M. M., Harrell, P. T., Eissenberg, T., Bullen, C. R., & Brandon, T. H. (2021). Targeted smoking cessation for dual users of combustible and electronic cigarettes: A randomised controlled trial. *The Lancet Public Health, 6*(7), e500–e509. https://doi.org/10.1016/S2468-2667%2820%2930307-8 | Wrong outcome |
| Martinez-Gonzalez, C., Casanova, C., de-Torres, J. P., Marin, J. M., de Lucas, P., Fuster, A., Cosio, B. G., Calle, M., Peces-Barba, G., Solanes, I., Aguero, R., Feu-Collado, N., Alfageme, I., Romero Plaza, A., Balcells, E., de Diego, A., Marin Royo, M., Moreno, A., Llunell Casanovas, A., … Lopez-Campos, J. L. (2018). Changes and Clinical Consequences of Smoking Cessation in Patients With COPD: A Prospective Analysis From the CHAIN Cohort. *Chest, 154*(2), 274–285. https://doi.org/10.1016/j.chest.2018.02.007 | Did not distinguish between unsuccessful quit attempt and no attempt to quit |
| Martinez-Vispo, C., Lopez-Duran, A., Rodriguez-Cano, R., Fernandez del Rio, E., Senra, C., Becona, E., & Audrain-McGovern, B. (2019). Effect of depressive symptoms and sex on the relationship between loneliness and cigarette dependence: A moderated mediation. The Journal of Psychology: Interdisciplinary and Applied, 153(7), 701–713. https://doi.org/10.1080/00223980.2019.1598929 | Wrong study design |
| Martinez-Vispo, C., Lopez-Duran, A., Rodriguez-Cano, R., Senra, C., Becona, E., & Assayag, B. (2021). Treatment completion and anxiety sensitivity effects on smoking cessation outcomes. Addictive Behaviors, 117. https://doi.org/10.1016/j.addbeh.2021.106856 | Wrong exposure |
| Martinez-Vispo, C., Lopez-Duran, A., Senra, C., & Becona, E. (2020). Specific Relapse Predictors: Could Cognitive-Behavioral Treatment for Smoking Cessation Be Improved?. International Journal of Environmental Research and Public Health, 17(12). https://doi.org/10.3390/ijerph17124317 | Only participants who successfully quit were included |
| Martinez-Vispo, C., Lopez-Duran, A., Senra, C., Becona, E., & Aldao, A. (2022). Brooding rumination and anxiety sensitivity: Associations with depressive and anxiety symptoms in treatment-seeking smokers. *Psicothema*, *34*(1), 49–55. | Mental health measured at baseline only, no follow up |
| Martinez-Vispo, C., Lopez-Duran, A., Senra, C., Rodriguez-Cano, R., del Rio, E. F., Becona, E., & Aldao, A. (2020). Environmental reward and depressive symptoms in the relationship between avoidance and cigarette dependence in treatment-seeking smokers. *Psicothema*, *32*(2), 176–181. | Wrong study design |
| Martinez-Vispo, C., Lopez-Duran, A., Senra, C., Rodriguez-Cano, R., Fernandez del Rio, E., & Becona, E. (2020). Behavioral activation and smoking cessation outcomes: The role of depressive symptoms. Addictive Behaviors, 102. https://doi.org/10.1016/j.addbeh.2019.106183 | Mental health measured at baseline only, no follow up |
| Martinez-Vispo, C., Senra, C., Lopez-Duran, A., Fernandez del Rio, E., Becona, E., & Adrian, B. (2020). Does rumination mediate the effect of depressive symptoms on cigarette dependence and craving in seeking treatment smokers? Journal of Psychopathology and Behavioral Assessment, 42(4), 765–773. https://doi.org/10.1007/s10862-020-09812-9 | Did not distinguish between unsuccessful quit attempt and no attempt to quit |
| Martinez-Vispo, C., Senra, C., Lopez-Duran, A., Fernandez del Rio, E., Becona, E., & Armento, B. (2019). Boredom susceptibility as predictor of smoking cessation outcomes: Sex differences. Personality and Individual Differences, 146, 130–135. https://doi.org/10.1016/j.paid.2019.03.026 | Wrong outcome |
| Masalin, S., Kautiainen, H., Gissler, M., Pennanen, P., Eriksson, J. G., & Laine, M. K. (2020). Impact of smoking on gestational diabetes mellitus and offspring birthweight in primiparous women. Acta Obstetricia et Gynecologica Scandinavica, 99(12), 1632–1639. https://doi.org/10.1111/aogs.13924 | Wrong study design |
| Masithah D., Soedirham O., & Triyoga R.S. (2019). The influence of emotional and spiritual intelligence on smoking cessation intention in college student. Indian Journal of Public Health Research and Development, 10(12), 1651–1655. https://doi.org/10.37506/v10/i12/2019/ijphrd/192098 | Wrong study design |
| Massey S.H., Estabrook R., Lapping-Carr L., Newmark R.L., Decety J., Wisner K.L., & Wakschlag L.S. (2022). Are empathic processes mechanisms of pregnancy’s protective effect on smoking? Identification of a novel target for preventive intervention. Social Science and Medicine, 305, 115071. https://doi.org/10.1016/j.socscimed.2022.115071 | Wrong study design |
| Matsuda, A., Hasegawa, J., Wang, X., Tsunot, S., & Miurat, N. (2015). Psychological characteristics in patients during treatment for tobacco dependence. *Yonago Acta Medica*, *58*(2), 81–84. | Did not analyse mental health outcomes by exposure of interest |
| Matta, J., Hoertel, N., Airagnes, G., Wiernik, E., Limosin, F., Goldberg, M., Zins, M., & Lemogne, C. (2020). Does substance use explain social differences in terms of depression? Findings from the Constances cohort. Comprehensive Psychiatry, 102(do9, 0372612), 152203. https://doi.org/10.1016/j.comppsych.2020.152203 | Wrong study design |
| Mattsson K., Hougaard K.S., & Sejbaek C.S. (2021). Exposure to psychosocial work strain and changes in smoking behavior during pregnancy—A longitudinal study within the Danish national birth cohort. Scandinavian Journal of Work, Environment and Health, 47(1), 70–77. https://doi.org/10.5271/sjweh.3921 | Wrong study design |
| Maxson, P. J., Edwards, S. E., Ingram, A., & Miranda, M. L. (2012b). Psychosocial differences between smokers and non-smokers during pregnancy. Addictive Behaviors, 37(2), 153–159. https://doi.org/10.1016/j.addbeh.2011.08.011 | Did not distinguish between unsuccessful quit attempt and no attempt to quit |
| May, J. R., Jao, N. C., McCarter, K., Klass, E., Pearman, T., Leone, F., Schnoll, R. A., & Hitsman, B. (2021). Change in Health-Related Quality of Life Among Individuals With Cancer Undergoing Smoking Cessation Treatment Involving Varenicline. Oncology Nursing Forum, 48(1), 112–120. https://doi.org/10.1188/21.ONF.112-120 | Wrong outcome |
| May, R., Walker, F., de Burgh, S., Bartrop, R., Tofler, G. H., & Ammerlaan, B. (2019). Pilot study of an internet-based, simulated teachable moment for smoking cessation. Journal of Smoking Cessation, 14(3), 139–148. https://doi.org/10.1017/jsc.2018.32 | Wrong study design |
| McCallum, M., Cooper, B., Matson, S., Renwick, B., & Messeder, S. J. (2021). Improving health behaviors in patients with peripheral arterial disease—A pilot study of supported self-management. Journal of Vascular Nursing : Official Publication of the Society for Peripheral Vascular Nursing, 39(1), 11–16. https://doi.org/10.1016/j.jvn.2020.10.001 | Wrong exposure |
| McCarter K, Baker A, Britton B, Carter G, Beck A, Bauer J, Wolfenden L, Wratten C, McElduff P, & Halpin S. (2015). Continued tobacco smoking, alcohol use and depressive symptoms in a sample of head and neck cancer patients about to undergo radiotherapy. Asia-Pacific Journal of Clinical Oncology, 11, 12. https://doi.org/10.1111/ajco.12444 | Wrong study design |
| McCarthy DE, Minami H, Yeh VM, & Bold KW. (2015). An experimental investigation of reactivity to ecological momentary assessment frequency among adults trying to quit smoking. Addiction, 110(10), 1549–1560. https://doi.org/10.1111/add.12996 | Wrong outcome |
| McClure E.A., Baker N.L., Hood C.O., Tomko R.L., Squeglia L.M., Flanagan J.C., Carpenter M.J., & Gray K.M. (2019). Cannabis and Alcohol Co-Use in a Smoking Cessation Pharmacotherapy Trial for Adolescents and Emerging Adults. Nicotine & Tobacco Research. https://doi.org/10.1093/ntr/ntz170 | Wrong study design |
| McClure, J. B., Catz, S. L., Ludman, E. J., Richards, J., Riggs, K., & Grothaus, L. (2011). Feasibility and acceptability of a multiple risk factor intervention: The Step Up randomized pilot trial. *BMC Public Health*, *11*, 167. | Did not analyse mental health outcomes by exposure of interest |
| McClure, J. B., Swan, G. E., Jack, L., Catz, S. L., Zbikowski, S. M., McAfee, T. A., Deprey, M., Richards, J., & Javitz, H. (2009). Mood, side-effects and smoking otcomes among persons with and without probable lifetime depression taking varenicline. J Gen Intern Med, 24(5), 563–569. https://doi.org/10.1007/s11606-009-0926-8 | Did not analyse mental health outcomes by exposure of interest |
| McDermott, L., Dobson, A., & Owen, N. (2008). Smoking reduction and cessation among young adult women: A 7-year prospective analysis. Nicotine Tob Res, 10(9), 1457–1466. https://doi.org/10.1080/14622200802323241 | Mental health not measured before quit attempt |
| McDonnell B.P. & Regan C. (2019). Smoking in pregnancy: Pathophysiology of harm and current evidence for monitoring and cessation. Obstetrician and Gynaecologist, 21(3), 169–175. https://doi.org/10.1111/tog.12585 | Not primary research |
| McDonnell, B. P., Keogan, S., Clancy, L., & Regan, C. (2019). Smoking cessation support and obstetric outcomes in an Irish maternity hospital. European Journal of Obstetrics, Gynecology, and Reproductive Biology, 232(e4l, 0375672), 1–4. https://doi.org/10.1016/j.ejogrb.2018.11.005 | Wrong outcome |
| Mcgaffin S., Taggart M., Smyth D., O’doherty D., Brown J., Teague S., Slevin C., Montgomery L., Coll M., Lindsay C., Crumley B., Gibson L., Elliott H., Hughes S., & Connolly S. (2021). Transitioning a cardiovascular health and rehabilitation programme to a virtual platform during covid 19. European Journal of Cardiovascular Nursing, 20(SUPPL 1), i78. https://doi.org/10.1093/eurjcn/zvab060.073 | Wrong study design |
| McGee, H. M., Doyle, F., Conroy, R. M., De La Harpe, D., & Shelley, E. (2006). Impact of briefly-assessed depression on secondary prevention outcomes after acute coronary syndrome: A one-year longitudinal survey. BMC Health Serv Res, 6, 9. https://doi.org/10.1186/1472-6963-6-9 | Wrong exposure |
| McGihon, R. E., Burns, R. J., Deschenes, S. S., Schmitz, N., & Anderson, B. (2019). Longitudinal associations between number of cigarettes per day and depressive symptoms in adult smokers with type 2 diabetes: A path analysis approach. Journal of Psychosomatic Research, 125. https://doi.org/10.1016/j.jpsychores.2019.109737 | Wrong exposure |
| McMeekin, N., Sinclair, L., Bauld, L., Tappin, D. M., Mitchell, A., & Boyd, K. A. (2020). A protocol for the economic evaluation of the smoking Cessation in Pregnancy Incentives Trial III (CPIT III). BMJ Open, 10(10), e038827. https://doi.org/10.1136/bmjopen-2020-038827 | Wrong outcome |
| McNutt, M. D. (2017). Examining weight gain in treatment-seeking African American smokers: A biopsychosocial approach. *Dissertation Abstracts International: Section B: The Sciences and Engineering*, *78*(3-B(E)). | Mental health measured at baseline only, no follow up |
| Mdege, N. D., Fairhurst, C., Wang, H.-I., Ferdous, T., Marshall, A.-M., Hewitt, C., Huque, R., Jackson, C., Kellar, I., Parrott, S., Semple, S., Sheikh, A., Wu, Q., Azdi, Z. A., Siddiqi, K., & MCLASS II trial team. (2021). Efficacy and cost-effectiveness of a community-based smoke-free-home intervention with or without indoor-air-quality feedback in Bangladesh (MCLASS II): A three-arm, cluster-randomised, controlled trial. The Lancet. Global Health, 9(5), e639–e650. https://doi.org/10.1016/S2214-109X(21)00040-1 | Wrong study design |
| Mdege, N., Fairhurst, C., Ferdous, T., Hewitt, C., Huque, R., Jackson, C., Kellar, I., Parrott, S., Semple, S., Sheikh, A., Swami, S., & Siddiqi, K. (2019). Muslim Communities Learning About Second-hand Smoke in Bangladesh (MCLASS II): Study protocol for a cluster randomised controlled trial of a community-based smoke-free homes intervention, with or without Indoor Air Quality feedback. Trials, 20(1), 11. https://doi.org/10.1186/s13063-018-3100-y | Wrong outcome |
| Meacham, M. C., Liang, O. S., Zhao, M., Yang, C. C., Thrul, J., & Ramo, D. E. (2021). ‘Connectedness based on shared engagement predicts remote biochemically verified quit status within smoking cessation treatment groups on Facebook’: Corrigendum. Nicotine & Tobacco Research, 23(4), 772. https://doi.org/10.1093/ntr/ntaa039 | Wrong study design |
| Medenblik A.M., Calhoun P.S., Maisto S.A., Kivlahan D.R., Moore S.D., Beckham J.C., Wilson S.M., Blalock D.V., & Dedert E.A. (2021). Pilot Cohorts for Development of Concurrent Mobile Treatment for Alcohol and Tobacco Use Disorders. Substance Abuse: Research and Treatment, 15. https://doi.org/10.1177/11782218211030524 | Wrong study design |
| Megias-Robles, A., Perea-Baena, J. M., Fernandez-Berrocal, P., & Abdollahi, B. (2020). The protective role of emotional intelligence in smoking relapse during a 12-month followup smoking cessation intervention. PLoS ONE, 15(6). https://doi.org/10.1371/journal.pone.0234301 | Wrong study design |
| Mehta J.J., Kapadia N.A., Kharadi R.D., & Shah A.R. (2022). Effect of Age and Preexisting Obstructive Diseases on Smoking Cessation Rate. *European Journal of Molecular and Clinical Medicine*, *9*(3), 5045–5054. | Wrong study design |
| Meier, E., Lindgren, B. R., Anderson, A., Reisinger, S. A., Norton, K. J., Jensen, J., Strayer, L., Dick, L., Tang, M.-K., Chen, M., Carmella, S. G., Hecht, S. S., Murphy, S. E., Yang, J., Stepanov, I., O’Connor, R. J., Shields, P. G., Hatsukami, D. K., & Bates, B. (2020). A randomized clinical trial of snus examining the effect of complete versus partial cigarette substitution on smoking-related behaviors, and biomarkers of exposure. Nicotine & Tobacco Research, 22(4), 473–481. https://doi.org/10.1093/ntr/ntz055 | Wrong study design |
| Melamed J. & Deepak J. (2020). Integrating tobacco health into mainstream pulmonary clinic: a novel approach. Chest, 158(4 Supplement), A2374. https://doi.org/10.1016/j.chest.2020.08.2015 | Wrong study design |
| Metse AP, Wiggers J, Wye P, Wolfenden L, Freund M, Clancy R, Stockings E, Terry M, Allan J, Colyvas K, Prochaska JJ, & Bowman JA. (2017). Efficacy of a universal smoking cessation intervention initiated in inpatient psychiatry and continued post-discharge: A randomised controlled trial. Australian and New Zealand Journal of Psychiatry, 51(4), 366–381. https://doi.org/10.1177/0004867417692424 | Mental health measured at baseline only, no follow up |
| Miguez, M. C., & Pereira, B. (2021). Factors Associated with Smoking Relapse in the Early Postpartum Period: A Prospective Longitudinal Study in Spain. Maternal and Child Health Journal, 25(6), 998–1006. https://doi.org/10.1007/s10995-020-03019-w | Wrong outcome |
| Miguez, M. C., Pereira, B., Pinto, T. M., Figueiredo, B., & Al-Sahab, A. (2019). Continued tobacco consumption during pregnancy and women’s depression and anxiety symptoms. International Journal of Public Health, 64(9), 1355–1365. https://doi.org/10.1007/s00038-019-01308-y | Wrong exposure |
| Miles, D. R. B., Bilal, U., Hutton, H. E., Lau, B., Lesko, C. R., Fojo, A., McCaul, M. E., Keruly, J., Moore, R. D., & Chander, G. (2019). Tobacco Smoking, Substance Use, and Mental Health Symptoms in People with HIV in an Urban HIV Clinic. Journal of Health Care for the Poor and Underserved, 30(3), 1083–1102. https://doi.org/10.1353/hpu.2019.0075 | Wrong study design |
| Minami H, Bloom EL, Reed KMP, Hayes SC, & Brown RA. (2015). The moderating role of experiential avoidance in the relationships between internal distress and smoking behavior during a quit attempt. Psychology of Addictive Behaviors, 29(2), 400–407. https://doi.org/10.1037/adb0000030 | Did not analyse mental health outcomes by exposure of interest |
| Minami, H., Nahvi, S., Arnsten, J. H., Brinkman, H. R., Rivera-Mindt, M., Wetter, D. W., Bloom, E. L., Price, L. H., Richman, E. K., Betzler, T. F., Stockmal, C., Donnelly, R., McClain, L. M., Kennedy, K. A., Vieira, C., Fine, M., McCarthy, D. E., Thomas, J. G., Hecht, J., … Baker, B. (2022). A pilot randomized controlled trial of smartphone-assisted mindfulness-based intervention with contingency management for smokers with mood disorders. Experimental and Clinical Psychopharmacology, 30(5), 653–665. https://doi.org/10.1037/pha0000506 | Wrong study design |
| Minian, N., Ahad, S., Ivanova, A., Veldhuizen, S., Zawertailo, L., Ravindran, A., de Oliveira, C., Baliunas, D., Mulder, C., Bolbocean, C., & Selby, P. (2021). The effectiveness of generic emails versus a remote knowledge broker to integrate mood management into a smoking cessation programme in team-based primary care: A cluster randomised trial. Implementation Science : IS, 16(1), 30. https://doi.org/10.1186/s13012-021-01091-6 | Wrong study design |
| Minian, N., Lingam, M., Moineddin, R., Thorpe, K. E., Veldhuizen, S., Dragonetti, R., Zawertailo, L., Taylor, V. H., Hahn, M., deRuiter, W. K., Melamed, O., & Selby, P. (2020). Impact of a Web-Based Clinical Decision Support System to Assist Practitioners in Addressing Physical Activity and/or Healthy Eating for Smoking Cessation Treatment: Protocol for a Hybrid Type I Randomized Controlled Trial. JMIR Research Protocols, 9(9), e19157. https://doi.org/10.2196/19157 | Wrong study design |
| Mitchell KE, Johnson-Warrington V, Apps LD, Bankart J, Sewell L, Williams JE, Rees K, Jolly K, Steiner M, Morgan M, & Singh SJ. (2014). A self-management programme for COPD: a randomised controlled trial. The European Respiratory Journal, 44(6), 1538–1547. https://doi.org/10.1183/09031936.00047814 | Wrong exposure |
| Mitchell, J. T., McClernon, F. J., Beckham, J. C., Brown, R. A., Lejuez, C. W., Kollins, S. H., & Baker, B. (2019). Smoking abstinence effects on emotion dysregulation in adult cigarette smokers with and without attention-deficit/hyperactivity disorder. Drug and Alcohol Dependence, 205. https://doi.org/10.1016/j.drugalcdep.2019.107594 | Follow up < 6 weeks |
| Mitjans M., Garcia-Portilla M.P., Garcia-Alvarez L., Sarramea F., Galvana G., Diaz-Mesa E., Bobes-Bascaran T., Al-Halabi S., Elizagarate E., Iglesias C., Saiz-Martinez P.A., Fananas L., Bobes J., & Arias B. (2019). SA104 smoking cessation improves clinical outcome in patients with schizophrenia and is modulated by genetic variability at chrna5 gene. European Neuropsychopharmacology, 29(Supplement 4), S1245–S1246. https://doi.org/10.1016/j.euroneuro.2018.08.326 | Wrong study design |
| Miyazaki, M., & Suzuki, S. (2021). Influence of smoking habits on mental status in Japanese women during the first trimester of pregnancy. The Journal of Maternal-Fetal & Neonatal Medicine, 34(8), 1284–1287. https://doi.org/10.1080/14767058.2019.1635110 | Wrong study design |
| Moayeri, F., Hsueh, Y.-S. (Arthur), Dunt, D., Clarke, P., & Abu Hassan, B. (2021). Smoking cessation and quality of life: Insights from analysis of longitudinal Australian data, an application for economic evaluations. Value in Health, 24(5), 724–732. https://doi.org/10.1016/j.jval.2020.11.022 | Wrong study design |
| Mofors, J., Bjork, A., Richardsdotter Andersson, E., Kvarnstrom, M., Forsblad d’Elia, H., Magnusson-Bucher, S., Padyukov, L., Kockum, I., Hillert, J., Eriksson, P., Mandl, T., Nordmark, G., Alfredsson, L., & Wahren-Herlenius, M. (2020). Cigarette smoking patterns preceding primary Sjogren’s syndrome. RMD Open, 6(3). https://doi.org/10.1136/rmdopen-2020-001402 | Wrong exposure |
| Mohamed A.S., ElHabiby M.M., Elkholy H.A., & Ahmed A.D. (2021). Studying the relationship between tobacco use and depression between Egyptian patients. QJM, 114(SUPPL 1). https://doi.org/10.1093/qjmed/hcab102 | Wrong study design |
| Monroe D.C., McDowell C.P., Kenny R.A., & Herring M.P. (2021). Dynamic associations between anxiety, depression, and tobacco use in older adults: Results from The Irish Longitudinal Study on Ageing. Journal of Psychiatric Research, 139, 99–105. https://doi.org/10.1016/j.jpsychires.2021.05.017 | Wrong outcome |
| Morales M.P., Dominguez C.E., Gonzalez M.M., Ramirez I.M., Sanchez M.M., & Munoz A.A. (2019). Characteristics of patients treated with nicotine replacement therapy (NRT) prescribed during hospitalization. European Respiratory Journal, 54(Supplement 63). https://doi.org/10.1183/13993003.congress-2019.PA2862 | Wrong study design |
| Moreno-Coutino A, Garcia-Anguiano F, Ruiz-Velasco S, & Medina-Mora ME. (2015). Assessment of depressive symptoms in severe smokers with minimal-mild depressive symptomatology receiving pre-smoking abstinence for integrated treatment: A randomized clinical trial. Salud Mental (Mexico City, Mexico), 38(6), 433–439. https://doi.org/10.17711/SM.0185-3325.2015.058 | Did not analyse mental health outcomes by exposure of interest |
| Moriarty, A. S., Louwagie, G. M., Mdege, N. D., Morojele, N., Tumbo, J., Omole, O. B., Bachmann, M. O., Kanaan, M., Turner, A., Parrott, S., Siddiqi, K., & Ayo-Yusuf, O. A. (2019). ImPROving TB outcomes by modifying LIFE-style behaviours through a brief motivational intervention followed by short text messages (ProLife): Study protocol for a randomised controlled trial. Trials, 20(1), 457. https://doi.org/10.1186/s13063-019-3551-9 | Wrong study design |
| Moritz, S., Goritz, A. S., Kraj, M., Gehlenborg, J., Hottenrott, B., Tonn, P., Ascone, L., Pedersen, A., Kuhn, S., & Aune, B. (2020). Imaginal retraining reduces cigarette smoking: A randomized controlled study. European Addiction Research, 26(6), 355–364. https://doi.org/10.1159/000509823 | Wrong study design |
| Morris, C. D., Lukowski, A. V., Vargas-Belcher, R. A., Ylioja, T. E., Nash, C. M., Bailey, L. A., & Anderson, A. (2021). Quitline programs tailored for mental health: Initial outcomes and feasibility. Special Issue, 60(3, Suppl 2), S163–S171. https://doi.org/10.1016/j.amepre.2020.02.025 | Wrong study design |
| Morris, C. D., Waxmonsky, J. A., May, M. G., Tinkelman, D. G., Dickinson, M., & Giese, A. A. (2011). Smoking reduction for persons with mental illnesses: 6-month results from community-based interventions. *Community Mental Health Journal*, *47*(6), 694–702. | Did not analyse mental health outcomes by exposure of interest |
| Mouine N. & El Malki Berrada N. (2020b). Evaluation of cardio respiratory efficiency in patients with coronary artery disease: An experience of the first cardiac rehabilitation in Morocco. Archives of Cardiovascular Diseases Supplements, 12(1), 78. https://doi.org/10.1016/j.acvdsp.2019.09.165 | Wrong study design |
| Mouine N. & Malki Berrada N. (2019). Evaluation of benefit of cardiac rehabilitation in Morocco: An experience of the first cardiac rehabilitation unit. Archives of Cardiovascular Diseases Supplements, 11(1), 115. https://doi.org/10.1016/j.acvdsp.2018.10.253 | Wrong study design |
| Muench, C., Malloy, E. J., Juliano, L. M., & Baker, B. (2020). Lower self-efficacy and greater depressive symptoms predict greater failure to recover from a single lapse cigarette. Journal of Consulting and Clinical Psychology, 88(10), 965–970. https://doi.org/10.1037/ccp0000605 | Mental health measured at baseline only, no follow up |
| Mujcic, A., Blankers, M., Boon, B., Verdonck-de Leeuw, I. M., Smit, F., van Laar, M., & Engels, R. (2022). Effectiveness, Cost-effectiveness, and Cost-Utility of a Digital Smoking Cessation Intervention for Cancer Survivors: Health Economic Evaluation and Outcomes of a Pragmatic Randomized Controlled Trial. Journal of Medical Internet Research, 24(3), e27588. https://doi.org/10.2196/27588 | Wrong study design |
| Mumtaz, H., Hameed, M., Sangah, A. B., Zubair, A., & Hasan, M. (2022). Association between smoking and non-alcoholic fatty liver disease in Southeast Asia. Frontiers in Public Health, 10(101616579), 1008878. https://doi.org/10.3389/fpubh.2022.1008878 | Wrong outcome |
| Munoz RF, Aguilera A, Schueller SM, Leykin Y, & Perez-Stable EJ. (2012). From online randomized controlled trials to participant preference studies: Morphing the San Francisco Stop Smoking site into a worldwide smoking cessation resource. Journal of Medical Internet Research, 14(3), e64. https://doi.org/10.2196/jmir.1852 | Mental health measured at baseline only, no follow up |
| Murphy C.M., Micalizzi L., Sokolovsky A.W., Borrelli B., Jennings E.G., Lee C.S., Parker D.R., & Risica P.M. (2021). Motivational interviewing telephone counseling to increase postpartum maintenance of abstinence from tobacco. Journal of Substance Abuse Treatment, 132, 108419. https://doi.org/10.1016/j.jsat.2021.108419 | Did not analyse mental health outcomes by exposure of interest |
| Mussulman, L. M., Scheuermann, T. S., Faseru, B., Nazir, N., & Richter, K. P. (2019). Rapid relapse to smoking following hospital discharge. Preventive Medicine Reports, 15(101643766), 100891. https://doi.org/10.1016/j.pmedr.2019.100891 | Wrong exposure |
| Mweseli R., Sadaka A., Bartlett E., Deveraj A., Kemp S., Addis J., Derbyshire J., Chen M., Morris K., & Hopkinson N. (2020). Participation in a targeted lung health check program and smoking cessation. European Respiratory Journal, 56(Supplement 64). https://doi.org/10.1183/13993003.congress-2020.3063 | Wrong study design |
| Myers M.G., Strong D.R., Chen T.C., & Linke S.E. (2020). Enhancing engagement in evidence-based tobacco cessation treatment for smokers with mental illness: A pilot randomized trial. Journal of Substance Abuse Treatment, 111((Myers, Chen) Veterans Affairs San Diego Healthcare System, 3350 La Jolla Village Dr., San Diego, CA 92161, United States), 29–36. https://doi.org/10.1016/j.jsat.2019.12.012 | Wrong study design |
| Mykletun, A., Overland, S., Aarø, L. E., Liabø, H.-M., & Stewart, R. (2008). Smoking in relation to anxiety and depression: Evidence from a large population survey: The HUNT study. Eur Psychiatry, 23(2), 77–84. https://doi.org/10.1016/j.eurpsy.2007.10.005 | Wrong study design |
| N’Gbo N’Gbo Ikazabo, R., Bier, J.-C., Jamart, J., Mostosi, C., Mavroudakis, N., & Abdolahi, A. (2022). Impact on quitting smoking of cognitive impairment in stroke patients. Journal of the Neurological Sciences, 439, 1–5. https://doi.org/10.1016/j.jns.2022.120296 | Wrong study design |
| Nagawa, C. S., Ito Fukunaga, M., Faro, J. M., Liu, F., Anderson, E., Kamberi, A., Orvek, E. A., Davis, M., Pbert, L., Cutrona, S. L., Houston, T. K., & Sadasivam, R. S. (2023). Characterizing pandemic-related changes in smoking over time in a cohort of current and former smokers. *Nicotine & Tobacco Research*, *25*(2), 203–210. https://doi.org/10.1093/ntr/ntac033 | Wrong outcome |
| Nagawa, C. S., Wang, B., Davis, M., Pbert, L., Cutrona, S. L., Lemon, S. C., & Sadasivam, R. S. (2022). Examining pathways between family or peer factors and smoking cessation in a nationally representative US sample of adults with mental health conditions who smoke: A structural equation analysis. BMC Public Health, 22(1), 1566. https://doi.org/10.1186/s12889-022-13979-z | Wrong study design |
| Nahum-Shani, I., Potter, L. N., Lam, C. Y., Yap, J., Moreno, A., Stoffel, R., Wu, Z., Wan, N., Dempsey, W., Kumar, S., Ertin, E., Murphy, S. A., Rehg, J. M., & Wetter, D. W. (2021). The mobile assistance for regulating smoking (MARS) micro-randomized trial design protocol. Contemporary Clinical Trials, 110(101242342), 106513. https://doi.org/10.1016/j.cct.2021.106513 | Follow up < 6 weeks |
| Nahvi S, Ning Y, Segal KS, Richter KP, & Arnsten JH. (2014). Varenicline efficacy and safety among methadone maintained smokers: A randomized placebo-controlled trial. Addiction (Abingdon, England), 109(9), 1554–1563. https://doi.org/10.1111/add.12631 | Wrong outcome |
| Nair, U. S., Haynes, P., Collins, B. N., & Asghari, B. (2019). Baseline sleep quality is a significant predictor of quit-day smoking self-efficacy among low-income treatment-seeking smokers. Journal of Health Psychology, 24(11), 1484–1493. https://doi.org/10.1177/1359105317740619 | Wrong outcome |
| Nakajima Y., Yamada S., Nishikido A., Katano-Toki A., Ishida E., Akuzawa M., Sakamaki K., Yamada E., Saito T., Ozawa A., Okada S., Shimomura Y., Kobayashi I., Andou Y., & Yamada M. (2019). Influence of Smoking on Thyroid Function in Japanese Subjects: Longitudinal Study for One Year of On-Off Smoking. Journal of the Endocrine Society, 3(12), 2385–2396. https://doi.org/10.1210/js.2019-00155 | Wrong exposure |
| Nanayakkara G.L., Krincic L., Lightfoot R., Reinhardt W., De Silva K., Senaratne J.M., & Senaratne M.P.J. (2022). Demographics and risk factors that influence the prevalence of depression in patients attending cardiac rehabilitation. Medicine (United States), 101(36), E30470. https://doi.org/10.1097/MD.0000000000030470 | Wrong study design |
| Naughton F., Brown C., High J., Notley C., Mascolo C., Coleman T., Barton G., Shepstone L., Sutton S., Prevost A.T., Crane D., Greaves F., & Hope A. (2021). Randomised controlled trial of a just-in-time adaptive intervention (JITAI) smoking cessation smartphone app: The Quit Sense feasibility trial protocol. BMJ Open, 11(4), 48204. https://doi.org/10.1136/bmjopen-2020-048204 | Wrong study design |
| Nawara, M., Abd El Moneam, M. H. E., Elkholy, H., Elhabiby, M., Rabie, M. A., ELNahas, G., & Abdelrahman, A. (2021). A clinical trial to assess the role of repetitive transcranial magnetic stimulation in smoking cessation in an Egyptian sample. Addictive Disorders & Their Treatment, 20(4), 554–566. https://doi.org/10.1097/ADT.0000000000000292 | Wrong study design |
| Nawaz, B., Zadeh, Z., Yousuf, T., Maheshwary, N., Das, S., & Khan, M. A. (2024). Effectiveness of Cognitive Behavioral Hypnotherapy to Reduce Smoking in Anxiety-Prone Individuals. *Medical Forum Monthly*, *35*(4), 35-39. https://doi.org/10.60110/medforum.350408 | Did not analyse mental health outcomes by exposure of interest |
| NCT01553084. (2012). A comparative effectiveness & long term health study in Wisconsin smokers. *Https://Clinicaltrials.Gov/Show/NCT01553084*. | Wrong outcome |
| NCT01569490. (2012). Striving to Quit: First Breath. *Https://Clinicaltrials.Gov/Show/NCT01569490*. | Did not analyse mental health outcomes by exposure of interest |
| NCT01592695. (2012). Tailored tobacco quitline for rural veterans. *Https://Clinicaltrials.Gov/Show/Nct01592695*. | Did not analyse mental health outcomes by exposure of interest |
| NCT01689168. (2012). Chiropractic treatment with counseling versus counseling alone for promoting smoking cessation. *Https://Clinicaltrials.Gov/Show/NCT01689168*. | Wrong outcome |
| NCT01753141. (2012). Smoking treatment and anxiety management program. *Https://Clinicaltrials.Gov/Show/NCT01753141*. | Did not analyse mental health outcomes by exposure of interest |
| NCT01789125. (2013). Smoking termination / anxiety reduction treatment (ST/ART). *Https://Clinicaltrials.Gov/Show/NCT01789125*. | Did not analyse mental health outcomes by exposure of interest |
| NCT01800019. (2013). The Canadian HIV Quit Smoking Trial: Tackling the Co-morbidities of Depression and Cardiovascular Disease in HIV+ Smokers. *Clinicaltrials.Gov/Ct2/Show/Nct01800019*. | REMOVE |
| NCT01801384. (2013). Voucher Based Incentives to Treat Pregnant Smokers. *Https://Clinicaltrials.Gov/Show/NCT01801384*. | Mental health measured at baseline only, no follow up |
| NCT01860924. (2013). Vigorous Exercise for Depressed Smokers. *Https://Clinicaltrials.Gov/Show/NCT01860924*. | Did not analyse mental health outcomes by exposure of interest |
| NCT01892813. (2013). Dissemination of a tailored tobacco quitline for rural veteran smokers. *Https://Clinicaltrials.Gov/Show/Nct01892813*. | Did not analyse mental health outcomes by exposure of interest |
| NCT01964898. (2013). Post acute coronary event smoking study. *Https://Clinicaltrials.Gov/Show/NCT01964898*. | Wrong outcome |
| NCT02211430. (2014). Smoking cessation for low-income pregnant women. *Https://Clinicaltrials.Gov/Show/NCT02211430*. | Mental health measured at baseline only, no follow up |
| NCT02245308. (2014). Abstinence reinforcement therapy (ART) for homeless veteran smokers. *Https://Clinicaltrials.Gov/Show/Nct02245308*. | Wrong outcome |
| NCT02327104. (2014). Effectiveness of mindfulness based relapse prevention for tobacco dependents. *Https://Clinicaltrials.Gov/Show/NCT02327104*. | Did not analyse mental health outcomes by exposure of interest |
| NCT02378714. (2015). Behavioral activation and varenicline for smoking cessation in depressed smokers. *Https://Clinicaltrials.Gov/Show/NCT02378714*. | Wrong outcome |
| NCT02422914. (2015). Benefits of tobacco free cigarette. *Https://Clinicaltrials.Gov/Show/NCT02422914*. | Did not analyse mental health outcomes by exposure of interest |
| NCT02432066. (2015). Effects of GTS-21 on smoking behavior and neurocognitive functions. *Https://Clinicaltrials.Gov/Show/NCT02432066*. | Did not analyse mental health outcomes by exposure of interest |
| NCT02500589. (2015). Telephone-based smoking cessation. *Https://Clinicaltrials.Gov/Show/Nct02500589*. | Did not analyse mental health outcomes by exposure of interest |
| NCT02506829. (2015). Financial incentives for smoking treatment. *Https://Clinicaltrials.Gov/Show/NCT02506829*. | Wrong outcome |
| NCT02511236. (2015). Addressing racial/ethnic tobacco health disparities via group intervention. *Https://Clinicaltrials.Gov/Show/NCT02511236*. | Did not analyse mental health outcomes by exposure of interest |
| NCT02538601. (2015). Enhanced smoking cessation intervention for smokers exposed to the World Trade Center (WTC) disaster. *Https://Clinicaltrials.Gov/Show/NCT02538601*. | Wrong outcome |
| NCT02576899. (2015). Improving functional outcomes of veterans with PTSD and tobacco dependence. *Https://Clinicaltrials.Gov/Show/NCT02576899*. | Wrong outcome |
| NCT02737566. (2016). Small financial incentives to promote smoking cessation. *Https://Clinicaltrials.Gov/Show/NCT02737566*. | Wrong outcome |
| NCT02742610. (2016). Smoking cessation intervention for smokers with depression receiving outpatient psychiatric treatment. *Https://Clinicaltrials.Gov/Show/NCT02742610*. | Wrong outcome |
| NCT02845440. (2016). Integrated smoking cessation treatment for smokers with serious mental illness. *Https://Clinicaltrials.Gov/Show/NCT02845440*. | Wrong outcome |
| NCT02997657. (2016). Positive psychotherapy for smoking cessation enhanced with text messaging: A randomized controlled trial. *Https://Clinicaltrials.Gov/Show/NCT02997657*. | Wrong outcome |
| NCT03029923. (2017). Integrated smoking cessation and mood management for ACS patients. *Https://Clinicaltrials.Gov/Show/NCT03029923*. | Did not analyse mental health outcomes by exposure of interest |
| NCT03371732. (2017). Effectiveness of Screening and Brief Interventions for Alcohol and Tobacco During Breast Cancer Treatment (ONKODETOX). *Https://Clinicaltrials.Gov/Show/NCT03371732*. | Did not analyse mental health outcomes by exposure of interest |
| NCT03413423. (2018). Post acute cardiac event smoking (PACES) study. *Https://Clinicaltrials.Gov/Show/NCT03413423*. | Did not analyse mental health outcomes by exposure of interest |
| NCT03551704. (2018). Smoking cessation treatment for substance use dependents. *Https://Clinicaltrials.Gov/Show/NCT03551704*. | Did not analyse mental health outcomes by exposure of interest |
| NCT03603340. (2018). The ESTxENDS trial- effects of using electronic nicotine delivery systems (ENDSvaporizer/e-cig) on depression. *Https://Clinicaltrials.Gov/Show/NCT03603340*. | Did not analyse mental health outcomes by exposure of interest |
| NCT03837379. (2019). Goal2Quit + NRT Sampling. *Https://Clinicaltrials.Gov/Show/Nct03837379*. | Did not analyse mental health outcomes by exposure of interest |
| NCT03904186. (2019). Effectiveness of an integrated treatment to address smoking cessation and anxiety/ depression in people living with HIV. *Https://Clinicaltrials.Gov/Show/Nct03904186*. | Did not analyse mental health outcomes by exposure of interest |
| NCT03920137. (2019). Adaptation and initial evaluation of transdiagnostic CBT for anxious and depressed smokers. *Https://Clinicaltrials.Gov/Show/Nct03920137*. | Did not analyse mental health outcomes by exposure of interest |
| NCT04088942. (2019). Tobacco STOP in chronic obstructive pulmonary disease-trial—Study protocol. *Https://Clinicaltrials.Gov/Show/Nct04088942*. | Did not analyse mental health outcomes by exposure of interest |
| Neale, J., Parkin, S., Hermann, L., Metrebian, N., Roberts, E., Robson, D., & Strang, J. (2022). Substance use and homelessness: A longitudinal interview study conducted during COVID-19 with implications for policy and practice. The International Journal on Drug Policy, 108(9014759), 103818. https://doi.org/10.1016/j.drugpo.2022.103818 | Wrong study design |
| Neil, J. M., Marotta, C., Gonzalez, I., Chang, Y., Levy, D. E., Wint, A., Harris, K., Hawari, S., Noonan, E., Styklunas, G., Crute, S., Howard, S. E., Sheppard, J., Lennes, I. T., Jacobson, F., Flores, E. J., Haas, J. S., Park, E. R., & Rigotti, N. A. (2021). Integrating tobacco treatment into lung cancer screening practices: Study protocol for the Screen ASSIST randomized clinical trial. Contemporary Clinical Trials, 111(101242342), 106586. https://doi.org/10.1016/j.cct.2021.106586 | Wrong study design |
| Nelson P.R., Chen P., Battista D.R., Pillitteri J.L., & Shiffman S. (2019). Randomized trial to compare smoking cessation rates of snus, with and without smokeless tobacco health-related information, and a nicotine lozenge. Nicotine and Tobacco Research, 21(1), 88–94. https://doi.org/10.1093/ntr/nty011 | Wrong study design |
| Nguyen L.-C., Durazzo T., Rauch A., Dwyer C., & Padula C. (2019). Cigarette smoking in veterans with an alcohol use disorder is associated with relapse 6 months following treatment. Alcoholism: Clinical and Experimental Research, 43(Supplement 1), 193A. https://doi.org/10.1111/acer.14059 | Wrong study design |
| Niaura, R., Britt, D. M., Borrelli, B., Shadel, W. G., Abrams, D. B., & Goldstein, M. G. (1999). History and symptoms of depression among smokers during a self-initiated quit attempt. Nicotine Tob Res, 1(3), 251–257. https://doi.org/10.1080/14622299050011371 | Follow up < 6 weeks |
| Niederstrasser, N. G., Rogers, N. T., Bandelow, S., & Aguayo, A.-O. (2019). Determinants of frailty development and progression using a multidimensional frailty index: Evidence from the English Longitudinal Study of Ageing. PLoS ONE, 14(10). https://doi.org/10.1371/journal.pone.0223799 | Wrong exposure |
| Niedzwiedz, C. L., Green, M. J., Benzeval, M., Campbell, D., Craig, P., Demou, E., Leyland, A., Pearce, A., Thomson, R., Whitley, E., & Katikireddi, S. V. (2021). Mental health and health behaviours before and during the initial phase of the COVID-19 lockdown: Longitudinal analyses of the UK Household Longitudinal Study. Journal of Epidemiology and Community Health, 75(3), 224–231. https://doi.org/10.1136/jech-2020-215060 | Wrong exposure |
| Nieh, C., Mancuso, J. D., Powell, T. M., Welsh, M. M., Gackstetter, G. D., Hooper, T. I., & Ballinger, B. (2021). Cigarette smoking patterns among U.S. military service members before and after separation from the military. PLoS ONE, 16(10). https://doi.org/10.1371/journal.pone.0257539 | Wrong study design |
| Nieh, C., Powell, T. M., Gackstetter, G. D., Hooper, T. I., & Ballinger, B. (2020). Smoking among U.S. Service members following transition from military to veteran status. Health Promotion Practice, 21(1, Suppl), 165S-175S. https://doi.org/10.1177/1524839919881478 | Did not distinguish between unsuccessful quit attempt and no attempt to quit |
| Nizio, P., Smit, T., Matoska, C. T., Chavez, J., Tullos, E. A., Garey, L., Vujanovic, A. A., & Zvolensky, M. J. (2022). Trauma exposure and smoking outcomes: The indirect effects of anxious and depressive symptoms. Addictive Behaviors, 134(2gw, 7603486), 107409. https://doi.org/10.1016/j.addbeh.2022.107409 | Wrong study design |
| Nollen NL, Cox LS, Yu Q, Ellerbeck EF, Scheuermann TS, Benowitz NL, Tyndale RF, Mayo MS, & Ahluwalia JS. (2016). A clinical trial to examine disparities in quitting between African-American and White adult smokers: Design, accrual, and baseline characteristics. Contemporary Clinical Trials, 47, 12–21. https://doi.org/10.1016/j.cct.2015.12.001 | Mental health measured at baseline only, no follow up |
| Noonan, D., Lyna, P., Kennedy, D. L., Gao, X., Bejarano Hernandez, S., Fish, L. J., & Pollak, K. I. (2022). Trajectories of Situational Temptations in Pregnant Smokers participating in a Scheduled Gradual Reduction Cessation Trial. Maternal and Child Health Journal, 26(1), 24–30. https://doi.org/10.1007/s10995-021-03321-1 | Wrong outcome |
| Noureddine, S., Massouh, A., & Abdallah, A.-B. (2019). Factors associated with continued smoking in Lebanese patients with acute coronary syndrome. Journal of Cardiovascular Nursing, 34(1), 94–98. https://doi.org/10.1097/JCN.0000000000000514 | Wrong study design |
| NTR5113. (2015). Blended smoking cessation treatment. *Http://Www.Who.Int/Trialsearch/Trial2.Aspx?TrialID=NTR5113*. | Did not analyse mental health outcomes by exposure of interest |
| Nurhaeni H., Aryani R., Suryati B., & Nuraeni A. (2020). Effectiveness of stress-adaptation and cognitive behavior (SACB) model for independent health recovery for clients with coronary heart disease in the community. Medico-Legal Update, 20(1), 1228–1234. https://doi.org/10.37506/v20/il/2020/mlu/194470 | Wrong study design |
| O’Cleirigh C, Zvolensky MJ, Smits JAJ, Labbe AK, Coleman JN, Wilner JG, Stanton AM, Gonzalez A, Garey L, Regenauer KS, & Rosenfield D. (2018). Integrated Treatment for Smoking Cessation, Anxiety, and Depressed Mood in People Living With HIV: a Randomized Controlled Trial. Journal of Acquired Immune Deficiency Syndromes: JAIDS, 79(2), 261–268. https://doi.org/10.1097/QAI.0000000000001787 | Did not analyse mental health outcomes by exposure of interest |
| O’Connor, M., Whelan, R., Bricker, J., & McHugh, L. (2019). Randomized controlled trial of a smartphone application as an adjunct to acceptance and commitment therapy for smoking cessation. Behavior Therapy. https://doi.org/10.1016/j.beth.2019.06.003 | Wrong outcome |
| Ocalewski, J., Jankowski, M., Zegarski, W., Migdalski, A., & Buczkowski, K. (2023). The Role of Health Behaviors in Quality of Life: A Longitudinal Study of Patients with Colorectal Cancer. *International Journal of Environmental Research and Public Health*, *20*(7), 5416. https://doi.org/10.3390/ijerph20075416 | Wrong study design |
| Ocalewski, J., Michalska, P., Izdebski, P., Jankowski, M., & Zegarski, W. (2021). Fear of Cancer Progression and Health Behaviors in Patients with Colorectal Cancer. American Journal of Health Behavior, 45(1), 138–151. https://doi.org/10.5993/AJHB.45.1.11 | Wrong study design |
| Ock M., Shin J.S., & Ra S.W. (2022). Safety and Effectiveness of Varenicline in Korean Smokers: A Nationwide Post-Marketing Surveillance Study. Patient Preference and Adherence, 16((Ock) Department of Preventive Medicine, Ulsan University Hospital, University of Ulsan College of Medicine, Ulsan, South Korea), 413–426. https://doi.org/10.2147/PPA.S344757 | Wrong study design |
| Ocskay K., Juhasz M., Farkas N., Szakacs Z., Zemplenyi A., Erdosi D., Parniczky A., & Hegyi P. (2020). Recurrent Acute Pancreatitis Prevention by the Elimination of Alcohol and Cigarette Smoking (REAPPEAR): Protocol of a randomized controlled trial and cohort study. Pancreatology, 20(Supplement 1), S79. https://doi.org/10.1016/j.pan.2020.07.120 | Wrong outcome |
| Offit L., O’Sullivan M.M., Lindner R., & Rahim A. (2019). Psychiatric diagnosis and treatment success in a community-based smoking cessation clinic. *American Journal of Respiratory and Critical Care Medicine*, *199*(9). | Wrong study design |
| Okati-Aliabad, H., Ansari-Moghaddam, A., Roohafza, H., Mohammadi, M., Vakili, L., Abbasi, M. H., Heidari, H., Masoudy, G., Yazdekhasti, S., & Sadeghi, M. (2022). The Effects of Comprehensive Home-Based Cardiac Rehabilitation versus Usual Care in Patients with Ischemic Heart Disease in Iran: Study Protocol for a Multicenter Randomized Controlled Trial. International Journal of Preventive Medicine, 13(101535380), 4. https://doi.org/10.4103/ijpvm.IJPVM_492_20 | Wrong study design |
| Okoli, C. T. C., & Khara, M. (2014). Smoking cessation outcomes and predictors among individuals with co-occurring substance use and/or psychiatric disorders. Journal of Dual Diagnosis, 10(1), 9–18. https://doi.org/10.1080/15504263.2013.866860 | Wrong outcome |
| Olando, Y., Kuria, M., Mathai, M., & Huffman, M. D. (2019). Efficacy of a group tobacco cessation behavioral intervention among tobacco users with concomitant mental illness in Kenya: Protocol for a controlled clinical trial. BMC Public Health, 19(1), 1700. https://doi.org/10.1186/s12889-019-8040-2 | Wrong study design |
| Olson, A. L., Boardman, M. B., & Johnson, D. J. (2019). Smoke-Free Moms: Financial Rewards for Smoking Cessation by Low-Income Rural Pregnant Women. American Journal of Preventive Medicine, 56(6), 852–859. https://doi.org/10.1016/j.amepre.2019.02.008 | Wrong study design |
| Oncken, C., Allen, S., Litt, M., Kenny, A., Lando, H., Allen, A., Domelas, E., & Alberg, A. (2020). Exercise for smoking cessation in postmenopausal women: A randomized, controlled trial. Nicotine & Tobacco Research, 22(9), 1587–1595. https://doi.org/10.1093/ntr/ntz176 | Wrong study design |
| Ong J, Plueckhahn I, Cruickshank D, Churilov L, & Mileshkin L. (2016). A smoking cessation programme for current and recent ex-smokers following diagnosis of a potentially curable cancer. Internal Medicine Journal, 46(9), 1089–1096. https://doi.org/10.1111/imj.13172 | Mental health measured at baseline only, no follow up |
| Ordell, S., & Ekback, G. (2019). Smoking cessation and associated dental factors in a cohort of smokers born in 1942: 5 year follow up. International Dental Journal, 69(2), 107–112. https://doi.org/10.1111/idj.12418 | Wrong study design |
| Otto, M. W., Zvolensky, M. J., Rosenfield, D., Hoyt, D. L., Witkiewitz, K., McKee, S. A., Bickel, W. K., Smits, J. A. J., & Arch, A. (2020). A randomized controlled trial protocol for engaging distress tolerance and working memory to aid smoking cessation in low socioeconomic status (SES) adults. Special Issue: The Science of Behavior Change: Implementing the Experimental Medicine Approach, 39(9), 815–825. https://doi.org/10.1037/hea0000858 | Wrong study design |
| Ozaki Y., Komiyama M., Ueshima K., Iso H., Sakata S., Morino A., Takahara M., Noguchi S., Kuwabara Y., Takahashi Y., & Hasegawa K. (2019). Determining the effects of exercise after smoking cessation therapy completion on continuous abstinence from smoking: Japanese study protocol. Trials, 20(1), 734. https://doi.org/10.1186/s13063-019-3820-7 | Did not analyse mental health outcomes by exposure of interest |
| Ozcelik, N., & Yilmaz Kara, B. (2021). Effect of coronaphobia on smoking habits. Journal of Addictive Diseases, 39(2), 241–247. https://doi.org/10.1080/10550887.2020.1849950 | Wrong study design |
| Pacheco, V. A., Fernandez, A. P. G.-B., Morales, A. V., Crespo, E. L., Garcia, S. M., & Rubio, T. M. (2017). Anxiety, depression and tobacco abstinence. *Adicciones*, *29*(4), 233–244. | Did not analyse mental health outcomes by exposure of interest |
| Pang, R. D., Chai, S. H., Tucker, C. J., Weinberger, A. H., D’Orazio, L. M., Kirkpatrick, M. G., & Aubin, A.-M. (2022). Effects of cigarette abstinence on negative and positive affect by depression symptom levels: A lab study. Journal of Affective Disorders, 307, 163–170. https://doi.org/10.1016/j.jad.2022.03.052 | Wrong study design |
| Papachristou, C., & Humair, J.-P. (2022). [Tobacco cessation treatment for people with schizophrenia]. Traitement Pharmacologique d’aide Au Sevrage Tabagique Chez Les Patients Souffrant de Schizophrenie., 18(787), 1275–1278. https://doi.org/10.53738/REVMED.2022.18.787.1275 | Wrong study design |
| Paraje, G., & Valdes, N. (2021). Changes in parental smoking behavior and children’s health status in Chile. Preventive Medicine, 153(pm4, 0322116), 106792. https://doi.org/10.1016/j.ypmed.2021.106792 | Wrong study design |
| Park AH, Lee SJ, & Oh SJ. (2015). The effects of a smoking cessation programme on health-promoting lifestyles and smoking cessation in smokers who had undergone percutaneous coronary intervention. International Journal of Nursing Practice, 21(2), 107–117. https://doi.org/10.1111/ijn.12230 | Wrong outcome |
| Park M.-J., Seo Y.-G., Noh H.-M., Kim Y., Yoon J.L., & Paek Y.-J. (2021). Effectiveness of national residential smoking cessation program. International Journal of Environmental Research and Public Health, 18(18), 9901. https://doi.org/10.3390/ijerph18189901 | Wrong study design |
| Park, D. W., Jang, J.-Y., Park, T. S., Lee, H., Moon, J.-Y., Kim, S.-H., Kim, T.-H., Yoon, H. J., Kang, D. R., & Sohn, J. W. (2020). Burden of male hardcore smokers and its characteristics among those eligible for lung cancer screening. BMC Public Health, 20(1), 151. https://doi.org/10.1186/s12889-020-8266-z | Wrong study design |
| Park, E. R., Skurla, S. E., Caballero, G. Y. J., Friedman, E. R., Ponzani, C., Wallace, R., Malin, J., & Keating, N. L. (2022). Long-term follow-up of smokers following lung and colorectal cancer diagnosis. Supportive Care in Cancer, 30(9), 7801–7809. https://doi.org/10.1007/s00520-022-07111-5 | Wrong exposure |
| Park, E., Chang, Y., Quinn, V., Regan, S., Cohen, L., Viguera, A., Psaros, C., Ross, K., & Rigotti, N. (2009). The association of depressive, anxiety, and stress symptoms and postpartum relapse to smoking: A longitudinal study. Nicotine & Tobacco Research : Official Journal of the Society for Research on Nicotine and Tobacco, 11, 707–714. https://doi.org/10.1093/ntr/ntp053 | Mental health not measured before quit attempt |
| Park, S.-K., Kang, D.-W., & Lee, E.-K. (2022). Cost-Effectiveness Analysis of Smoking Cessation Interventions With Behavioral Support: A Study Based on the Benefits of Smoking Cessation on Outcomes (BENESCO) Model. Nicotine & Tobacco Research, 24(12), 2011–2017. https://doi.org/10.1093/ntr/ntac172 | Wrong study design |
| Parkerson, H. A., Sareen, J., Asmundson, G. J. G., & Abrams, A. (2021). Breaking the cycle of smoking and pain: Do pain-related anxiety and pain reduction expectancies sabotage attempts to quit smoking and can smoking cessation improve pain and pain-related disability outcomes? Cognitive Behaviour Therapy, 50(2), 154–171. https://doi.org/10.1080/16506073.2020.1798498 | Did not distinguish between unsuccessful quit attempt and no attempt to quit |
| Parkerson, H. E. R. (2021). A pilot test of an internet-based smoking cessation intervention: Outcomes across chronic pain and pain-free samples. *Dissertation Abstracts International: Section B: The Sciences and Engineering*, *82*(3-B), No-Specified. | Wrong study design |
| Partos, T. R., Hiscock, R., Gilmore, A. B., Branston, J. R., Hitchman, S., & McNeill, A. (2020). Impact of tobacco tax increases and industry pricing on smoking behaviours and inequalities: A mixed-methods study. https://doi.org/10.3310/phr08060 | Wrong study design |
| Patel Y., Churchill I., Sullivan K., Beauchamp M., Wald J., Mbuagbaw L., Agzarian J., Shargall Y., Finley C., Fahim C., & Hanna W. (2021). Move For Surgery, a novel preconditioning program to optimize health before thoracic surgery: A randomized controlled trial. CMAJ. Canadian Medical Association Journal, 64(6 Supplement 2), S107. https://doi.org/10.1503/cjs.021321 | Did not analyse mental health outcomes by exposure of interest |
| Patil S.S. (2022). Treatment Outcomes of Anti-Tubercular Therapy in Chronic Smokers. American Journal of Respiratory and Critical Care Medicine, 205(1). https://doi.org/10.1164/ajrccm-conference.2022.205.1_MeetingAbstracts.A5645 | Wrong study design |
| Patten CA, Bronars CA, Vickers Douglas KS, Ussher MH, Levine JA, Tye SJ, Hughes CA, Brockman TA, Decker PA, DeJesus RS, Williams MD, Olson TP, Clark MM, & Dieterich AM. (2017). Supervised, vigorous intensity exercise intervention for depressed female smokers: A pilot study. Nicotine & Tobacco Research, 19(1), 77–86. https://doi.org/10.1093/ntr/ntw208 | Did not analyse mental health outcomes by exposure of interest |
| Patten, C. A., Hiratsuka, V. Y., Nash, S. H., Day, G., Redwood, D. G., Beans, J. A., Howard, B. V., Umans, J. G., & Koller, K. R. (2022). Smoking Patterns Among Urban Alaska Native and American Indian Adults: The Alaska EARTH 10-Year Follow-up Study. Nicotine & Tobacco Research, 24(6), 840–846. https://doi.org/10.1093/ntr/ntab245 | Mental health measured at baseline only, no follow up |
| Patten, C. A., Koller, K. R., Flanagan, C. A., Hiratsuka, V., Merritt, Z. T., Sapp, F., Meade, C. D., Hughes, C. A., Decker, P. A., Murphy, N., & Thomas, T. K. (2019). Postpartum tobacco use and perceived stress among alaska nativewomen: MAWPhase 4 study. International Journal of Environmental Research and Public Health, 16(17). https://doi.org/10.3390/ijerph16173024 | Mental health measured at baseline only, no follow up |
| Patterson, F., Robson, S., McGarry, C., Taylor, D., Halvorsen, S., Rex, S., Landgraf, R., & America, A. (2020). Testing the feasibility of a system-based approach to deliver a smoking cessation and food nudging intervention at food pantry sites. Translational Behavioral Medicine, 10(1), 146–154. https://doi.org/10.1093/tbm/ibz078 | Wrong outcome |
| Paul C.L., Tzelepis F., Boyes A.W., D’Este C., Sherwood E., & Girgis A. (2019). Continued smoking after a cancer diagnosis: A longitudinal study of intentions and attempts to quit. Journal of Cancer Survivorship : Research and Practice, 13(5), 687–694. https://doi.org/10.1007/s11764-019-00787-5 | Wrong study design |
| Paul, C. L., Warren, G., Vinod, S., Meiser, B., Stone, E., Barker, D., White, K., McLennan, J., Day, F., McCarter, K., McEnallay, M., Tait, J., Canfell, K., Weber, M., & Segan, C. (2021). Care to Quit: A stepped wedge cluster randomised controlled trial to implement best practice smoking cessation care in cancer centres. Implementation Science : IS, 16(1), 23. https://doi.org/10.1186/s13012-021-01092-5 | Did not analyse mental health outcomes by exposure of interest |
| Paulus, D. J., Gallagher, M. W., Raines, A. M., Schmidt, N. B., & Zvolensky, M. J. (2019). Intraindividual change in anxiety sensitivity and alcohol use severity 12-months following smoking cessation treatment. Behaviour Research and Therapy, 116, 10–18. https://doi.org/10.1016/j.brat.2019.01.008 | Did not distinguish between unsuccessful quit attempt and no attempt to quit |
| Paz Castro, R., Haug, S., Wenger, A., & Schaub, M. P. (2022). Longer-Term Efficacy of a Digital Life-Skills Training for Substance Use Prevention. American Journal of Preventive Medicine, 63(6), 944–953. https://doi.org/10.1016/j.amepre.2022.06.017 | Wrong study design |
| Pearson, J. L., Sharma, E., Rui, N., Halenar, M. J., Johnson, A. L., Cummings, K. M., Hammad, H. T., Kaufman, A. R., Tworek, C., Goniewicz, M. L., Kimmel, H. L., Tanski, S., Compton, W. M., Day, H., Ambrose, B. K., Bansal-Travers, M., Silveira, M. L., Abrams, D., Limpert, J., … Stanton, C. A. (2020). Association of Electronic Nicotine Delivery System Use With Cigarette Smoking Progression or Reduction Among Young Adults. JAMA Network Open, 3(11), e2015893. https://doi.org/10.1001/jamanetworkopen.2020.15893 | Wrong outcome |
| Peckham E, Arundel C, Bailey D, Crosland S, Fairhurst C, Heron P, Hewitt C, Li J, Parrott S, Bradshaw T, Horspool M, Hughes E, Hughes T, Ker S, Leahy M, McCloud T, Osborn D, Reilly J, Steare T, … Gilbody S. (2019). A bespoke smoking cessation service compared with treatment as usual for people with severe mental ill health: The SCIMITAR+ RCT. Health Technology Assessment, 23(50), 1–116. https://doi.org/10.3310/hta23500 | Did not analyse mental health outcomes by exposure of interest |
| Peckham, E., Allgar, V., Crosland, S., Heron, P., Johnston, G., Newbronner, E., Ratschen, E., Spanakis, P., Wadman, R., Walker, L., Gilbody, S., & Dregan, D. (2021). Investigating smoking and nicotine dependence among people with severe mental illness during the COVID-19 pandemic: Analysis of linked data from a UK Closing the Gap cohort. BJPsych Open, 7. https://doi.org/10.1192/bjo.2021.45 | Wrong study design |
| Pedersen, E. R., Linnemayr, S., Shadel, W. G., Zutshi, R., DeYoreo, M., Cabreros, I., & Tucker, J. S. (2022). Substance Use and Mental Health Outcomes from a Text Messaging-Based Intervention for Smoking Cessation Among Young People Experiencing Homelessness. Nicotine & Tobacco Research, 24(1), 130–134. https://doi.org/10.1093/ntr/ntab160 | Wrong study design |
| Pederson, L. L., Wanklin, J. M., & Lefcoe, N. M. (1988). Self-reported long-term smoking cessation in patients with respiratory disease: Prediction of success and perception of health effects. Int J Epidemiol, 17(4), 804–809. https://doi.org/10.1093/ije/17.4.804 | Mental health not measured before quit attempt |
| Peltier, M. R. (2022). Motivation for tobacco cessation among nicotine dependent postmenopausal females. *Dissertation Abstracts International: Section B: The Sciences and Engineering*, *83*(12-B), No-Specified. | Wrong study design |
| Peltier, M. R., Flores, J. M., Smith, P. H., Roberts, W., Verplaetse, T. L., Moore, K. E., Hacker, R., Oberleitner, L. M., & McKee, S. A. (2019). Smoking across the menopausal transition in a 10-year longitudinal sample: The role of sex hormones and depressive symptoms. *Nicotine & Tobacco Research*, *06*, 06. | Did not analyse mental health outcomes by exposure of interest |
| Peltzer Karl & Pengpid Supa. (2015). Anxiety and depression symptoms following smoking cessation and/or brief alcohol treatment among moderate risk smokers and drinkers. *Journal of Psychology in Africa*, *25*(4), 361–363. | Did not distinguish between unsuccessful quit attempt and no attempt to quit |
| Pereira, B., Figueiredo, B., Miguel Pinto, T., & Miguez, M. C. (2022). Tobacco consumption from the 1st trimester of pregnancy to 7 months postpartum: Effects of previous tobacco consumption, and depression and anxiety symptoms. Addictive Behaviors, 124(2gw, 7603486), 107090. https://doi.org/10.1016/j.addbeh.2021.107090 | Did not distinguish between unsuccessful quit attempt and no attempt to quit |
| Pereira, B., Figueiredo, B., Pinto, T. M., & Miguez, M. C. (2020). Effects of Tobacco Consumption and Anxiety or Depression during Pregnancy on Maternal and Neonatal Health. International Journal of Environmental Research and Public Health, 17(21). https://doi.org/10.3390/ijerph17218138 | Wrong exposure |
| Perez, G. K., Gareen, I. F., Sicks, J., Lathan, C., Carr, A., Kumar, P., Ponzani, C., Hyland, K., Park, E. R., & Bach, B. (2019). Racial differences in smoking-related disease risk perceptions among adults completing lung cancer screening: Follow-up results from the ACRIN/NLST ancillary study. Journal of Racial and Ethnic Health Disparities, 6(4), 676–685. https://doi.org/10.1007/s40615-019-00566-z | Wrong study design |
| Perez-Pareja, F. J., Garcia-Pazo, P., Jimenez, R., Escalas, T., Gervilla, E., & Almadana, A.-P. (2020). Quitting smoking, cognitive behavioral therapy and differential profiles with decision trees. Dejar de Fumar, Terapia Cognitivo-Conductual y Perfiles Diferenciales Con Arboles de Decision., 31(3), 137–145. https://doi.org/10.5093/clysa2020a12 | Wrong study design |
| Perkins, K. A., Marcus, M. D., Levine, M. D., D’Amico, D., Miller, A., Broge, M., Ashcom, J., & Shiffman, S. (2001). Cognitive–behavioral therapy to reduce weight concerns improves smoking cessation outcome in weight-concerned women. Journal of Consulting and Clinical Psychology, 69(4), 604–613. https://doi.org/10.1037/0022-006X.69.4.604 | Did not analyse mental health outcomes by exposure of interest |
| Peterson J, Prochazka AV, & Battaglia C. (2015). Smoking cessation and care management for veterans with posttraumatic stress disorder: A study protocol for a randomized controlled trial. BMC Health Services Research, 15(1), 46. https://doi.org/10.1186/s12913-015-0706-6 | Mental health not measured before quit attempt |
| Petrie, K., Abramson, M. J., & George, J. (2024). Case-Finding and Treatment Effects in COPD: Secondary Analysis of an Interdisciplinary Intervention Trial. *International Journal of COPD*, *19*, 451-458. https://doi.org/10.2147/COPD.S436690 | Wrong exposure |
| Phusahat P., Dilokthornsakul P., Boonsawat W., Zaeoue U., Hansuri N., Tawinkan N., Theeranut A., & Lertsinudom S. (2022). Efficacy and Safety of Cytisine in Combination with a Community Pharmacists’ Counselling for Smoking Cessation in Thailand: A Randomized Double-Blinded Placebo-Controlled Trial. International Journal of Environmental Research and Public Health, 19(20), 13358. https://doi.org/10.3390/ijerph192013358 | Wrong study design |
| Piasecki, T. M., Fiore, M. C., & Baker, T. B. (1998). Profiles in discouragement: Two studies of variability in the time course of smoking withdrawal symptoms. Journal of Abnormal Psychology, 107(2), 238–251. https://doi.org/10.1037/0021-843x.107.2.238 | Wrong outcome |
| Piasecki, T., Kenford, S., Smith, S. S., Fiore, M., & Baker, T. (1997). Listening to nicotine: Negative affect and the smoking withdrawal conundrum. https://doi.org/10.1111/j.1467-9280.1997.tb00409.x | Not primary research |
| Plebani JG, Lynch KG, Rennert L, Pettinati HM, O’Brien CP, & Kampman KM. (2013). Results from a pilot clinical trial of varenicline for the treatment of alcohol dependence. Drug and Alcohol Dependence, 133(2), 754–758. https://doi.org/10.1016/j.drugalcdep.2013.06.019 | Did not analyse mental health outcomes by exposure of interest |
| Plever, S., McCarthy, I., Anzolin, M., Emmerson, B., Allan, J., Hay, K., & Campion, D. (2020). Queensland smoking care in adult acute mental health inpatient units: Supporting practice change. Australian and New Zealand Journal of Psychiatry, 54(9), 919–927. https://doi.org/10.1177/0004867420917443 | Wrong study design |
| Pollak KI, Fish LJ, Sutton LM, Gao X, Lyna P, Owen L, Patel ML, & Somers TJ. (2018). A smoking cessation and pain management program for cancer survivors. Journal of Cancer Survivorship, 12(6), 821–827. https://doi.org/10.1007/s11764-018-0719-3 | Did not analyse mental health outcomes by exposure of interest |
| Polosa, R., Morjaria, J. B., Prosperini, U., Busa, B., Pennisi, A., Gussoni, G., Rust, S., Maglia, M., & Caponnetto, P. (2021). Health outcomes in COPD smokers using heated tobacco products: A 3-year follow-up. Internal and Emergency Medicine, 16(3), 687–696. https://doi.org/10.1007/s11739-021-02674-3 | Wrong study design |
| Polosa, R., Morjaria, J. B., Prosperini, U., Busa, B., Pennisi, A., Malerba, M., Maglia, M., & Caponnetto, P. (2020). COPD smokers who switched to e-cigarettes: Health outcomes at 5-year follow up. Therapeutic Advances in Chronic Disease, 11(101532140), 2040622320961617. https://doi.org/10.1177/2040622320961617 | Wrong study design |
| Poltyn-Zaradna, K., Psikus, P., & Zatonska, K. (2022). Changes in Attitudes toward Tobacco Smoking and Factors Associated with Quitting in 9-Year Observation of PURE Poland Cohort Study. International Journal of Environmental Research and Public Health, 19(11). https://doi.org/10.3390/ijerph19116564 | Wrong study design |
| Postolache, P., Nemes, R. M., Petrescu, O., & Merisanu, I. O. (2015). Smoking cessation, pulmonary rehabilitation and quality of life at smokers with COPD. *Revista Medico-Chirurgicala a Societatii de Medici Si Naturalisti Din Iasi*, *119*(1), 77–80. | Wrong outcome |
| Potretzke S., Lemieux A., Nakajima M., & al’Absi M. (2022). Circulating ghrelin changes as a biomarker of the stress response and craving in abstinent smokers. Pharmacology Biochemistry and Behavior, 218, 173423. https://doi.org/10.1016/j.pbb.2022.173423 | Wrong study design |
| Powers MB, Kauffman BY, Kleinsasser AL, Lee-Furman E, Smits JA, Zvolensky MJ, & Rosenfield D. (2016). Efficacy of smoking cessation therapy alone or integrated with prolonged exposure therapy for smokers with PTSD: study protocol for a randomized controlled trial. Contemporary Clinical Trials, 50(pp 213-221), 213–221. https://doi.org/10.1016/j.cct.2016.08.012 | Did not analyse mental health outcomes by exposure of interest |
| Prakash, S., Xu, Y., Goldenson, N. I., Wissmann, R., Gougelet, R., Shiffman, S., & Amato, B. (2021). Transitions in smoking among adults newly purchasing the JUUL system. American Journal of Health Behavior, 45(3), 546–562. https://doi.org/10.5993/AJHB.45.3.9 | Wrong outcome |
| Pratt, R., Xiong, S., Kmiecik, A., Strobel-Ayres, C., Joseph, A., Rose, S. A. E., Luo, X., Cooney, N., Thomas, J., Specker, S., & Okuyemi, K. (2022). The implementation of a smoking cessation and alcohol abstinence intervention for people experiencing homelessness. BMC Public Health, 22(1), 1260. https://doi.org/10.1186/s12889-022-13563-5 | Wrong study design |
| Pratt, S. I., Brunette, M. F., Wolfe, R., Scherer, E. A., Xie, H., Bartels, S., Ferron, J. C., & Capuchino, K. (2019). Incentivizing healthy lifestyle behaviors to reduce cardiovascular risk in people with serious mental illness: An equipoise randomized controlled trial of the wellness incentives program. Contemporary Clinical Trials, 81(101242342), 1–10. https://doi.org/10.1016/j.cct.2019.04.005 | Wrong study design |
| Prochaska, J. J., Brown-Johnson, C., Baiocchi, M., Lazaro, A. S., Chieng, A., Stinson, S., Anzai, N., & Anderson, A. (2020). Treating tobacco dependence to aid re-employment among job-seekers: A randomized controlled trial. Preventive Medicine: An International Journal Devoted to Practice and Theory, 141(Occupational Interests&Guidance [3610]). https://doi.org/10.1016/j.ypmed.2020.106259 | Wrong study design |
| Prochaska, J. J., Gates, E. F., Davis, K. C., Gutierrez, K., Prutzman, Y., & Rodes, R. (2019). The 2016 Tips From Former Smokers R Campaign: Associations With Quit Intentions and Quit Attempts Among Smokers With and Without Mental Health Conditions. Nicotine & Tobacco Research : Official Journal of the Society for Research on Nicotine and Tobacco, 21(5), 576–583. https://doi.org/10.1093/ntr/nty241 | Mental health measured at baseline only, no follow up |
| Prochaska, J. J., Hall, S. M., Tsoh, J. Y., Eisendrath, S., Rossi, J. S., Redding, C. A., Rosen, A. B., Meisner, M., Humfleet, G. L., & Gorecki, J. A. (2008). Treating tobacco dependence in clinically depressed smokers: Effect of smoking cessation on mental health functioning. American Journal of Public Health, 98(3), 446–448. https://doi.org/10.2105/AJPH.2006.101147 | Did not distinguish between unsuccessful quit attempt and no attempt to quit |
| Puddey, I. B., Vandongen, R., Beilin, L. J., English, D. R., & Ukich, A. W. (1985). The effect of stopping smoking on blood pressure—A controlled trial. J Chronic Dis, 38(6), 483–493. https://doi.org/10.1016/0021-9681(85)90032-3 | Did not analyse mental health outcomes by exposure of interest |
| Pulakka, A., Halonen, J. I., Pentti, J., Kivimaki, M., Vahtera, J., & Stenholm, S. (2019). Changes in Smoking During Retirement Transition: A Longitudinal Cohort Study. Scandinavian Journal of Public Health, 47(8), 876–884. https://doi.org/10.1177/1403494818804408 | Wrong outcome |
| Purborini, N., Lee, M.-B., Devi, H. M., & Chang, H.-J. (2021). Associated factors of depression among young adults in Indonesia: A population-based longitudinal study. Journal of the Formosan Medical Association = Taiwan Yi Zhi, 120(7), 1434–1443. https://doi.org/10.1016/j.jfma.2021.01.016 | Not adult population |
| Qiao, L., & Li, R. (2022). Influence of Personalized Health Management Model Based on Internet Mode on Self-Management Ability and Life Quality of Patients with Chronic Diseases Undergoing Physical Examination. Computational and Mathematical Methods in Medicine, 2022(101277751), 4434436. https://doi.org/10.1155/2022/4434436 | Wrong outcome |
| Quansah, D. & Mullen, K.-A. (2024). Effectiveness of cardioprevent postpartum program on cardiovascular disease risk scores and individual risk factor changes in women with prior hypertensive disorders of pregnancy. *Journal of the American College of Cardiology*, *83*(13 Supplement), 1725. https://doi.org/10.1016/S0735-1097%2824%2903715-X | Wrong exposure |
| Quinn G. & Brown J. (2020). PHarm Reduction—Smoking Cessation by a Pharmacist in a Psychiatric Clinic. *Journal of Addiction Medicine*, *14*(6), e409–e410. | Wrong study design |
| Quinn M.H., Bauer A.-M., Fox E.N., Hatzell J., Randle T., Purnell J., Rogers T., Stevens N., Leone F., Achenbach C., Wileyto E.P., Josephson S., Gollan J., Ashare R., Hitsman B., Schnoll R., & Gross R. (2021). Rationale and design of a randomized factorial clinical trial of pharmacogenetic and adherence optimization strategies to promote tobacco cessation among persons with HIV. Contemporary Clinical Trials, 110, 106410. https://doi.org/10.1016/j.cct.2021.106410 | Wrong study design |
| Quinn, M. H., Bauer, A.-M., Flitter, A., Lubitz, S. F., Ashare, R. L., Thompson, M., Leone, F., Gross, R., Schnoll, R., & Althoff, A. (2020). Correlates of varenicline adherence among smokers with HIV and its association with smoking cessation. Addictive Behaviors, 102. https://doi.org/10.1016/j.addbeh.2019.106151 | Wrong study design |
| Quisenberry, A. J., Pittman, J., Goodwin, R. D., Bickel, W. K., D’Urso, G., Sheffer, C. E., & Adler, A. (2019). Smoking relapse risk is increased among individuals in recovery. Drug and Alcohol Dependence, 202, 93–103. https://doi.org/10.1016/j.drugalcdep.2019.07.001 | Wrong outcome |
| Quittschalle, J., Pabst, A., Lobner, M., Luppa, M., Heser, K., Wagner, M., van den Bussche, H., Hajek, A., Konig, H.-H., Wiese, B., Angermeyer, M. C., Maier, W., Scherer, M., & Riedel-Heller, S. G. (2021). Association of Alcohol and Tobacco Consumption with Depression Severity in the Oldest Old. Results from the Age Different Old Age Cohort Platform. International Journal of Environmental Research and Public Health, 18(15). https://doi.org/10.3390/ijerph18157959 | Wrong study design |
| Rabe, B. J., Stafford, J. W., Hassinger, A. D., Swartzwelder, H. S., & Shofer, S. L. (2022). Implementation and Effectiveness of a Veterans Affairs-Based Comprehensive Lung Cancer Survivorship Program. Journal of Cardiopulmonary Rehabilitation and Prevention, 42(3), 196–201. https://doi.org/10.1097/HCR.0000000000000658 | Wrong exposure |
| Raherison, C., Marjary, A., Valpromy, B., Prevot, S., Fossoux, H., & Taytard, A. (2005). Evaluation of smoking cessation success in adults. Respir Med, 99(10), 1303–1310. https://doi.org/10.1016/j.rmed.2004.12.002 | Mental health measured at baseline only, no follow up |
| Ramclam, A., Taing, M., Kyburz, B., Williams, T., Casey, K., Correa-Fernandez, V., Obasi, E. M., Martinez Leal, I., Chen, T. A., O’Connor, D. P., Reitzel, L. R., & Alexander, B. (2022). An epidemic and a pandemic collide: Assessing the feasibility of tobacco treatment among vulnerable groups at COVID-19 protective lodging. Families, Systems, & Health, 40(1), 120–125. https://doi.org/10.1037/fsh0000658 | Wrong outcome |
| Ramotowski B. & Budaj A. (2021). Is cytisine contraindicated in smoking patients with coronary artery disease after percutaneous coronary intervention? Kardiologia Polska, 79(8), 813–819. https://doi.org/10.33963/KP.a2021.0025 | Wrong study design |
| Ranjit, A., Latvala, A., Kinnunen, T. H., Kaprio, J., Korhonen, T., & Aldi, A. (2020). Depressive symptoms predict smoking cessation in a 20-year longitudinal study of adult twins. Addictive Behaviors, 108. https://doi.org/10.1016/j.addbeh.2020.106427 | Did not distinguish between unsuccessful quit attempt and no attempt to quit |
| Rasmussen, M., Hovhannisyan, K., Adami, J., Tonnesen, H., & Baker, B. (2021). Characteristics of patients in treatment for alcohol and drug addiction who succeed in changing smoking, weight, and physical activity: A secondary analysis of an RCT on combined lifestyle interventions. European Addiction Research, 27(2), 123–130. https://doi.org/10.1159/000510608 | Wrong study design |
| RBR-79wnrc. (2018). Effects of aerobic and resistance exercise associated with health care therapy of those who want to quit smoking. *Http://Www.Who.Int/Trialsearch/Trial2.Aspx?TrialID=RBR-79wnrc*. | Did not analyse mental health outcomes by exposure of interest |
| Redfern, J., Hyun, K., Singleton, A., Hafiz, N., Raeside, R., Spencer, L., Carr, B., Caterson, I., Cullen, J., Ferry, C., Santo, K., Hayes, A., Leung, R. W. M., Raadsma, S., Swinbourne, J., Cho, J. G., King, M., Roberts, M., Kok, C., … Chow, C. (2019). ITM support for patients with chronic respiratory and cardiovascular diseases: A protocol for a randomised controlled trial. BMJ Open, 9(3). https://doi.org/10.1136/bmjopen-2018-023863 | Did not distinguish between unsuccessful quit attempt and no attempt to quit |
| Reed, B. W., Miller, S. A., Bobak, T. J., Stevens, E., Jason, L. A., & Baca, B. (2020). The experience of smoking in recovery settings: An ecological momentary assessment pilot study. Journal of Social Work Practice in the Addictions, 20(1), 59–72. https://doi.org/10.1080/1533256X.2020.1710080 | Wrong study design |
| Reid, H. H. (2017). The moderating effects of protective and risk factors on outcomes for behavioral smoking cessation treatment. *Dissertation Abstracts International: Section B: The Sciences and Engineering*, *78*(1-B(E)), No-Pagination Specified. | Mental health measured at baseline only, no follow up |
| Reif J., Chan D., Jones D., Payne L., & Molitor D. (2020). Effects of a Workplace Wellness Program on Employee Health, Health Beliefs, and Medical Use: A Randomized Clinical Trial. JAMA Internal Medicine, 180(7), 952–960. https://doi.org/10.1001/jamainternmed.2020.1321 | Wrong study design |
| Reinhardt, C., Harden, M., Herrmann-Lingen, C., Rittmeyer, A., & Andreas, S. (2022). Smoking cessation by combined medication and counselling: A feasibility study in lung cancer patients. BMC Pulmonary Medicine, 22(1), 252. https://doi.org/10.1186/s12890-022-02048-1 | Did not analyse mental health outcomes by exposure of interest |
| Rennard S, Hughes J, Cinciripini PM, Kralikova E, Raupach T, Arteaga C, St Aubin LB, Russ C, & Flexible Quit Date Study Group. (2012). A randomized placebo-controlled trial of varenicline for smoking cessation allowing flexible quit dates. Nicotine & Tobacco Research, 14(3), 343–350. https://doi.org/10.1093/ntr/ntr220 | Did not analyse mental health outcomes by exposure of interest |
| Represas-Carrera, F., Couso-Viana, S., Mendez-Lopez, F., Masluk, B., Magallon-Botaya, R., Recio-Rodriguez, J. I., Pombo, H., Leiva-Rus, A., Gil-Girbau, M., Motrico, E., Marti-Lluch, R., Gude, F., & Claveria, A. (2021). Effectiveness of a Multicomponent Intervention in Primary Care That Addresses Patients with Diabetes Mellitus with Two or More Unhealthy Habits, Such as Diet, Physical Activity or Smoking: Multicenter Randomized Cluster Trial (EIRA Study). International Journal of Environmental Research and Public Health, 18(11). https://doi.org/10.3390/ijerph18115788 | Wrong study design |
| Reuven, S. M., Chen, T.-A., Zvolensky, M. J., Businelle, M. S., Kendzor, D. E., Reitzel, L. R., & Arozullah, B. (2021). Examining the moderating effect of anxiety sensitivity on past-month pain severity and heaviness of smoking among adult smokers experiencing homelessness. Addictive Behaviors, 112. https://doi.org/10.1016/j.addbeh.2020.106610 | Wrong study design |
| Revel M.-P., Abdoul H., Chassagnon G., Canniff E., Durand-Zaleski I., & Wislez M. (2022). Lung CAncer SCreening in French women using low-dose CT and Artificial intelligence for DEtection: The CASCADE study protocol. BMJ Open, 12(12), e067263. https://doi.org/10.1136/bmjopen-2022-067263 | Wrong study design |
| Richardson, S., McNeill, A., & Brose, L. S. (2019). Corrigendum to ‘Smoking and quitting behaviours by mental health conditions in Great Britain (1993-2014)’. Addictive Behaviors, 93(2gw, 7603486), 274. https://doi.org/10.1016/j.addbeh.2019.04.005 | Wrong study design |
| Richmond, R., Indig, D., Butler, T., Wilhelm, K., Archer, V., & Wodak, A. (2013). A randomized controlled trial of a smoking cessation intervention conducted among prisoners. Addiction, 108(5), 966–974. https://doi.org/10.1111/add.12084 | Mental health measured at baseline only, no follow up |
| Richter, A., Sason, A., Adelson, M., Frish, O., & Peles, E. (2020). Cognitive state, substance use patterns and outcome after discharge from Kfar Izun, a unique rehabilitation facility. Journal of Addictive Diseases, 38(4), 387–399. https://doi.org/10.1080/10550887.2020.1773730 | Wrong study design |
| Riley, E. D., Delucchi, K., Rubin, S., Weiser, S. D., Vijayaraghavan, M., Lynch, K., Tsoh, J. Y., & al’Absi, A. (2022). Ongoing tobacco use in women who experience homelessness and unstable housing: A prospective study to inform tobacco cessation interventions and policies. Addictive Behaviors, 125. https://doi.org/10.1016/j.addbeh.2021.107125 | Wrong study design |
| Robinson CD, Rogers CR, & Okuyemi KS. (2016). Depression symptoms among homeless smokers: Effect of motivational interviewing. Substance Use & Misuse, 51(10), 1393–1397. https://doi.org/10.3109/10826084.2016.1170143 | Mental health not measured before quit attempt |
| Robinson, C. D., Wiseman, K. P., Webb Hooper, M., El-Toukhy, S., Grenen, E., Vercammen, L., Prutzman, Y. M., & Cheng, C. (2020). Engagement and short-term abstinence outcomes among Blacks and Whites in the National Cancer Institute’s SmokefreeTXT program. Nicotine & Tobacco Research, 22(9), 1622–1626. https://doi.org/10.1093/ntr/ntz178 | Wrong outcome |
| Robinson, J. D., Cui, Y., Linares Abrego, P., Engelmann, J. M., Prokhorov, A. V., Vidrine, D. J., Shete, S., Cinciripini, P. M., & Attwood, B. (2022). Sustained reduction of attentional bias to smoking cues by smartphone-delivered attentional bias modification training for smokers. Psychology of Addictive Behaviors. https://doi.org/10.1037/adb0000805 | Wrong outcome |
| Robinson, J. D., Karam-Hage, M., Kypriotakis, G., Beneventi, D., Blalock, J. A., Cui, Y., Gonzalez, R., Tayar, J., Chaftari, P., & Cinciripini, P. M. (2022). Bupropion xl and sr have similar effectiveness and adverse event profiles when used to treat smoking among patients at a comprehensive cancer center. The American Journal on Addictions, No-Specified. https://doi.org/10.1111/ajad.13282 | Mental health measured at baseline only, no follow up |
| Robinson, J. D., Kypriotakis, G., Al’absi, M., Denlinger-Apte, R. L., Drobes, D. J., Leischow, S. J., McClernon, F. J., Pacek, L. R., Severson, H. H., Smith, T. T., Donny, E. C., Luo, X., Jensen, J. A., Strayer, L. G., Cinciripini, P. M., Hatsukami, D. K., & Baker, B. (2020). Very low nicotine content cigarettes disrupt the feedback loop of affective states and smoking behavior. Nicotine & Tobacco Research, 22(8), 1294–1300. https://doi.org/10.1093/ntr/ntz209 | Wrong outcome |
| Robinson, J. D., Li, L., Chen, M., Lerman, C., Tyndale, R. F., Schnoll, R. A., Hawk, L. W. Jr., George, T. P., Benowitz, N. L., Cinciripini, P. M., & Aguirre, A. (2019). Evaluating the temporal relationships between withdrawal symptoms and smoking relapse. Psychology of Addictive Behaviors, 33(2), 105–116. https://doi.org/10.1037/adb0000434 | Wrong outcome |
| Robles, Z., Anjum, S., Garey, L., Kauffman, B. Y., Rodriguez-Cano, R., Langdon, K. J., Neighbors, C., Reitzel, L. R., & Zvolensky, M. J. (2017). Financial strain and cognitive-based smoking processes: The explanatory role of depressive symptoms among adult daily smokers. Addictive Behaviors, 70, 18–22. https://doi.org/10.1016/j.addbeh.2017.01.034 | Mental health measured at baseline only, no follow up |
| Robson, E. K., Kamper, S. J., Davidson, S., Viana da Silva, P., Williams, A., Hodder, R. K., Lee, H., Hall, A., Gleadhill, C., & Williams, C. M. (2019). Healthy Lifestyle Program (HeLP) for low back pain: Protocol for a randomised controlled trial. BMJ Open, 9(9), e029290. https://doi.org/10.1136/bmjopen-2019-029290 | Wrong outcome |
| Rocha, V., Guerra, M. P., Lemos, M. S., Maciel, J., & Williams, G. C. (2017). Smoking abstinence twelve months after an acute coronary syndrome. *Spanish Journal of Psychology*, *20*, E63. | Wrong exposure |
| Rodgers, J., Friede, T., Vonberg, F. W., Constantinescu, C. S., Coles, A., Chataway, J., Duddy, M., Emsley, H., Ford, H., Fisniku, L., Galea, I., Harrower, T., Hobart, J., Huseyin, H., Kipps, C. M., Marta, M., McDonnell, G. V., McLean, B., Pearson, O. R., … Nicholas, R. (2022). The impact of smoking cessation on multiple sclerosis disease progression. Brain : A Journal of Neurology, 145(4), 1368–1378. https://doi.org/10.1093/brain/awab385 | Wrong exposure |
| Rodriguez, D., Goulazian, T., Strasser, A. A., O’Loughlin, J., Dugas, E. N., Kuoiloi, C., Hitsman, B. L., Schnoll, R., & Agaku, A. (2019). The role of the subjective importance of smoking (SIMS) in cessation and abstinence. Journal of Smoking Cessation, 14(1), 1–11. https://doi.org/10.1017/jsc.2018.7 | Wrong study design |
| Rodriguez-Cano R., Robinson J.D., Karam-Hage M., Cinciripini P.M., Zvolensky M.J., & Blalock J.A. (2020). Association of major depressive disorder with smoking abstinence by hazardous drinking among smoking cessation treatment-seekers with cancer. Alcoholism: Clinical and Experimental Research, 44(Supplement 1), 162A. https://doi.org/10.1111/acer.14356 | Wrong outcome |
| Rodriguez-Cano, R., Lopez-Duran, A., Martinez-Vispo, C., Becona, E., & Ameringer, A. (2021). Causes of smoking relapse in the 12 months after smoking cessation treatment: Affective and cigarette dependence-related factors. Addictive Behaviors, 119. https://doi.org/10.1016/j.addbeh.2021.106903 | Wrong outcome |
| Rodriguez-Cano, R., Paulus, D. J., Zvolensky, M. J., Lopez-Duran, A., Martinez-Vispo, C., & Becona, E. (2018). Depressive symptoms in the trajectory of craving during smoking cessation treatment: A latent growth curve model. American Journal of Drug and Alcohol Abuse, 44(4), 472–479. https://doi.org/10.1080/00952990.2018.1423687 | Did not analyse mental health outcomes by exposure of interest |
| Roelsgaard, I. K., Ikdahl, E., Rollefstad, S., Wibetoe, G., Esbensen, B. A., Kitas, G. D., van Riel, P., Gabriel, S., Kvien, T. K., Douglas, K., Wallberg-Jonsson, S., Rantapaa Dahlqvist, S., Karpouzas, G., Dessein, P. H., Tsang, L., El-Gabalawy, H., Hitchon, C. A., Pascual-Ramos, V., Contreras-Yanez, I., … Semb, A. G. (2020). Smoking cessation is associated with lower disease activity and predicts cardiovascular risk reduction in rheumatoid arthritis patients. Rheumatology (Oxford, England), 59(8), 1997–2004. https://doi.org/10.1093/rheumatology/kez557 | Wrong study design |
| Rogers, A. H., Bakhshaie, J., Garey, L., Piasecki, T. M., Gallagher, M. W., Schmidt, N. B., Zvolensky, M. J., & Aguirre, A. (2019). Individual differences in emotion dysregulation and trajectory of withdrawal symptoms during a quit attempt among treatment-seeking smokers. Behaviour Research and Therapy, 115, 4–11. https://doi.org/10.1016/j.brat.2018.10.007 | Wrong outcome |
| Rohsenow DJ, Tidey JW, Martin RA, Colby SM, Swift RM, Leggio L, & Monti PM. (2017). Varenicline versus nicotine patch with brief advice for smokers with substance use disorders with or without depression: Effects on smoking, substance use and depressive symptoms. Addiction (Abingdon, England), 112(10), 1808–1820. https://doi.org/10.1111/add.13861 | Did not analyse mental health outcomes by exposure of interest |
| Romm, K. F., Wang, Y., Duan, Z., Bennett, B., Fuss, C., Ma, Y., Blank, M. D., Bray, B. C., Ahluwalia, J. S., & Berg, C. J. (2022). Psychosocial predictors of longitudinal changes in tobacco and cannabis use among young adults. Addictive Behaviors, 129(2gw, 7603486), 107264. https://doi.org/10.1016/j.addbeh.2022.107264 | Wrong study design |
| Rosen, R. L., Borges, A. M., Kibbey, M. M., Steinberg, M. L., Leyro, T. M., Farris, S. G., & Abrams, A. (2019). Distress intolerance and withdrawal severity among daily smokers: The role of smoking abstinence expectancies. Addictive Behaviors, 99. https://doi.org/10.1016/j.addbeh.2019.106048 | Wrong study design |
| Roson NF, Panadero-Paz C, Almadana-Pacheco V, Benito-Bernaldez C, Rodriguez-Martin PJ, & Montemayor-Rubio T. (2017). Influence of psychiatric disorders in patients treated with varenicline. European Respiratory Journal, 50. https://doi.org/10.1183/1393003.congress-2017.PA4478 | Did not analyse mental health outcomes by exposure of interest |
| Rubenstein, D., Aston, E. R., Nollen, N. L., Mayo, M. S., Brown, A. R., & Ahluwalia, J. S. (2020). Factors Associated With Cannabis Use Among African American Nondaily Smokers. Journal of Addiction Medicine, 14(5), e170–e174. https://doi.org/10.1097/ADM.0000000000000652 | Wrong study design |
| Ruokolainen, O., Harkanen, T., Lahti, J., Haukkala, A., Heliovaara, M., & Rahkonen, O. (2021). Association between educational level and smoking cessation in an 11-year follow-up study of a national health survey. Scandinavian Journal of Public Health, 49(8), 951–960. https://doi.org/10.1177/1403494821993721 | Wrong exposure |
| Ruppert A.M. & Urban T. (2019). Le sevrage tabagique dans le cancer bronchique: Les methodes et les resultats; place de la e-cigarette. Revue Des Maladies Respiratoires Actualites, 11(3), 177–183. https://doi.org/10.1016/S1877-1203%2819%2930084-9 | Wrong study design |
| Russell, C., Haseen, F., McKeganey, N., & Babb, B. (2019). Factors associated with past 30-day abstinence from cigarette smoking in adult established smokers who used a JUUL vaporizer for 6 months. Harm Reduction Journal, 16. https://doi.org/10.1186/s12954-019-0331-5 | Wrong outcome |
| Russo, C., Walicka, M., Caponnetto, P., Cibella, F., Maglia, M., Alamo, A., Campagna, D., Frittitta, L., Di Mauro, M., Caci, G., Krysinski, A., Franek, E., & Polosa, R. (2022). Efficacy and Safety of Varenicline for Smoking Cessation in Patients With Type 2 Diabetes: A Randomized Clinical Trial. JAMA Network Open, 5(6), e2217709. https://doi.org/10.1001/jamanetworkopen.2022.17709 | Wrong study design |
| Sabbagh, H. J., Abdelaziz, W., Quritum, M., AlKhateeb, N. A., Abourdan, J., Qureshi, N., Qureshi, S., Hamoud, A. H. N., Mahmoud, N., Odeh, R., Al-Khanati, N. M., Jaber, R., Balkhoyor, A. L., Shabi, M., Folayan, M. O., Alade, O., Gomaa, N., Alnahdi, R., Mahmoud, N. A., … El Tantawi, M. (2022). Cigarettes’ use and capabilities-opportunities-motivation-for-behavior model: A multi-country survey of adolescents and young adults. Frontiers in Public Health, 10(101616579), 875801. https://doi.org/10.3389/fpubh.2022.875801 | Wrong study design |
| Sachs-Ericsson, N., Schmidt, N., Zvolensky, M., Mitchell, M., Rushing, N., & Blazer, D. (2009). Smoking cessation behavior in older adults by race and gender: The role of health problems and psychological distress. Nicotine & Tobacco Research : Official Journal of the Society for Research on Nicotine and Tobacco, 11, 433–443. https://doi.org/10.1093/ntr/ntp002 | Did not distinguish between unsuccessful quit attempt and no attempt to quit |
| Saeed, M. I., Sivapalan, P., Eklof, J., Ulrik, C. S., Pisinger, C., Lapperre, T., Tonnesen, P., Hoyer, N., Janner, J., Karlsson, M. L., Bech, C. S., Marsa, K., Godtfredsen, N., Brondum, E., Munk, B., Raaschou, M., Browatzski, A., Lutken, P., & Jensen, J.-U. S. (2020). TOB-STOP-COP (TOBacco STOP in COPd trial): Study protocol-a randomized open-label, superiority, multicenter, two-arm intervention study of the effect of ‘high-intensity’ vs. ‘low-intensity’ smoking cessation intervention in active smokers with chronic obstructive pulmonary disease. Trials, 21(1), 730. https://doi.org/10.1186/s13063-020-04653-z | Wrong study design |
| Saeidi, M., Komasi, S., Heydarpour, B., Karim, H., Nalini, M., & Ezzati, P. (2016). Predictors of clinical anxiety aggravation at the end of a cardiac rehabilitation program. Research in Cardiovascular Medicine, 5(1). https://doi.org/10.5812/cardiovascmed.30091 | Wrong exposure |
| Sagayadevan V., Abdin E., Shahwan S., Satghare P., Devi F., Cetty L., Sendren J.R., Verma S.K., Chong S.A., & Subramaniam M. (2019). Motivations to quit smoking and challenges faced during cessation among individuals with first episode psychosis in Singapore. Early Intervention in Psychiatry, 13(6), 1488–1494. https://doi.org/10.1111/eip.12799 | Wrong outcome |
| Sahin, H., & Naz, I. (2021). The effect of pulmonary rehabilitation on smoking and health outcomes in COPD patients. The Clinical Respiratory Journal, 15(8), 855–862. https://doi.org/10.1111/crj.13373 | Wrong study design |
| Saiz Martinez PA, Al-Halabi S, Fernandez-Artamendi S, Garcia-Alvarez L, Diaz-Mesa E, Martinez-Santamaria E, Florez G, Arrojo M, Garcia-Portilla MP, & Bobes J. (2016). Effects of nicotine abstinence on clinical symptoms. Study at 3 and 6-months follow-up of outpatients with schizophrenia. *European Psychiatry*, *Conference: 24th European Congress of Psychiatry, EPA 2016 Madrid Spain. Conference Start: 20160312 Conference End: 20160315. Conference Publication:*, S261–S262. | Did not analyse mental health outcomes by exposure of interest |
| Salameh, T. N., Hall, L. A., Hall, M. T., & Crawford, T. N. (2022). Cigarette smoking cessation and mental health treatment receipt in a U.S national sample of pregnant women with mental illness. Journal of Nursing Scholarship : An Official Publication of Sigma Theta Tau International Honor Society of Nursing, 54(2), 202–212. https://doi.org/10.1111/jnu.12731 | Wrong study design |
| Salas, J., Gillis, A., Schneider, D., Scherrer, J., van den Berk-Clark, C., Tuerk, P., Gebauer, S., Schnurr, P., & Lustman, P. (2022). PTSD symptom improvement and smoking cessation among a sample of veterans. Annals of Family Medicine, 20 Suppl 1. https://doi.org/10.1370/afm.20.s1.2604 | Wrong study design |
| Salgado Garcia F.I., Derefinko K.J., Bursac Z., Klesges R.C., Ebbert J.O., Womack C.R., & Krukowski R.A. (2019). Fit & quit: An efficacy trial of two behavioral post-cessation weight gain interventions. *Contemporary Clinical Trials, 76*, 31–40. https://doi.org/10.1016/j.cct.2018.11.009 | Wrong study design |
| Salk, R. H., Germeroth, L. J., Emery, R. L., Conlon, R. P. K., Wang, Z., Cheng, Y., Marcus, M. D., Perkins, K. A., Levine, M. D., & Beck, B. (2019). Predictive utility of subtyping women smokers on depression, eating, and weight-related symptoms. Health Psychology, 38(3), 248–258. https://doi.org/10.1037/hea0000702 | Mental health measured at baseline only, no follow up |
| Salman, A., & Doherty, P. (2020). Is Weight Gain Inevitable for Patients Trying to Quit Smoking as Part of Cardiac Rehabilitation?. International Journal of Environmental Research and Public Health, 17(22). https://doi.org/10.3390/ijerph17228565 | Wrong study design |
| Samaan, J. S., Mohan, S., Toubat, O., Qian, E., Lee, N., Subramanyam, C., Alicuben, E. T., Dobrowolsky, A., Sandhu, K., & Samakar, K. (2021). Effects of smoking on bariatric surgery postoperative weight loss and patient satisfaction. Surgical Endoscopy, 35(7), 3584–3591. https://doi.org/10.1007/s00464-020-07827-7 | Wrong outcome |
| Sancho-Domingo, C., Carballo, J. L., Coloma-Carmona, A., van-der Hofstadt Roman, C., Asensio Sanchez, S., & Acquadro, A.-M. (2022). Psychometric validity of the minnesota tobacco withdrawal scale in smokers attempting to quit: Longitudinal invariance across smokers and abstainers. *Journal of Psychoactive Drugs, 55*(4), 493-500. https://doi.org/10.1080/02791072.2022.2113483 | Did not analyse mental health outcomes by exposure of interest |
| Sancho-Domingo, C., Carballo, J. L., Coloma-Carmona, A., van der Hofstadt, C., Garcia del Castillo-Lopez, A., & Asensio Sanchez, S. (2024). Effectiveness of the brief guided self-change therapy combined with varenicline under ‘real-life’ conditions and mediators for smoking cessation. *Substance Use & Misuse*, *59*(1), 110–118. https://doi.org/10.1080/10826084.2023.2262021 | Mental health measured at baseline only, no follow up |
| Sanford, B. T., Toll, B. A., Palmer, A. M., Foster, M. G., Cummings, K. M., Stansell, S., & Rojewski, A. M. (2022). Tobacco Treatment Outcomes for Hospital Patients With and Without Mental Health Diagnoses. Frontiers in Psychiatry, 13(101545006), 853001. https://doi.org/10.3389/fpsyt.2022.853001 | Wrong study design |
| Santi S.A., Conlon M.S.C., Meigs M.L., Davidson S.M., Mispel-Beyer K., & Saunders D.P. (2022). Rates of Smoking Cessation at 6 and 12 Months after a Clinical Tobacco Smoking Cessation Intervention in Head and Neck Cancer Patients in Northern Ontario, Canada. Current Oncology, 29(3), 1544–1558. https://doi.org/10.3390/curroncol29030130 | Wrong study design |
| Santiago-Torres, M., Mull, K. E., Sullivan, B. M., Kwon, D. M., Nez Henderson, P., Nelson, L. A., Patten, C. A., & Bricker, J. B. (2022). Efficacy and Utilization of Smartphone Applications for Smoking Cessation among American Indians and Alaska Natives: Results from the iCanQuit Trial. *Nicotine and Tobacco Research, 24*(4), 544–554. https://doi.org/10.1093/ntr/ntab213 | Mental health measured at baseline only, no follow up |
| Santiago-Torres M., Mull K.E., Sullivan B.M., Zvolensky M.J., Kahler C.W., & Bricker J.B. (2022). Efficacy of smartphone applications for smoking cessation in heavy-drinking adults: Secondary analysis of the iCanQuit randomized trial. *Addictive Behaviors, 132*, 107377. https://doi.org/10.1016/j.addbeh.2022.107377 | Wrong study design |
| Santiago-Torres, M., Mull, K. E., Sullivan, B. M., Ferketich, A. K., Bricker, J. B., & Adibe, B. (2022). Efficacy of an acceptance and commitment therapy-based smartphone application for helping rural populations quit smoking: Results from the iCanQuit randomized trial. *Preventive Medicine, 157,* 107008. https://doi.org/10.1016/j.ypmed.2022.107008 | Wrong study design |
| Santiago-Torres, M., Mull, K. E., Sullivan, B. M., Prochaska, J. J., Zvolensky, M. J., & Bricker, J. B. (2024). Can an Acceptance and Commitment Therapy-Based Smartphone App Help Individuals with Mental Health Disorders Quit Smoking? *Depression and Anxiety*, 1055801. https://doi.org/10.1155/2024/1055801 | Mental health measured at baseline only, no follow up |
| Santus, P., Radovanovic, D., Raiteri, D., Pini, S., Spagnolo, G., Maconi, G., & Rizzi, M. (2020). The effect of a multidisciplinary approach for smoking cessation in patients with Crohn’s disease: Results from an observational cohort study. *Tobacco Induced Diseases, 18*, 29. https://doi.org/10.18332/tid/119161 | Mental health measured at baseline only, no follow up |
| Sargent, J. D., Halenar, M. J., Edwards, K. C., Woloshin, S., Schwartz, L., Emond, J., Tanski, S., Taylor, K. A., Pierce, J. P., Liu, J., Goniewicz, M. L., Niaura, R., Anic, G., Chen, Y., Callahan-Lyon, P., Gardner, L. D., Thekkudan, T., Borek, N., Kimmel, H. L., … Brunette, M. (2022). Tobacco Use and Respiratory Symptoms Among Adults: Findings From the Longitudinal Population Assessment of Tobacco and Health (PATH) Study 2014-2016. *Nicotine & Tobacco Research, 24*(10), 1607–1618. https://doi.org/10.1093/ntr/ntac080 | Wrong exposure |
| Sarramea, F., Jaen-Moreno, M. J., Balanza-Martinez, V., Osuna, M. I., Alcala, J. A., Montiel, F. J., Gomez, C., Sanchez, M. D., Rico, A. B., Redondo-Ecija, J., Gil, S., Valdivia, F., Caballero-Villarraso, J., Gutierrez-Rojas, L., & Aharonovich, A.-H. (2019). Setting the stage to quit smoking in Bipolar Disorder patients: Brief advice in clinical practice. *Adicciones, 31*(2), 136–145. https://doi.org/10.20882/adicciones.1006 | Wrong study design |
| Sarwar, M. R., McDonald, V. M., Abramson, M. J., Wilson, S., Holland, A. E., Bonevski, B., Mahal, A., Paul, E., Meier, B., & George J. (2024). Credentialed pharmacist-led home medicines reviews targeting treatable traits and their impact on health outcomes in people with chronic obstructive pulmonary disease: A pre- and post-intervention study. *International Journal of Clinical Pharmacy*. https://doi.org/10.1007/s11096-024-01819-6 | Did not analyse mental health outcomes by exposure of interest |
| Sathya S.G., Zaw C., & Holt G.E. (2022). OA10.06 An Unconventional Financial Incentive Based Feasibility Trial for Smoking Cessation. Journal of Thoracic Oncology, 17(9 Supplement), S29. https://doi.org/10.1016/j.jtho.2022.07.055 | Wrong study design |
| Savoy E.J., Businelle M.S., Nguyen N., Chen T.-A., Neighbors C., Norton P.J., Taing M., & Reitzel L.R. (2021). Examining moment to moment affective determinants of smoking rate following a quit attempt among homeless daily smokers. Addictive Behaviors, 115, 106788. https://doi.org/10.1016/j.addbeh.2020.106788 | Wrong study design |
| Scarinci, I. C., Kienen, N., Wiltenburg, T. D., Bittencourt, L., & Person, S. D. (2022). Efficacy of a Gender-Relevant Smoking Cessation Intervention Among Women in Brazil: Findings from a Group Randomized Controlled Trial. Journal of Women’s Health (2002), 31(11), 1620–1629. https://doi.org/10.1089/jwh.2021.0443 | Wrong study design |
| Scheenstra, B., Mohansingh, C., Bongers, B. C., Dahmen, S., Wouters, Y. I. M. S., Lenssen, T. F., Geerlings, P., Knols, H. F. M., van Kuijk, S. M. J., Kimman, M. L., Nieman, M., Maessen, J. G., Van’t Hof, A. W. J., & Peyman, S. N. (2021). Personalized teleprehabilitation in elective cardiac surgery: A study protocol of the Digital Cardiac Counselling randomized controlled trial. European Heart Journal. Digital Health, 2(3), 477–486. https://doi.org/10.1093/ehjdh/ztab041 | Did not analyse mental health outcomes by exposure of interest |
| Scheuermann, T. S., Saint Onge, J. M., Ramaswamy, M., Cox, L. S., Ahluwalia, J. S., Nollen, N. L., & Almedom, A. (2020). The role of neighborhood experiences in psychological distress among African American and white smokers. Race and Social Problems, 12(2), 133–144. https://doi.org/10.1007/s12552-020-09281-5 | Mental health measured at baseline only, no follow up |
| Schiavon, S., Hodgin, K., Sellers, A., Hendricks, P. S., Gaggar, A., Scarinci, I., Cropsey, K. L., & Binswanger, C. (2019). Differences among cigarette-only smokers compared to dual users of cigarettes and little cigars/cigarillos in the criminal justice population. *Addictive Behaviors, 90.* https://doi.org/10.1016/j.addbeh.2018.11.001 | Wrong exposure |
| Schirmbeck, F., van der Ven, E., Boyette, L.-L., McGuire, P., Valmaggia, L. R., Kempton, M. J., van der Gaag, M., Riecher-Rossler, A., Barrantes-Vidal, N., Nelson, B., Krebs, M.-O., Ruhrmann, S., Sachs, G., Rutten, B. P. F., Nordentoft, M., EU-GEI High Risk Study Group, de Haan, L., Vermeulen, J. M., & Calem M, T. S. (2022). Differential trajectories of tobacco smoking in people at ultra-high risk for psychosis: Associations with clinical outcomes. *Frontiers in Psychiatry, 13*(101545006), 869023. https://doi.org/10.3389/fpsyt.2022.869023 | Wrong study design |
| Schlam, T. R., & Baker, T. B. (2020). Playing Around with Quitting Smoking: A Randomized Pilot Trial of Mobile Games as a Craving Response Strategy. Games for Health Journal, 9(1), 64–70. https://doi.org/10.1089/g4h.2019.0030 | Follow up < 6 weeks |
| Schlam, T. R., Baker, T. B., Smith, S. S., Cook, J. W., Piper, M. E., & Aiken, B. (2020). Anxiety sensitivity and distress tolerance in smokers: Relations with tobacco dependence, withdrawal, and quitting success. Nicotine & Tobacco Research, 22(1), 58–65. https://doi.org/10.1093/ntr/ntz070 | Wrong study design |
| Schmidt NB, Raines AM, Allan NP, & Zvolensky MJ. (2016). Anxiety sensitivity risk reduction in smokers: A randomized control trial examining effects on panic. Behaviour Research and Therapy, 77, 138–146. https://doi.org/10.1016/j.brat.2015.12.011 | Did not analyse mental health outcomes by exposure of interest |
| Schnitzer K., Jones S., Kelley J.H.K., Tindle H.A., Rigotti N.A., & Kruse G.R. (2021). A qualitative study of the impact of covid-19 on smoking behavior for participants in a post-hospitalization smoking cessation trial. International Journal of Environmental Research and Public Health, 18(10), 5404. https://doi.org/10.3390/ijerph18105404 | Wrong study design |
| Schnitzer, K., Senft, N., Tindle, H. A., Kelley, J. H. K., Notier, A. E., Davis, E. M., Rigotti, N. A., Douaihy, A., Levy, D. E., Singer, D. E., Kruse, G., & Adamle, A. (2022). Understanding engagement behaviors and rapport building in tobacco cessation telephone counseling: An analysis of audio-recorded counseling calls. *Journal of Substance Abuse Treatment, 135.* https://doi.org/10.1016/j.jsat.2021.108643 | Mental health measured at baseline only, no follow up |
| Scott, W. D., Beevers, C. G., & Mermelstein, R. J. (2008). Depression vulnerable and nonvulnerable smokers after a failure experience: Examining cognitive self-regulation and motivation. Behav Modif, 32(4), 519–539. https://doi.org/10.1177/0145445507310484 | Wrong outcome |
| Scoville E.A., Tindle H.A., Wells Q.S., Peyton S.C., Gurwara S., Pointer S.O., Horst S.N., Schwartz D.A., Adams D.W., Freiberg M.S., Gatskie V., King S., Abney L.R., & Beaulieu D.B. (2020). Precision nicotine metabolism-informed care for smoking cessation in Crohn’s disease: A pilot study. PLoS ONE, 15(3), e0230656. https://doi.org/10.1371/journal.pone.0230656 | Mental health measured at baseline only, no follow up |
| Seaman, E. L., Robinson, C. D., Crane, D., Taber, J. M., Ferrer, R. A., Harris, P. R., & Klein, W. M. P. (2021). Association of Spontaneous and Induced Self-Affirmation With Smoking Cessation in Users of a Mobile App: Randomized Controlled Trial. Journal of Medical Internet Research, 23(3), e18433. https://doi.org/10.2196/18433 | Wrong study design |
| Segan CJ, Maddox S, & Borland R. (2015). Homeless clients benefit from smoking cessation treatment delivered by a homeless persons’ program. Nicotine & Tobacco Research, 17(8), 996–1001. https://doi.org/10.1093/ntr/ntv062 | Did not analyse mental health outcomes by exposure of interest |
| Segan, C. J., Borland, R., Wilhelm, K. A., Bhar, S. S., Hannan, A. T., Dunt, D. R., & Ferretter, I. T. (2011). Helping smokers with depression to quit smoking: Collaborative care with Quitline. *Medical Journal of Australia*, *195*(3), S7-11. | Did not analyse mental health outcomes by exposure of interest |
| Seidman, D., Westmaas, J., Goldband, S., Rabius, V., Katkin, E., Pike, K. J., Wiatrek, D., & Sloan, R. (2010). Randomized controlled trial of an interactive internet smoking cessation program with long-term follow-up. Annals of Behavioral Medicine : A Publication of the Society of Behavioral Medicine, 39, 48–60. https://doi.org/10.1007/s12160-010-9167-7 | Wrong outcome |
| Seif Rabiei, M. A., Bahrami Mashouf, S., Sanaei, Z., & Albert-Lorincz, B. (2022). Status of social capital and its relationship with the success rate of smoking cessation in patients referred to the smoking cessation clinic of farshchian cardiovascular hospital in hamadan. *Journal of Substance Use*. https://doi.org/10.1080/14659891.2022.2047807 | Wrong study design |
| Seo, Y.-G., Jo, M.-W., Paek, Y.-J., Choi, J., & Caponnetto, C. (2021). Effects of morbidity on smoking cessation: A national smoking cessation program. Addiction Science & Clinical Practice, 16. https://doi.org/10.1186/s13722-021-00257-3 | Mental health measured at baseline only, no follow up |
| Seoudy K., Kypriotakis G., Beneventi D., Blalock J., Minnix J., Robinson J., Cui Y., Cinciripini P., & Hage M.K. (2019). Real-time audio/video versus in-person treatment for smoking. American Journal on Addictions, 28(3), 168. https://doi.org/10.1002/ajad.12887 | Wrong study design |
| Shadel, W. G., & Mermelstein, R. J. (1993). Cigarette smoking under stress: The role of coping expectancies among smokers in a clinic-based smoking cessation program. Health Psychol, 12(6), 443–450. https://doi.org/10.1037//0278-6133.12.6.443 | Wrong outcome |
| Shafie-Khorassani, F., Piper, M. E., Jorenby, D. E., Baker, T. B., Benowitz, N. L., Hayes-Birchler, T., Meza, R., & Brouwer, A. F. (2022). Associations of demographics, dependence, and biomarkers with transitions in tobacco product use in a cohort of cigarette users and dual users of cigarettes and e-cigarettes. Nicotine & Tobacco Research, drz, 9815751. https://doi.org/10.1093/ntr/ntac207 | Wrong outcome |
| Shahab, L., Andrew, S., & West, R. (2014). Changes in prevalence of depression and anxiety following smoking cessation: Results from an international cohort study (ATTEMPT). *Psychological Medicine*, *44*(1), 127–141. | Wrong outcome |
| Shahab, L., Gilchrist, G., Hagger-Johnson, G., Shankar, A., West, E., & West, R. (2015). Reciprocal associations between smoking cessation and depression in older smokers: Findings from the English Longitudinal Study of Ageing. British Journal of Psychiatry, 207(3), 243–249. https://doi.org/10.1192/bjp.bp.114.153494 | Wrong exposure |
| Sharath S.E., Lee M., Kougias P., Taylor W.C., Zamani N., & Barshes N.R. (2019). Successful Smoking Cessation Associated with Walking Behavior in Patients with Claudication. Annals of Vascular Surgery, 56, 287–293. https://doi.org/10.1016/j.avsg.2018.09.017 | Wrong outcome |
| Shariati, H., Armstrong, H. L., Cui, Z., Lachowsky, N. J., Zhu, J., Anand, P., Roth, E. A., Hogg, R. S., Oudman, G., Tonella, C., & Moore, D. M. (2017). Changes in smoking status among a longitudinal cohort of gay, bisexual, and other men who have sex with men in Vancouver, Canada. Drug and Alcohol Dependence, 179, 370–378. https://doi.org/10.1016/j.drugalcdep.2017.07.025 | Mental health not measured before quit attempt |
| Sheffer C., Bickel W., Mahoney M., Hanlon C., Shevorykin A., & Carl E. (2021). High Frequency rTMS Dosing for Smoking Cessation: Preliminary Findings. Brain Stimulation, 14(6), 1732. https://doi.org/10.1016/j.brs.2021.10.478 | Wrong study design |
| Sheffer C., Carl E., & Shevorykin A. (2021). rTMS Dosing for Smoking Cessation: Pre-COVID Preliminary Results. Brain Stimulation, 14(6), 1611. https://doi.org/10.1016/j.brs.2021.10.073 | Wrong study design |
| Shevorykin A., Ruglass L.M., Mancini A.D., Carl E., Legg A., & Sheffer C.E. (2021). Exploring the Role of Traumatic Event Exposure in Tobacco Dependence Treatment Outcomes Among African Americans. Journal of Psychoactive Drugs, 53(5), 452–459. https://doi.org/10.1080/02791072.2021.1985661 | Wrong study design |
| Shi, Y., Hooten, W. M., & Warner, D. O. (2011). Effects of smoking cessation on pain in older adults. *Nicotine & Tobacco Research*, *13*(10), 919–925. | Did not distinguish between unsuccessful quit attempt and no attempt to quit |
| Shiffman, S. (2005). Dynamic influences on smoking relapse process. Journal of Personality, 73(6), 1715–1748. https://doi.org/10.1111/j.0022-3506.2005.00364.x | Did not analyse mental health outcomes by exposure of interest |
| Shiffman, S., & Waters, A. J. (2004). Negative affect and smoking lapses: A prospective analysis. Journal of Consulting and Clinical Psychology, 72(2), 192–201. https://doi.org/10.1037/0022-006X.72.2.192 | Wrong outcome |
| Shimadu, S., Hamajima, N., Okada, Y., Oguri, T., Murohara, T., Ban, N., Sato, M., & Hasegawa, Y. (2016). Factors influencing sustainable efficacy of smoking cessation treatment with varenicline beyond nine months. *Nagoya Journal of Medical Science*, *78*(2), 205–213. | Mental health measured at baseline only, no follow up |
| Shoorijeh, F. T., Palenik, C. J., & Askarian, M. (2019). Effect of a Smoking Cessation Program on Inpatients in the Largest Hospital in Southern Iran. International Journal of Preventive Medicine, 10(101535380), 54. https://doi.org/10.4103/ijpvm.IJPVM_57_17 | Wrong study design |
| Shuter, J., Hosgood, H. D., Kim, R. S., Ye, K., Montagna, C., Shan, J., & Weinberger, A. H. (2021). Behavioral and Genetic Factors Associated with Successful Long-Term Cessation in Persons with HIV Who Smoke Cigarettes. Journal of Smoking Cessation, 2021(101478447), 1894160. https://doi.org/10.1155/2021/1894160 | Wrong outcome |
| Siddiqi, K., Keding, A., Marshall, A.-M., Dogar, O., Li, J., Huque, R., Fatima, R., Khan, A., Elsey, H., Gabe, R., Kotz, D., & Sheikh, A. (2022). Effect of quitting smoking on health outcomes during treatment for tuberculosis: Secondary analysis of the TB & Tobacco Trial. Thorax, 77(1), 74–78. https://doi.org/10.1136/thoraxjnl-2020-215926 | Wrong study design |
| Siddiqi, K., Siddiqui, F., Khan, A., Ansaari, S., Kanaan, M., Khokhar, M., Islam, Z., Mishu, M. P., & Bauld, L. (2021). The Impact of COVID-19 on Smoking Patterns in Pakistan: Findings From a Longitudinal Survey of Smokers. Nicotine & Tobacco Research : Official Journal of the Society for Research on Nicotine and Tobacco, 23(4), 765–769. https://doi.org/10.1093/ntr/ntaa207 | Not adult population |
| Siddiqui F., Bauld L., Croucher R., Jackson C., Kellar I., Kanaan M., Pokhrel S., Huque R., Iqbal R., Khan J.A., Mehrotra R., & Siddiqi K. (2022). Behavioural support and nicotine replacement therapy for smokeless tobacco cessation: Protocol for a pilot randomised-controlled multi-country trial. Pilot and Feasibility Studies, 8(1), 189. https://doi.org/10.1186/s40814-022-01146-5 | Wrong study design |
| Silva A.L.R.C.E., Matias M.V., Mestre A.M., Carvalho J., Santos A.F., Matos C., & Nogueira F. (2020). Cigarette smoking and the female gender: What has changed in 10 years? European Respiratory Journal, 56(Supplement 64). https://doi.org/10.1183/13993003.congress-2020.1386 | Wrong study design |
| Silva-Jose, C., Sanchez-Polan, M., Barakat, R., Diaz-Blanco, A., Mottola, M. F., & Refoyo, I. (2022). A Virtual Exercise Program throughout Pregnancy during the COVID-19 Pandemic Modifies Maternal Weight Gain, Smoking Habits and Birth Weight-Randomized Clinical Trial. Journal of Clinical Medicine, 11(14). https://doi.org/10.3390/jcm11144045 | Wrong exposure |
| Singh M., Srivastava G.N., & Yadav D. (2019). Effect of non-pharmacological interventions on treatment outcomes in COPD patients: Little changes for bigger rewards. European Respiratory Journal, 54(Supplement 63). https://doi.org/10.1183/13993003.congress-2019.PA722 | Wrong study design |
| Singh P.N., Moses O., Shih W., & Hubbard M. (2022). Cohort profile for the Loma Linda University Health BREATHE programme: A model to study continuously incentivised employee smoking cessation. BMJ Open, 12(4), e053303. https://doi.org/10.1136/bmjopen-2021-053303 | Wrong study design |
| Sinnathamby, H., Robinson, F., Ping, N. P. T., Kean, L. E., Joe, O. S., Suraya, A., Selvaraj, J., Qi, L. J., Xin, L. Y., & Alagasan, V. (2023). Mindfulness-based therapy for smoking cessation and mental health: A randomised controlled trial. *Medical Journal of Malaysia*, *78*(3), 336-343. | Did not analyse mental health outcomes by exposure of interest |
| Smith RC, Amiaz R, Si TM, Maayan L, Jin H, Boules S, Sershen H, Li C, Ren J, Liu Y, Youseff M, Lajtha A, Guidotti A, Weiser M, & Davis JM. (2016). Varenicline effects on smoking, cognition, and psychiatric symptoms in schizophrenia: A double-blind randomized trial. PloS One, 11(1), e0143490. https://doi.org/10.1371/journal.pone.0143490 | Did not analyse mental health outcomes by exposure of interest |
| Smith, P. M., Seamark, L. D., & Beck, K. (2020). Integration of an evidence-based tobacco cessation program into a substance use disorders program to enhance equity of treatment access for northern, rural, and remote communities. Translational Behavioral Medicine, 10(3), 555–564. https://doi.org/10.1093/tbm/ibz162 | Wrong study design |
| Smits, J. A. J., Otto, M. W., Powers, M. B., Baird, S. 0, & Anestis, B. (2019). Anxiety sensitivity as a transdiagnostic treatment target. *The Clinician’s Guide to Anxiety Sensitivity Treatment and Assessment.* https://doi.org/10.1016/B978-0-12-813495-5.00001-2 | Not primary research |
| Smits, J. A. J., Zvolensky, M. J., Otto, M. W., Piper, M. E., Baird, S. O., Kauffman, B. Y., Lee-Furman, E., Alavi, N., Dutcher, C. D., Papini, S., Rosenfield, B., Rosenfield, D., & Abrams, A. (2020). Enhancing panic and smoking reduction treatment with D-cycloserine: A pilot randomized clinical trial. *Drug and Alcohol Dependence, 208.* https://doi.org/10.1016/j.drugalcdep.2020.107877 | Did not analyse mental health outcomes by exposure of interest |
| Smits, J. A. J., Zvolensky, M. J., Rosenfield, D., Brown, R. A., Freeman, S. Z., Dutcher, C. D., Conroy, H. E., & Alavi, N. (2019). YMCA exercise intervention to augment smoking cessation treatment in adults with high anxiety sensitivity: Study protocol for a randomized controlled trial. *Contemporary Clinical Trials, 77*(101242342), 1–7. https://doi.org/10.1016/j.cct.2018.12.001 | Wrong study design |
| Smits, J. A. J., Zvolensky, M. J., Rosenfield, D., Brown, R. A., Otto, M. W., Dutcher, C. D., Papini, S., Freeman, S. Z., DiVita, A., Perrone, A., Garey, L., & Allan, A. (2021). Community-based smoking cessation treatment for adults with high anxiety sensitivity: A randomized clinical trial. *Addiction, 116*(11), 3188–3197. https://doi.org/10.1111/add.15586 | Wrong study design |
| Snaterse M., Jorstad H.T., Minneboo M., Lachman S., Boekholdt S.M., ter Riet G., Scholte op Reimer W.J.M., & Peters R.J.G. (2019). Smoking cessation after nurse-coordinated referral to a comprehensive lifestyle programme in patients with coronary artery disease: A substudy of the RESPONSE-2 trial. *European Journal of Cardiovascular Nursing, 18*(2), 113–121. https://doi.org/10.1177/1474515118795722 | Mental health measured at baseline only, no follow up |
| Snell, M., Harless, D., Shin, S., Cunningham, P., Barnes, A., & Aditya, B. (2021). A longitudinal assessment of nicotine dependence, mental health, and attempts to quit smoking: Evidence from waves 1-4 of the Population Assessment of Tobacco and Health (PATH) study. *Addictive Behaviors, 115*. https://doi.org/10.1016/j.addbeh.2020.106787 | Wrong study design |
| Sohanpal, R., Jumbe, S., James, W.-Y., Steed, L., Yau, T., Rivas, C., Madurasinghe, V., Houlihan, C., Berdunisov, V., Taylor, M., Taylor, S. J. C., Griffiths, C., Eldridge, S., & Walton, R. (2019). Evaluating the effectiveness and cost-effectiveness of the Smoking Treatment Optimisation in Pharmacies (STOP) intervention: Protocol for a cluster randomised controlled trial. *Trials, 20*(1), 337. https://doi.org/10.1186/s13063-019-3368-6 | Wrong study design |
| Sohlberg T. & Wennberg P. (2020). Snus cessation patterns—A long-term follow-up of snus users in Sweden. *Harm Reduction Journal, 17*(1), 62. https://doi.org/10.1186/s12954-020-00405-z | Wrong study design |
| Sokolovsky A.W., Hertel A.W., Micalizzi L., White H.R., Hayes K.L., & Jackson K.M. (2021). Preliminary impact of the COVID-19 pandemic on smoking and vaping in college students. *Addictive Behaviors, 115*, 106783. https://doi.org/10.1016/j.addbeh.2020.106783 | Wrong study design |
| Song, F., Holland, R., Barton, G. R., Bachmann, M., Blyth, A., Maskrey, V., Aveyard, P., Sutton, S., Leonardi-Bee, J., & Brandon, T. H. (2012). Self-help materials for the prevention of smoking relapse: Study protocol for a randomized controlled trial. *Trials, 13*. https://doi.org/10.1186/1745-6215-13-69 | Mental health not measured before quit attempt |
| Sonneville, B., Salhi, B., Van Damme, G., Derom, E., & Van Meerbeeck, J. P. (2015). Outpatient smoking cessation in non-healthy smokers. [Dutch]. *Tijdschrift Voor Geneeskunde, 71*(7), 470–477. https://doi.org/10.2143/TVG.71.07.2001835 | Wrong outcome |
| Sorensen M.K., Rasmussen M., Hjorth P., & Christiansen R. (2022). Success rates of smoking cessation therapies to patients with mental illness by video consultants or by treatment in the community: A Randomized Controlled Trial. *European Psychiatry, 65*(Supplement 1), S178. https://doi.org/10.1192/j.eurpsy.2022.471 | Wrong study design |
| Sornpaisarn B., Parvez N., Chatakan W., Thitiprasert W., Precha P., Kongsakol R., Saengow U., & Rehm J. (2022). Methods and factors influencing successful smoking cessation in Thailand: A case-control study among smokers at the community level. *Tobacco Induced Diseases, 20*(July), 67. https://doi.org/10.18332/tid/150345 | Wrong outcome |
| Souto, M. L. S., Carrer, F. C. de A., Braga, M. M., & Pannuti, C. M. (2021). Smoking Cessation therapy is a cost-effective intervention to avoid tooth loss in Brazilian subjects with periodontitis: An economic evaluation. *BMC Oral Health, 21*(1), 616. https://doi.org/10.1186/s12903-021-01932-2 | Wrong study design |
| Spears, C. A., Mhende, J., Hawkins, C., Do, V. V., Hayat, M. J., Eriksen, M. P., Hedeker, D., Abroms, L. C., & Wetter, D. W. (2022). Mindfulness-Based Smoking Cessation Delivered Through Telehealth and Text Messaging for Low-Income Smokers: Protocol for a Randomized Controlled Trial. *JMIR Research Protocols, 11*(8), e35688. https://doi.org/10.2196/35688 | Wrong study design |
| Sprio, A. E., Ciprandi, G., Riccardi, E., Giannoccaro, F., Carriero, V., Bertolini, F., & Ricciardolo, F. L. M. (2020). The influence of smoking on asthma in the real-life. *Respiratory Medicine, 170*(8908438, rme), 106066. https://doi.org/10.1016/j.rmed.2020.106066 | Wrong study design |
| Stansell, S. M. (2020). Treatment interventions for tobacco cessation at an inpatient mental health facility. *Dissertation Abstracts International: Section B: The Sciences and Engineering*, *81*(11-B), No-Specified. | Wrong study design |
| Stanton, C. A., Bansal-Travers, M., Johnson, A. L., Sharma, E., Katz, L., Ambrose, B. K., Silveira, M. L., Day, H., Sargent, J., Borek, N., Compton, W. M., Johnson, S. E., Kimmel, H. L., Kaufman, A. R., Limpert, J., Abrams, D., Cummings, K. M., Goniewicz, M. L., Tanski, S., … Pearson, J. L. (2019). Longitudinal e-Cigarette and Cigarette Use Among US Youth in the PATH Study (2013-2015). *Journal of the National Cancer Institute, 111*(10), 1088–1096. https://doi.org/10.1093/jnci/djz006 | Wrong outcome |
| Steegers-Theunissen, R., Hoek, A., Groen, H., Bos, A., van den Dool, G., Schoonenberg, M., Smeenk, J., Creutzberg, E., Vecht, L., Starmans, L., Laven, J., & Anderson, B. (2020). Pre-conception interventions for subfertile couples undergoing assisted reproductive technology treatment: Modeling analysis. *JMIR mHealth and uHealth, 8*(11). https://doi.org/10.2196/19570 | Wrong study design |
| Stein, J. H., Smith, S. S., Hansen, K. M., Korcarz, C. E., Piper, M. E., Fiore, M. C., & Baker, T. B. (2020). Longitudinal effects of smoking cessation on carotid artery atherosclerosis in contemporary smokers: The Wisconsin Smokers Health Study. *Atherosclerosis, 315*(95x, 0242543), 62–67. https://doi.org/10.1016/j.atherosclerosis.2020.11.010 | Wrong study design |
| Stein, M. D., Weinstock, M. C., Anderson, B. J., & Anthony, J. L. (2007). Relationship of depression to smoking outcomes in a methadone-maintained population. *J Addict Dis, 26*(1), 35–40. https://doi.org/10.1300/J069v26n01_05 | Mental health measured at baseline only, no follow up |
| Steinberg, M. L., Williams, J. M., Stahl, N. F., Budsock, P. D., & Cooperman, N. A. (2016). An adaptation of motivational interviewing increases quit attempts in smokers with serious mental illness. *Nicotine and Tobacco Research, 18*(3), 243–250. https://doi.org/10.1093/ntr/ntv043 | Follow up < 6 weeks |
| Stepankova, L., Kralikova, E., Zvolska, K., Pankova, A., Adamcekova, Z., Kuhn, M., Noland, D., & Annemans, B. (2021). Comparison between success rates for smokers re-treated by a smokers’ clinic and success rates for smokers treated for the first time. *Addiction, 116*(2), 346–355. https://doi.org/10.1111/add.15175 | Wrong study design |
| Stepankova, L., Kralikova, E., Zvolska, K., Pankova, A., Felbrova, V., & Kulovana, S. (2016). Improvement of depressive symptoms after a successful treatment of tobacco dependence. [Czech]. *Ceska a Slovenska Psychiatrie*, *112*(5), 221–225. | Did not analyse mental health outcomes by exposure of interest |
| Stevens, E. R., Lei, L., Cleland, C. M., Vojjala, M., El-Shahawy, O., Berger, K. I., Kirchner, T. R., Sherman, S. E., & Altose, A. (2022). Electronic cigarettes as a harm reduction strategy among patients with COPD: Protocol for an open-label two arm randomized controlled pilot trial. *Addiction Science & Clinical Practice, 17*. https://doi.org/10.1186/s13722-021-00284-0 | Wrong study design |
| Stockings, E. A. L., Bowman, J. A., Baker, A. L., Terry, M., Clancy, R., Wye, P. M., Knight, J., Moore, L. H., Adams, M. F., Colyvas, K., & Wiggers, J. H. (2014). Impact of a postdischarge smoking cessation intervention for smokers admitted to an inpatient psychiatric facility: A randomized controlled trial. *Nicotine and Tobacco Research, 16*(11), 1417–1428. https://doi.org/10.1093/ntr/ntu097 | Wrong outcome |
| Stockings, E. A., Bowman, J. A., Wiggers, J., Baker, A. L., Terry, M., Clancy, R., Wye, P. M., Knight, J., & Moore, L. H. (2011). A randomised controlled trial linking mental health inpatients to community smoking cessation supports: A study protocol. *BMC Public Health*, *11*, 570. | Wrong outcome |
| Stone K.C. (2023). Postpartum Behavioral Sleep Intervention for Smoking Relapse Prevention: A Pilot Trial. *Maternal and Child Health Journal*. https://doi.org/10.1007/s10995-022-03575-3 | Wrong study design |
| Strasser, A. A., Kaufmann, V., Jepson, C., Perkins, K. A., Pickworth, W. B., Wileyto, E. P., Rukstalis, M., Audrain-McGovern, J., & Lerman, C. (2005). Effects of different nicotine replacement therapies on postcessation psychological responses. *Addict Behav, 30*(1), 9–17. https://doi.org/10.1016/j.addbeh.2004.04.005 | Mental health measured at baseline only, no follow up |
| Streck, J. M., Luberto, C. M., Muzikansky, A., Skurla, S., Ponzani, C. J., Perez, G. K., Hall, D. L., Gonzalez, A., Mahaffey, B., Rigotti, N. A., Ostroff, J. S., & Park, E. R. (2021). Examining the effects of stress and psychological distress on smoking abstinence in cancer patients. *Preventive Medicine Reports, 23*(101643766), 101402. https://doi.org/10.1016/j.pmedr.2021.101402 | Wrong study design |
| Stone, K. C. (2023). Postpartum Behavioral Sleep Intervention for Smoking Relapse Prevention: A Pilot Trial. *Maternal and Child Health Journal, 27,* 272-285. https://doi.org/10.1007/s10995-022-03575-3 | Wrong study design |
| Strong, D., Kahler, C., Leventhal, A., Abrantes, A., Lloyd-Richardson, E., Niaura, R., & Brown, R. (2009). Impact of bupropion and cognitive-behavioral treatment for depression on positive affect, negative affect, and urges to smoke during cessation treatment. *Nicotine & Tobacco Research, 11*, 1142–1153. https://doi.org/10.1093/ntr/ntp111 | Wrong outcome |
| Stubbs, B., Hoots, V., Clements, A., & Bailey, B. (2019). Psychosocial well-being and efforts to quit smoking in pregnant women of South-Central Appalachia. *Addictive Behaviors Reports, 9*. https://doi.org/10.1016/j.abrep.2019.100174 | Mental health measured at baseline only, no follow up |
| Suarez-Castro, D., Barroso-Hurtado, M., Martinez-Vispo, C., Becona, E., & Lopez-Duran, A. (2023). Boredom susceptibility and quit smoking: The role of anxiety symptoms. *The Journal of Psychology: Interdisciplinary and Applied*, *157*(4), 242–251. https://doi.org/10.1080/00223980.2023.2183933 | Mental health measured at baseline only, no follow up |
| Suchting, R., Hebert, E. T., Ma, P., Kendzor, D. E., Businelle, M. S., & Akaike, A. (2019). Using elastic net penalized cox proportional hazards regression to identify predictors of imminent smoking lapse. *Nicotine & Tobacco Research, 21*(2), 173–179. https://doi.org/10.1093/ntr/ntx201 | Wrong study design |
| Sukhovskaya O., Titova O., Kulikov V., & Volchkova E. (2019). The value of behavioral regulation in patients with bronchial asthma in case of smoking cessation. *European Respiratory Journal, 54*(Supplement 63). https://doi.org/10.1183/13993003.congress-2019.PA4495 | Wrong study design |
| Sun, R. (2020). Interaction between smoking and body weight: Implications for public health policy. *Dissertation Abstracts International: Section B: The Sciences and Engineering*, *81*(8-B). | Wrong study design |
| Sung, W.-S., Choi, I. S., Moon, J.-H., Chae, S.-Y., Jo, M.-G., Kim, J.-H., Park, Y.-C., Kim, E.-J., Baek, Y.-H., Kim, G.-W., & Seo, B.-K. (2022). Efficacy and safety evaluation of adjuvant auricular acupuncture for smoking cessation: A study protocol of randomized, assessor-blinded, pragmatic pilot trial. *Medicine, 101*(43), e31456. https://doi.org/10.1097/MD.0000000000031456 | Wrong study design |
| Suteerangkul, P., Lagampan, S., Kalampakorn, S., & Auemaneekul, N. (2021). The effects of community participation program on smoke-free homes in a suburban community of Thailand. *Tobacco Induced Diseases, 19*(101201591), 35. https://doi.org/10.18332/tid/133876 | Wrong outcome |
| Sverre E., Peersen K., Perk J., Husebye E., Gullestad L., Dammen T., Otterstad J.E., & Munkhaugen J. (2021). Challenges in coronary heart disease prevention-experiences from a long-term follow-up study in Norway. *Scandinavian Cardiovascular Journal, 55*(2), 73–81. https://doi.org/10.1080/14017431.2020.1852308 | Wrong outcome |
| Sweeney, R., Moodie, M., Baker, A. L., Borland, R., Castle, D., Segan, C., Turner, A., Attia, J., Kelly, P. J., Brophy, L., Bonevski, B., Williams, J. M., Baird, D., White, S. L., McCarter, K., & Baker, B. (2019). Protocol for an economic evaluation of the quitlink randomized controlled trial for accessible smoking cessation support for people with severe mental illness. *Frontiers in Psychiatry, 10*. https://doi.org/10.3389/fpsyt.2019.00618 | Wrong outcome |
| Tabi, S., Heitner, S. A., Shivale, S., Minchenberg, S., Faraone, S. V., & Johnson, B. (2020). Opioid Addiction/Pregnancy and Neonatal Abstinence Syndrome (NAS): A Preliminary Open-Label Study of Buprenorphine Maintenance and Drug Use Targeted Psychotherapy (DUST) on Cessation of Addictive Drug Use. *Frontiers in Psychiatry, 11*(101545006), 563409. https://doi.org/10.3389/fpsyt.2020.563409 | Wrong study design |
| Tadzimirwa, G. Y., Day, C., Esmail, A., Cooper, C., Kamkuemah, M., Dheda, K., & Van Zyl-Smit, R. N. (2019). Challenges for dedicated smoking cessation services in developing countries. *South African Medical Journal [Suid-Afrikaanse Tydskrif Vir Geneeskunde], 109*(6), 431–436. https://doi.org/10.7196/SAMJ.2019.v109i6.13631 | Wrong study design |
| Taniguchi, C., Hashiba, C., Saka, H., Tanaka, H., & Addington, B. (2020). Characteristics, outcome and factors associated with success of quitting smoking in 77 people living with HIV/AIDS who received smoking cessation therapy in Japan. *Japan Journal of Nursing Science, 17*(1). https://doi.org/10.1111/jjns.12264 | Wrong outcome |
| Taniguchi, C., Narisada, A., Tanaka, H., Iida, H., Iida, M., Mori, R., Nakayama, A., Suzuki, K., & Biala, B. (2022). Smoking cessation after cancer diagnosis reduces the risk of severe cancer pain: A longitudinal cohort study. *PLoS ONE, 17*(8). https://doi.org/10.1371/journal.pone.0272779 | Wrong outcome |
| Taniguchi, C., Saka, H., Oze, I., Nakamura, S., Nozaki, Y., Tanaka, H., & Allen, B. (2020). Relationship between the strength of craving as assessed by the Tobacco Craving Index and success of quitting smoking in Japanese smoking cessation therapy. *PLoS ONE, 15*(12). https://doi.org/10.1371/journal.pone.0243374 | Wrong exposure |
| Taskin O., Demir U., & Soylu V.G. (2021). Investigation of the effect of smoking on depression and stress in healthcare workers during a pandemic. *Annals of Clinical and Analytical Medicine, 12*(6), 638–641. https://doi.org/10.4328/ACAM.20367 | Wrong study design |
| Tavakoli-Ardakani, M., Gholamzadeh Sani, Z., Beyraghi, N., Najarimoghadam, S., & Kheradmand, A. (2024). Comparison between cytisine and Nicotine Replacement Therapy in smoking cessation among inpatient psychiatric patients. *Journal of Addictive Diseases*, *42*(4), 352-359. https://doi.org/10.1080/10550887.2023.2237395 | Wrong outcome |
| Taylor A., Thompson T.P., Ussher M., Aveyard P., Murray R.L., Harris T., Creanor S., Green C., Streeter A.J., Chynoweth J., Ingram W., Greaves C.J., Hancocks H., Snowsill T., Callaghan L., Price L., Horrell J., King J., Gude A., … Preece D. (2020). Randomised controlled trial of tailored support to increase physical activity and reduce smoking in smokers not immediately ready to quit: Protocol for the Trial of physical Activity-Assisted Reduction of Smoking (TARS) Study. *BMJ Open, 10*(12), e043331. https://doi.org/10.1136/bmjopen-2020-043331 | Wrong study design |
| Taylor S., Pinnock H., Sohanpal R., Steed E., Chan C., Healey A., Hooper R., Heslop K., Kelly M., Priebe S., Roberts C.M., Singh S., Saqi-Waseem S., Underwood M., White P., & Warburton C. (2021). RCT Abstract- Tailored psychological intervention for anxiety/depression in people with COPD (TANDEM): A randomised controlled trial. *European Respiratory Journal, 58*(SUPPL 65). https://doi.org/10.1183/13993003.congress-2021.RCT211 | Wrong study design |
| Taylor, G. M. J., Itani, T., Thomas, K. H., Rai, D., Jones, T., Windmeijer, F., Martin, R. M., Munafo, M. R., Davies, N. M., Taylor, A. E., & Anthenelli, B. (2020). Prescribing prevalence, effectiveness, and mental health safety of smoking cessation medicines in patients with mental disorders. *Nicotine & Tobacco Research, 22*(1), 48–57. https://doi.org/10.1093/ntr/ntz072 | Did not distinguish between unsuccessful quit attempt and no attempt to quit |
| Taylor, G., Aveyard, P., Bartlem, K., Shaw, A., Player, J., Metcalfe, C., Kessler, D., & Munafo, M. (2019). IntEgrating Smoking Cessation treatment As part of usual Psychological care for dEpression and anxiety (ESCAPE): Protocol for a randomised and controlled, multicentre, acceptability, feasibility and implementation trial. *Pilot and Feasibility Studies, 5*(101676536), 16. https://doi.org/10.1186/s40814-018-0385-2 | Wrong study design |
| Taylor, K. L., Deros, D. E., Fallon, S., Stephens, J., Kim, E., Lobo, T., Davis, K. M., Luta, G., Jayasekera, J., Meza, R., Stanton, C. A., Niaura, R. S., Abrams, D. B., McKee, B., Howell, J., Ramsaier, M., Batlle, J., Dornelas, E., Parikh, V., & Anderson, E. (2019). Study protocol for a telephone-based smoking cessation randomized controlled trial in the lung cancer screening setting: The lung screening, tobacco, and health trial. *Contemporary Clinical Trials, 82*(101242342), 25–35. https://doi.org/10.1016/j.cct.2019.05.006 | Wrong outcome |
| TCTR20180312001. (2017). Efficacy safety and Health-Related Quality of Life (HRQoL) of cytisine in Smoking cessation. http://Www.Who.Int/Trialsearch/Trial2.Aspx?TrialID=TCTR20180312001. | Did not analyse mental health outcomes by exposure of interest |
| Tekeli, A. E., Demirkiran, H., Kacar, C., Duzenli, U., & Gulhas, N. (2022). The Relationship between Preoperative Smoking Cessation, Anxiety, and Postoperative Anxiety and Pain: A Prospective Clinical Trial at a University Hospital in the East of Turkey on 120 Participants. *JPMA. The Journal of the Pakistan Medical Association, 72*(9), 1699–1703. https://doi.org/10.47391/JPMA.369 | Wrong study design |
| Thanomsat K., Yunibhand J., & Preechawong S. (2022). An Integrated Smoking Cessation Intervention in the Primary Care Service System: An Intervention Mapping. *Open Public Health Journal, 15*(1), e187494452207280. https://doi.org/10.2174/18749445-v15-e2207280 | Wrong study design |
| Thao, V., Nyman, J. A., Nelson, D. B., Joseph, A. M., Clothier, B., Hammett, P. J., Fu, S. S., & Abrams, B. (2019). Cost-effectiveness of population-level proactive tobacco cessation outreach among socio-economically disadvantaged smokers: Evaluation of a randomized control trial. *Addiction, 114*(12), 2206–2216. https://doi.org/10.1111/add.14752 | Wrong study design |
| Thayer, L. S., Tiffany, E. M., & Carreira, D. S. (2021). Addressing Smoking in Musculoskeletal Specialty Care. *The Journal of Bone and Joint Surgery. American Volume, 103*(22), 2145–2152. https://doi.org/10.2106/JBJS.21.00108 | Wrong study design |
| Theuerkauff O. & Hanak C. (2020). TOBACCO USE CESSATION in THREE PATIENTS SUFFERING from PSYCHOTIC DISORDERS: The IMPACT of the COVID-19 PANDEMIC. *Psychiatria Danubina*, *32*, S21–S23. | Wrong outcome |
| Thirlway F., Nyamurungi K.N., Matovu J.K.B., Miti A.K., & Mdege N.D. (2021). Tobacco use and cessation in the context of ART adherence: Insights from a qualitative study in HIV clinics in Uganda. *Social Science and Medicine, 273*, 113759. https://doi.org/10.1016/j.socscimed.2021.113759 | Wrong study design |
| Thomas A., Ivanova A., Baliunas D., Zawertailo L., & Selby P. (2019). Association of self-reported chronic pain and smoking cessation outcomes in primary care. *Journal of Addiction Medicine, 13*(3), E22. https://doi.org/10.1097/ADM.0000000000000548 | Wrong outcome |
| Thomas, D. P., Davey, M., van der Sterren, A. E., Panaretto, K. S., & Lyons, L. (2020). Do stress, life satisfaction, depression and alcohol use predict quitting among Aboriginal and Torres Strait Islander smokers?. *Australian and New Zealand Journal of Public Health, 44*(3), 186–192. https://doi.org/10.1111/1753-6405.12993 | Wrong study design |
| Thomas, D., Farrell, M., McRobbie, H., Tutka, P., Petrie, D., West, R., Siahpush, M., Gartner, C., Walker, N., Mendelsohn, C. P., Hall, W., Paul, C., Zwar, N., Ferguson, S. G., Boland, V. C., Richmond, R., Doran, C. M., Shakeshaft, A., Mattick, R. P., … Anthenelli, A. (2019). The effectiveness, safety and cost-effectiveness of cytisine versus varenicline for smoking cessation in an Australian population: A study protocol for a randomized controlled non-inferiority trial. *Addiction, 114*(5), 923–933. https://doi.org/10.1111/add.14541 | Wrong study design |
| Thorndike, A. N., Regan, S., McKool, K., Pasternak, R. C., Swartz, S., Torres-Finnerty, N., & Rigotti, N. A. (2008). Depressive symptoms and smoking cessation after hospitalization for cardiovascular disease. *Arch Intern Med, 168*(2), 186–191. https://doi.org/10.1001/archinternmed.2007.60 | Did not analyse mental health outcomes by exposure of interest |
| Timberlake, D. S., Rhee, J., & Antognoli, B. (2022). Do smokers’ harm perceptions of cigarillos differ by modified use of the tobacco product? Findings from waves 3 and 4 of the PATH study. *Psychology of Addictive Behaviors.* https://doi.org/10.1037/adb0000819 | Wrong study design |
| Tindle, H. A., Freiberg, M. S., Cheng, D. M., Gnatienko, N., Blokhina, E., Yaroslavtseva, T., Bendiks, S., Patts, G., Hahn, J., So-Armah, K., Stein, M. D., Bryant, K., Lioznov, D., Krupitsky, E., & Samet, J. H. (2022). Effectiveness of Varenicline and Cytisine for Alcohol Use Reduction Among People With HIV and Substance Use: A Randomized Clinical Trial. *JAMA Network Open, 5*(8), e2225129. https://doi.org/10.1001/jamanetworkopen.2022.25129 | Wrong study design |
| Tinsel, I., Metzner, G., Schlett, C., Sehlbrede, M., Bischoff, M., Anger, R., Brame, J., Konig, D., Wurst, R., Fuchs, R., Lindinger, P., Bredenkamp, R., & Farin-Glattacker, E. (2021). Effectiveness of an interactive web-based health program for adults: A study protocol for three concurrent controlled-randomized trials (EVA-TK-Coach). *Trials, 22*(1), 526. https://doi.org/10.1186/s13063-021-05470-8 | Wrong outcome |
| Tojal, C., & Costa, R. (2020). Anxiety and depression symptoms among pregnant women with different smoking habits. *Psychology, Health & Medicine, 25*(4), 410–417. https://doi.org/10.1080/13548506.2019.1634820 | Wrong exposure |
| Tomioka H., Hashimoto R., & Wada T. (2019). Smoking cessation in COPD patients with nicotine dependence in a single-center clinic in Japan. *American Journal of Respiratory and Critical Care Medicine*, *199*(9). | Wrong study design |
| Tomita, A., Manuel, J. I., & Albers, A. (2020). Evidence on the association between cigarette smoking and incident depression from the South African National Income Dynamics Study 2008-2015: Mental health implications for a resource-limited setting. *Nicotine & Tobacco Research, 22*(1), 118–123. https://doi.org/10.1093/ntr/nty163 | Did not analyse mental health outcomes by exposure of interest |
| Tomson, T. (2022). Telephone support for smoking cessation: The Swedish example. *Dissertation Abstracts International: Section B: The Sciences and Engineering*, *83*(2-B). | Wrong study design |
| Tonkin, S. S., Colder, C., Mahoney, M. C., Swan, G. E., Cinciripini, P., Schnoll, R., George, T. P., Tyndale, R. F., & Hawk, L. W. (2022). Evaluating Treatment Mechanisms of Varenicline: Mediation by Affect and Craving. *Nicotine & Tobacco Research, 24*(11), 1803–1810. https://doi.org/10.1093/ntr/ntac138 | Wrong outcome |
| Tonnesen H., Lydom L.N., Joensen U.N., Egerod I., Pappot H., & Lauridsen S.V. (2022). STRONG for Surgery & Strong for Life—Against all odds: Intensive prehabilitation including smoking, nutrition, alcohol and physical activity for risk reduction in cancer surgery—A protocol for an RCT with nested interview study (STRONG-Cancer). *Trials, 23*(1), 333. https://doi.org/10.1186/s13063-022-06272-2 | Wrong study design |
| Tranel, D., McNutt, A., & Bechara, A. (2012). Smoking cessation after brain damage does not lead to increased depression: Implications for understanding the psychiatric complications of varenicline. *Cognitive and Behavioral Neurology, 25*(1), 16–24. https://doi.org/10.1097/WNN.0b013e3182492a9c | Mental health not measured before quit attempt |
| Tsima B.M., Moedi P., Maunge J., MacHangane K., Kgogwane M., Mudojwa T., Bastian J., Bilker W., Ashare R., Schnoll R., & Gross R. (2020). Feasibility of implementing a novel behavioural smoking cessation intervention amongst human immunodeficiency virus-infected smokers in a resource-limited setting: A single-arm pilot trial. *Southern African Journal of HIV Medicine, 21*(1), a1075. https://doi.org/10.4102/SAJHIVMED.V21I1.1075 | Wrong study design |
| Tsoh, J. Y., Humfleet, G. L., Muð®¸¼, R. F., Reus, V. I., Hartz, D. T., & Hall, S. M. (2000). Development of major depression after treatment for smoking cessation. *American Journal of Psychiatry, 157*(3), 368–374. https://doi.org/10.1176/appi.ajp.157.3.368 | Wrong outcome |
| Tucker, C. J., Bello, M. S., Weinberger, A. H., D’Orazio, L. M., Kirkpatrick, M. G., Pang, R. D., & al’Absi, A. (2022). Association of depression symptom level with smoking urges, cigarette withdrawal, and smoking reinstatement: A preliminary laboratory study. *Drug and Alcohol Dependence, 232*, 1–9. https://doi.org/10.1016/j.drugalcdep.2022.109267 | Did not analyse mental health outcomes by exposure of interest |
| Tulloch H, Pipe A, Els C, Aitken D, Clyde M, Corran B, & Reid RD. (2014). Flexible and extended dosing of nicotine replacement therapy or varenicline in comparison to fixed dose nicotine replacement therapy for smoking cessation: Rationale, methods and participant characteristics of the FLEX trial. *Contemporary Clinical Trials, 38*(2), 304–313. https://doi.org/10.1016/j.cct.2014.05.011 | Did not analyse mental health outcomes by exposure of interest |
| Ukai, T., Tabuchi, T., & Iso, H. (2022). The impact of spousal behavior changes on smoking, drinking and physical activity: The longitudinal survey of middle-aged and elderly persons in Japan. *Preventive Medicine, 164*(pm4, 0322116), 107293. https://doi.org/10.1016/j.ypmed.2022.107293 | Wrong outcome |
| Um Y.H., Wang S.-M., Han K.-D., Kim N.-Y., Kang D.W., Na H.-R., Lee C.U., & Lim H.K. (2020). Differential impact of cigarette smoking on fracture risks in subjective cognitive decline and dementia: A nationwide longitudinal study. *Psychiatry Investigation, 17*(8), 786–795. https://doi.org/10.30773/pi.2020.0165 | Wrong outcome |
| UMIN000027036. (2017). A randomized, multi-center, double-blind, placebo-controlled trial for the effects of yokukansan on depressive or neurotic smoking patients during smoking cessation therapy. http://Www.Who.Int/Trialsearch/Trial2.Aspx?TrialID=JPRN-UMIN000027036. | Did not analyse mental health outcomes by exposure of interest |
| Uzer F. & Uzun R. (2019). General health status and smoking cessation rates of individuals admitted to smoking cessation outpatient clinic. *Turkish Thoracic Journal, 20*(Supplement 1), S113. https://doi.org/10.5152/TurkThoracJ.2019.113 | Wrong exposure |
| Valdiviezo W.V., Aldaz E.M., Paredes F.P., & De Las Mercedes Hernandez Bandera N. (2022). Self-Management Of Patients With Mild Copd In Primary Care: A Random Controlled Trial. *Journal of Pharmaceutical Negative Results, 13*, 1904–1914. https://doi.org/10.47750/pnr.2022.13.S07.262 | Wrong study design |
| Valera, P., Malarkey, S., Smith, N., & McLaughlin, C. (2021). Exploring the role of telehealth: A novel approach to group-based smoking cessation treatment for men incarcerated in a rural state prison. *Journal of Telemedicine and Telecare, 9506702*, cpj, 1357633X211034734. https://doi.org/10.1177/1357633X211034734 | Wrong study design |
| Valera, P., Malarkey, S., Smith, N., & McLaughlin, C. (2024). Exploring the role of telehealth: A novel approach to group-based smoking cessation treatment for men incarcerated in a rural state prison. *Journal of Telemedicine and Telecare*, *30*(1), 142–150. https://doi.org/10.1177/1357633X211034734 | Mental health measured at baseline only, no follow up |
| Van De Ven D., Robroek S., Hengel K.O., Van Zon S., Brouwer S., Ots P., Burdorf L., & Schuring M. (2021). The impact of within-individual changes in working conditions, health behaviour and bmi on work ability and self-rated health: A fixed-effects analysis among dutch workers. *Occupational and Environmental Medicine, 78*(SUPPL 1), A56–A57. https://doi.org/10.1136/OEM-2021-EPI.151 | Wrong study design |
| van de Ven, D., Robroek, S. J., Oude Hengel, K. M., van Zon, S. K., Brouwer, S., Ots, P., Burdorf, A., & Schuring, M. (2022). Associations of within-individual changes in working conditions, health behaviour and BMI with work ability and self-rated health: A fixed effects analysis among Dutch workers. BMJ Open, 12(4), e058574. https://doi.org/10.1136/bmjopen-2021-058574 | Wrong outcome |
| Van Den Brand F.A., Candel M.J.J.M., Nagelhout G.E., Winkens B., & Van Schayck C.P. (2021). How financial incentives increase smoking cessation: A two-level path analysis. *Nicotine and Tobacco Research, 23*(1), 99–106. https://doi.org/10.1093/ntr/ntaa024 | Wrong outcome |
| van denBrand, F. A., Nagelhout, G. E., Winkens, B., Chavannes, N. H., vanSchayck, O. C. P., Evers, S. M. A. A., & Berman, B. (2020). Cost-effectiveness and cost-utility analysis of a work-place smoking cessation intervention with and without financial incentives. *Addiction, 115*(3), 534–545. https://doi.org/10.1111/add.14861 | Wrong study design |
| Van Wijk L., Buis C.I., & Klaase J.M. (2020). Feasibility of a prehabilitation clinic for patients undergoing oncologic abdominal surgery: The FRAIL study. *European Journal of Surgical Oncology, 46*(2), e15. https://doi.org/10.1016/j.ejso.2019.11.459 | Wrong study design |
| Varela, L. B., Correa, F., Cazaux, A., Spaccesi, A., Salica, D. A., & Vanoni, S. (2021). Stable Chronic Obstructive Pulmonary Disease Associated With Cognitive Impairment: Possible Causality Factor. [La Enfermedad Pulmonar Obstructiva Cronica Estable Asociada a Deterioro Cognitivo: Posible Factor de Causalidad], *78*(2), 97–102. https://doi.org/10.31053/1853.0605.v78.n2.28721 | Wrong study design |
| Veldheer, S., Yingst, J., Midya, V., Hummer, B., Lester, C., Krebs, N., Hrabovsky, S., Wilhelm, A., Liao, J., Yen, M.-S., Cobb, C., Eissenberg, T., Foulds, J., & Abrams, B. (2019). Pulmonary and other health effects of electronic cigarette use among adult smokers participating in a randomized controlled smoking reduction trial. *Addictive Behaviors, 91.* https://doi.org/10.1016/j.addbeh.2018.10.041 | Wrong outcome |
| Veldhuizen, S., Mahinpey, N., Zawertailo, L., Minian, N., Melamed, O., & Selby, P. (2022). Effects of COVID-19-Related Disruptions on Service Use in a Large Smoking Cessation Program. *Nicotine & Tobacco Research, 24*(10), 1567–1572. https://doi.org/10.1093/ntr/ntac103 | Wrong study design |
| Veldhuizen, S., Zawertailo, L., & Selby, P. (2021). Variability in outcomes and quality-of-care indicators across clinics participating in a large smoking-cessation program. *Journal of Substance Abuse Treatment, 130*(kai, 8500909), 108409. https://doi.org/10.1016/j.jsat.2021.108409 | Wrong study design |
| Vermeulen, J., Schirmbeck, F., Blankers, M., van Tricht, M., van den Brink, W., de Haan, L., Alizadeh, B. Z., van Amelsvoort, T., Bartels-Velthuis, A. A., van Beveren, N. J., Bruggeman, R., Cahn, W., Delespaul, P., Luykx, J. J., Myin-Germeys, I., Kahn, R. S., Simons, C. J. P., van Haren, N. E., van Os, J., & van Winkel, R. (2019). Smoking, symptoms, and quality of life in patients with psychosis, siblings, and healthy controls: A prospective, longitudinal cohort study. *The Lancet Psychiatry, 6*(1), 25–34. https://doi.org/10.1016/S2215-0366%2818%2930424-3 | Did not distinguish between unsuccessful quit attempt and no attempt to quit |
| Verveer I., Remmerswaal D., Jongerling J., van der Veen F.M., & Franken I.H.A. (2020). No effect of repetitive tDCS on daily smoking behaviour in light smokers: A placebo controlled EMA study. *PloS One, 15*(5), e0233414. https://doi.org/10.1371/journal.pone.0233414 | Wrong study design |
| Vidrine DJ, Kypriotakis G, Li L, Arduino RC, Fletcher FE, Tami-Maury I, & Gritz ER. (2015). Mediators of a smoking cessation intervention for persons living with HIV/AIDS. *Drug and Alcohol Dependence, 147*, 76–80. https://doi.org/10.1016/j.drugalcdep.2014.12.003 | Did not analyse mental health outcomes by exposure of interest |
| Vidrine Jennifer Irvin, Businelle Michael S, Reitzel Lorraine R, Cao Yumei, Cinciripini Paul M, Marcus Marianne T, Li Yisheng, & Wetter David W. (2014). Coping mediates the association of mindfulness with psychological stress, affect, and depression among smokers preparing to quit. *Mindfulness*, Advance online publication. https://doi.org/10.1007/s12671-014-0276-4 | Wrong study design |
| Vieira A.C.R., Ferra J., Carvalho J., Oliveira I., Matos C., & Nogueira F. (2019). Impact of educational level in smoking cessation. *European Respiratory Journal, 54*(Supplement 63). https://doi.org/10.1183/13993003.congress-2019.PA2850 | Wrong study design |
| Vojjala, M., Wysota, C. N., Oketunbi, O., King, Q., & Rogers, E. S. (2023). Integrating the ‘Quit and Stay Quit Monday’ Model into Smoking Cessation Services for Smokers with Mental Health Conditions: A Pilot Randomized Controlled Trial. *Journal of Smoking Cessation*, *2023*(101478447), 8165232. https://doi.org/10.1155/2023/8165232 | Wrong exposure |
| Walker, N., Smith, B., Barnes, J., Verbiest, M., Parag, V., Pokhrel, S., Wharakura, M.-K., Lees, T., Cubillos Gutierrez, H., Jones, B., Bullen, C., & Anraad, A. (2021). Cytisine versus varenicline for smoking cessation in New Zealand indigenous Maori: A randomized controlled trial. *Addiction, 116*(10), 2847–2858. https://doi.org/10.1111/add.15489 | Wrong study design |
| Wallace, J. D., Doshi, U., Chaiken, S. R., & Caughey, A. B. (2023). Financial incentives for smoking cessation during pregnancy and postpartum: A cost-effectiveness analysis. *American Journal of Obstetrics and Gynecology*, *228*(1 Supplement), S494–S495. https://doi.org/10.1016/j.ajog.2022.11.850 | Full text not available |
| Wand, H., Richmond, R., Adily, A., Le, A., Wilhelm, K., & Butler, T. (2020). Identifying significant contributors for smoking cessation among male prisoners in Australia: Results from a randomised clinical trial. *BMJ Open, 10*(7), e034046. https://doi.org/10.1136/bmjopen-2019-034046 | Did not distinguish between unsuccessful quit attempt and no attempt to quit |
| Wang F., Chen Y., & Li S. (2021). The effect of art therapy on reoccurring smoking among youths. *American Journal of Translational Research*, *13*(9), 10633–10640. | Wrong study design |
| Wang Y., Van Boven J.F.M., Bos J.H.J., Schuiling-Veninga C.C.M., Boezen H.M., Wilffert B., & Hak E. (2020). Risk of neuropsychiatric adverse events associated with varenicline treatment for smoking cessation: A prescription sequence symmetry analysis. *Pharmacoepidemiology and Drug Safety, 29*(SUPPL 3), 428–429. https://doi.org/10.1002/pds.5114 | Wrong exposure |
| Wang, L., Guo, Y., Wang, M., Zhao, Y., & Audulv, B. (2021). A mobile health application to support self-management in patients with chronic obstructive pulmonary disease: A randomised controlled trial. *Clinical Rehabilitation, 35*(1), 90–101. https://doi.org/10.1177/0269215520946931 | Wrong outcome |
| Wang, Y., Bos, J. H., Schuiling-Veninga, C. C. M., Boezen, H. M., van Boven, J. F. M., Wilffert, B., & Hak, E. (2021). Neuropsychiatric safety of varenicline in the general and COPD population with and without psychiatric disorders: A retrospective cohort study in a real-world setting. *BMJ Open, 11*(5), e042417. https://doi.org/10.1136/bmjopen-2020-042417 | Did not analyse mental health outcomes by exposure of interest |
| Wang, Y.-Z., Chen, H.-H., Yeh, M.-L., & Lin, S.-D. (2010). Auricular acupressure combined with multimedia instruction or alone for quitting smoking in young adults: A quasi-experimental study. *International Journal of Nursing Studies, 47*, 1089–1095. https://doi.org/10.1016/j.ijnurstu.2010.02.009 | Wrong outcome |
| Waters, A. F., Peltier, M. R., Roys, M. R., Stewart, S. A., & Copeland, A. L. (2021). Smoking and suicidal ideation among college students: Smoking expectancies as potential moderators. *Journal of American College Health, 69*(8), 951–958. https://doi.org/10.1080/07448481.2020.1719112 | Wrong study design |
| Watson N.L., Heffner J.L., Mull K.E., McClure J.B., & Bricker J.B. (2021). Which method of assessing depression and anxiety best predicts smoking cessation: Screening instruments or self-reported conditions? *Nicotine and Tobacco Research, 22*(10), 1860–1866. https://doi.org/10.1093/NTR/NTAA099 | Mental health measured at baseline only, no follow up |
| Watson, N. L., Heffner, J. L., Mull, K. E., McClure, J. B., Bricker, J. B., & An, A. (2019). Comparing treatment acceptability and 12-month cessation rates in response to Web-based smoking interventions among smokers who do and do not screen positive for affective disorders: Secondary analysis. *Journal of Medical Internet Research, 21*(6). https://doi.org/10.2196/13500 | Wrong study design |
| Watson, N. L., Mull, K. E., Bricker, J. B., & Amato, B. (2021). The association between frequency of e-cigarette use and long-term smoking cessation outcomes among treatment-seeking smokers receiving a behavioral intervention. *Drug and Alcohol Dependence, 218.* https://doi.org/10.1016/j.drugalcdep.2020.108394 | Wrong study design |
| Webb Hooper, M., & Kolar, S. K. (2015). Distress, race/ethnicity and smoking cessation in treatment-seekers: Implications for disparity elimination. *Addiction*, *110*(9), 1495–1504. | Did not analyse mental health outcomes by exposure of interest |
| Webb Hooper, M., Lee, D. J., Simmons, V. N., Brandon, K. O., Antoni, M. H., Unrod, M., Asfar, T., Correa, J. B., Koru-Sengul, T., & Brandon, T. H. (2018). Reducing racial/ethnic tobacco cessation disparities via cognitive behavioral therapy: Design of a dualsite randomized controlled trial. *Contemporary Clinical Trials, 68,* 127–132. https://doi.org/10.1016/j.cct.2018.03.017 | Wrong study design |
| Webb J., Peerbux S., Smittenaar P., Siddiqui S., Sherwani Y., Ahmed M., MacRae H., Puri H., Bhalla S., & Majeed A. (2020). Preliminary outcomes of a digital therapeutic intervention for smoking cessation in adult smokers: Randomized controlled trial. *JMIR Mental Health, 7*(10), e22833. https://doi.org/10.2196/22833 | Wrong study design |
| Weidberg, S., Gonzalez-Roz, A., Garcia-Fernandez, G., Secades-Villa, R., & Anderson, A.-M. (2021). Activation level as a mediator between behavioral activation, sex, and depression among treatment-seeking smokers. *Addictive Behaviors, 114.* https://doi.org/10.1016/j.addbeh.2020.106715 | Wrong study design |
| Weinberger A.H., Chaiton M.O., Zhu J., Wall M.M., Hasin D.S., & Goodwin R.D. (2020). Trends in the Prevalence of Current, Daily, and Nondaily Cigarette Smoking and Quit Ratios by Depression Status in the U.S.: 2005-2017. *American Journal of Preventive Medicine, 58*(5), 691–698. https://doi.org/10.1016/j.amepre.2019.12.023 | Wrong study design |
| Weinberger, A. H., Pilver, C. E., Desai, R. A., Mazure, C. M., & McKee, S. A. (2012). The relationship of major depressive disorder and gender to changes in smoking for current and former smokers: Longitudinal evaluation in the US population. *Addiction*, *107*(10), 1847–1856. | Wrong outcome |
| Weiss de Souza, I. C., Kozasa, E. H., Bowen, S., Richter, K. P., Sartes, L. M. A., Colugnati, F. A. B., Noto, A. R., & Andreu, A. (2020). Effectiveness of mindfulness-based relapse prevention program as an adjunct to the standard treatment for smoking: A pragmatic design pilot study. *Nicotine & Tobacco Research, 22*(9), 1605–1613. https://doi.org/10.1093/ntr/ntaa057 | Wrong study design |
| Welch, A. E., Jasek, J. P., Caramanica, K., Chiles, M. C., & Johns, M. (2015). Cigarette smoking and 9/11-related posttraumatic stress disorder among world trade center health registry enrollees, 2003-12. *Preventive Medicine, 73*, 94–99. https://doi.org/10.1016/j.ypmed.2015.01.023 | Did not analyse mental health outcomes by exposure of interest |
| Wen, S., Wiers, R. W., Boffo, M., Grasman, R. P. P. P., Pronk, T., & Larsen, H. (2021). Subtypes of smokers in a randomized controlled trial of a web-based smoking cessation program and their role in predicting intervention non-usage attrition: Implications for the development of tailored interventions. *Internet Interventions, 26*(101631612), 100473. https://doi.org/10.1016/j.invent.2021.100473 | Wrong exposure |
| Werba, J. P., Giroli, M. G., Simonelli, N., Vigo, L., Gorini, A., Bonomi, A., Veglia, F., & Tremoli, E. (2022). Uptake and effectiveness of a primary cardiovascular prevention program in an underserved multiethnic urban community. *Nutrition, Metabolism, and Cardiovascular Diseases, 32*(5), 1110–1120. https://doi.org/10.1016/j.numecd.2022.01.013 | Wrong study design |
| West, R., Gilsenan, A., Coste, F., Zhou, X., Brouard, R., Nonnemaker, J., Curry, S. J., & Sullivan, S. D. (2006). The ATTEMPT cohort: A multi-national longitudinal study of predictors, patterns and consequences of smoking cessation; introduction and evaluation of internet recruitment and data collection methods*. Addiction, 101*(9), 1352–1361. https://doi.org/10.1111/j.1360-0443.2006.01534.x | Wrong outcome |
| Westmaas, J. L., Thewes, B., Seguin Leclair, C., & Lebel, S. (2019). Smoking versus quitting and fear of cancer recurrence 9 years after diagnosis in the American Cancer Society’s Longitudinal Study of Cancer Survivors-I (SCS-I). *Cancer, 125*(23), 4260–4268. https://doi.org/10.1002/cncr.32431 | Wrong outcome |
| Wewers ME, Shoben A, Conroy S, Curry E, Ferketich AK, Murray DM, Nemeth J, & Wermert A. (2017). Effectiveness of two community health worker models of tobacco dependence treatment among community residents of Ohio Appalachia. *Nicotine & Tobacco Research, 19*(12), 1499–1507. https://doi.org/10.1093/ntr/ntw265 | Mental health measured at baseline only, no follow up |
| Wewers, M. E., & Ahijevych, K. L. (1991). Work Stress after Smoking Cessation. *AAOHN Journal, 39*(12), 547–551. https://doi.org/10.1177/216507999103901201 | Wrong outcome |
| Wiebe, S. A., Balfour, L., Cameron, W. D., Sandre, D., Holly, C., Tasca, G. A., MacPherson, P. A., & Ahrens, B. (2021). Psychological changes in successful completers of an hiv-tailored smoking cessation program: Mood, attachment and self-efficacy. *AIDS Care*. https://doi.org/10.1080/09540121.2021.1909697 | Did not distinguish between unsuccessful quit attempt and no attempt to quit |
| Wiley, R. C., Oliver, A. C., Snow, M. B., Bunn, J. Y., Barrows, A. J., Tidey, J. W., Lee, D. C., Sigmon, S. C., Gaalema, D. E., Heil, S. H., Markesich, C., Villanti, A. C., & Higgins, S. T. (2023). The Impact of the Covid-19 Pandemic on Smoking Among Vulnerable Populations. *Nicotine & Tobacco Research, 25*(2), 282–290. https://doi.org/10.1093/ntr/ntac135 | Wrong study design |
| Williams, J. M., Anthenelli, R. M., Morris, C. D., Treadow, J., Thompson, J. R., Yunis, C., & George, T. P. (2012). A randomized, double-blind, placebo-controlled study evaluating the safety and efficacy of varenicline for smoking cessation in patients with schizophrenia or schizoaffective disorder, *Journal of Clinical Psychiatry, 73*(7), 1035. https://doi.org/10.4088/JCP.J12-lcx07908 | Wrong outcome |
| Williams R., Cao P., Li T., Luta G., Smith L., Mandelblatt J., Jeon J., Zhao A., Levy D., Davis K., Stanton C., Niaura R., Abrams D., Lobo T., Anderson E., Meza R., Jayasekera J., & Taylor K.L. (2022). OA10.03 A Randomized Trial of Telephone-Based Smoking Cessation Treatment in the Lung Cancer Screening Setting. *Journal of Thoracic Oncology, 17*(9 Supplement), S28. https://doi.org/10.1016/j.jtho.2022.07.052 | Wrong study design |
| Williams R.M., Cordon M., Eyestone E., Smith L., Luta G., McKee B.J., Regis S.M., Abrams D.B., Niaura R.S., Stanton C.A., Parikh V., Taylor K.L., Anderson E., Batlle J., Harper H., McKee A., McKee B., Dornellas E., Howell J., … Anderson R. (2022). Improved motivation and readiness to quit shortly after lung cancer screening: Evidence for a teachable moment. *Cancer, 128*(10), 1976–1986. https://doi.org/10.1002/cncr.34133 | Wrong exposure |
| Williamson, T. J., Park, E. R., Warner, E. T., Rasmussen, A. W., & Ostroff, J. S. (2023). Quitting smoking after a cancer diagnosis is associated with reductions in stigma and anxiety: A longitudinal mediation analysis. *Stigma and Health*. Advance online publication*.* https://doi.org/10.1037/sah0000461 | Did not analyse mental health outcomes by exposure of interest |
| Wilson D.K., Lorig K., Klein W.M.P., Riley W., Sweeney A.M., & Christensen A. (2019). Efficacy and cost-effectiveness of behavioral interventions in nonclinical settings for improving health outcomes. *Health Psychology, 38*(8), 689–700. https://doi.org/10.1037/hea0000773 | Wrong study design |
| Wilson, S. M., Hair, L. P., Hertzberg, J. S., Kirby, A. C., Olsen, M. K., Lindquist, J. H., Maciejewski, M. L., Beckham, J. C., & Calhoun, P. S. (2016). Abstinence reinforcement therapy (ART) for rural veterans: Methodology for an mHealth smoking cessation intervention. *Contemporary Clinical Trials, 50*, 157–165. https://doi.org/10.1016/j.cct.2016.08.008 | Did not analyse mental health outcomes by exposure of interest |
| Winhusen, T. M., Theobald, J., Lewis, D. F., & Angarita, A. (2019). Substance use outcomes in cocaine-dependent tobacco smokers: A mediation analysis exploring the role of sleep disturbance, craving, anxiety, and depression. *Journal of Substance Abuse Treatment, 96.* https://doi.org/10.1016/j.jsat.2018.10.011 | Wrong study design |
| Wongphan T., Rungruanghiranya S., Tulatammakit S., Chittawatanarat K., Preedapornpakorn K., Sutanthavibul N., Preechawong S., & Petborom P. (2021). A real-world economic evaluation of vernonia cinerea lozenges vs placebo in smoking cessation. *American Journal of Respiratory and Critical Care Medicine, 203*(9). https://doi.org/10.1164/ajrccm-conference.2021.203.1_MeetingAbstracts.A1650 | Wrong study design |
| Wootton, R. E., Greenstone, H. S. R., Abdellaoui, A., Denys, D., Verweij, K. J. H., Munafo, M. R., Treur, J. L., & Abdellaoui, A. (2021). Bidirectional effects between loneliness, smoking and alcohol use: Evidence from a Mendelian randomization study. *Addiction, 116*(2), 400–406. https://doi.org/10.1111/add.15142 | Wrong outcome |
| Wootton, R. E., Richmond, R. C., Stuijfzand, B. G., Lawn, R. B., Sallis, H. M., Taylor, G. M. J., Hemani, G., Jones, H. J., Zammit, S., Davey Smith, G., Munafo, M. R., & Benowitz, B. (2020). Evidence for causal effects of lifetime smoking on risk for depression and schizophrenia: A Mendelian randomisation study. *Psychological Medicine, 50*(14), 2435–2443. https://doi.org/10.1017/S0033291719002678 | Wrong study design |
| Wrucke B., Bauer L., & Bernstein R. (2022). Factors Associated with Cigarette Smoking in Homeless Adults: Findings From an Outpatient Counseling Clinic. *Wisconsin Medical Journal*, *121*(2), 106–110. | Wrong outcome |
| Wu Q., Gilbody S., Li J., Wang H.-I., & Parrott S. (2021). Long-Term Cost-Effectiveness of Smoking Cessation Interventions in People With Mental Disorders: A Dynamic Decision Analytical Model. *Value in Health, 24*(9), 1263–1272. https://doi.org/10.1016/j.jval.2021.04.002 | Wrong outcome |
| Wu, F., Laza-Cagigas, R., Pagarkar, A., Olaoke, A., El Gammal, M., & Rampal, T. (2021). The Feasibility of Prehabilitation as Part of the Breast Cancer Treatment Pathway. *PM & R: The Journal of Injury, Function, and Rehabilitation, 13*(11), 1237–1246. https://doi.org/10.1002/pmrj.12543 | Wrong exposure |
| Xi, Q., Meza, R., Leventhal, A., & Tam, J. (2023). Modeling cigarette smoking disparities between people with and without serious psychological distress in the US, 1997-2100. *Preventive Medicine, 166*(pm4, 0322116), 107385. https://doi.org/10.1016/j.ypmed.2022.107385 | Wrong study design |
| Xie, L., Xu, W., & Zhou, Y. (2021). Spillover effects of adult children’s schooling on parents’ smoking cessation: Evidence from China’s compulsory schooling reform. *Journal of Epidemiology and Community Health, 75*(11), 1104–1110. https://doi.org/10.1136/jech-2020-215326 | Wrong outcome |
| Yadav A., Agarwal D., Kumar T.A., Kishore K., & Warrier R. (2019). Study of Outcomes of Smoking Cessation Interventions in Tuberculosis Patients using Urinary Cotinine Levels. *Journal of Clinical and Diagnostic Research, 13*(11), OC07-OC10. https://doi.org/10.7860/JCDR/2019/42584.13267 | Wrong study design |
| Yang, C.-C., Liu, C.-Y., Wang, K.-Y., Chang, Y.-K., Wen, F.-H., Lee, Y.-C., Chen, M.-L., & Balduyck, B. (2021). Trajectory of smoking behaviour during the first 6 months after diagnosis of lung cancer: A study from Taiwan. *Journal of Advanced Nursing, 77*(5), 2363–2373. https://doi.org/10.1111/jan.14745 | Wrong study design |
| Yang, J., Lin, J.-L., Liu, J., Jiang, X.-W., Zhang, H., & Peng, L. (2022). Estimates of prevalence, time-trend, and association of smoking in adults living with HIV, HBV, and HCV (NHANES 1999-2018). *Scientific Reports, 12*(1), 19925. https://doi.org/10.1038/s41598-022-24291-6 | Wrong study design |
| Yavagal, P. C., & L, N. (2021). Efficacy of Laser Auricular Acupuncture for Smoking Cessation: A randomised controlled trial. *Sultan Qaboos University Medical Journal, 21*(2), e275–e281. https://doi.org/10.18295/squmj.2021.21.02.017 | Wrong study design |
| Yingst, J. M., Lester, C., Livelsberger, C., Allen, S. I., Hammett, E., Veldheer, S., Hummer, B., Bordner, C., Zhu, J., Sciamanna, C. N., Trushin, N., Tan, H. S., Wilson, S. J., Twining, R. C., Foulds, J., Grigson, P. S., & Azzopardi, B. (2022). Pilot randomized controlled trial evaluating the effect of random nicotine delivery on cigarettes per day and smoke exposure. *Special Issue: Sleep & Arousal in Health and Disease, 188.* https://doi.org/10.1016/j.brainresbull.2022.07.017 | Wrong study design |
| Yonek, J. C., Meacham, M. C., Shumway, M., Tolou-Shams, M., Satre, D. D., & Ames, B. (2021). Smoking reduction is associated with lower alcohol consumption and depressive symptoms among young adults over one year. *Drug and Alcohol Dependence, 227*. https://doi.org/10.1016/j.drugalcdep.2021.108922 | Wrong exposure |
| Yook, V., Yoo, J., Han, K., Fava, M., Mischoulon, D., Park, M. J., Kim, H., & Jeon, H. J. (2022). Association between pre-pregnancy tobacco smoking and postpartum depression: A nationwide cohort study. *Journal of Affective Disorders, 316*(h3v, 7906073), 56–62. https://doi.org/10.1016/j.jad.2022.07.065 | Wrong outcome |
| Zangen, A., Moshe, H., Martinez, D., Barnea-Ygael, N., Vapnik, T., Bystritsky, A., Duffy, W., Toder, D., Casuto, L., Grosz, M. L., Nunes, E. V., Ward, H., Tendler, A., Feifel, D., Morales, O., Roth, Y., Iosifescu, D. V., Winston, J., Wirecki, T., … Amiaz, B. (2021). Repetitive transcranial magnetic stimulation for smoking cessation: A pivotal multicenter double-blind randomized controlled trial. *World Psychiatry, 20*(3), 397–404. https://doi.org/10.1002/wps.20905 | Wrong outcome |
| Zarghami M, Taghizadeh F, Sharifpour A, & Alipour A. (2018). Efficacy of smoking cessation on stress, anxiety, and depression in smokers with chronic obstructive pulmonary disease: A randomized controlled clinical trial. *Addict Health, 10*(3), 137–147. https://doi.org/10.22122/ahj.v10i3.600 | Did not analyse mental health outcomes by exposure of interest |
| Zarghami, M., Taghizadeh, F., Sharifpour, A., & Alipour, A. (2019). Efficacy of guided self-change for smoking cessation in chronic obstructive pulmonary disease patients: A randomized controlled clinical trial. *Tobacco Induced Diseases, 17*(101201591), 90. https://doi.org/10.18332/tid/114227 | Wrong study design |
| Zeller M., Sales-Wuillemin E., Guinchard S., Chappe J., Chague F., Ayari H., Maza M., Aboa-Eboule C., Truchot C., Lorgis L., Giroud M., Cottin Y., & Bejot Y. (2020). Psychosocial and behavioral characteristics of still smokers at 6 months after acute cerebro or cardiovascular events: Findings from INEV@L, a prospective pilot study. *Archives of Cardiovascular Diseases Supplements, 12*(1), 156. https://doi.org/10.1016/j.acvdsp.2019.09.323 | Wrong outcome |
| Zeller M., Sales-Wuillemin E., Guinchard S., Chappe J., Chague F., Ayari H., Maza M., Aboa-Eboule C., Truchot D., Lorgis L., Giroud M., Cottin Y., & Bejot Y. (2021). Psychosocial and behavioral characteristics of still smokers at 6 months after acute cerebro or cardiovascular events: Preliminary findings from INEV@L, a prospective pilot study. *European Heart Journal, 42*(SUPPL 1), 2595. https://doi.org/10.1093/eurheartj/ehab724.2595 | Wrong study design |
| Zelman, D. C., Brandon, T. H., Jorenby, D. E., & Baker, T. B. (1992). Measures of affect and nicotine dependence predict differential response to smoking cessation treatments. *J Consult Clin Psychol, 60*(6), 943–952. https://doi.org/10.1037//0022-006x.60.6.943 | Mental health measured at baseline only, no follow up |
| Zeng, J., Liao, Y., Wei, X., Chen, G., Cai, Z., Chen, M., Gou, Y., & Lin, G. (2022). Efficacy and safety of acupuncture combined with auricular acupressure for smoking cessation: A study protocol of a multicentre, randomized, controlled clinical trial. *Frontiers in Neurology, 13*(101546899), 921054. https://doi.org/10.3389/fneur.2022.921054 | Wrong study design |
| Zhai, D., Schiavone, G., Van Diest, I., Vrieze, E., DeRaedt, W., & Van Hoof, C. (2019). Ambulatory Smoking Habits Investigation based on Physiology and Context (ASSIST) using wearable sensors and mobile phones: Protocol for an observational study. *BMJ Open, 9*(9), e028284. https://doi.org/10.1136/bmjopen-2018-028284 | Wrong study design |
| Zhang DD, Eisenberg M, Grandi SM, Joseph L, Pilote L, & Filion K. (2013). Bupropion, smoking cessation, and health-related quality of life following an acute myocardial infarction. *Canadian Journal of Cardiology, 29*(10 SUPPL. 1), S290–S291. https://doi.org/10.1016/j.cjca.2013.07.485 | Wrong outcome |
| Zhang, A., Wang, L., Long, L., Yan, J., Liu, C., Zhu, S., & Wang, X. (2020). Effectiveness and Economic Evaluation of Hospital-Outreach Pulmonary Rehabilitation for Patients with Chronic Obstructive Pulmonary Disease. *International Journal of Chronic Obstructive Pulmonary Disease, 15*(101273481), 1071–1083. https://doi.org/10.2147/COPD.S239841 | Wrong study design |
| Zhang, H., Gilbert, E., Hussain, S., Veldhuizen, S., Le Foll, B., Selby, P., & Zawertailo, L. (2022). Effectiveness of bupropion and varenicline for smokers with baseline depressive symptoms. *Nicotine & Tobacco Research, drz,* 9815751. https://doi.org/10.1093/ntr/ntac288 | Mental health measured at baseline only, no follow up |
| Zhang, J. H., Zhang, L. Q., Yang, Y. P., Li, X., Zhang, Y., Wang, L. Y., Shi, H., Jiang, H., Guo, W., Mu, L., & Zeng, Y. (2020). [Clinical effect of nutritional and psychological intervention combined with pulmonary rehabilitation exercise on patients with chronic obstructive pulmonary disease]. *Zhonghua Yi Xue Za Zhi, 100*(2), 110–115. https://doi.org/10.3760/cma.j.issn.0376-2491.2020.02.006 | Wrong study design |
| Zhang, X., Anandasabapathy, S., Abrams, J., Othman, M., & Badr, H. J. (2021). Lifestyle Risk Factors, Quality of Life, and Intervention Preferences of Barrett’s Esophagus Patients: A Prospective Cohort Study. *Global Advances in Health and Medicine, 10*(101584936), 21649561211001346. https://doi.org/10.1177/21649561211001346 | Wrong study design |
| Zhao, H., Turel, O., Brevers, D., Bechara, A., He, Q., & Aron, A. (2020). Smoking cues impair monitoring but not stopping during response inhibition in abstinent male smokers. *Behavioural Brain Research, 386*. https://doi.org/10.1016/j.bbr.2020.112605 | Follow up < 6 weeks |
| Zhao, S., Chen, F., Wang, D., Wang, H., Han, W., & Zhang, Y. (2019). Effect of preoperative smoking cessation on postoperative pain outcomes in elderly patients with high nicotine dependence. *Medicine, 98*(3), e14209. https://doi.org/10.1097/MD.0000000000014209 | Wrong study design |
| Zhuang, T., Ku, S., Shapiro, L. M., Hu, S. S., Cabell, A., & Kamal, R. N. (2020). A Cost-Effectiveness Analysis of Smoking-Cessation Interventions Prior to Posterolateral Lumbar Fusion. T*he Journal of Bone and Joint Surgery. American Volume, 102*(23), 2032–2042. https://doi.org/10.2106/JBJS.20.00393 | Wrong study design |
| Zhuikova E., Durrant P., Macauley E., Goss H., Goldsmith N., Ioannides C., Marczylo T., & Bailey A. (2019). Monitoring the transition from cigarette smoking to electronic cigarette use: Nicotine intake, psychometric, and clinical outcomes. *British Journal of Clinical Pharmacology, 85*(7), 1627–1628. https://doi.org/10.1111/bcp.13937 | Wrong exposure |
| Zubovic J., Zdravkovic A., & Jovanovic O. (2022). Smoking patterns during COVID-19: Evidence from Serbia. *Tobacco Induced Diseases, 20*(5), 51. https://doi.org/10.18332/tid/148169 | Wrong study design |
| Zuo, Y., Rabinovich, N. E., & Gilbert, D. G. (2017). Negative affect subtypes and craving differentially predict long-term cessation success among smokers achieving initial abstinence. *Psychopharmacology, 234*(5), 761–771. https://doi.org/10.1007/s00213-016-4509-1 | Mental health measured at baseline only, no follow up |
| Zvolensky, M. J., Bakhshaie, J., Shepherd, J. M., Peraza, N., Garey, L., Viana, A. G., Glover, N., Brown, J. T., & Brown, R. A. (2019). Anxiety sensitivity and smoking among Spanish-speaking Latinx smokers. *Addictive Behaviors, 90*, 55–61. https://doi.org/10.1016/j.addbeh.2018.10.022 | Wrong study design |
| Zvolensky, M. J., Redmond, B. Y., Smit, T., Matoska, C. T., Mayorga, N. A., Gallagher, M., & Garey, L. (2022). Anxiety sensitivity and its association with perceived barriers for quitting, smoking inflexibility, and severity of quit problems among Black smokers. *Journal of Ethnicity in Substance Abuse*. https://doi.org/10.1080/15332640.2022.2129538 | Wrong exposure |
| Zvolensky, M. J., Shepherd, J. M., Clausen, B. K., Garey, L., Redmond, B. Y., & Asfar, T. (2023). Perceived racial/ethnic discrimination in relation to smoking abstinence expectancies among adult Latinx smokers in the United States. *Addictive Behaviors, 140*, 107627. https://doi.org/10.1016/j.addbeh.2023.107627 | Wrong study design |
| Zvolensky, M. J., Garey, L., Allan, N. P., Farris, S. G., Raines, A. M., Smits, J. A. J., Kauffman, B. Y., Manning, K., & Schmidt, N. B. (2018). Effects of anxiety sensitivity reduction on smoking abstinence: An analysis from a panic prevention program. *Journal of Consulting and Clinical Psychology, 86*(5), 474–485. https://doi.org/10.1037/ccp0000288 | Mental health measured at baseline only, no follow up |
| Zvolensky, M. J., Rosenfield, D., Garey, L., Kauffman, B. Y., Langdon, K. J., Powers, M. B., Otto, M. W., Davis, M. L., Marcus, B. H., Church, T. S., Frierson, G. M., Hopkins, L. B., Paulus, D. J., Baird, S. O., & Smits, J. A. J. (2018). Does exercise aid smoking cessation through reductions in anxiety sensitivity and dysphoria? *Health Psychology, 30*(7). https://doi.org/10.1037/hea0000588 | Did not analyse mental health outcomes by exposure of interest |
| Zvolensky, M. J., Bakhshaie, J., Shepherd, J. M., Garey, L., Viana, A. G., Peraza, N., & Asnaani, B. (2020). Anxiety symptoms and smoking among Latinx adult smokers: The importance of sensitivity to internal cues in terms of dependence, barriers for quitting, and quit problems. *Journal of Behavioral Medicine, 43*(1), 88–98. https://doi.org/10.1007/s10865-019-00059-8 | Wrong study design |
| Zvolensky, M. J., Bogiaizian, D., Salazar, P. L., Farris, S. G., & Bakhshaie, J. (2014). An anxiety sensitivity reduction smoking-cessation program for spanish-speaking smokers (Argentina). *Cognitive and Behavioral Practice, 21*(3), 350–363. https://doi.org/10.1016/j.cbpra.2013.10.005 | Did not analyse mental health outcomes by exposure of interest |
| Zvolensky, M. J., Shepherd, J. M., Bakhshaie, J., Garey, L., Viana, A. G., Peraza, N., & Alamilla, A. (2019). Emotion dysregulation and cigarette dependence, perceptions of quitting, and problems during quit attempts among Spanish-speaking Latinx adult smokers. *Addictive Behaviors, 96,* 127–132. https://doi.org/10.1016/j.addbeh.2019.05.002 | Wrong study design |
| Zvolensky, M. J., Shepherd, J. M., Bakhshaie, J., Garey, L., Viana, A. G., Peraza, N., & Aldao, A. (2019). Emotion dysregulation and smoking outcome expectancies among Spanish-speaking Latinx adult cigarette smokers in the United States. *Psychology of Addictive Behaviors, 33*(6), 574–579. https://doi.org/10.1037/adb0000481 | Wrong study design |
| Zvolensky, M. J., Shepherd, J. M., Bakhshaie, J., Peraza, N., Garey, L., Mayorga, N. A., & Berger-Cardoso, J. (2020). Acculturative Stress, Anxiety Sensitivity, and Smoking among Spanish-Speaking Latinx Adult Smokers. *Substance Use & Misuse, 55*(7), 1086–1096. https://doi.org/10.1080/10826084.2020.1729195 | Wrong outcome |
| Zvolensky, M. J., Shepherd, J. M., Clausen, B. K., Garey, L., Kauffman, B. Y., Heggeness, L. F., Viana, A. G., & Bizier, A. (2023). Anxiety symptoms and anxiety sensitivity in relation to cigarette dependence, perceived barriers for smoking cessation and quit problems among adult Latinx smokers. *Journal of Ethnicity in Substance Abuse, 101083217*, 1–21. https://doi.org/10.1080/15332640.2022.2159911 | Wrong study design |
| Zvolensky, M. J., Shepherd, J. M., Clausen, B. K., Garey, L., Redmond, B. Y., Brown, R. A., Bogiaizian, D., Salazar, P. L., Viana, A. G., & Abelson, A. (2022). Anxiety-related constructs and smoking outcome expectancies among Latinx smokers. *Experimental and Clinical Psychopharmacology,* 31(5), 942-952. https://doi.org/10.1037/pha0000625 | Wrong study design |
| Zvorsky, I., Skelly, J. M., & Higgins, S. T. (2018). Effects of financial incentives for smoking cessation on mood and anxiety symptoms among pregnant and newly postpartum women. *Nicotine & Tobacco Research, 20*(5), 620–627. https://doi.org/10.1093/ntr/ntx111 | Did not analyse mental health outcomes by exposure of interest |
| Zwar, N. A., Bunker, J. M., Reddel, H. K., Dennis, S. M., Middleton, S., van Schayck, O. C. P., Crockett, A. J., Hasan, I., Hermiz, O., Vagholkar, S., Xuan, W., & Marks, G. B. (2016). Early intervention for chronic obstructive pulmonary disease by practice nurse and GP teams: A cluster randomized trial. *Family Practice, 33*(6), 663–670. https://doi.org/10.1093/fampra/cmw077 | Wrong study design |
| Zyambo, C. M. (2019). Cigarette smoking among HIV-positive patients in routine clinical care. *Dissertation Abstracts International: Section B: The Sciences and Engineering*, *80*(1-B(E)). | Wrong study design |

**Appendix C**

References for Included Studies

Anthenelli, R. M., Benowitz, N. L., West, R., St Aubin, L., McRae, T., Lawrence, D., Ascher, J., Russ, C., Krishen, A., & Evins, A. (2016). Neuropsychiatric safety and efficacy of varenicline, bupropion, and nicotine patch in smokers with and without psychiatric disorders (EAGLES): A double-blind, randomised, placebo-controlled clinical trial. *The Lancet*, *387*(10037), 2507–2520. https://doi.org/10.1016/S0140-6736%2816%2930272-0

Anthenelli, R. M., Morris, C., Ramey, T. S., Dubrava, S. J., Tsilkos, K., Russ, C., & Yunis, C. (2013). Effects of varenicline on smoking cessation in adults with stably treated current or past major depression: A randomized trial. *Annals of Internal Medicine*, *159*(6), 390–400. https://doi.org/10.7326/0003-4819-159-6-201309170-00005

Avery, N., Kenny, A. M., Kleppinger, A., Brindisi, J., Litt, M. D., & Oncken, C. A. (2014). Effects of varenicline, nicotine or placebo on depressive symptoms in postmenopausal smokers. *The American Journal on Addictions*, *23*(5), 459–465. https://doi.org/10.1111/j.1521-0391.2014.12130.x

Baker, A., Richmond, R., Lewin, T. J., & Kay-Lambkin, F. (2010). Cigarette smoking and psychosis: Naturalistic follow up 4 years after an intervention trial. *Australian and New Zealand Journal of Psychiatry*, *44*(4), 342–350. https://doi.org/10.3109/00048670903489841

Becoña, E., Vázquez, F. L., & del Carmen Mı́guez, M. (2002). Smoking cessation and anxiety in a clinical sample. *Personality and Individual Differences*, *32*(3), 489–494. https://doi.org/10.1016/S0191-8869(01)00050-2

Berlin, I., Chen, H., & Covey, L. S. (2010). Depressive mood, suicide ideation and anxiety in smokers who do and smokers who do not manage to stop smoking after a target quit day. *Addiction*, *105*(12), 2209–2216. https://doi.org/10.1111/j.1360-0443.2010.03109.x

Blalock, J. A., Robinson, J. D., Wetter, D. W., Schreindorfer, L. S., & Cinciripini, P. M. (2008). Nicotine withdrawal in smokers with current depressive disorders undergoing intensive smoking cessation treatment. *Psychology of Addictive Behaviors*, *22*, 122–128. https://doi.org/10.1037/0893-164X.22.1.122

Bock, B. C., Fava, J. L., Gaskins, R., Morrow, K. M., Williams, D. M., Jennings, E., Becker, B. M., Tremont, G., & Marcus, B. H. (2012). Yoga as a complementary treatment for smoking cessation in women. *Journal of Women’s Health*, *21*(2), 240–248. https://doi.org/10.1089/jwh.2011.2963

Bozkurt, N. & Bozkurt, A. I. (2019). Anxiety and depression status in smokers and effects of depression and anxiety on smoking cessation treatment. *Respirology, 24*(Supplement 2), 281–282. https://doi.org/10.1111/resp.13706_47

Bozkurt, N., & Bozkurt, A. I. (2024). Relationship between anxiety and depression with smoking cessation treatments: a follow-up study. *Journal of Substance Use.* https://doi.org/10.1080/14659891.2024.2414832

Bricker, J. B., Watson, N. L., Heffner, J. L., Sullivan, B., Mull, K., Kwon, D., Westmaas, J. L., & Ostroff, J. (2020). A smartphone app designed to help cancer patients stop smoking: results from a pilot randomized trial on feasibility, acceptability, and effectiveness. *JMIR Formative Research, 4*(1), e16652. https://doi.org/10.2196/16652

Busch, A. M., Nederhoff, D. M., Dunsiger, S. I., Japuntich, S. J., Chrastek, M., Adkins-Hempel, M., Rinehart, L. M., & Lando, H. (2021). Chronic care treatment for smoking cessation in patients with serious mental illness: A pilot randomized trial. *BMC Psychiatry*, *21*(1), 104. https://doi.org/10.1186/s12888-021-03113-5

Busch, A. M., Wagener, T. L., Gregor, K. L., Ring, K. T., & Borrelli, B. (2011). Utilizing reliable and clinically significant change criteria to assess for the development of depression during smoking cessation treatment: The importance of tracking idiographic change. *Addictive Behaviors*, *36*(12), 1228–1232.

Busch, A. M., Tooley, E. M., Dunsiger, S., Chattillion, E. A., Srour, J. F., Pagoto, S. L., Kahler, C. W., & Borrelli, B. (2017). Behavioral activation for smoking cessation and mood management following a cardiac event: Results of a pilot randomized controlled trial. *BMC Public Health*, *17*(1), 323. https://doi.org/10.1186/s12889-017-4250-7

Cather, C., Hoeppner, S., Pachas, G., Pratt, S., Achtyes, E., Cieslak, K. M., & Evins, A. E. (2017). Improved depressive symptoms in adults with schizophrenia during a smoking cessation attempt with varenicline and behavioral therapy. *Journal of Dual Diagnosis*, *13*(3), 168–178. https://doi.org/10.1080/15504263.2017.1319585

Catley, D., Ahluwalia, J. S., Resnicow, K., & Nazir, N. (2003). Depressive symptoms and smoking cessation among inner-city African Americans using the nicotine patch. *Nicotine Tob Res*, *5*(1), 61–68.

Cossette, S., Frasure-Smith, N., Robert, M., Chouinard, M. C., Juneau, M., Guertin, M. C., Cournoyer, A., & Mailhot, T. (2011). [A pre assessment for nursing intervention to support tobacco cessation in patients hospitalized for cardiac problems: A pilot study (So-Live)]. *Recherche En Soins Infirmiers*, *105*, 60–75.

Covey, L. S., Hu, M.-C., Winhusen, T., Lima, J., Berlin, I., & Nunes, E. (2015). Anxiety and depressed mood decline following smoking abstinence in adult smokers with attention deficit hyperactivity disorder. *Journal of Substance Abuse Treatment*, *59*, 104–108. https://doi.org/10.1016/j.jsat.2015.07.004

Daley, A., Riaz, M., Lewis, S., Aveyard, P., Coleman, T., Manyonda, I., West, R., Lewis, B., Marcus, B., Taylor, A., Ibison, J., Kent, A., & Ussher, M. (2018). Physical activity for antenatal and postnatal depression in women attempting to quit smoking: Randomised controlled trial. *BMC Pregnancy and Childbirth*, *18*(1), 156.

Dawkins, L., Bauld, L., Ford, A., Robson, D., Hajek, P., Parrott, S., Best, C., Li, J., Tyler, A., Uny, I., & Cox, S. (2020). A cluster feasibility trial to explore the uptake and use of e-cigarettes versus usual care offered to smokers attending homeless centres in Great Britain. *PLoS ONE, 15*, e0240968. https://doi.org/10.1371/journal.pone.0240968

Dedert, E. A., Resick, P. A., Dennis, P. A., Wilson, S. M., Moore, S. D., & Beckham, J. C. (2019). Pilot trial of a combined cognitive processing therapy and smoking cessation treatment. *Journal of Addiction Medicine*, *13*(4), 322–330. https://doi.org/10.1097/ADM.0000000000000502

Farris, S. G., Allan, N. P., Morales, P. C., Schmidt, N. B., & Zvolensky, M. J. (2015). Does successful smoking cessation reduce anxious arousal among treatment-seeking smokers? *Journal of Anxiety Disorders*, *36*, 92–98. https://doi.org/10.1016/j.janxdis.2015.07.009

Field, K., Derella, C., Harris, R., May, K., & Tingen, M. (2021). Depression scores and TNF-alpha in participants of a smoking cessation program. *Journal of Allergy and Clinical Immunology, 147*(Supplement 2), AB82. https://doi.org/10.1016/j.jaci.2020.12.315

Freibott, C. E., Biondi, B. E., Rao, S. R., Blokhina, E., Dugas, J. N., Patts, G., Bendiks, S., Krupitsky, E., Chichetto, N. E., Samet, J. H., Freiberg, M. S., Stein, M. D., & Tindle, H. A. (2024). Is abstinence from alcohol and smoking associated with less anxiety and depressive symptoms among people with HIV? *AIDS and Behaviour, 28,* 1447-1455. https://doi.org/10.1007/s10461-023-04231-9

Garvey, A. J., Kalman, D., Hoskinson, R. A., Jr., Kinnunen, T., Wadler, B. M., Thomson, C. C., & Rosner, B. (2012). Front-loaded versus weekly counseling for treatment of tobacco addiction. *Nicotine & Tobacco Research*, *14*(5), 578–585. https://doi.org/10.1093/ntr/ntr256

Gruder, C. L., Trinidad, D. R., Palmer, P. H., Xie, B., Li, L., & Johnso, C. A. (2013). Tobacco smoking, quitting, and relapsing among adult males in mainland China: The China seven cities study. *Nicotine and Tobacco Research*, *15*(1), 223–230. https://doi.org/10.1093/ntr/nts116

Japuntich, S. J., Hammett, P. J., Rogers, E. S., Fu, S., Burgess, D. J., El Shahawy, O., Melzer, A. C., Noorbaloochi, S., Krebs, P., Sherman, S. E., & Aclet, A. (2020). Effectiveness of proactive tobacco cessation treatment outreach among smokers with serious mental illness. *Nicotine & Tobacco Research, 22*(9), 1433–1438. https://doi.org/10.1093/ntr/ntaa013

Kahler, C. W., Brown, R. A., Ramsey, S. E., Niaura, R., Abrams, D. B., Goldstein, M. G., Mueller, T. I., & Miller, I. W. (2002). Negative mood, depressive symptoms, and major depression after smoking cessation treatment in smokers with a history of major depressive disorder. *Journal of Abnormal Psychology*, *111*, 670–675. https://doi.org/10.1037/0021-843X.111.4.670

Kahler, C. W., Spillane, N. S., Busch, A. M., & Leventhal, A. M. (2011). Time-varying smoking abstinence predicts lower depressive symptoms following smoking cessation treatment. *Nicotine & Tobacco Research*, *13*(2), 146–150.

Kahler, C. W., Spillane, N. S., Day, A. M., Cioe, P. A., Parks, A., Leventhal, A. M., & Brown, R. A. (2015). Positive psychotherapy for smoking cessation: A pilot randomized controlled trial. *Nicotine & Tobacco Research*, *17*(11), 1385–1392. https://doi.org/10.1093/ntr/ntv011

Krebs, P., Rogers, E., Smelson, D., Fu, S., Wang, B., & Sherman, S. (2018). Relationship between tobacco cessation and mental health outcomes in a tobacco cessation trial. *Journal of Health Psychology*, *23*(8), 1119–1128.

Lechner, W. V., Sidhu, N. K., Cioe, P. A., & Kahler, C. W. (2019). Effects of time-varying changes in tobacco and alcohol use on depressive symptoms following pharmaco-behavioral treatment for smoking and heavy drinking. *Drug and Alcohol Dependence*, *194*, 173–177. https://doi.org/10.1016/j.drugalcdep.2018.09.030

Lee, E. J. (2019). Long-term effects of smoking cessation on depressive symptoms, resilience, coping skills, and serotonin. *Psychiatric Quarterly.* https://doi.org/10.1007/s11126-019-09689-2

Lerman, C., Niaura, R., Collins, B. N., Wileyto, P., Audrain-McGovern, J., Pinto, A., Hawk, L., & Epstein, L. H. (2004). Effect of bupropion on depression symptoms in a smoking cessation clinical trial. *Psychol Addict Behav*, *18*(4), 362–366. https://doi.org/10.1037/0893-164X.18.4.362

Levy, D. E., Chang, Y., Regan, S., Tindle, H. A., Singer, D. E., & Rigotti, N. A. (2018). Improvements in health-related quality of life among smokers who quit after hospitalization. *Preventive Medicine*, *110*, 38–46.

Lopez, A. A., Skelly, J. M., & Higgins, S. T. (2015). Financial incentives for smoking cessation among depression-prone pregnant and newly postpartum women: Effects on smoking abstinence and depression ratings. *Nicotine & Tobacco Research*, *17*(4), 455–462. https://doi.org/10.1093/ntr/ntu193

Lubitz, S. F., Flitter, A., Ashare, R. L., Thompson, M., Leone, F., Gross, R., & Schnoll, R. (2019). Improved clinical outcomes among persons with HIV who quit smoking. *AIDS Care Psychological and Socio Medical Aspects of AIDS/HIV.* https://doi.org/10.1080/09540121.2019.1703891

Malte, C. A., Dennis, P. A., Saxon, A. J., McFall, M., Carmody, T. P., Unger, W., & Beckham, J. C. (2015). Tobacco use trajectories among a large cohort of treated smokers with posttraumatic stress disorder. *Addictive Behaviors*, *41*, 238–246. https://doi.org/10.1016/j.addbeh.2014.10.034

Martinez-Vispo, C., Rodriguez-Cano, R., Lopez-Duran, A., Senra, C., del Rio, E. F., & Becona, E. (2019). Cognitive behavioral activation for smoking cessation: Randomized controlled trial. *PLoS ONE, 14*(4), e0214252. [https://doi.org/10.1371/journal. pone.0214252](https://doi.org/10.1371/journal.%20pone.0214252)

Martinez-Vispo, C., del Rio, E. F., Lopez-Duran, A., & Becona, E. (2016). Influence of anxiety sensitivity in a psychological smoking cessation intervention. *Revista de Psicopatologia y Psicologia Clinica*, *21*(1), 11–19.

Mathew, A. R., Robinson, J. D., Norton, P. J., Cinciripini, P. M., Brown, R. A., & Blalock, J. A. (2013). Affective trajectories before and after a quit attempt among smokers with current depressive disorders. *Nicotine & Tobacco Research*, *15*(11), 1807–1815. https://doi.org/10.1093/ntr/ntt036

McDermott, M. S., Marteau, T. M., Hollands, G. J., Hankins, M., & Aveyard, P. (2013). Change in anxiety following successful and unsuccessful attempts at smoking cessation: Cohort study. *British Journal of Psychiatry*, *202*(1), 62–67. https://doi.org/10.1192/bjp.bp.112.114389

Moadel, A. B., Bernstein, S. L., Mermelstein, R. J., Arnsten, J. H., Dolce, E. H., & Shuter, J. (2012). A randomized controlled trial of a tailored group smoking cessation intervention for HIV-infected smokers. *Journal of Acquired Immune Deficiency Syndromes (1999)*, *61*(2), 208–215. https://doi.org/10.1097/QAI.0b013e3182645679

Moreno-Coutiño, A., Ezquerro, C., & Drucker-Colin, R. (2007). Long-term changes in sleep and depressive symptoms of smokers in abstinence. *Nicotine & Tobacco Research : Official Journal of the Society for Research on Nicotine and Tobacco*, *9*, 389–396. https://doi.org/10.1080/14622200701188901

Moss-Alonso, E., Martínez-Vispo, C., López-Durán, A., & Becoña, E. (2024). Does quitting smoking affect depressive symptoms? A longitudinal study based on treatment-seeking smokers with a history of depressive episode. *Int J Ment Health Addiction*. https://doi.org/10.1007/s11469-024-01317-w

Munafò, M. R., Heron, J., & Araya, R. (2008). Smoking patterns during pregnancy and postnatal period and depressive symptoms. *Nicotine & Tobacco Research*, *10*(11), 1609–1602. https://doi.org/10.1080/14622200802412895

Nagawa, C. S., Rigotti, N. A., Chang, Y., Levy, D. E., Streck, J. M., Ylioja, T., Lee, S. S., & Tindle, H. A. (2024). Association between smoking abstinence and depression and anxiety symptoms after hospital discharge: the helping HAND 4 trial. *Journal of Addiction Medicine.* doi: 10.1097/ADM.0000000000001358

Pawlina, M. M., Rondina Rde, C., Espinosa, M. M., & Botelho, C. (2015). Depression, anxiety, stress, and motivation over the course of smoking cessation treatment. *Jornal Brasileiro De Pneumologia: Publicacao Oficial Da Sociedade Brasileira De Pneumologia E Tisilogia*, *41*(5), 433–439.

Rodriguez-Cano, R., Lopez-Duran, A., Del Rio, E. F., Martinez-Vispo, C., Martinez, U., & Becona, E. (2015). Smoking cessation and depressive symptoms at 1-, 3-, 6-, and 12-months follow-up. *Journal of Affective Disorders*, *191*, 94–99. https://doi.org/10.1016/j.jad.2015.11.042

Sankaranarayanan, A., Clark, V., Baker, A., Palazzi, K., Lewin, T. J., Richmond, R., Kay-Lambkin, F. J., Filia, S., Castle, D., & Williams, J. M. (2016). Reducing smoking reduces suicidality among individuals with psychosis: Complementary outcomes from a healthy lifestyles intervention study. *Psychiatry Research*, *243*, 407–412. https://doi.org/10.1016/j.psychres.2016.07.006

Schnoll, R. A., Hitsman, B., Blazekovic, S., Veluz-Wilkins, A., Wileyto, E. P., Leone, F. T., & Audrain-McGovern, J. E. (2016). Longitudinal changes in smoking abstinence symptoms and alternative reinforcers predict long-term smoking cessation outcomes. *Drug and Alcohol Dependence*, *165*, 245–252. https://doi.org/10.1016/j.drugalcdep.2016.06.017

Secades-Villa, R., Gonzalez-Roz, A., Vallejo-Seco, G., Weidberg, S., Garcia-Perez, A., & Alonso-Perez, F. (2019). Additive effectiveness of contingency management on cognitive behavioural treatment for smokers with depression: Six-month abstinence and depression outcomes. *Drug and Alcohol Dependence*, *204*(no pagination), 107495. https://doi.org/10.1016/j.drugalcdep.2019.06.003

Secades-Villa, R., Vallejo-Seco, G., Garcia-Rodriguez, O., Lopez-Nunez, C., Weidberg, S., & Gonzalez-Roz, A. (2015). Contingency management for cigarette smokers with depressive symptoms. *Experimental and Clinical Psychopharmacology*, *23*(5), 351–360. https://doi.org/10.1037/pha0000044

Shuter, J., Morales, D. A., Considine-Dunn, S. E., An, L. C., & Stanton, C. A. (2014). Feasibility and preliminary efficacy of a web-based smoking cessation intervention for HIV-infected smokers: A randomized controlled trial. *Journal of Acquired Immune Deficiency Syndromes (1999)*, *67*(1), 59–66. https://doi.org/10.1097/QAI.0000000000000226

Solomon, L. J., Higgins, S. T., Heil, S. H., Badger, G. J., Mongeon, J. A., & Bernstein, I. M. (2006). Psychological symptoms following smoking cessation in pregnant smokers. *Journal of Behavioral Medicine*, *29*(2), 151–160. https://doi.org/10.1007/s10865-005-9041-4

Spears, C. A., Hedeker, D., Li, L., Wu, C., Anderson, N. K., Houchins, S. C., Vinci, C., Hoover, D. S., Vidrine, J. I., Cinciripini, P. M., Waters, A. J., & Wetter, D. W. (2017). Mechanisms underlying mindfulness-based addiction treatment versus cognitive behavioral therapy and usual care for smoking cessation. *Journal of Consulting and Clinical Psychology*, *85*(11), 1029–1040. https://doi.org/10.1037/ccp0000229

Thompson, M., Schnoll, R., Serrano, K., Leone, F., Gross, R., Collman, R. G., & Ashare, R. L. (2020). The effect of varenicline on mood and cognition in smokers with HIV. *Psychopharmacology, 237,* 1223-1231. https://doi.org/10.1007/s00213-020-05451-w

Tzartzas, K., Chappuis, A., & Clair, C. (2023). Influence of smoking reduction on depressive and anxiety symptoms and quality of life in smokers with type 2 diabetes: a study focusing on the role of gender. *European Psychiatry, 66*(S1), S585-586. doi: 10.1192/j.eurpsy.2023.1223

Vázquez, F. L., & Becoña, E. (1999). Depression and smoking in a smoking cessation programme. *Journal of Affective Disorders*, *55*(2), 125–132. https://doi.org/10.1016/S0165-0327(98)00215-8

Vilardaga, R., Rizo, J., Palenski, P., Mannelli, P., Oliver, J. A., & McClernon, F. J. (2019). Pilot randomized controlled trial of a novel smoking cessation app designed for individuals with co-occurring tobacco dependence and serious mental illness. *Nicotine & Tobacco Research*, *31*, 31.

Weinberger, A. H., Hitsman, B., Papandonatos, G. D., Sacco, K. A., Vessicchio, J. C., & George, T. P. (2009). Predictors of abstinence and changes in psychiatric symptoms in a pooled sample of smokers with schizophrenia receiving combination pharmacotherapy and behavioral therapy for smoking cessation. *Journal of Clinical Psychopharmacology*, *29*(6), 601–603. <https://doi.org/10.1097/JCP.0b013e3181bfd0b4>

Wu, A. D., Gao, M., Aveyard, P., Taylor, G. (2023). Smoking Cessation and Changes in Anxiety and Depression in Adults With and Without Psychiatric Disorders. *JAMA Netw Open, 6*(5), e2316111. doi:10.1001/jamanetworkopen.2023.16111

**Appendix D**

Characteristics of Included Studies, Ordered Alphabetically by Study ID

|  | Anthenelli 2013 |
| --- | --- |
| Method |  |
| Intervention(s) | Pharmacological intervention: Varenicline or placebo.  Behavioural intervention: 10-minute sessions of manual-guided smoking cessation counselling at each visit, up to week 52. |
| Definition(s) of successful quit attempt | Self-reported continuous abstinence, verified by exhaled CO ≤ 10 ppm, for the last four weeks of treatment (weeks 9-12), weeks 9-24, and weeks 9-52.  Self-reported 7-day point-prevalence abstinence, verified by exhaled CO ≤ 10 ppm, at weeks 12, 24, and 52. |
| Definition used for meta-analysis | N/A |
| Length of follow-up(s) | Baseline, weekly during the treatment phase (12 weeks) and weeks 13, 16, 24, 32, 40, and 52. |
| Participants |  |
| Number of participants | 525 (130 successful quitters, 269 unsuccessful quitters) |
| Number included in meta-analysis | N/A |
| Population type | General population |
| Baseline characteristics |  |
| Age *M* (*SD*) | 46.3 (10.8) |
| Sex | 37.3% male, 62.7% female |
| Cigarettes per day *M* (*SD*) | 21.7 (8.1) |
| FTND score *M* (*SD*) | 5.9 (2.0) |
| Outcomes |  |
| Mental health outcomes measured | Depressive symptoms, anxiety symptoms (did not analyse anxiety symptoms by smoking status) |
| Outcome measure(s) | Montgomery-Åsberg Depression Rating Scale (MADRS), Hamilton Rating Scale for Anxiety (HAM-A) |
| Data source | Published data |
| Design |  |
| Study design | Secondary analysis of an RCT |
| Data collection period | 25 March 2010 – 13 June 2012 |
| Country | Bosnia and Herzegovina, Croatia, Germany, Hungary, Romania, Russian Federation, Spain, USA |
| Trial registration ID | NCT01078298 |
| Funding and conflicts of interest | Financial Support: This study was funded by Pfizer. Dr. Anthenelli’s writing of this manuscript was funded, in part, by a Department of Veterans Affairs Merit Review award (NEUA-003-08S) and by a National Institute on Alcohol Abuse and Alcoholism grant (AA019720). Dr. Morris was supported, in part, by grants from the University of California, San Francisco, Smoking Cessation Leadership Center, and Colorado Department of Public Health and Environment. Drs. Ramey, Tsilkos, Russ, and Yunis and Ms. Dubrava are employees of Pfizer. Editorial support was provided by Abegale Templar, PhD, of Engage Scientific and funded by Pfizer.  Potential Conflicts of Interest: Disclosures can be viewed at www.acponline.org/authors/icmje/ConflictOfInterestForms.do?msNum=M13-0777 |

|  | Anthenelli 2016 |
| --- | --- |
| Method |  |
[truncated: 154,880 more chars]
