# Supplementary material for: What if I fail? Unsuccessful smoking cessation attempts and symptoms of depression and anxiety: a systematic review and meta-analysis
Source: BMJ Open. 2025 May 2;15(5):e091419. doi: 10.1136/bmjopen-2024-091419 (PMC12049876; doi:10.1136/bmjopen-2024-091419)
Supplement: Supplementary file 2 [file bmjopen-2024-091419.reviewer_comments.pdf]

PEER REVIEW HISTORY

BMJ Open publishes all reviews undertaken for accepted manuscripts. Reviewers are asked to complete a checklist review form and are provided with free text boxes to elaborate on their assessment. These free text comments are reproduced below.

ARTICLE DETAILS

Title (Provisional)

What If I Fail? Unsuccessful Smoking Cessation Attempts and Symptoms of Depression and Anxiety: A Systematic Review and Meta-Analysis

Authors

Crabb, Amy Bethan; Allen, Jennifer; Taylor, Gemma

VERSION 1 - REVIEW

Reviewer 1

Name Chenoweth, Meghan J.

Affiliation Campbell Family Mental Health Research Institute, Centre for Addiction and Mental Health

Date 21-Aug-2024

COI N/A

This paper seeks to address an important question- whether unsuccessful smoking cessation attempts negatively impact mental health. It often takes numerous failed attempts before an individual achieves long-term smoking cessation and there are strong links between mental health traits (e.g. anxiety and depression) and smoking. I read this paper with great interest. Overall, it is very well written and clear, and the methodology is sound. I have a few relatively minor concerns that should be addressed before I can recommend publication.

- Results section in abstract: “Fifty-six studies were included, representing 30,722”: need to add ‘participants’

- Intro, 3rd paragraph- adding the average # of quit attempts it typically takes to achieve long-term cessation would add impact

- Are all the point prevalence and continuous abstinence measures biochemically verified? I’m wondering about differences between self-report and biochemically verified cessation, but perhaps this is fully addressed in Tables G11 and G12

- Additional subgroup analysis- pharmacotherapy-aided cessation vs behavioural intervention. Smoking cessation aids include bupropion (which is also prescribed as an anti-

depressant), nicotine replacement therapy, and varenicline. Do the outcomes differ as a function of whether pharmacotherapy was used to promote cessation?

- Is there a way to quantify/show the lack of worsening of depression/anxiety symptoms from baseline to failed quit attempt in those who attempted but failed to quit?

- For studies screened by both the initial reviewer and the second reviewer, how many disagreements were there, and why did they occur?

- Did any of the included studies examine sex differences? Is there any indication that post-quit anxiety/depression symptoms differ between men and women?

- Page 13- bottom of results paragraph on anxiety- the last sentence says "…however, an unsuccessful attempt was most often associated with either no change or a reduction in depressive symptoms"; I believe the authors mean anxiety symptoms

**Reviewer**	**2**
**Name**	**Berlin, Ivan**
**Affiliation**	**Hopital Pitie-Salpetriere Service de Pharmacologie, Pharmacology**
**Date**	**02-Sep-2024**
**COI**	**None**

What If I Fail? Unsuccessful Smoking Cessation Attempts and Symptoms of Depression and Anxiety: A Systematic Review and Meta-Analysis. Bmjopen-2024-091419

This manuscript (Ms) aims to investigate comparatively the association between depression and anxiety with successful versus unsuccessful smoking cessation after a quit attempt. The authors run a very extensive literature search and included 56 studies. They report that compared to unsuccessful quit, successful quit of smoking was associated with reduction in symptoms of depression and anxiety. These results confirm findings of previous publications and have relevance for clinical practice.

Comments

Introduction, page 5, 1st paragraph: "Subgroup analyses… changes… more directly related to the quit attempt". More directly than what? This sentence should be rewritten.

Introduction, page 5, 1st paragraph: "… interventions…mitigate negative impacts…" Negative impact of quit or of relapse? This sentence should be rewritten.

Introduction, "In conclusion… the myth of smoking's positive impact": The authors should provide a paragraph in the Introduction about withdrawal symptoms (DSM-V): risk of increased depressive or anxiety symptoms when quit; duration of these symptoms; how to distinguish withdrawal symptoms from those occurring later after quit, i.e. short-term vs

long-term post-quit changes. Smokers when quit report withdrawal symptoms and smoking suppresses these symptoms – the authors should discuss the “benefit” of smoking i.e. smoking suppresses withdrawal symptoms occurring during a short non-smoking period i.e. improves withdrawal symptoms. The wording “myth” is incorrect, smoking improves withdrawal symptoms and this is not a myth.

Page 6, “Exposure”: It seems implicit that dual users of electronic cigarettes (EC) were included but this should be made explicit. Also, pls provide number of studies with EC users and report if a successful quit meant stopping smoking/stopping both EC and smoking.

Page 6, “Outcomes”: The authors should provide here criteria of a successful quit and also the assessment instruments of anxiety and depression symptoms. One can read on page 10, bottom, that

“The most common definition of a successful quit attempt was self-reported point-prevalence abstinence.” Was this the usual 7-day point prevalence abstinence as the measure of smoking cessation? However, this short-term measure of smoking cessation a) may confound with the occurrence of withdrawal symptoms if quit occurred e.g. 7 days earlier; b) does not reflect abstinence for the whole previous period since the quit attempt. How did the authors deal with these confounding issues? What were the lengths of the not smoking periods of time? Pls provide descriptive statistics for this.

Page 7, “Study selection”, line 14: Should one understand that only 25 % of all abstracts were screened by a second reviewer? If yes, what were the criteria of this second lecture of abstract? Same questions for full texts of articles included, line 19-21.

Page 11, first paragraph: “others did not specify a definition of abstinence”. How many did not specify a definition of abstinence? Were these studies, despite lack of abstinence definition included in the analyses?

Page 11, “Interventions”. 38/56 studies included pharmacological interventions. The authors do not seem to having run a sensitivity analysis to estimate the role of pharmacological interventions in post-quit depression or anxiety symptoms. However, some papers used antidepressant medications and one cannot exclude that e.g. nicotine replacement therapy or bupropion, an antidepressant even at the dose of 300 mg/day, improves mood and anxiety indirectly by increasing the likelihood of successful quit or directly of by any other way. The authors should compare the results of studies with and without pharmacological interventions as they do in Tables Gx “Subgroup Analyses”, Appendix G.

“Change in Mental Health”. Both for depression and anxiety, before-after comparisons are not to report; only changes between successful and unsuccessful quitters should be reported and also only statistically significant changes. (Before-after improvements are mainly due to the Hawthorne effect/trial effect/observer effect.) Overall, the description of variability in outcomes by number of studies does not provide substantial information and lengthens the Ms. ; Figures 2 and 3 provide information to the readers. The authors, if they

wish, they could provide the number of studies/total number of studies showing statistically significant differences vs the number of those that did not.

Page 13, paragraph 3, wording such as “more pronounced changes” without showing the data are to avoid all over the Ms.

Page 13 and page 14 “no evidence for subgroup differences… (Table G4)”. Inspection of Table G4 shows that despite the overlapping 95%CI, randomized studies showed differences in anxiety symptoms but non-randomized or observational studies did not. Similarly, inspection of Table G5 shows a clear difference in the general population but not in HIV or Psychotic disorder populations. Overall, the authors cannot state so clearly lack of subgroup differences: in the comparison groups the number of studies are lower, the number of participants probably less, the population more resistant to change (psychotic disorder) etc.

Discussion, page 16: The authors should be aware that one cannot quit without a quit attempt (exception: e.g. acute brain injury). So the overall wording should be successful/unsuccessful quit attempt.

Unsuccessful quit attempt, withdrawal symptoms and their role in failed quit attempts should be discussed.

Line38-39: “categorized participants by smoking outcomes rather than quit attempt”. What is meant here?

Legend for Figures 2 and 3: the wording “quit attempt” should be added also for successful quit, i.e. successfully or unsuccessfully quit after a quit attempt.

**Reviewer**	**3**
**Name**	**Kelson, Zoe**
**Affiliation**	**University of Exeter, Mathematics**
**Date**	**25-Oct-2024**
**COI**	**None**

This review investigated the association between the success/failure of smoking cessation attempts and changes in symptoms of depression and anxiety.

Reviewer comments:

"We updated searches from a previous review using Embase, Medline, and PsycINFO. Metaanalyses summarised the standardised mean difference (SMD) in change in symptoms of depression or anxiety from baseline to follow-up for successful quitters compared to unsuccessful quitters." [Abstract]

Can the Design section in the Abstract please be revised to include the dates of the search, the type of meta-analysis model (i.e. random-effects), and the mode of assessment for heterogeneity, study quality, publication bias, and certainty of evidence?

"Overall, successfully quitting smoking was associated with a decrease in symptoms of depression [SMD=- 0.20, 95%CI: -0.26 to -0.14] and anxiety [SMD=-0.19, 95%CI:-0.31 to - 0.07] compared to an unsuccessful quit attempt." [Abstract]

Can the pooled outcomes in the Results section of the Abstract please report heterogeneity and certainty of evidence?

Can study quality and risk of publication bias outcomes please also be summarised here?

Can the limitations please be further addressed in the 'Strengths and limitations of this study' section?

"This review followed PRISMA and MOOSE reporting guidelines (Brooke et al., 2021; Page et al., 2021)."

Thanks for providing a copy of the PRISMA checklist.

Can a copy of the MOOSE checklist also be provided please?

"The protocol was registered with the international prospective register of systematic reviews (PROSPERO; Crabb et al., 2022). "

The study protocol has been suitably registered.

"Inclusion and exclusion criteria for the initial stage of screening did not differ from two previous reviews (Taylor et al., 2014, 2021; see Appendix A for search strategies)."

This is noted, and important for consistency and comparison.

"Update searches of Embase, Medline, and PsyINFO were conducted from 7th January 2020, the date of the most recent search, to 1st February 2023, to identify any new studies."

Can the literature review please be updated prior to publication?

"Titles and abstracts of all studies were screened by one researcher, 25% were screened by a second reviewer. "

and

"The full texts of articles included at the title and abstract stage were examined by one researcher (AC), 25% were screened by a second reviewer. "

and

" Disagreements were resolved by discussion."

Can the authors please consider an independent researcher reviewing 100% of the articles, to ensure studies are not missed?

"Studies were not excluded based on language; non-English language studies were translated for screening"

Can the authors please consider utilising a translation tool (search as Google Translate, for example) to include non-English language studies?

"For each study, two independent researchers extracted data. Any disagreements were resolved by discussion, involving a third researcher where necessary"

The authors demonstrate that a rigorous data extraction process has been followed.

"Quality assessment was performed using the adapted Newcastle Ottawa Scale (Taylor et al., 2014)"

and

"For each study, quality and risk of bias were assessed using the Newcastle-Ottawa quality assessment scale (NOS), adapted by Taylor and colleagues for use with studies measuring associations between smoking cessation and mental health (Taylor et al., 2014). "

The authors have suitably assessed study quality.

Can the authors please clarify how many authors were independently involved in this assessment process, and how were cases of discordance resolved?

Can the authors please confirm the cut-offs for overall categorisation of study quality into high, moderate and low?

"A generic inverse variance random effects model was used to pool SMDs for each outcome - anxiety and depression - using RevMan (2020)."

An appropriate modelling methodology has been applied.

"Heterogeneity was estimated using I 2 , the percentage of variability explained by differences between studies rather than sampling error. Consistent with the Cochrane Handbook (Higgins et al., 2022), 50% to 90% was considered to represent substantial heterogeneity"

The authors have adequately assessed and quantified statistical heterogeneity.

Can the authors please assess the risk of publication bias?

Can the authors please assess the certainty of evidence for pooled outcomes (e.g. GRADE)?

"Effect estimates and direction of change were compared for studies which scored highly on the adapted Newcastle Ottawa scale and studies with low-to-medium scores. "

The authors have undertaken an insightful subgroup sensitivity analysis based on the assessment of study quality.

"Subgroup Analyses"

A number of useful analyses have been conducted, which are clearly specified and reported.

Can the authors please consider conducting a leave-one-out sensitivity analyses, to explore if any one study may be having an impact on the study inferences?

"On the adapted Newcastle Ottawa Scale, 20 studies obtained a low-to-medium score (≤ 3), 36 studies obtained a score of four or five. "

Can the outcomes of this quality assessment please also be reported in full in the supplementary material?

"Figure 1 PRISMA Flow Diagram, Depicting the Number of Records Identified, Included and Excluded at Each Stage of the Review"

Can this figure please follow the PRISMA template?

"Limitations include the types of data reported. Exclusion often resulted from mental health outcomes not analyzed by cessation success/failure. Many studies collected relevant data but didn't analyze it. Many studies investigated mental health predictors, not cessation impact. The majority were RCTs, but participants weren't randomized by quit attempt success/failure. Only one observational study detailed smoking and depression trajectories, suggesting more research is needed. The review focused on depression and anxiety due to their close association with smoking (Levine et al., 2016; Richardson et al., 2019; Smith et al., 2020). Other outcomes, like post-traumatic stress, quality of life, and suicidality, identified through the review, warrant further exploration in policies for combined smoking and mental health interventions."

Can the authors please also address heterogeneity, study quality, risk of publication bias, and certainty of evidence in the discussion of the study limitations?

VERSION 1 - AUTHOR RESPONSE

Reviewer: 1

Dr. Meghan J. Chenoweth, Campbell Family Mental Health Research Institute, Centre for Addiction and Mental Health

Comments to the Author:
This paper seeks to address an important question- whether unsuccessful smoking cessation attempts negatively impact mental health. It often takes numerous failed attempts before an individual achieves long-term smoking cessation and there are strong links between mental health traits (e.g. anxiety and depression) and smoking. I read this paper with great interest. Overall, it is very well written and clear, and the methodology is sound. I have a few relatively minor concerns that should be addressed before I can recommend publication.

- Results section in abstract: “Fifty-six studies were included, representing 30,722”: need to add ‘participants’
- Intro, 3rd paragraph- adding the average # of quit attempts it typically takes to achieve long-term cessation would add impact

- Are all the point prevalence and continuous abstinence measures biochemically verified? I'm wondering about differences between self-report and biochemically verified cessation, but perhaps this is fully addressed in Tables G11 and G12
- Additional subgroup analysis- pharmacotherapy-aided cessation vs behavioural intervention. Smoking cessation aids include bupropion (which is also prescribed as an anti-depressant), nicotine replacement therapy, and varenicline. Do the outcomes differ as a function of whether pharmacotherapy was used to promote cessation?

Author reply: Thank you for the helpful suggestions. We have addressed the typing errors and added the average number of quit attempts. We have altered the previous subgroup analysis found in Tables G11 and G12, to clarify which studies did and did not include biological verification (Tables H9 and H10). We have added another subgroup analysis considering the impact of antidepressant medication and other pharmacological interventions (Tables H13 and H14).

- Is there a way to quantify/show the lack of worsening of depression/anxiety symptoms from baseline to failed quit attempt in those who attempted but failed to quit?

Author reply:

We appreciate the reviewer's suggestion and recognise the value of quantifying the lack of worsening of depression and anxiety symptoms from baseline to follow-up after a failed quit attempt. We had initially hoped to include such an analysis in our manuscript. However, after extensive consultation with researchers and statisticians, we were unable to identify a robust method for synthesising the data quantitatively due to variability in how outcomes were reported across studies. We also explored the possibility of representing the data visually, but the heterogeneity in the available data rendered these visual representations ineffective and potentially misleading. Despite these challenges, we remain confident that our qualitative synthesis accurately captures the key trends and insights.

- For studies screened by both the initial reviewer and the second reviewer, how many disagreements were there, and why did they occur?

Author reply: We have added the following statement "Inter-rater reliability was 88% for titles and abstracts and 94% for full texts. The majority of disagreements occurred when the analyses of interest were not published and it was unclear whether the data could be obtained by contacting the researchers."

- Did any of the included studies examine sex differences? Is there any indication that post-quit anxiety/depression symptoms differ between men and women?

Author reply: This is an interesting question. We did not extract data on sex differences. Unfortunately, without re-reading all the included papers, we cannot recall whether any of the studies included in our original submission reported data separately by sex; if they did, it must have been a minority. One of the studies identified in the recent update examined sex differences (Tzartzas 2023). We have added these results to the narrative paragraph in the Results section and to the narrative syntheses in Appendices F and G.

- Page 13- bottom of results paragraph on anxiety- the last sentence says "...however, an unsuccessful attempt was most often associated with either no change or a reduction in depressive symptoms"; I believe the authors mean anxiety symptoms

Author reply: Thank you for highlighting this error.

Reviewer: 2

Dr. Ivan Berlin, Hopital Pitie-Salpetriere Service de Pharmacologie, Université de Lausanne Faculté de biologie et médecine

Comments to the Author:

What If I Fail? Unsuccessful Smoking Cessation Attempts and Symptoms of Depression and Anxiety: A Systematic Review and Meta-Analysis. Bmjopen-2024-091419

This manuscript (Ms) aims to investigate comparatively the association between depression and anxiety with successful versus unsuccessful smoking cessation after a quit attempt. The authors run a very extensive literature search and included 56 studies. They report that compared to unsuccessful quit, successful quit of smoking was associated with reduction in symptoms of depression and anxiety. These results confirm findings of previous publications and have relevance for clinical practice.

Comments

Introduction, page 5, 1st paragraph: “Subgroup analyses… changes… more directly related to the quit attempt”. More directly than what? This sentence should be rewritten.

Introduction, page 5, 1st paragraph: “… interventions…mitigate negative impacts…” Negative impact of quit or of relapse? This sentence should be rewritten.

Author reply: Thank you for highlighting this, we have reworded these sentences to provide clarification.

Introduction, “In conclusion… the myth of smoking’s positive impact”: The authors should provide a paragraph in the Introduction about withdrawal symptoms (DSM-V): risk of increased depressive or anxiety symptoms when quit; duration of these symptoms; how to distinguish withdrawal symptoms from those occurring later after quit, i.e. short-term vs long-term post-quit changes. Smokers when quit report withdrawal symptoms and smoking suppresses these symptoms – the authors should discuss the “benefit” of smoking i.e. smoking suppresses withdrawal symptoms occurring during a short non-smoking period i.e. improves withdrawal symptoms. The wording “myth” is incorrect, smoking improves withdrawal symptoms and this is not a myth.

Author reply: We acknowledge that, in editing the paper for publication, we removed some of this information from the introduction. Therefore, the clarity around distinguishing withdrawal symptoms from those occurring later after quit was not provided until the method section (Eligibity criteria - outcomes), “The minimum follow-up of six weeks was chosen to exclude studies that reported data during the tobacco withdrawal period only.” We have added a sentence about withdrawal to the introduction (paragraph 2), aiming to provide clarity whilst keeping the paper concise.

Page 6, “Exposure”: It seems implicit that dual users of electronic cigarettes (EC) were included but this should be made explicit. Also, pls provide number of studies with EC users and report if a successful quit meant stopping smoking/stopping both EC and smoking.

Author reply: Thank you, we have provided clarification on page 6. We did not extract data on EC use unless it formed part of the smoking cessation intervention or was included in the definition of a successful quit attempt. This information can be found in Appendix C:

Characteristics of included studies. One study provided ECs as a cessation intervention (Dawkins, 2020). One study included self-reported EC use at follow-up in their definition of an unsuccessful quit attempt (Busch 2017)

Page 6, “Outcomes”: The authors should provide here criteria of a successful quit and also the assessment instruments of anxiety and depression symptoms. One can read on page 10, bottom, that
“The most common definition of a successful quit attempt was self-reported point-prevalence abstinence.” Was this the usual 7-day point prevalence abstinence as the measure of smoking cessation? However, this short-term measure of smoking cessation a) may confound with the occurrence of withdrawal symptoms if quit occurred e.g. 7 days earlier; b) does not reflect abstinence for the whole previous period since the quit attempt. How did the authors deal with these confounding issues? What were the lengths of the not smoking periods of time? Pls provide descriptive statistics for this.

Author reply:

The criteria for assessment instruments of anxiety and depression symptoms was broad, “any standardised measure”. We have added the most common outcome measures to the Results – Description of Studies (paragraph 2). The assessment instruments used for each study can be found in Appendix C: Characteristics of included studies.

The criteria of a successful quit attempt was included in the previous paragraph (Exposure), although the criteria was broad “as defined by the included studies”. One of the aims of the review was to identify the types of definitions used in the research, so we have included this in the results section rather than the methods. We have slightly edited the sentence you quoted – most studies used either 7-day or 30-day PPA, more detailed information on the definition used in each study can be found in Appendix C. We believe we have avoided the confounding with withdrawal symptoms through our inclusion criteria – follow-up > 6 weeks post quit attempt. We acknowledge that PPA does not reflect abstinence for the whole period since the quit attempt, we have attempted to address this through the sub-analyses in Tables H9 and H10, which compare the different definitions of a successful quit attempt.

Page 7, “Study selection”, line 14: Should one understand that only 25 % of all abstracts were screened by a second reviewer? If yes, what were the criteria of this second lecture of abstract? Same questions for full texts of articles included, line 19-21.

Author reply: Unfortunately, due to the limited resources associated with this project, it was not possible for two independent reviewers to screen 100% of studies. A minimum of 25% titles and abstracts were screened by a second reviewer. A minimum of 25% full texts were screened by a second reviewer. This was done through Covidence software, no criteria were specified, so we believe this was done randomly.

Page 11, first paragraph: “others did not specify a definition of abstinence”. How many did not specify a definition of abstinence? Were these studies, despite lack of abstinence definition included in the analyses?

Author reply: Thank you for this comment, we have edited this section to make it clearer. Some studies that defined a successful quit attempt in terms of self-reported point-prevalence or continuous abstinence, further specified their criteria for the meaning of “abstinence”, i.e. “not even a puff” vs less than five cigarettes. Some studies did not include this additional

information, but did provide an adequate definition of a successful vs unsuccessful quit attempt.

Page 11, “Interventions”. 38/56 studies included pharmacological interventions. The authors do not seem to having run a sensitivity analysis to estimate the role of pharmacological interventions in post-quit depression or anxiety symptoms. However, some papers used antidepressant medications and one cannot exclude that e.g. nicotine replacement therapy or bupropion, an antidepressant even at the dose of 300 mg/day, improves mood and anxiety indirectly by increasing the likelihood of successful quit or directly of by any other way. The authors should compare the results of studies with and without pharmacological interventions as they do in Tables Gx “Subgroup Analyses”, Appendix G.

Author reply: Thank you for this helpful comment. We have added a subgroup analysis comparing studies that included antidepressant medications, studies that used other pharmacological intervention, and no pharmacological intervention (Tables H13 and H14).

“Change in Mental Health”. Both for depression and anxiety, before-after comparisons are not to report; only changes between successful and unsuccessful quitters should be reported and also only statistically significant changes. (Before-after improvements are mainly due to the Hawthorne effect/trial effect/observer effect.) Overall, the description of variability in outcomes by number of studies does not provide substantial information and lengthens the Ms. ; Figures 2 and 3 provide information to the readers. The authors, if they wish, they could provide the number of studies/total number of studies showing statistically significant differences vs the number of those that did not.

Author reply: Thank you for your comment, we have considered this carefully. If we include only the comparison between successful and unsuccessful quitters, this does not address the following question, which is highly relevant: Does an unsuccessful quit attempt carry a risk of worsening depression or anxiety?

We appreciate that this lengthened the Results section, and have summarised the information more concisely, focusing on the information relevant to risk-benefit decisions – i.e. change from baseline to follow-up for unsuccessful quit attempts – rather than including all comparisons.

We acknowledge that describing the data in this way is not ideal. We had initially hoped to include a quantitative analysis, however, after extensive consultation with researchers and statisticians, we were unable to identify a robust method for synthesising the data quantitatively due to variability in how outcomes were reported across studies.

We have acknowledged in the discussion (paragraph 2) that it would be a more helpful comparison to compare those who experience an unsuccessful quit attempt with those who do not attempt to quit smoking, as this would better inform the risk of engaging in a quit attempt.

Page 13, paragraph 3, wording such as “more pronounced changes” without showing the data are to avoid all over the Ms.

Author reply: Thank you for highlighting this, this was taken verbatim from the original paper, so we have added quotation marks.

Page 13 and page 14 “no evidence for subgroup differences… (Table G4)”. Inspection of Table G4 shows that despite the overlapping 95%CI, randomized studies showed differences in

anxiety symptoms but non-randomized or observational studies did not. Similarly, inspection of Table G5 shows a clear difference in the general population but not in HIV or Psychotic disorder populations. Overall, the authors cannot state so clearly lack of subgroup differences: in the comparison groups the number of studies are lower, the number of participants probably less, the population more resistant to change (psychotic disorder) etc.

Author reply: Thank you for highlighting this. Some of the results of the subgroup analyses have shifted with the inclusion of more studies, and we have re-assessed our interpretation of the data.

Discussion, page 16: The authors should be aware that one cannot quit without a quit attempt (exception: e.g. acute brain injury). So the overall wording should be successful/unsuccessful quit attempt.

Unsuccessful quit attempt, withdrawal symptoms and their role in failed quit attempts should be discussed.

Line38-39: “categorized participants by smoking outcomes rather than quit attempt”. What is meant here?

Legend for Figures 2 and 3: the wording “quit attempt” should be added also for successful quit, i.e. successfully or unsuccessfully quit after a quit attempt.

Author reply: Thank you for your comments. We have adjusted wording and provided clarification. We have not discussed the role of withdrawal symptoms, as the review excludes outcomes reported during the withdrawal period (less than six weeks post quit date).

Reviewer: 3

Prof. Zoe Kelson, University of Exeter

Comments to the Author:
This review investigated the association between the success/failure of smoking cessation attempts and changes in symptoms of depression and anxiety.

Reviewer comments:

"We updated searches from a previous review using Embase, Medline, and PsycINFO. Metaanalyses summarised the standardised mean difference (SMD) in change in symptoms of depression or anxiety from baseline to follow-up for successful quitters compared to unsuccessful quitters." [Abstract]
Can the Design section in the Abstract please be revised to include the dates of the search, the type of meta-analysis model (i.e. random-effects), and the mode of assessment for heterogeneity, study quality, publication bias, and certainty of evidence?

"Overall, successfully quitting smoking was associated with a decrease in symptoms of depression [SMD=- 0.20, 95%CI: -0.26 to -0.14] and anxiety [SMD=-0.19, 95%CI:-0.31 to -0.07] compared to an unsuccessful quit attempt." [Abstract]
Can the pooled outcomes in the Results section of the Abstract please report heterogeneity and certainty of evidence?

Can study quality and risk of publication bias outcomes please also be summarised here?

Author reply: Thank you for your comments. We have edited the abstract to include this information, with one exception – we do not have a summary statistic for study quality or risk of publication bias, so have not included these in the Results section of the Abstract, as it would significantly lengthen the word count.

"This review followed PRISMA and MOOSE reporting guidelines (Brooke et al., 2021; Page et al., 2021)."
Thanks for providing a copy of the PRISMA checklist.
Can a copy of the MOOSE checklist also be provided please?

Author reply: We have included a copy of the MOOSE checklist.

"The protocol was registered with the international prospective register of systematic reviews (PROSPERO; Crabb et al., 2022). "
The study protocol has been suitably registered.

"Inclusion and exclusion criteria for the initial stage of screening did not differ from two previous reviews (Taylor et al., 2014, 2021; see Appendix A for search strategies)."
This is noted, and important for consistency and comparison.

"Update searches of Embase, Medline, and PsyINFO were conducted from 7th January 2020, the date of the most recent search, to 1st February 2023, to identify any new studies."
Can the literature review please be updated prior to publication?

Author reply: We have updated the review to include studies up to 2nd January 2025.

"Titles and abstracts of all studies were screened by one researcher, 25% were screened by a second reviewer. "
and
"The full texts of articles included at the title and abstract stage were examined by one researcher (AC), 25% were screened by a second reviewer. "
and
" Disagreements were resolved by discussion."
Can the authors please consider an independent researcher reviewing 100% of the articles, to ensure studies are not missed?

Author reply: We would like to be able to do this. Unfortunately, due to limited resources, it was not possible.

"Studies were not excluded based on language; non-English language studies were translated for screening"
Can the authors please consider utilising a translation tool (search as Google Translate, for example) to include non-English language studies?

Author reply: Non-English language studies were translated for the purposes of screening using Google Translate. We would have included studies published in other languages, however,

there were no non-English language studies that met the criteria for inclusion.

"For each study, two independent researchers extracted data. Any disagreements were resolved by discussion, involving a third researcher where necessary"
The authors demonstrate that a rigorous data extraction process has been followed.

"Quality assessment was performed using the adapted Newcastle Ottawa Scale (Taylor et al., 2014)"
and
"For each study, quality and risk of bias were assessed using the Newcastle-Ottawa quality assessment scale (NOS), adapted by Taylor and colleagues for use with studies measuring associations between smoking cessation and mental health (Taylor et al., 2014). "
The authors have suitably assessed study quality.
Can the authors please clarify how many authors were independently involved in this assessment process, and how were cases of discordance resolved?
Can the authors please confirm the cut-offs for overall categorisation of study quality into high, moderate and low?

Author reply: Thank you, we have clarified this information. We have removed the labels of "high" and "low-to-medium", just citing the scores.

"A generic inverse variance random effects model was used to pool SMDs for each outcome - anxiety and depression - using RevMan (2020)."
An appropriate modelling methodology has been applied.

"Heterogeneity was estimated using I 2 , the percentage of variability explained by differences between studies rather than sampling error. Consistent with the Cochrane Handbook (Higgins et al., 2022), 50% to 90% was considered to represent substantial heterogeneity"
The authors have adequately assessed and quantified statistical heterogeneity.

Can the authors please assess the risk of publication bias?

Author reply: We have now included a section on assessment of publication bias in the methods and results, and funnel plots for each outcome (Tables E3 and E4).

Can the authors please assess the certainty of evidence for pooled outcomes (e.g. GRADE)?

Author reply: Thank you for this suggestion. After exploring the GRADE approach, we believe that this may not be the best way of summarising this information for our review. The GRADE approach has been criticised when used with pooled data for observational studies, as it only allows for RCTs to be rated highly. Although many of our included studies were RCTs by design, the analyses we were interested in did not retain the benefit of randomisation, and so all would be rated low. This formed part of our rationale for using the adapted Newcastle Ottawa Scale, which has overlapping features with GRADE. We hope that this, combined with the funnel plots to assess publication bias, cover what you have asked us to assess.

"Effect estimates and direction of change were compared for studies which scored highly on the adapted Newcastle Ottawa scale and studies with low-to-medium scores. "
The authors have undertaken an insightful subgroup sensitivity analysis based on the assessment of study quality.

"Subgroup Analyses"
A number of useful analyses have been conducted, which are clearly specified and reported.

Can the authors please consider conducting a leave-one-out sensitivity analyses, to explore if any one study may be having an impact on the study inferences?

Author reply: Thank you for this suggestion. Unfortunately, this analysis can only be done manually in RevMan, so we could not produce a figure summarising this information. As expected, some studies had a larger impact than others, however, there was no single study which changed the conclusions/inferences when omitted. Three studies had a disproportionate impact on heterogeneity - we have included a comment at the end of the subgroup analyses in the Results section.

"On the adapted Newcastle Ottawa Scale, 20 studies obtained a low-to-medium score (≤ 3), 36 studies obtained a score of four or five. "
Can the outcomes of this quality assessment please also be reported in full in the supplementary material?

Author reply: Thank you for the helpful suggestion. We have included the criteria for the Newcastle Ottawa Scale (Table E1) and a table showing the quality assessment for all studies (Table E2).

"Figure 1 PRISMA Flow Diagram, Depicting the Number of Records Identified, Included and Excluded at Each Stage of the Review"
Can this figure please follow the PRISMA template?

Author reply: Thank you. In this version, we have used the PRISMA template. Unfortunately, Covidence does not currently have a method of producing separate flowcharts for studies identified via databases and registers and studies identified via other methods, so we were only able to provide combined numbers for the screening process.

"Limitations include the types of data reported. Exclusion often resulted from mental health outcomes not analyzed by cessation success/failure. Many studies collected relevant data but didn't analyze it. Many studies investigated mental health predictors, not cessation impact. The majority were RCTs, but participants weren't randomized by quit attempt success/failure. Only one observational study detailed smoking and depression trajectories, suggesting more research is needed. The review focused on depression and anxiety due to their close association with smoking (Levine et al., 2016; Richardson et al., 2019; Smith et al., 2020). Other outcomes, like post-traumatic stress, quality of life, and suicidality, identified through the review, warrant further exploration in policies for combined smoking and mental health

interventions."
Can the authors please also address heterogeneity, study quality, risk of publication bias, and certainty of evidence in the discussion of the study limitations?

Can the limitations please be further addressed in the 'Strengths and limitations of this study' section?

Author reply: We have added to the study limitations in the Discussion and the "Strengths and limitations" section.

"This review's strength lies in its comprehensive literature identification methods with broad search terms and inclusive screening criteria. Authors were contacted for additional data when not reported useably. There were very few studies with major concerns regarding quality or risk of bias. Although some publication bias is inevitable, it is unlikely to have impacted the conclusions that can be drawn from this review. Limitations include substantial heterogeneity, some of which may be attributed to a few studies. The review was also limited by the types of data reported. Exclusion often resulted from mental health outcomes not analysed by cessation success/failure. Many studies collected relevant data but didn't report the relevant analyses. Many studies investigated mental health symptoms as predictors, not outcomes. The majority were RCTs, but participants were not randomized by quit attempt success/failure. Only two observational studies detailed smoking and depression trajectories, suggesting more research is needed. The review focused on depression and anxiety due to their close association with smoking (Levine et al., 2016; Richardson et al., 2019; Smith et al., 2020). Other outcomes, like post-traumatic stress, quality of life, and suicidality, identified through the review, warrant further exploration in policies for combined smoking and mental health interventions."

Reviewer: 1
If you have selected 'Yes' above, please provide details of any competing interests.: N/A

Reviewer: 2
If you have selected 'Yes' above, please provide details of any competing interests.: None

Reviewer: 3
If you have selected 'Yes' above, please provide details of any competing interests.: None

---

VERSION 2 - REVIEW

Reviewer **1**

Name **Chenoweth, Meghan J.**

Affiliation **Campbell Family Mental Health Research Institute, Centre for Addiction and Mental Health**

Date **14-Feb-2025**

COI

Excellent revision, kudos to the authors.

**Reviewer**	**2**
**Name**	**Berlin, Ivan**
**Affiliation**	**Hopital Pitie-Salpetriere Service de Pharmacologie, Pharmacology**
**Date**	**25-Feb-2025**
**COI**	

The authors correctly answered my comments.

**Reviewer**	**3**
**Name**	**Kelson, Zoe**
**Affiliation**	**University of Exeter, Mathematics**
**Date**	**12-Feb-2025**
**COI**	

Many thanks to the authors for their responses and revisions. The following reviewer requests are raised.

Reviewer previous comment:

Can the authors please consider an independent researcher reviewing 100% of the articles, to ensure studies are not missed?

Author reply:

"We would like to be able to do this. Unfortunately, due to limited resources, it was not possible."

Reviewer request:

Can this please be acknowledged in the discussion of the study limitations?

Author response:

"We have now included a section on assessment of publication bias in the

methods and results, and funnel plots for each outcome (Tables E3 and E4)"

and

"Publication bias was assessed by visual examination of funnel plots, produced using RevMan[30]."

Reviewer request:

Can lines of symmetry please be added to these funnel plots for ease of interpretation?

Can the authors please also consider statistically testing for publication bias, such as applying Egger's, Begg's or Deek's tests?

Reviewer previous comment:

Can the authors please assess the certainty of evidence for pooled outcomes (e.g.

GRADE)?

Author reply:

"Thank you for this suggestion. After exploring the GRADE approach, we

believe that this may not be the best way of summarising this information for our review.

The GRADE approach has been criticised when used with pooled data for observational studies, as it only allows for RCTs to be rated highly. Although many of our included studies were RCTs by design, the analyses we were interested in did not retain the benefit of randomisation, and so all would be rated low. This formed part of our rationale for using the adapted Newcastle Ottawa Scale, which has overlapping features with GRADE. We hope that this, combined with the funnel plots to assess publication bias, cover what you have asked us to assess."

Reviewer request:

GRADE is a rigorous methodology for assessing the certainty of evidence (as outlined in BMJ best practice: https://bestpractice.bmj.com/info/us/toolkit/learn-ebm/what-is-grade/, and in the Cochrane Handbook: https://handbook-5-1.cochrane.org/chapter_12/12_2_1_the_grade_approach.htm), and should ideally be reported alongside all pooled outcomes, even if this implies low certainty of evidence.

Of note, the assessment of certainty of evidence for pooled outcomes is not to be confused with the assessment of study quality for individual articles - both can and should be reported.

Can the authors please revisit this request, and assess and report on certainty of evidence (GRADE) accordingly?

Reviewer request:

Can the authors please consider conducting a leave-one-out sensitivity analyses, to explore if any one study may be having an impact on the study inferences?

Author reply:

"Thank you for this suggestion. Unfortunately, this analysis can only be done

manually in RevMan, so we could not produce a figure summarising this information. As expected, some studies had a larger impact than others, however, there was no single studywhich changed the conclusions/inferences when omitted. Three studies had a

disproportionate impact on heterogeneity - we have included a comment at the end of the subgroup analyses in the Results section."

Reviewer request:

Thanks for manually conducting this leave-one-out analysis. In addition to the summary provided in the text, can the authors please report (tabulate or plot) the outcomes of this analysis in full?

VERSION 2 - AUTHOR RESPONSE

Reviewer: 3
Prof. Zoe Kelson, University of Exeter
Comments to the Author:
Many thanks to the authors for their responses and revisions. The following reviewer requests are raised.

Reviewer previous comment:
Can the authors please consider an independent researcher reviewing 100% of the articles, to ensure studies are not missed?
Author reply:
"We would like to be able to do this. Unfortunately, due to limited resources, it was not possible."
Reviewer request:
Can this please be acknowledged in the discussion of the study limitations?

Author reply: We have added the following statement to the limitations section of the discussion:

"Despite this, there remains a possibility that some relevant studies were not identified, as it was not feasible for all studies to be screened by a second independent reviewer; 25% was chosen as a reasonable target based on other published reviews."

Author response:
"We have now included a section on assessment of publication bias in the methods and results, and funnel plots for each outcome (Tables E3 and E4)"
and
"Publication bias was assessed by visual examination of funnel plots, produced using RevMan[30]."
Reviewer request:
Can lines of symmetry please be added to these funnel plots for ease of interpretation?
Can the authors please also consider statistically testing for publication bias, such as applying Egger's, Begg's or Deek's tests?

Author reply: Thank you for this suggestion. We have added lines of symmetry to the funnel plots. We have applied Egger's test and included Galbraith plots in the Appendices.

“Egger’s test identified weak evidence of small study bias for depressive symptoms, *p* = 0.04 (Figure E5), and no evidence for anxiety symptoms, *p* = 0.31 (Figure E6).”

Reviewer previous comment:
Can the authors please assess the certainty of evidence for pooled outcomes (e.g. GRADE)?
Author reply:
"Thank you for this suggestion. After exploring the GRADE approach, we believe that this may not be the best way of summarising this information for our review. The GRADE approach has been criticised when used with pooled data for observational studies, as it only allows for RCTs to be rated highly. Although many of our included studies were RCTs by design, the analyses we were interested in did not retain the benefit of randomisation, and so all would be rated low. This formed part of our rationale for using the adapted Newcastle Ottawa Scale, which has overlapping features with GRADE. We hope that this, combined with the funnel plots to assess publication bias, cover what you have asked us to assess."
Reviewer request:
GRADE is a rigorous methodology for assessing the certainty of evidence (as outlined in BMJ best practice: https://bestpractice.bmj.com/info/us/toolkit/learn-ebm/what-is-grade/, and in the Cochrane Handbook: https://handbook-5-1.cochrane.org/chapter_12/12_2_1_the_grade_approach.htm), and should ideally be reported alongside all pooled outcomes, even if this implies low certainty of evidence.
Of note, the assessment of certainty of evidence for pooled outcomes is not to be confused with the assessment of study quality for individual articles - both can and should be reported. Can the authors please revisit this request, and assess and report on certainty of evidence (GRADE) accordingly?

Author reply: Thank you for suggesting we revisit this. We have included a statement on the certainty of evidence to the results section and include the GRADE summary table in the appendices.

“The certainty of evidence, as assessed by Grading of Recommendations, Assessment, Development, and Evaluation (GRADE) criteria, was *low* for depressive symptoms and *very low* for anxiety symptoms (Appendix I). Both outcomes began with a *low* rating, as studies were not randomised by the exposure of interest. The evidence for anxiety symptoms was downgraded due to substantial unexplained heterogeneity.”

Reviewer request:
Can the authors please consider conducting a leave-one-out sensitivity analyses, to explore if any one study may be having an impact on the study inferences?
Author reply:
"Thank you for this suggestion. Unfortunately, this analysis can only be done manually in RevMan, so we could not produce a figure summarising this information. As

expected, some studies had a larger impact than others, however, there was no single study which changed the conclusions/inferences when omitted. Three studies had a disproportionate impact on heterogeneity - we have included a comment at the end of the subgroup analyses in the Results section."
Reviewer request:
Thanks for manually conducting this leave-one-out analysis. In addition to the summary provided in the text, can the authors please report (tabulate or plot) the outcomes of this analysis in full?

Author reply: Thank you for this suggestion. We have included a table containing this information for each outcome in the appendices.

“No single study had a disproportionate impact on effect estimates, for either depressive symptoms (Table H15) or anxiety symptoms (Table H16).”

Reviewer: 1
Dr. Meghan J. Chenoweth, Campbell Family Mental Health Research Institute, Centre for Addiction and Mental Health
Comments to the Author:
Excellent revision, kudos to the authors.

Author reply: Thank you, we are very grateful for your feedback, which improved the quality and clarity of our review.

Reviewer: 2
Dr. Ivan Berlin, Hopital Pitie-Salpetriere Service de Pharmacologie, Université de Lausanne Faculté de biologie et médecine
Comments to the Author:
The authors correctly answered my comments.

Author reply: Thank you for your feedback, which has greatly improved the quality and clarity of our review.

Reviewer: 3
If you have selected ‘Yes’ above, please provide details of any competing interests.: Not applicable

Reviewer: 1
If you have selected ‘Yes’ above, please provide details of any competing interests.: Not applicable

Reviewer: 2
If you have selected ‘Yes’ above, please provide details of any competing interests.: Not applicable
